# From Inhibitors to PET: SAR-Based Development of [^18^F]SK60 for mIDH1 Imaging

**DOI:** 10.1021/acs.jmedchem.5c00584

**Published:** 2025-06-29

**Authors:** Sarandeep Kaur, Sladjana Dukic-Stefanovic, Winnie Deuther-Conrad, Magali Toussaint, Barbara Wenzel, Peter Lönnecke, Cornelius K. Donat, Rareş-Petru Moldovan, Klaus Kopka

**Affiliations:** † Helmholtz-Zentrum Dresden-Rossendorf (HZDR), Institute of Radiopharmaceutical Cancer Research, Department of Experimental Neurooncological Radiopharmacy, Research Site Leipzig, Leipzig 04318, Germany; ‡ Helmholtz-Zentrum Dresden-Rossendorf (HZDR), Institute of Radiopharmaceutical Cancer Research, Dresden 01328, Germany; § Technische Universität Dresden, School of Science, Faculty of Chemistry and Food Chemistry, Dresden 01069, Germany; ∥ German Cancer Consortium (DKTK), Partner Site Dresden, Dresden 01307, Germany; ⊥ National Center for Tumor Diseases (NCT), NCT/UCC Dresden, a partnership between DKFZ, Faculty of Medicine and Univ. Hosp. Carl Gustav Carus, TU Dresden & Helmholtz-Zentrum Dresden-Rossendorf (HZDR), Dresden 01307, Germany; # University of Leipzig, Faculty of Chemistry and Mineralogy, Leipzig 04103, Germany

## Abstract

Mutations in isocitrate dehydrogenase 1/2 (mIDH1/2) are clinically significant biomarkers for diagnosis, prognosis, and therapy in cancer. To advance the noninvasive molecular imaging of mIDH1, we aim to develop a positron emission tomography (PET) radiotracer targeting IDH1R132H, the most common type of mIDH1/2. Starting from compound **GSK321**, a systematic structure–activity relationships (SAR) optimization was performed leading to the dimethylated derivative **SK60** (**19**) with low nanomolar potency and high selectivity for IDH1R132H. Consequently, **[** ^**18**^ **F]­SK60** was developed via copper-mediated radiofluorination. Various *in vitro* studies with **[** ^**18**^ **F]­SK60** showed a high fraction of nonspecific binding. The *in vivo* evaluation revealed high metabolic stability with no detectable brain-permeable radiometabolite. In addition, limited brain uptake was observed by PET suggesting that further structural modifications to reduce lipophilicity might be needed for this structure. The present study led thus to a novel series of dimethylated **GSK321** derivatives for further investigation in IDH1R132H-related therapies and PET imaging.

## Graphical abstract

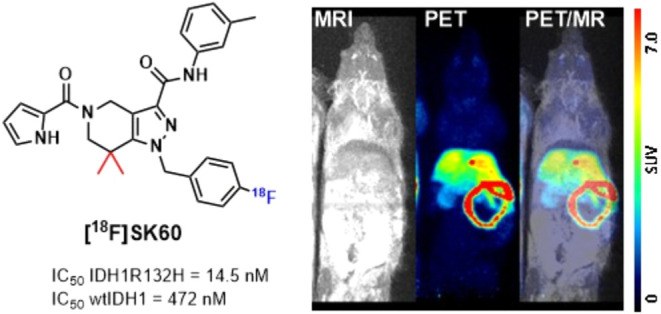

## Introduction

Isocitrate dehydrogenase (IDH) is an important class of enzymes playing an essential role in the tricarboxylic acid (TCA) cycle or Krebs cycle, catalyzing the oxidative decarboxylation of isocitrate to α-ketoglutarate (α-KG) producing CO_2_ and nicotinamide adenine dinucleotide phosphate (NADPH) or NAD­(^+^).^1,2^ It has three isoforms, IDH1, IDH2, and IDH3: IDH1 is located in the cytosol and peroxisome, and IDH2 and IDH3 are located in the mitochondria.^1,2^ In 2008, Parsons et al. reported mutations in IDH1/2 (mIDH1/2) which are found in more than 70% of low-grade gliomas (LGGs)^3^ and up to 20% of higher-grade gliomas (HGGs).^4^ Subsequent studies revealed mutations in mIDH1/2 in 8–20% of acute myeloid leukemia (AML) cases,^5,6^ 10% of cholangiocarcinomas,^7,8^ and chondrosarcomas.^9^ LGGs harbor IDH1R132H, the most common type of mutation mIDH1, accounting for approximately 90% of cases.^4,10^ It is a point mutation at codon 395 when guanine gets replaced with adenine, resulting in the replacement of amino acids of arginine (R) with histidine (H) at position 132.^4^ Other less frequent mutations include IDH1R132C, IDH1R132L, and IDH1R132S.^4^ The mIDH1/2 gains neomorphic activity, converting α-KG into (*D*)-2-hydroxyglutarate (*D*-2-HG) and oxidizing NADPH to NADP^+^.^2,4,11–14^ This results in a significant accumulation of *D*-2-HG (5–30 mM) in the cytoplasm.^2,4,11–14^ *D*-2-HG is structurally similar to α-KG, resulting in competitive inhibition of α-KG-dependent enzymes, such as EglN prolyl hydroxylases.^2,11–15^ The effects of *D*-2-HG as an *″oncometabolite”* are well described in recent reviews and studies.^2,11–15^

Several independent studies showed that brain tumor patients with mIDH1/2-positive gliomas have a more favorable prognosis, including prolonged overall survival (OS) and progression-free survival (PFS).^16–18^ Consequently, mIDH1/2 is considered a therapeutic, prognostic, and diagnostic biomarker. Therefore, numerous mIDH1/2 inhibitors have been developed, out of which more than ten were evaluated in clinical trials, with some approved by the FDA (**AG-881**, **AG-221**, **AG-120**, **FT-2102**) (Figure 1).^4,19–21^ In response to the prognostic importance of mIDH1/2, the WHO included mIDH1/2 biomarkers for the classification of CNS tumors (2016, 2021).^3,22,23^ Hence, the accurate detection of the mIDH1/2 status becomes essential for the diagnosis and classification of patients with gliomas.

**Figure 1:**
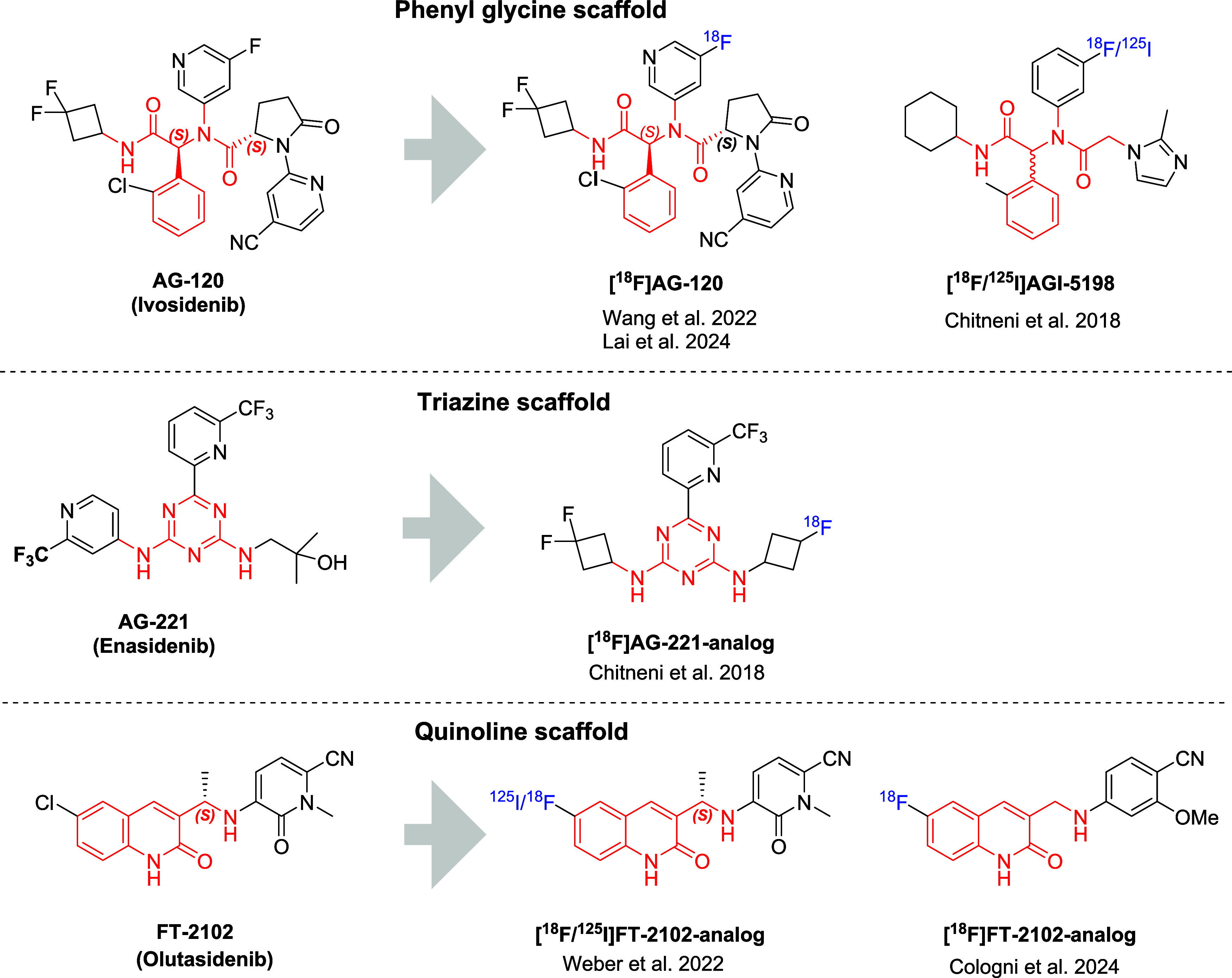
Examples of different mIDH1/2 inhibitors and their respective radiolabeled analogs for noninvasive imaging of mIDH1/2.^37–44^

Currently, the mIDH1/2 detection in clinical settings uses techniques such as immunohistochemistry^24–27^ and gene sequencing,^3,4,27–32^ which requires invasive tissue sampling. Among alternatives, 2-HG-based magnetic resonance spectroscopy (MRS) is under investigation; however, it has certain limitations, such as low specificity for 2-HG, spectral overlap with other endometabolites at typical clinical field strength of MRI (1.5 −3.0 T) which complicate its routine clinical application.^33,34^ To advance the noninvasive detection of mIDH1, we aim to develop a PET radiotracer targeting IDH1R132H. This would reduce the burden of invasive sampling and could be employed repeatedly. Several radiolabeled analogs of known mIDH1/2 inhibitors (Figure 1), have been developed and showed potential, but revealed challenges related to specificity, biodistribution, and metabolic stability.^35^

In this study, **GSK321** (Figure 2), a tetrahydropyrazolopyridine (THPP) based mIDH1 inhibitor with IC_50_ values of 4.6 nM against IDH1R132H and 46 nM against wild-type IDH1 (wtIDH1),^36^ was selected as a lead compound for the development of a radiofluorinated PET radiotracer to target the IDH1R132H-positive tumors. Its nanomolar potency toward IDH1R132H and the presence of the fluorine atom, which provides a position for radiolabeling with fluorine-18 without any structural modification, make it a suitable starting point. However, **GSK321** has only 10-fold selectivity for inhibiting IDH1R132H over wtIDH1, and hence, SAR optimization was required to improve its selectivity. To guide this process, insights from the cocrystal structure of **GSK321** bound to IDH1R132H (PDB ID: 5DE1) were utilized.^36^ Structural analysis revealed that **GSK321** occupies an allosteric site, engaging in key noncovalent interactions within the binding pocket, but does not interact directly with the R132H mutation site or with the NADPH cofactor.^36^ These structural insights, along with the highlighted interactions shown in Figure 2, informed the rational design of analogs of **GSK321**. To achieve an IDH1R132H-specific PET radiotracer starting from **GSK321**, a stepwise synthetic work plan was employed. First, the synthesis of **GSK321** was carried out, and all its stereoisomers were investigated for their inhibitory potency against IDHR132H. Second, our efforts focused on the removal of the chiral center at position *R* ^1^, aiming for a straightforward and simplified synthesis and allowing an efficient preparation with improved atom efficiency of achiral **GSK321** analogs. Third, a systematic SAR was carried out at various subunits (*R* ^2–4^, Figure 2) to increase the selectivity over wtIDH1 and physicochemical properties of the compounds (lipophilicity, molecular weight, number of aromatic rings). This led to the development of **[** ^**18**^ **F]­SK60**, a PET radioligand without stereocenters, that was preclinically evaluated as a potential imaging agent for IDH1R132H.

**Figure 2:**
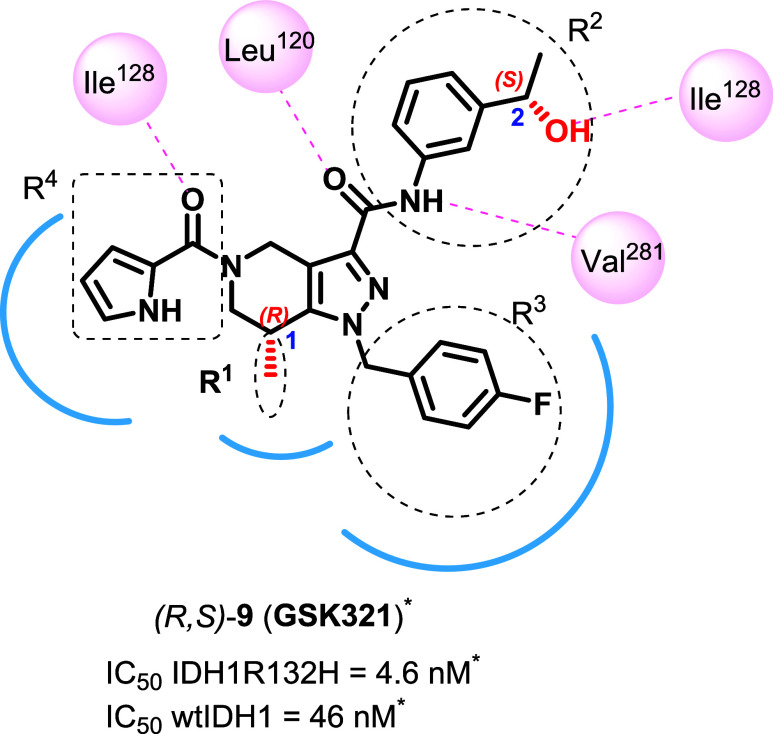
Structure of **GSK321**, here named (R,S)-**9**. SAR studies were performed with modifications at subunits *R* ^1–4^ of **GSK321**. *IC_50_ values reported in the literature.^36^ The key **GSK321**-IDH1R132H (PDB: 5DE1) interactions are also shown.^36^ Key hydrogen bonds are indicated by dotted lines between the inhibitor and pink highlighted residues; hydrophobic interactions are shown in blue. The figure is adapted from the literature.^35^

## Result and Discussion

### Chemistry

#### Synthesis of **GSK321** and Its Stereoisomers

The synthesis of **GSK321** was adapted from the literature (Scheme 1).^36^ Briefly, the synthesis began from the commercially available *tert*-butyl 3-methyl-4-oxopiperidine-1-carboxylate (**1**), which was alkylated with the diethyloxalate in the presence of LDA in THF at −78 °C overnight to afford derivative **2** with a yield of 70% (Scheme 1). Compound **2** underwent cyclic condensation with hydrazine hydrate in acetic acid at room temperature for 2 h to form THPP derivative **3** (Scheme 1). The THPP derivative **3** was subjected to nucleophilic substitution (S_N_2) with *p*-fluorobenzyl bromide in the presence of Cs_2_CO_3_. The resulting ester derivative **4** was treated with 4 N HCl in dioxane at room temperature for 2 h to remove the Boc group, affording the respective ammonium salt (**5**), which was then coupled with the 2-pyrrole carboxylic acid in the presence of pyBOP and *i*-Pr_2_NEt to achieve compound **6** with a yield of 80%. Intermediate **6** was first hydrolyzed with 1 M NaOH in EtOH to give the corresponding carboxylic acid (**7**), which was then coupled with the 3-(1-hydroxyethyl)­aniline (**8**) in the presence of pyBOP as a coupling agent and *i*-Pr_2_NEt to give compound **9** as a mixture of (*R*,*S*)-**9** (**GSK321**), (*S*,*S*)-**9**, (*R*,*R*)-**9** and (*S*,*R*)-**9** (stereoisomeric mixture **I**, Scheme 1).

**Scheme 1:**
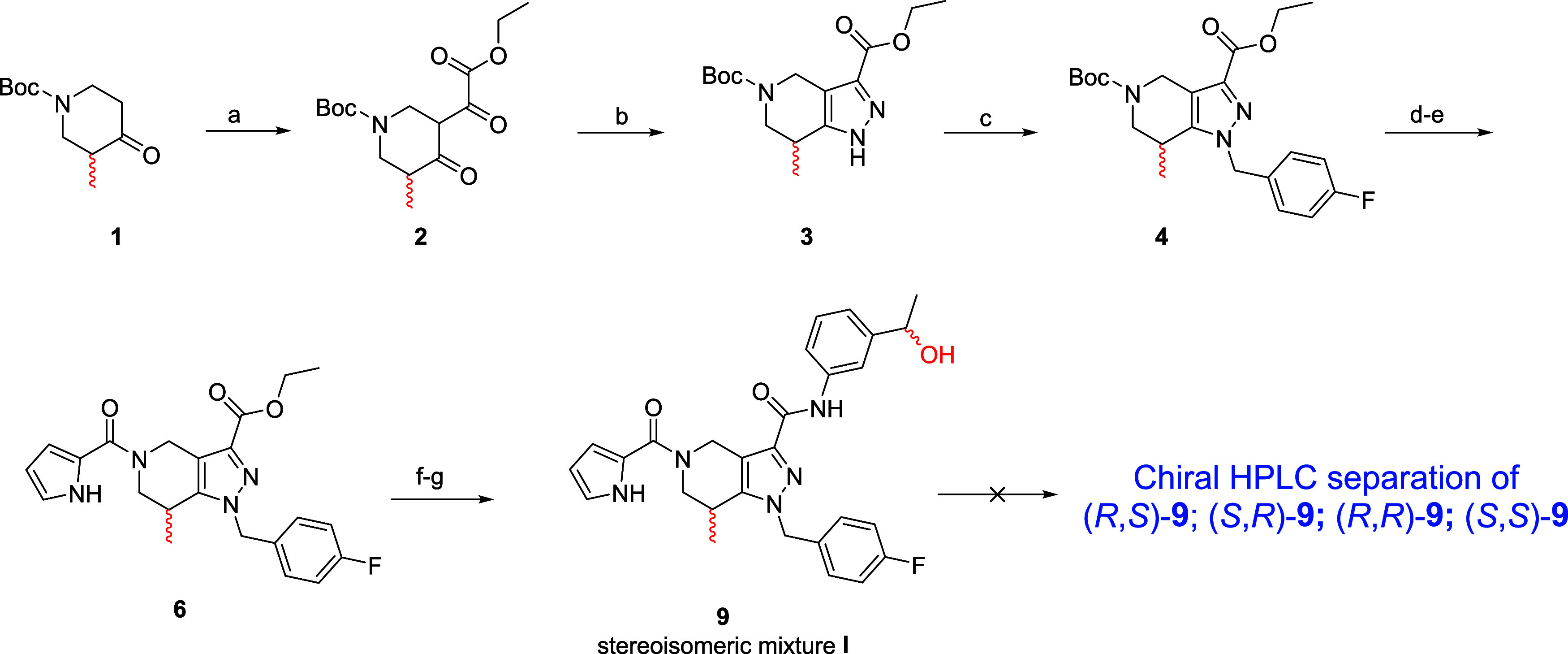
Synthesis of Lead Compound **GSK321** and Its Stereoisomers^a^

a Reagents and conditions: (a) LDA, diethyloxalate, THF, −78 °C, 15 h (yield 70%); (b) N_2_H_4_·H_2_O, AcOH, r.t., 2 h (yield 94%); (c) *p*-fluorobenzyl bromide, Cs_2_CO_3_, THF, r.t., 16 h (yield 76%); (d) 4 N HCl in dioxane, 2 h, r.t. (quantitative); (e) 2-pyrrole carboxylic acid, pyBOP, i-Pr_2_NEt, THF, r.t., 16 h, (yield 80%); (f) 1 M NaOH, EtOH, r.t., 2 h (yield 87%); (g) 3-(1-hydroxyethyl)­aniline (**8**), pyBOP, i-Pr_2_NEt, THF, r.t., 17 h.

With the aim to separate all four stereoisomers by semipreparative HPLC, the stereoisomeric mixture **I** was first investigated by analytical chiral HPLC. Although two different chiral columns were tested in reversed and normal phase mode (Table S2, SI), the isomers could not be separated. Only two peaks were detected (Figure S6, SI). Therefore, to obtain all isomers in pure form, another synthetic route was chosen in which the acid **7** was coupled either with (*S*)-3-(1-hydroxyethyl)­aniline ((*S*)-**8**) or with (*R*)-3-(1-hydroxyethyl)­aniline ((*R*)-**8**) (Figure 3) to provide stereoisomeric mixtures **II** ((*R*,*S*)-**9**, (*S*,*S*)-**9**) and **III** ((*R*,*R*)-**9**, (S,R)-**9**), respectively. The synthesis of (*S*)-**8** and (*R*)-**8** is described in the Supporting Information (Scheme S1, SI). The obtained diastereoisomeric mixtures **II** and **III** were again investigated by analytical chiral HPLC, resulting in the separation of (*R*,*S*)-**9** and (*S*,*S*)-**9** (chromatogram A in Figure 3) as well as (*R*,*R*)-**9** and (*S*,*R*)-**9** (chromatogram B in Figure 3) using a CHIRALPAKIA column in reversed-phase mode. The exact assignment of the peaks and the absolute configuration of the separated isomers could be achieved using a circular dichroism (CD) detector and (*R*,*S*)-**GSK321** as a commercially acquired standard with a known absolute configuration. Circular dichroism is the ability of optically active compounds to differently absorb right and left circularly polarized light. This difference is recorded, and the value is referred to as ellipticity, often given in millidegrees (mdeg). To find the most suitable wavelength for CD monitoring of the stereoisomers of **9**, the corresponding CD spectra were recorded during the chiral HPLC runs in stopped-flow mode. As shown in Figure 3D, the compounds have a strong CD band in the range 260–275 nm. Therefore, 268 nm was chosen as the optimal monitoring wavelength at which (*R*,*S*)-**GSK321** demonstrated a positive maximal ellipticity, resulting in a positive amplitude in the corresponding chromatogram in Figure 3C. As the isomer with *R*,*S*-configuration is part of the stereoisomeric mixture **II**, only peak 1 with a positive ellipticity in the chromatogram in Figure 3A can be assigned to (*R*,*S*)-**9** (**GSK321**) and consequently, peak 2 with a negative amplitude has to be assigned to the *S*,*S*-stereoisomer. As two enantiomers give mirror-image CD spectra, the positive peak 3 in the chromatogram of the stereoisomeric mixture **III** in Figure 3B can be assigned to the *R*,*R*-enantiomer, and peak 4 can be assigned to (*S*,*R*)-**9**.

**Figure 3:**
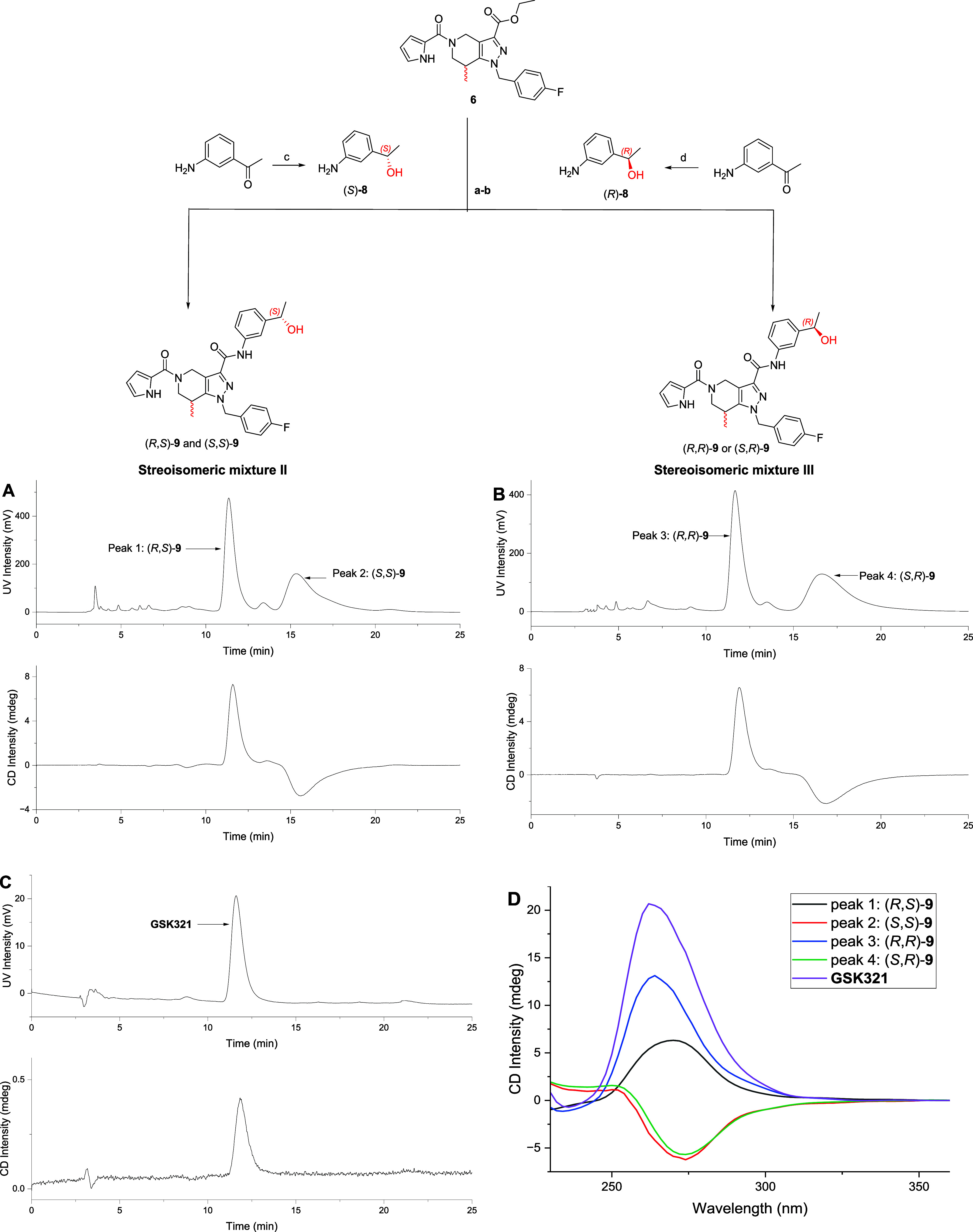
Synthesis of stereoisomeric mixtures II and III and chiral separation of stereoisomers. Reagents and conditions: (a) 1 M NaOH, EtOH, r.t, 2 h (quantitative); (b) (S)-**8** or (R)-**8**, pyBOP, i-Pr_2_NEt, DMF, r.t, 17 h, (15–40%); synthesis of (S)-**8** and (R)-**8**. (c) H_2_, (R)-RUCY-xylBINAP, *t*-BuOK, i-PrOH, r.t, 16 h (yield 69%, e.*e* >99%); (d) H_2_, (S)-RUCY-xylBINAP, *t*-BuOK, i-PrOH, r.t, 16 h (yield 74%, e.*e* >99%). (A–C): Chromatograms of the chiral HPLC separation of stereoisomeric mixture **II** (**A**), **III** (**B**), and **GSK321** (C) using CHIRALPAKIA (250 × 4.6 mm) with 62% of MeCN in aqueous 20 mM NH_4_OAc at a flow rate of 1 mL/min with CD detection at 268 nm. (D): CD spectra of **GSK321** and the stereoisomers of **9** were measured with chiral HPLC in the stopped-flow mode.

Based on the conditions for the analytical chiral HPLC separation, all four stereoisomers were then isolated by semipreparative chiral HPLC, and the enantiomeric purity was determined (Figure S7, SI).

### Synthesis of New Derivatives

Given the challenging analytical separation of **GSK321** stereoisomers, the SAR was initiated first, aiming at the development of derivatives without the stereogenic centers at positions 1 and 2 (Figure 2). Thus, our SAR study started with the modification of *R* ^1^, followed by the modification of *R* ^2^ and *R* ^3^. Previously reported modifications at subunits *R* ^2^ and *R* ^3^ ^36,45^ indicated a possible improvement of inhibitory potency and selectivity toward IDH1R132H. Structural alterations at the pyrrole subunit (*R* ^4^, Figure 2) were performed last, as no SAR data for this position has been reported to date.

#### Structural Modifications at *R* ^1^

Modification at *R* ^1^ was carried out to eliminate the stereocenter at position 1 in **GSK321** by substituting for *R* ^1^ (Figure 2). Okoye-Okafor et al. showed that replacing the CH_3_ group with hydrogen leads to a significant loss in inhibitory potency (**GSK849**, IC_50_ IDH1R132H = 115.1 ± 21.6 nM).^36^ Therefore, a second CH_3_ group was introduced, resulting in dimethylated derivative **21** (Scheme 3). This approach was also tested on **GSK864**,^36^ where the amide group at position 1 was replaced with CH_3_ to get a dimethylated derivative **17** (Scheme 3). This substitution would likely enhance the metabolic stability, as the CH_3_ group is less susceptible to hydrolysis compared to the amide group in **GSK864**. The synthesis of **17** and **21** is depicted in Scheme 2, together with a novel series of dimethylated derivatives with further structural modifications (*R* ^2^). For this, a modified synthetic sequence was developed, starting from *tert*-butyl 3,3-dimethyl-4-oxopiperidine-1-carboxylate (**10**), which was alkylated with diethyloxalate using LDA in THF at −78 °C yielding **11** (Scheme 2). Compound **11** underwent cyclic condensation with hydrazine hydrate to form THPP derivative **12** with a yield of 93%. This was then subjected to S_N_2 with *p*-fluorobenzyl bromide, producing ester derivative **13**. Afterward, the removal of the Boc group (**13**) using HCl yielded ammonium salt **14**, which was coupled with 2-pyrrole carboxylic acid to form **15**. The hydrolysis of **15** gave carboxylic acid **16** with a yield of 80%, which was further coupled with the corresponding amines to produce the final derivatives (Scheme 2).

**Scheme 2:**
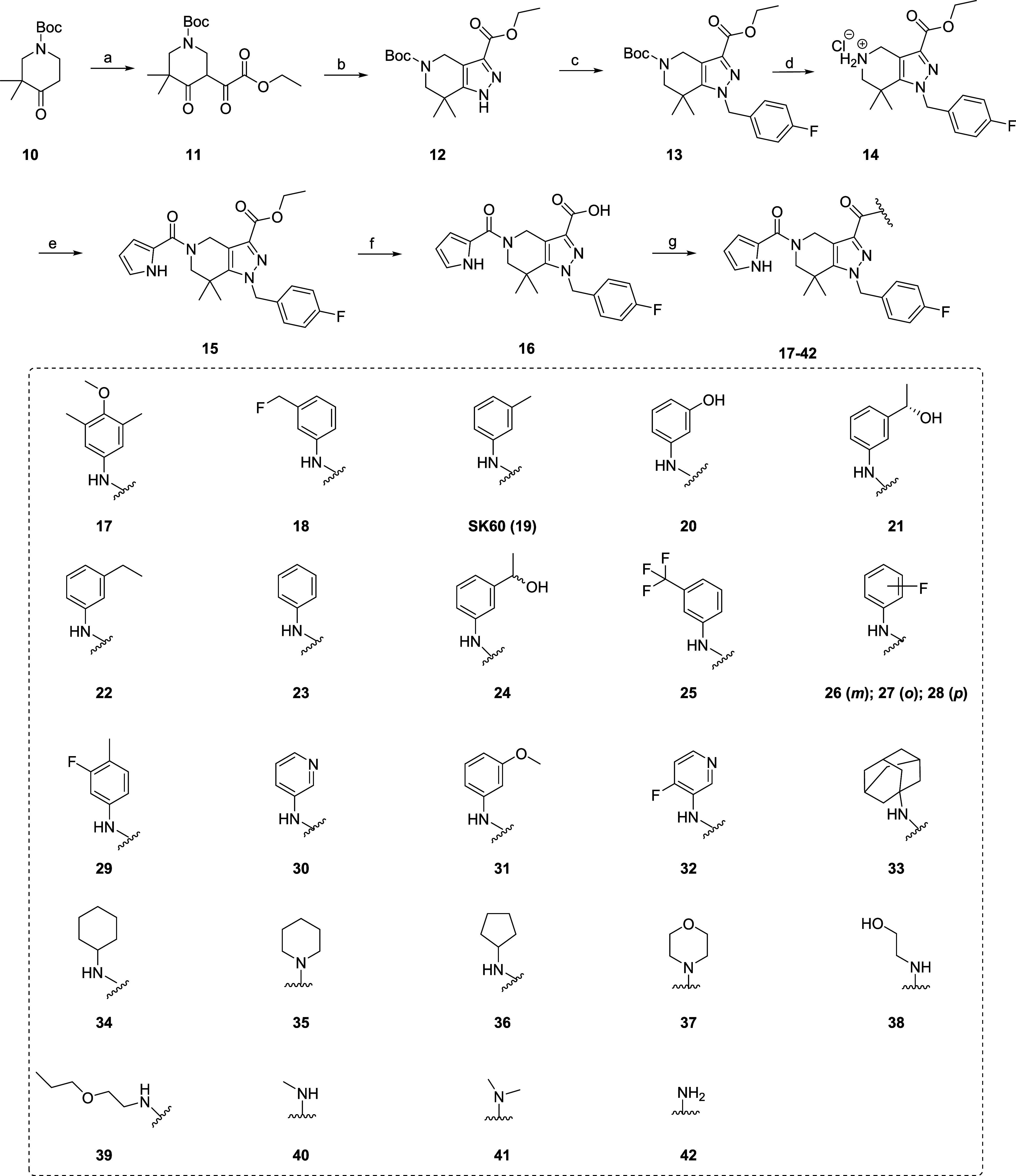
Synthesis and Structures of Derivatives with Modifications at *R* ^1^ and *R* ^2^ ^a^

a Reagents and conditions: (a) LDA, diethyloxalate, THF, −78 °C, 15 h (yield 85%); (b) N_2_H_4_·H_2_O, AcOH, r.t., 2 h (yield 93%); (c) *p*-fluorobenzyl bromide, Cs_2_CO_3_, THF, r.t., 16 h (yield 57%) (d) 4 N HCl in dioxane, 2 h, r.t.; (quantitative) (e) 2-pyrrole carboxylic acid, pyBOP, i-Pr_2_NEt, DMF, r.t., 16 h. (yield 95%); (f) 1 M NaOH, EtOH, r.t., 2 h (yield 80%); (g) alkyl or aryl amine, pyBOP, i-Pr_2_NEt, DMF, r.t., 17 h (yield 26-98%).

#### Structural Modifications at *R* ^2^

Modifications at *R* ^2^ were aimed at modifying the phenyl ring (*R* ^2^) (Figure 2) and to remove the OH group at position 2. Moreover, the tolerance of the introduction of a F atom at *R* ^2^ was also investigated, leading to an alternative ^18^F-labeling position. The final derivatives **17**–**42** were synthesized through the coupling of carboxylic acid **16** with the corresponding amines, as outlined in Scheme 2.

Consequently, a novel series of **GSK321** derivatives was designed by introducing *(i)* (hetero)­aromatic rings (**17–32**), *(ii)* 5–6 membered aliphatic rings (**33–37**), *(iii)* alkyl chains (**33–41**) and *(iv)* hydrogen (**42**) at *R* ^2^ as shown in Scheme 2.

Compounds **30** and **32** were developed by substituting *R* ^2^ with (fluoro)­pyridyl residues as position 2 of pyridine is widely used to introduce the ^18^F-label. This is due to the activation toward S_N_Ar, enabling a facile radiofluorination by substituting leaving groups like NMe_3_^+^, NO_2_, or halogen with [^18^F]­fluoride. To find an alternative position for ^18^F-radiolabeling, fluoroaryl compounds **18**, **25**–**29** (Scheme 2) were synthesized. The effect of substitution at the *ortho*, *meta*, and *para* position on inhibitory potencies was also investigated through the toludinyl derivatives **26** (*meta*), **27** (*ortho*), and **28** (*para*). In derivatives **33**–**42**, the aromatic ring was replaced to evaluate the importance of *π–π* interactions on inhibitory activity and to partially reduce the molecular weight.

#### Structural Modifications at *R* ^3^

In the next step, the 4-fluorobenzyl group (*R* ^3^, Figure 2) was diversely modified, resulting in five new derivatives (**47**–**51**, Scheme 3). A slightly altered synthesis route was applied to compound **SK60** (**19**), which includes the *m*-toluidine moiety from previous modifications at *R* ^2^ (Scheme 3). Briefly, the Boc group in **12** was removed using 4 N HCl in dioxane, yielding ammonium salt **43**, which was coupled with pyrrole carboxylic acid to produce **44**. Afterward, the hydrolysis of **44** generated carboxylic acid **45**, which was further coupled with *m*-toluidine to give **46**. Finally, the alkylation of **46** with a suitable halide led to derivatives **47**–**51** (Scheme 3).

**Scheme 3:**
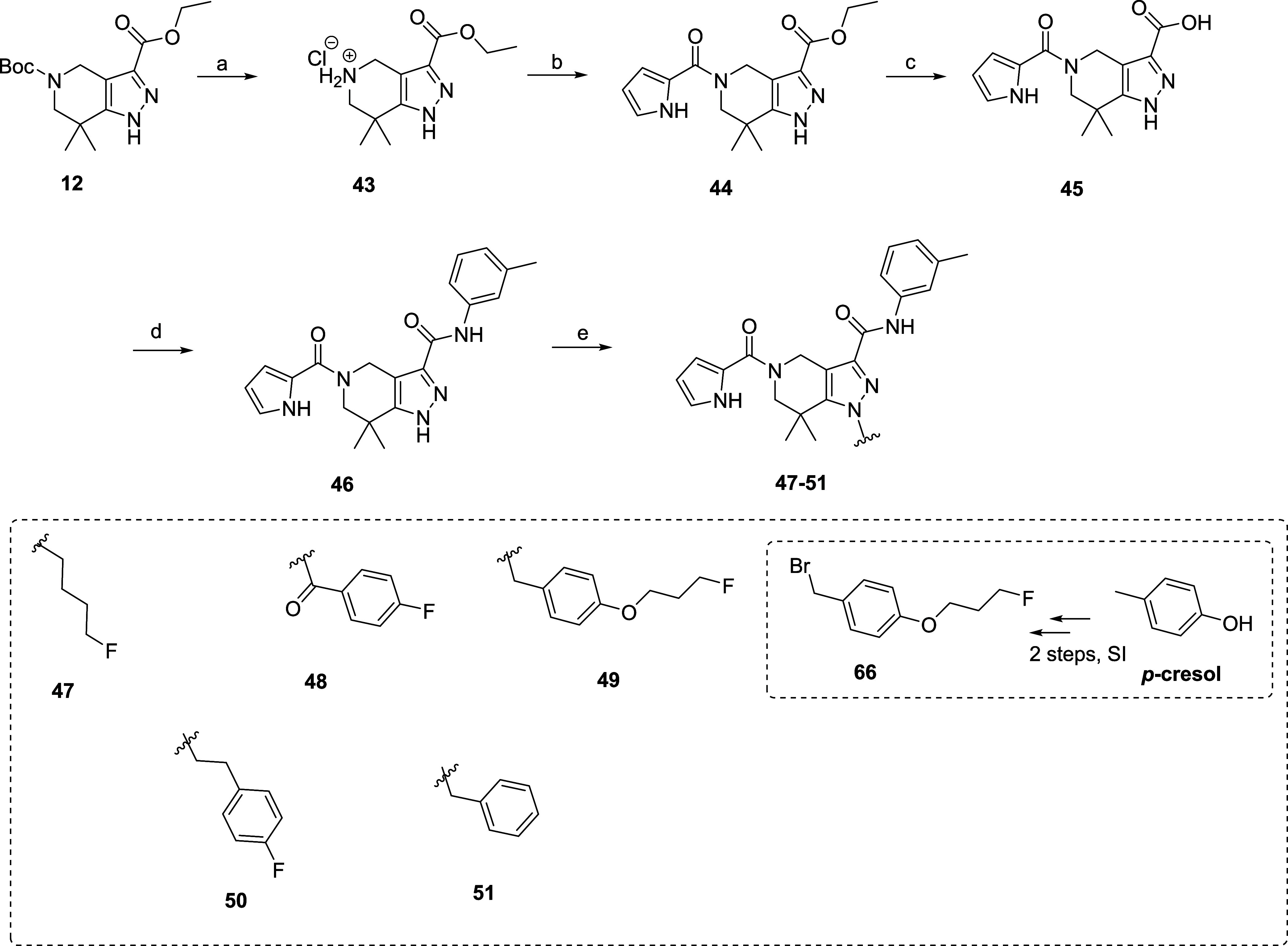
Synthesis and Structures of Derivatives Modified at *R* ^3^ ^a^

a Reagents and conditions: (a) 4 N HCl in dioxane, 2 h, r.t. (quantitative); (b) 2-pyrrole carboxylic acid, pyBOP, i-Pr_2_NEt, DMF, r.t., 16 h. (yield 79%); (c) 1 M NaOH, EtOH, r.t., 2 h (yield 97%); (d) m-toluidine, pyBOP, i-Pr_2_NEt, DMF, r.t., 17 h (yield 61%); (e) alkyl bromide, Cs_2_CO_3_, THF, r.t., 16 h (for **47**, **49**-**51**) (yield 37-81%); *p*-fluorobenzoylchloride, NEt_3_, DMAP, DCM, r.t., 18 h (yield 55%, for **48**).

The aromatic ring at *R* ^3^ was substituted with a fluoroalkyl chain in derivative **47**, which would enable S_N_2-based radiofluorination. Moreover, a 4-fluorobenzaldehyde derivative **48** (Scheme 3) was synthesized to investigate the impact of the carboxyl group, and as the 4-position of benzaldehydes is activated toward S_N_Ar, enabling a facile radiolabeling with fluorine-18. Although fluoropyridine derivatives could facilitate radiofluorination, they were not synthesized due to a reported decrease in inhibitory potency of compounds containing a polar heteroaromatic ring at the *R* ^3^ position.^36^ This polar ring likely disrupts interactions with the lipophilic pocket within the IDH1R132H enzyme.^36^ Consequently, an aryl fluoroalkoxy derivative **49** was synthesized (Scheme 3). Additionally, the effect of introducing another methylene group (**50**) and removal of the fluorine atom on inhibitory potency (**51**) was also investigated (Scheme 3).

#### Structural Modifications at *R* ^4^

Through modifications at *R* ^4^, the impact of the pyrrole carboxamide ring (Figure 2) on the inhibitory potency was evaluated to determine the feasibility of its replacement or removal for higher stability and a potential reduction in molecular weight. Pyrroles are extensively metabolized by cytochrome P450 enzymes, with oxidation preferred on the carbons adjacent to the nitrogen (pyrrole-2-position).^46,47^ Additionally, by replacing the pyrrole moiety with various scaffolds, the compound’s ability to form hydrogen bonding can be modified, which might improve the blood–brain barrier (BBB) passage.^48,49^ Consequently, the influence of the pyrrole moiety at *R* ^4^ on the binding affinity and potency toward IDH1R132H was investigated, by substitution with other heteroaromatic rings (**55**–**59**) and aliphatic chains (**60**–**63**). This is shown in Scheme 4 and allowed for the last step derivatization at the *R* ^4^ position. The ester derivative **13** was first hydrolyzed to get the respective carboxylic acid (**52**), which was coupled with *m*-toluidine in the presence of pyBOP to get derivative **53** with a yield of 79% (Scheme 4). Afterward, derivative **53** was treated with the 4 N HCl in dioxane to remove the Boc group to get the ammonium salt derivative **54**, which was last coupled with the corresponding carboxylic acid to the derivatives **55**–**63**.

**Scheme 4:**
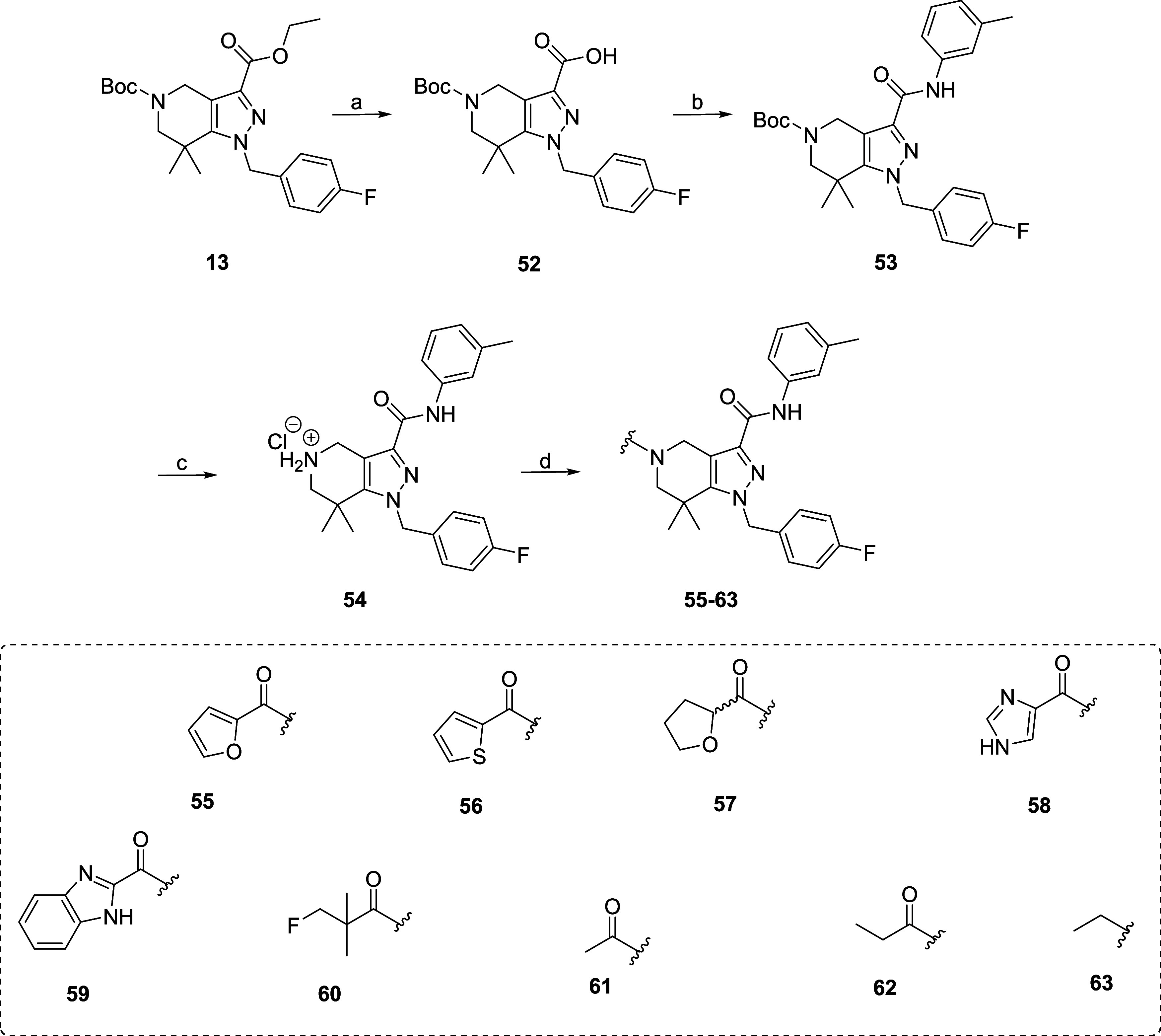
Synthesis and Structures of Derivatives Modified at *R* ^4^ ^a^

a Reagents and conditions: (a) 1 M NaOH, EtOH, r.t., 2 h (yield 96%); (b) m-toluidine, pyBOP, i-Pr_2_NEt, DMF, r.t., 17 h (yield 79%); (c) 4 N HCl in dioxane, 2 h, r.t. (quantitative); (d) RCOOH, pyBOP, i-Pr_2_NEt, DMF, r.t., 16 h (55–59,**61**-**62**) (yield 45-90%); C_2_H_5_I for **63**, Cs_2_CO_3_, THF, r.t., 16 h (yield 30%); 3-fluoro-2,2-dimethylpropanoyl chloride for **60**, NEt_3_, DMAP, DCM, r.t., 18 h (yield 58%).

### *In Vitro* Inhibitory Potency Determination

#### Inhibitory Potencies of GSK321 and Its Stereoisomers

The IC_50_ values of all isolated stereoisomers of **GSK321** were determined and are listed in Table 1. The (*R*,*S*)-**9** (IC_50_ IDH1R132H = 27.4 nM) and (*R*,*R*)-**9** (IC_50_ IDH1R132H = 33.7 nM) have similar inhibitory potencies toward IDH1R132H. On the contrary, their diastereomers [(*S*,*S*)-**9** and (*S*,*R*)-**9**] have shown a loss in the inhibition activity (Table 1), indicating that CH_3_ with (*R*) configuration at position 1 is important for the inhibitory activity.

**Table 1:**
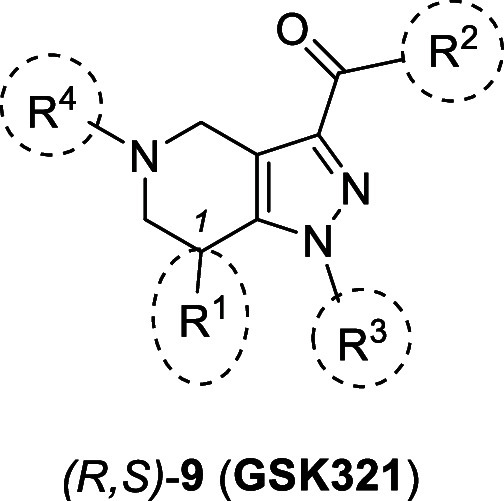

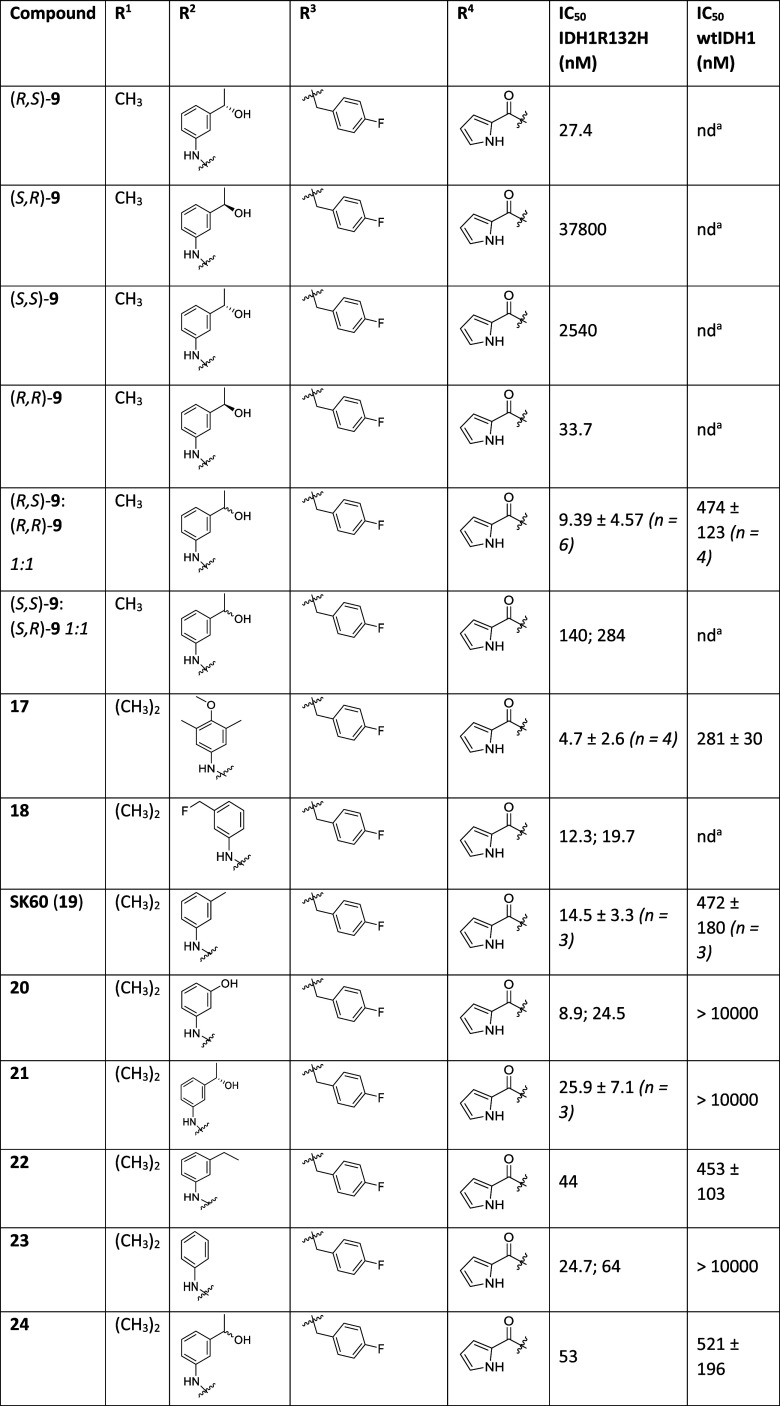

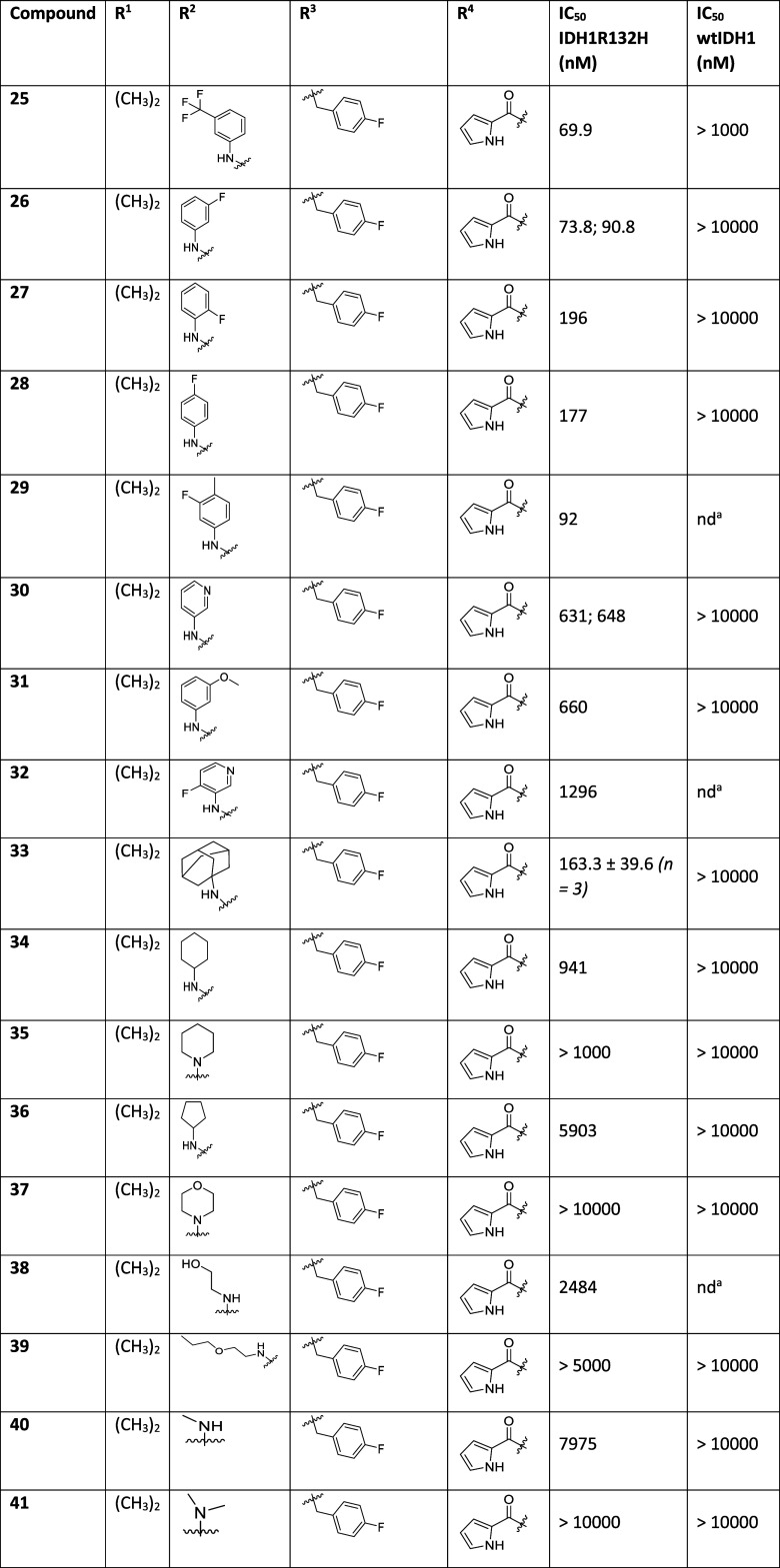

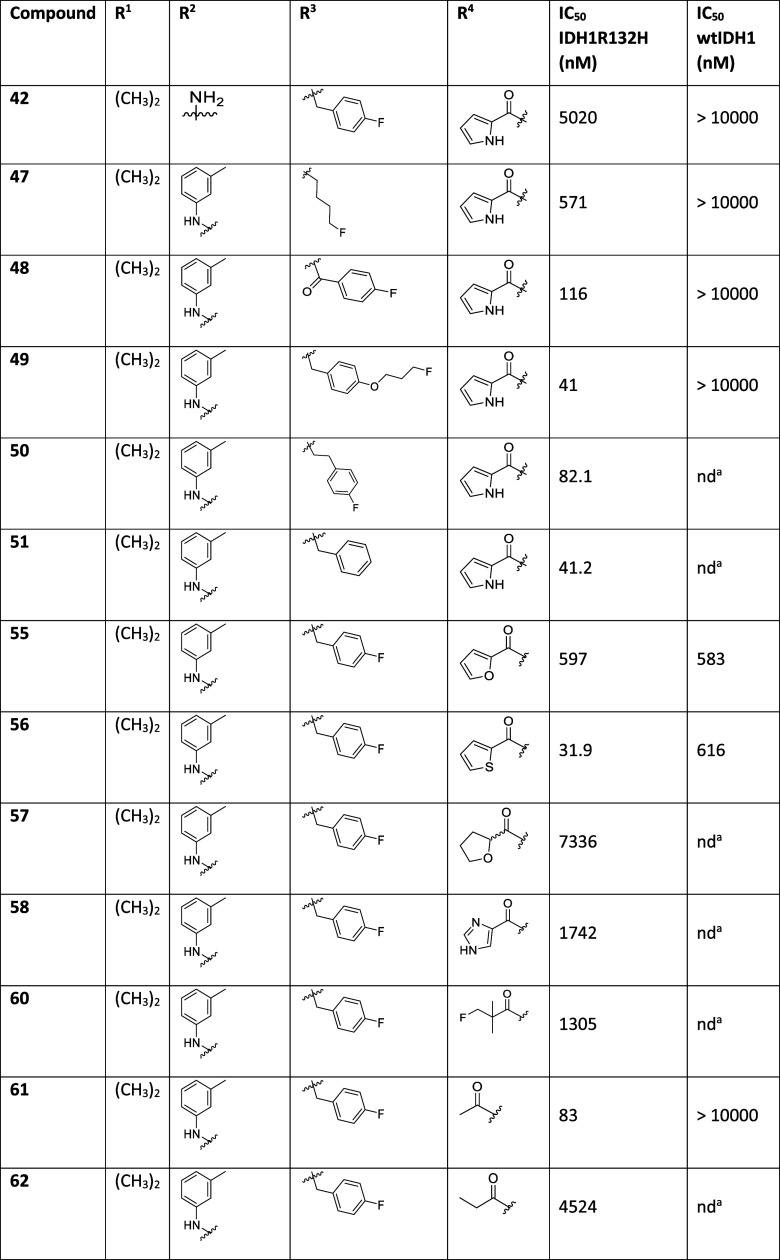
IC_50_ Values of the Developed Derivatives Against IDH1R132H and wtIDH1

a nd, not determined; IC_50_ values for compounds **59**, and **63** were not determined.

#### Inhibitory Potencies of GSK321 Derivatives: SAR

##### SAR Studies on *R* ^1^

The introduction of an additional CH_3_ group at position 1 (Figure 2) gave derivative **21** (IC_50_ IDH1R132H = 25.9 ± 7.1 nM, IC_50_ wtIDH1 >10,000 nM) with improved selectivity and maintained inhibitory potency toward IDH1R132H, as compared to **GSK321** (Table 1). Similarly, when this approach was tested on **GSK864** ^36^ (replacing the amide group with CH_3_ at position 1), it resulted in derivative **17** with enhanced inhibitory potency (IC_50_ IDH1R132H = 4.7 ± 2.5 nM) (Table 1). Overall, the introduction of an additional CH_3_ group at position 1 to eliminate the stereocenter is well-tolerated, both in terms of inhibitory potency and selectivity toward IDH1R132H.

##### SAR Studies on *R* ^2^

The replacement of *(S)*-3-(1-hydroxyethyl)­aniline (*R* ^2^) with its aromatic analogs resulted in derivatives **17–32** (Table 1). Most of these derivatives had inhibitory potencies for IDH1R132H lower than 50 nM (**17–23**) (Table 1). However, the substitution at *R* ^2^ with (fluoro)­pyridyl residues (compounds **30** and **32**) resulted in the loss of inhibitory potency toward IDH1R132H. Among the fluoroaryl compounds **18**, **25**–**29** (Table 1), only the derivative with CH_2_F group (**18**) yielded inhibition in the nanomolar range (IC_50_ IDH1R132H = 12.3 nM), while the substitution with the CF_3_ group in **25** resulted in the decrease of its inhibitory potency (IC_50_ IDH1R132H = 69.9 nM).

To compare the effect of substitution at the *ortho*, *meta*, and *para* position, toludinyl derivatives **26** (*meta*), **27** (*ortho*), and **28** (*para*) were synthesized. Substitution at the *meta*-position resulted in a more potent derivative compared to the *ortho* and *para* positions (Table 1). The introduction of 5–6 membered aliphatic rings at *R* ^2^ (**33–37** in Table 1) resulted in a loss of the inhibitory potency toward IDH1R132H. Among these compounds, only the adamantyl-substituted compound **33** showed inhibitory activity (IC_50_ ∼ 100 nM) for IDH1R132H. Moreover, the substitution with alkyl chains and hydrogen (**38**–**42**) also resulted in a complete loss of inhibitory activity.

Overall, these results showed that the aromatic ring at *R* ^2^ is important for maintaining the inhibitory potency for IDH1R132H. Additionally, the findings suggested the potential to replace this moiety with bulkier and highly lipophilic scaffolds, such as adamantane, without compromising the inhibitory activity. This highlighted the important *π-π* and lipophilic interactions between *R* ^2^ with the enzyme IDH1R132H. Altogether, structural modifications on *R* ^2^ resulted in several derivatives with inhibitory potencies of less than 20 nM as promising candidates (**17**–**20** in Table 1). Among these, compound **SK60** (**19**) was selected as the most suitable derivative for further derivatization at *R* ^3–4^ due to the presence of a metabolically stable *m*-toluidine group at *R* ^2^, in contrast to the more metabolism-prone groups such as methoxy (**17**), benzylic (**18**), and phenolic (**20**).

##### SAR Studies on *R* ^3^

The substitution of the aromatic ring (*R* ^3^, Figure 2) with an alkyl chain in **47** led to a significant loss in inhibitory potency toward IDH1R132H, indicating that a phenyl ring is important for maintaining the inhibitory property at this position (Table 1). To develop derivatives facilitating the incorporation of fluorine-18, 4-fluorobenzaldehyde derivative **48** was synthesized. However, **48** showed reduced inhibitory potency (IC_50_ IDH1R132H = 116 nM) compared to **SK60** (IC_50_ IDH1R132H = 14.5 nM). The fluoroalkoxy derivative **49** (IC_50_ IDH1R132H = 41 nM) exhibited better inhibitory potency than **48** and similar potency to **51** (without the F atom) (Table 1). However, adding another methylene group to **50** reduced its inhibitory potency (IC_50_ IDH1R132H = 82 nM). Despite these derivatives having lower potency than **SK60**, the results provide valuable insights for future optimization efforts.

##### SAR Studies on *R* ^4^

The substitution of the pyrrole ring at *R* ^4^ (Figure 2) with heteroaromatic rings (**55**–**58**) showed that only the thiophene-containing derivative (**56**) is tolerated in terms of inhibitory potency (IC_50_ IDH1R132H = 32 nM) (Table 1). The alkyl substituted derivatives **60**–**62** also show a significant loss of potency, except for **61** with an (IC_50_ IDH1R132H of 83 nM). Overall, the derivatives from SAR studies of *R* ^4^ did not exceed the potency of **SK60**, but these findings could guide future modifications and optimization efforts.

Based on SAR findings (*R* ^1–4^, Figure 2 and Table 1), compounds **17**, **18**, **SK60**, and **20** exhibited the highest (<20 nM) potency against IDH1R132H along with adequate selectivity over wtIDH1, making them potential candidates for ^18^F-labeled PET radiotracer development. Compound **17**, although the most potent, has a high molecular weight (>500 g/mol), elevated lipophilicity (clogP ≈ 5), and a metabolically labile methoxy group, which may result in nonspecific binding *in vivo*. Compound **18** contains a fluoromethyl-aryl moiety suitable for ^18^F-labeling but prone to metabolic defluorination, which would result in undesired bone uptake. Compound **20**, bearing a phenol group, presents a metabolic “soft spot” for oxidation and glucuronidation.^50^ In contrast, **SK60**, with a toluidine residue, offers a favorable balance of potency, selectivity, and metabolic stability, making it the most suitable candidate for radioligand development. Additionally, the inhibitory potency of **SK60** was determined against IDH1R132C, the second most common type of mIDH1/2 and found sufficiently selective over IDH1R132C (IC_50_ IDH1R132C = 509 ± 298 nM, n = 3), supporting its potential utility as a selective radiotracer for IDH1R132H. Before proceeding with the radiolabeling process, an X-ray crystallography study of **SK60** was conducted to understand and confirm the molecular structure (Figure 4). The crystals of **SK60** were grown in MeCN. The detected intra -and intermolecular interactions are shown in Figure S1, SI.

**Figure 4:**
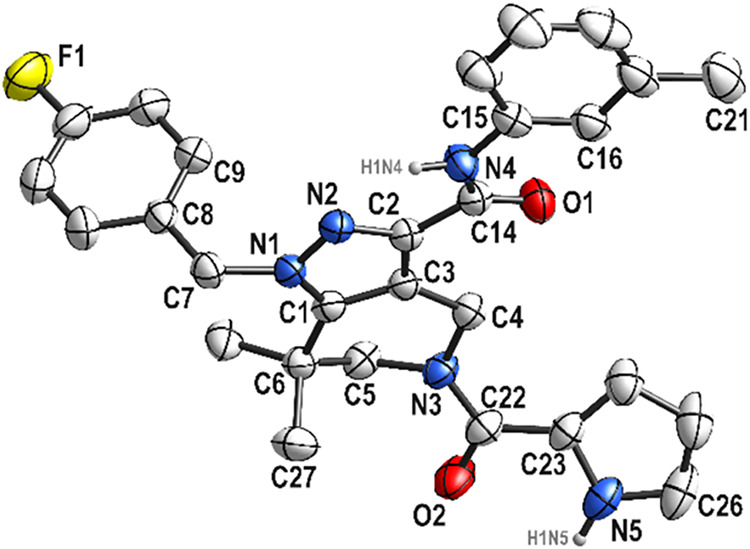
Molecular structure of **SK60**. Hydrogen atoms, except NH, are omitted for clarity. Displacement ellipsoids are drawn at the 30% probability level.

### Radiochemistry

Given that the *p*-fluorobenzyl moiety is not activated for S_N_Ar radiofluorination, copper-mediated radiofluorination (CMRF) was used. The corresponding -Bpin precursor (**64**) was synthesized following the synthetic route outlined in Scheme 5. The optimization of the radiosynthesis for **[** ^**18**^ **F]­SK60** was initiated using parameters that are well-established in the literature.^51–57^ Subsequently, several critical reaction variables were systematically explored to maximize the radiochemical conversion (RCC), including prestirring [Cu­(Py)_4_(OTf)_2_]_2_ (abbreviated as [Cu]) with ^18^F-agent, solvent (combination of DMI vs DMA with *tert*-BuOH *vs n*-BuOH), reaction temperature (110, 120, and 130 °C), drying method of [^18^F]­fluoride (azeotropic drying vs nonazeotropic drying),^54^ and the precursor-to-[Cu] molar ratio, as summarized in Table 2. Radio-HPLC or radio-TLC was used to monitor the reaction at 5, 10, and 15 min.

**Scheme 5:**
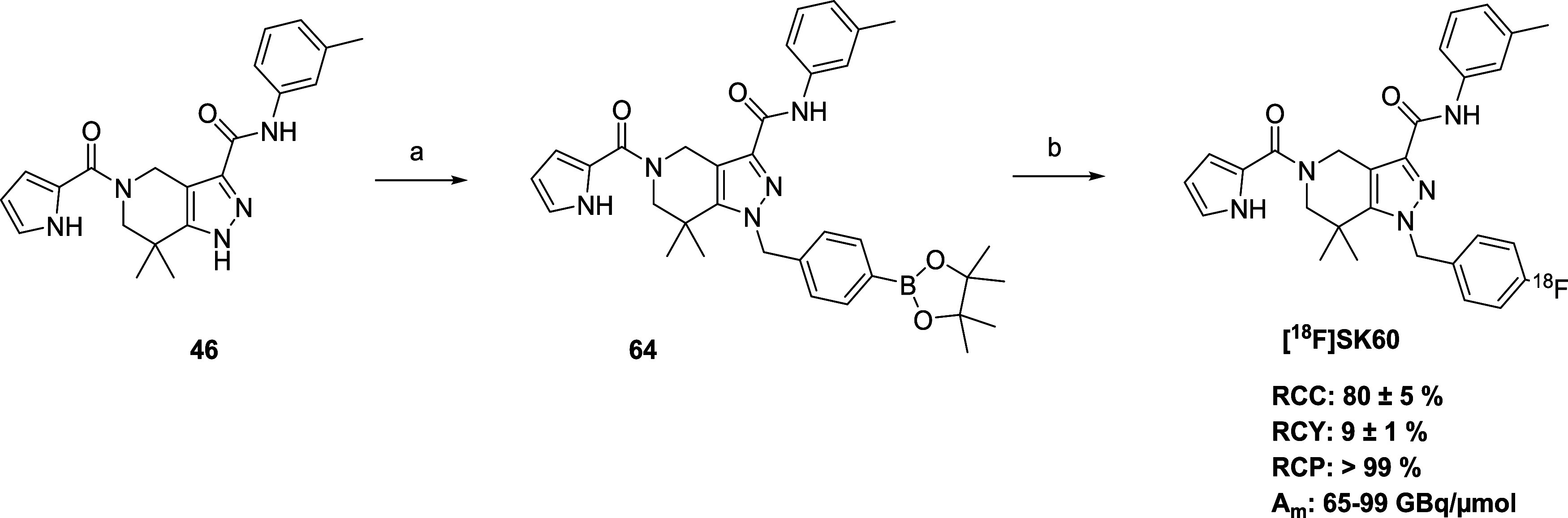
Synthesis of the Precursor **64** and Radiofluorination to Yield **[** ^**18**^ **F]­SK60^a^**

a Reagents and conditions: (a) 4-(Bromomethyl)­phenylboronic ester, Cs_2_CO_3_, THF, r.t., 16 h (yield 48%); (b) [^18^F]­TBAF and [Cu­(Py)_4_(OTf)_2_] (prestirred 2 min), DMI: n-BuOH (2:1), 110 °C, 10 min.

**Table 2:** Reaction Conditions for Optimization of the Manual Radiolabeling of **[** ^**18**^ **F]­SK60**

**drying of** **[** ^**18**^ **F]fluoride**	**PTC**	**prestirring** **(min)**	**solvent** ^a^	**temp**. **(°C)**	**ratio 64 to** **[Cu]** ^g^	**RCC** ^b^ ^c^ **(%)**
*prestirring of [Cu(Py)*_*4*_ *(OTf)*_*2*_ *] with [* ^18^ *F]agent*						
azeotropic	TBAHCO_3_ ^d^	no	DMA: *t*-BuOH (2:1)	110	1:3	3
azeotropic	TBAHCO3	2	DMA: *t*-BuOH (2:1)	110	1:3	21
*Solvent*						
azeotropic	TBAHCO_3_	2	DMA: *t-*BuOH (2:1)	110	1:3	21
azeotropic	TBAHCO_3_	2	DMA: *n*-BuOH (2:1)	110	1:3	35
azeotropic	TBAHCO_3_	2	DMI: *n*-BuOH (2:1)	110	1:3	81
*temperature*						
azeotropic	TBAHCO_3_	2	DMI: *n*-BuOH (2:1)	130	1:3	64
azeotropic	TBAHCO_3_	2	DMI: *n*-BuOH (2:1)	120	1:3	74
azeotropic	TBAHCO_3_	2	DMI: *n*-BuOH (2:1)	110	1:3	81
*molar ratio 64 to [Cu(Py)*_*4*_ *(OTf)*_*2*_ *]* ^*g*^						
azeotropic	TBAHCO_3_	2	DMI: *n*-BuOH (2:1)	110	1:3	81
azeotropic	TBAHCO_3_	2	DMI: *n*-BuOH (2:1)	110	1:4	77
azeotropic	TBAHCO_3_	2	DMI: *n*-BuOH (2:1)	110	1:8	65
*drying process*						
azeotropic	TBAHCO_3_	2	DMI: *n*-BuOH (2:1)	110	1:4	77
nonazeotropic	DMAPHOTf^e^	2	DMI: *n*-BuOH (2:1)	110	1:4	69
nonazeotropic	TBAOTf^f^	2	DMI: *n*-BuOH (2:1)	110	1:4	76

a Solvent mixture (v/v), total reaction volume 900 μL.

b Number of experiments (*n* = 2).

c RCC of **[** ^**18**^ **F]­SK60** was determined by radio-TLC/radio-HPLC of samples taken from the reaction mixture at 10 min.

d 7.50 μmol (100 μL of a 0.075 M solution) TBAHCO_3_ was used.

e 37 μmol (5 mg) DMAPHOTf was used.

f 26 μmol (10 mg) TBAOTf was used.

g Amount of **64** was varied, and [Cu] was kept constant (15 μmol) in the molar ratio of **64** to [Cu].

The prestirring of [Cu­(Py)_4_(OTf)_2_] with ^18^F-agent at room temperature for 2 min, prior to the addition of the precursor **64**, significantly increased RCC from 3% (without prestirring) to 21% (Table 2). Furthermore, changing the solvent mixture from DMA:*t*-BuOH (2:1, v/v) to DMI:*n*-BuOH (2:1, v/v) resulted in a substantial increase of RCC from 21 to 81%, respectively. The effect of temperature on the reaction was investigated by testing 110, 120, and 130 °C. Higher temperatures led to the formation of radiofluorinated byproducts, thereby resulting in the decrease of RCC from 81% at 110 °C to 64% at 130 °C. Additionally, the influence of the molar ratio of precursor **64** to the copper complex was examined by keeping the amount of [Cu] constant. A molar ratio of 1:4 (**64** to [Cu]) resulted in a maintained high RCC as compared to 1:3 (Table 2). However, when the ratio was further increased to 1:8, a slightly reduced RCC of 65% was observed. Although the highest RCC of 81% for **[** ^**18**^ **F]­SK60** was obtained with a molar ratio of 1:3, a molar ratio of 1:4 was ultimately selected as it required less precursor, balancing efficiency, and resource optimization. Regarding the reaction time, for most of the labeling conditions, there was no further increase of RCC found after 10 min.

This optimized manual labeling procedure was successfully transferred to a remotely controlled radiosynthesizer (SynChrom R&D from Elysia-Raytest GmbH, details in the experimental section). For purification of the crude reaction mixture using solid phase extraction (SPE) followed by semipreparative HPLC, 0.4% aqueous TFA was added and stirred at 110 °C for 5 min to hydrolyze the unreacted -Bpin precursor **64** to -B­(OH)_2_. This step was necessary to avoid contamination of the product due to the continuous hydrolysis of **64** on the HPLC column. Notably, the addition of TFA also improved the sorption and overall recovery of **[** ^**18**^ **F]­SK60** on the Sep-Pak C18 plus cartridge during the SPE step. The radiolabeled product was then isolated by semipreparative HPLC at a retention time of about 73 min (Figure S9). The long retention time was required to separate the protodeboronated side product (**51**) from **[** ^**18**^ **F]­SK60** (For detailed information, see Supporting Information S5, SI). For the removal of the HPLC solvent, another SPE was performed, and the radiotracer was formulated in an isotonic saline solution to obtain a final product containing 10% EtOH (v/v) as an injectable solution. Analytical radio-HPLC analysis of the final product coinjected with the reference compound confirmed the identity of **[** ^**18**^ **F]­SK60** (Figure 5). The difference between the retention times of **SK60** (*t*_R_ = 21.36 min) and **[** ^**18**^ **F]­SK60** (*t*_R_ = 21.88 min) corresponds to the delay between UV and radio detection.

**Figure 5:**
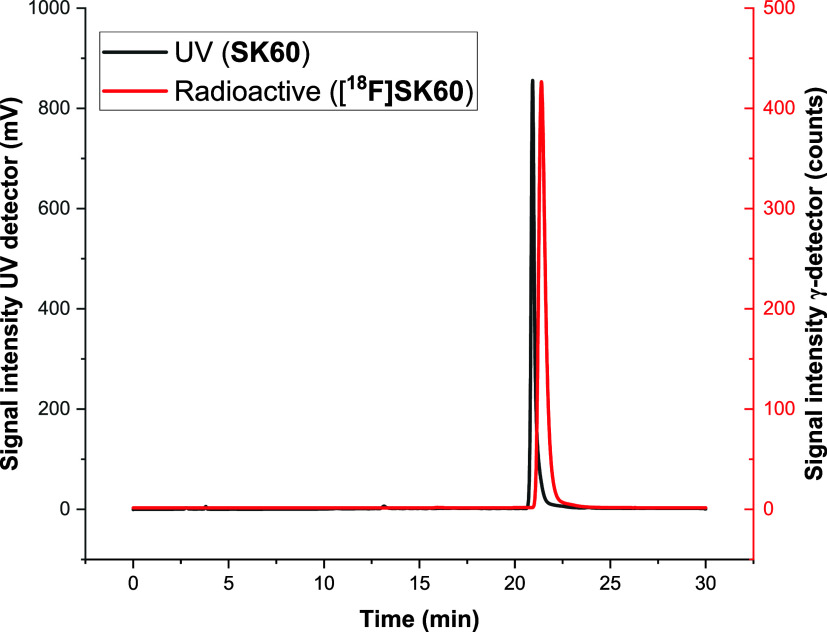
HPLC analysis of formulated product **[** ^**18**^ **F]­SK60** spiked with **SK60**. The difference between the retention times of **SK60** (*t*_R_ = 21.36 min) and **[** ^**18**^ **F]­SK60** (*t*_R_ = 21.88 min) corresponds to the delay between UV and radio detection. HPLC conditions: Nucleodur PFP from Macherey-Nagel, 250 mm × 4.6 mm, 5 μm, 52% MeCN/20 mM NH_4_OAc_aq_, flow 1 mL/min, 268 nm.

Altogether, with this procedure **[** ^**18**^ **F]­SK60** could be obtained with a RCY of 9 ± 1% (EOB, *n* = 10), RCP of more than 99% and A_m_ in the range of 66–99 GBq/μmol (EOS, *n* = 10) at starting activities of 5–6 GBq. The total synthesis time was ∼3 h.

### Determination of log*D*_7.4_

The log*D*_7.4_ value of **[** ^**18**^ **F]­SK60** was experimentally determined by the conventional shake-flask method using *n*-octanol and PBS as the partition system. The measured log*D*_7.4_ value for **[** ^**18**^ **F]­SK60** was 2.3 ± 0.5 (*n* = 4), indicating the potential of passive diffusion through BBB.^58^

### *In Vitro* Studies

Along with inhibitory potency, uptake experiments were performed. As the potency of compound **SK60** to inhibit the IDH1R132H enzyme activity is 35-fold higher than that for the IDH1 enzyme, a difference in uptake in the two cell lines could be expected. However, the data demonstrated a similar pattern for **[** ^**18**^ **F]­SK60** in both cell lines (Figure 6a,b).

**Figure 6:**
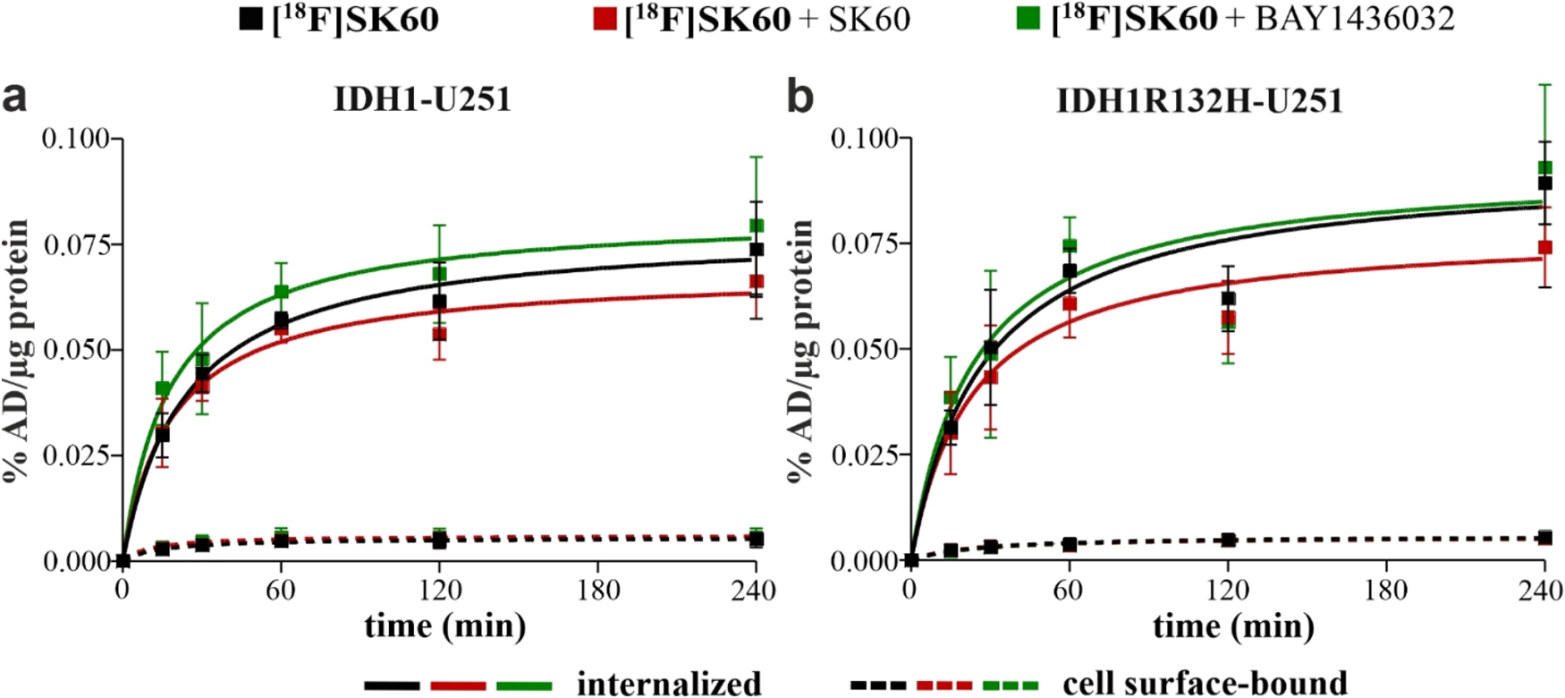
Cellular uptake of **[** ^**18**^ **F]­SK60** in IDH1-U251 (a) and IDH1R132H–U251 (b) cells. Surface-bound and internalized radioligand fraction kinetics in IDH1-U251 or IDH1R132-U251 preincubated (2 h) with vehicle (0.01% DMSO; control) or **BAY1436032** (1 μM) or **SK60** (1 μM) before the addition of **[** ^**18**^ **F]­SK60** (4.86 ± 0.33 nM). Results are presented as % of the applied dose of the radioligand per μg protein (% AD/μg protein) vs incubation time. Protein concentration per well was 150 ± 105 μg for IDH1-U251 and 177 ± 94 μg for IDH1R132H–U251 cells. All curves fit best with a one-site model. Data were obtained from 4 independent experiments.

Proportions of surface-bound (0.052 ± 0.029% AD/μg protein at 240 min incubation) and internalized (0.074 ± 0.023% AD/μg protein activity) compound in IDH1-U251 cells appear to be similar to that of IDH1R132H–U251 (0.005 ± 0.001 and 0.089 ± 0.02% AD/μg protein, respectively). Furthermore, preadministration of the pan-mIDH1 inhibitor **BAY1436032** or **SK60** in excess did not reduce the surface-bound nor the internalized radioligand in either of the cell lines. Despite the potency of **SK60** to specifically inhibit the IDH1R132H enzyme, the results of the uptake study did not indicate specific binding. This is supported by additional preliminary *in vitro* studies investigating real-time radioligand binding with both cell lines and radioligand binding with the cytosolic fraction of both cell line lysates (Figures S3 and S4, SI). Overall, all *in vitro* studies indicated substantial nonspecific binding of **[** ^**18**^ **F]­SK60** to plastic surfaces.

### *In Vivo* Metabolism in Naïve Mice

The metabolic stability of **[** ^**18**^ **F]­SK60** was investigated *ex vivo* by radio-RP-HPLC and radio-micellar-HPLC (MLC) analyses of plasma and brain samples obtained from naïve female mice (*n* = 3) at 30 min post intravenous injection (*p*.*i*.). For the preparation of the RP-HPLC samples, two solvent systems MeCN/water (9/1; v/v) and MeOH/water (9/1; v/v) were tested for activity extraction from the plasma and brain samples. With both systems, recoveries of higher than 95% could be obtained. The RP-HPLC chromatograms revealed that the parent radioligand fraction was 80 ± 2% and 100% in the plasma and brain, respectively (Figure 7A). For the radio-MLC analyses, the brain and plasma samples were treated with sodium dodecyl sulfate and directly injected into the MLC system. Despite the slightly different elution profile compared to the RP-HPLC system caused by different separation mechanisms, the quantification of the peaks gave similar results (Figure 7B). Altogether, the results obtained with these two complementary systems indicate a high metabolic stability of **[** ^**18**^ **F]­SK60** in mice without the formation of brain-penetrable radiometabolites.

**Figure 7:**
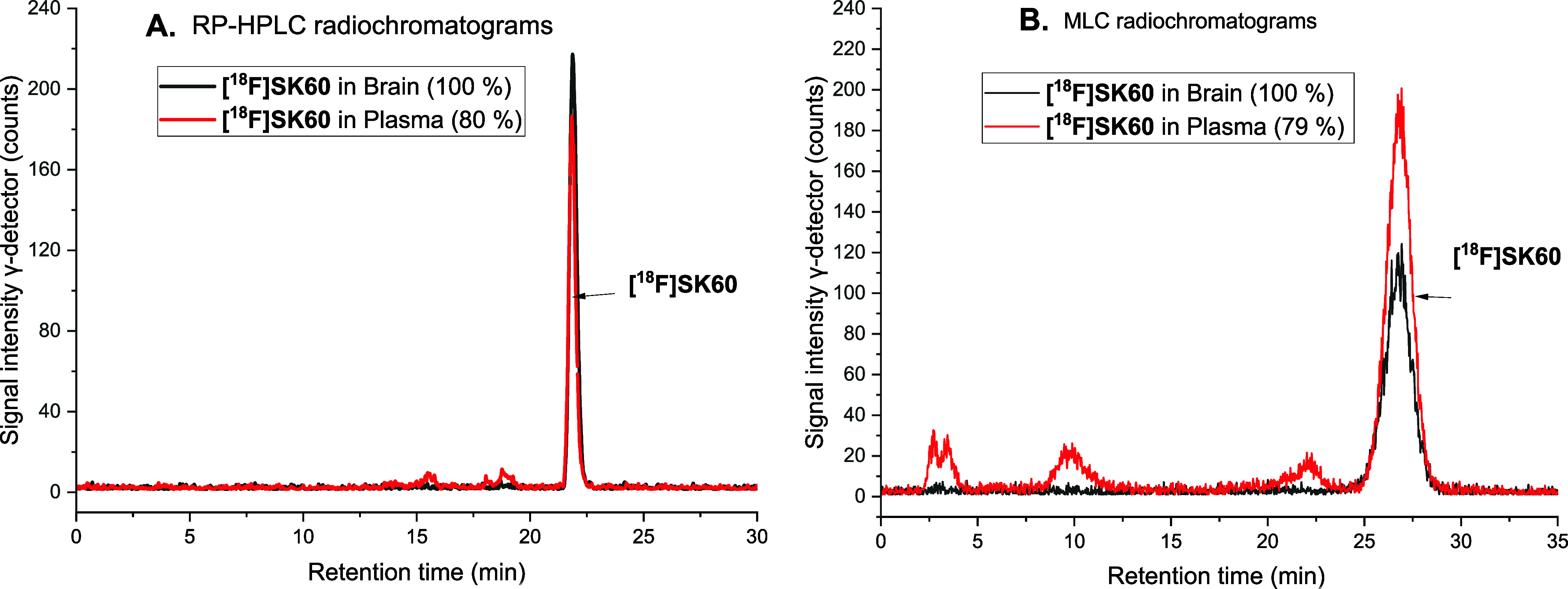
(A) RP-HPLC radiochromatograms of extracts from brain and plasma samples of naïve mice, 30 min p.i., of **[** ^**18**^ **F]­SK60**. HPLC conditions: Reprosil-Pur C18-AQ (250 × 4.6 mm; 5 μm); Gradient mode (10–90% MeCN, 20 mM NH_4_OAc_aq_); flow 1 mL/min; (B) MLC radiochromatograms of brain and plasma samples of naïve CD-1 mice at 30 min p.i. of **[** ^**18**^ **F]­SK60**. HPLC conditions: Reprosil-Pur 120 C18-AQ column (250 × 4.6 mm, 10 μm) and an eluent mixture of n-PrOH/100 mM SDS_aq_/25 mM (NH_4_)_2_HPO_4_ in gradient mode, flow 1 mL/min.

### Biodistribution in Naïve Mice

The *ex vivo* biodistribution study was performed on naïve CD-1 mice at 5, 10, 15, 30, and 60 min post intravenous (*i*.*v*.) injection (*n* = 2 or 3 at each time point) of **[** ^**18**^ **F]­SK60**. The results indicated negligible radiodefluorination *in vivo*, as demonstrated by the low bone uptake (Figure 8). Second, it revealed the highest initial accumulation occurring in the liver (∼7 SUV_mean_ at 5 min) and, over time, in the small intestine (∼13 SUV_mean_ at 60 min), indicative of a predominantly hepatobiliary excretion route (Figure S5, SI). Interestingly, a low amount of activity was found in the kidneys and bladder (<1 SUV_mean_ at 60 min). In addition, a pilot PET-based biodistribution study (*n* = 2) was performed and demonstrated that the high accumulation of activity found in the liver in the *ex vivo* study was presumably due to an accumulation in the gallbladder (liver: 1.6 SUV_mean_ at 30 min); (gallbladder: 60 SUV_mean_ at 30 min) (Figure 8B). Finally, limited brain uptake (∼0.3 SUV_mean_ at 15 min *p*.*i*.) was observed (Figure 8).

**Figure 8:**
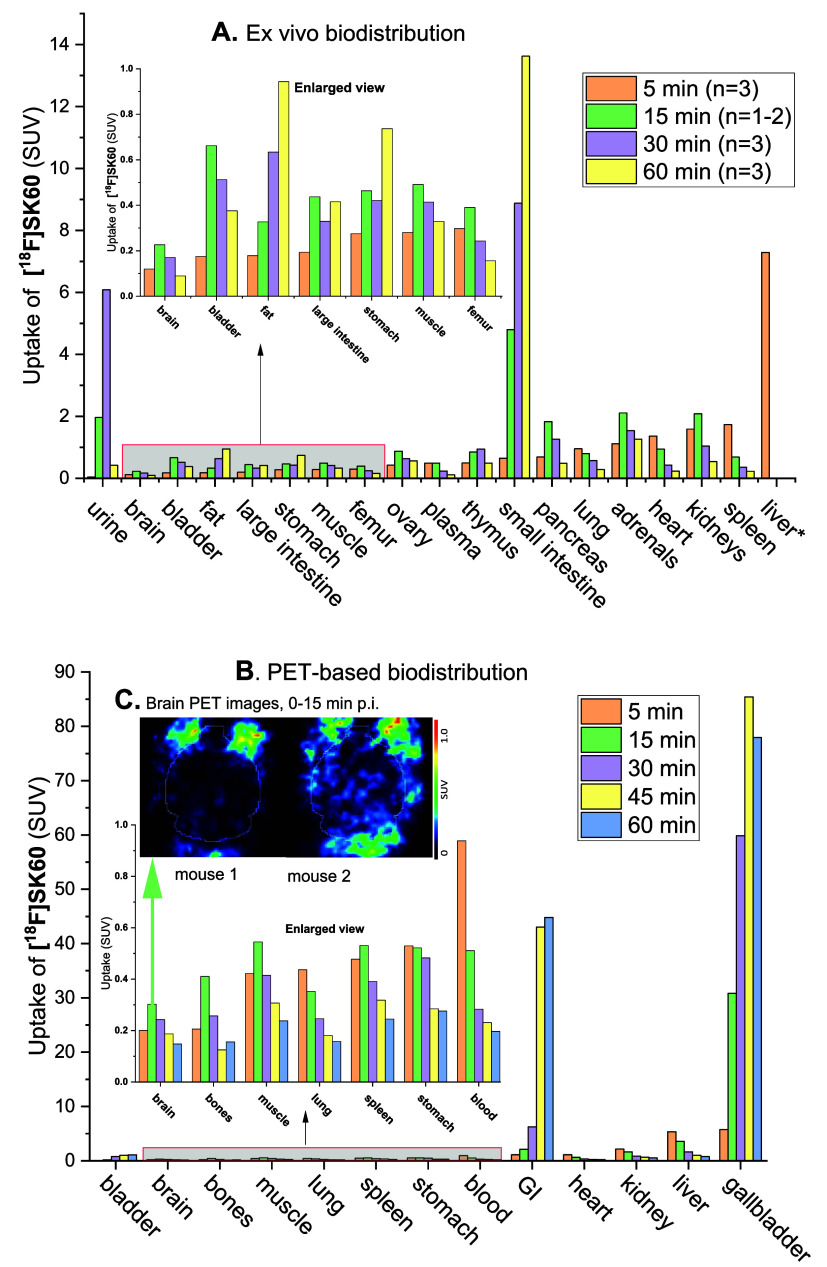
Biodistribution of radioactivity in naïve mice after **[** ^**18**^ **F]­SK60** injection. (A) *Ex vivo* biodistribution at 5, 15, 30, and 60 min (for liver* only at 5 min measured). (B) PET-based biodistribution from 0 to 60 min. (C) Representative horizontal averaged PET brain images overlapping with Ma-Benveniste Atlas (white outlines) (0–15 min p.i.).

The Permeability-glycoprotein (P-gp) efflux transporter expressed on the endothelial cells of the BBB was considered as a possible reason for the limited brain penetration of **[** ^**18**^ **F]­SK60**, together with the indicated biliary excretion of the radioligand (Figure S5), most likely related to P-gp.^59^ To investigate the role of P-gp, mice were treated with cyclosporine A, an inhibitor of the P-gp efflux transporter. This increased brain uptake of **[** ^**18**^ **F]­SK60** by approximately 2.5-fold, compared to vehicle (Figure 9). Overall, while the biological evaluation of **[** ^**18**^ **F]­SK60** underlines its unsuitability as a tracer for brain imaging, our study provides valuable insights into metabolism, pharmacokinetics, and nonspecific binding to support further drug development efforts based on this class of compounds for the brain as well as peripheral tumors.

**Figure 9:**
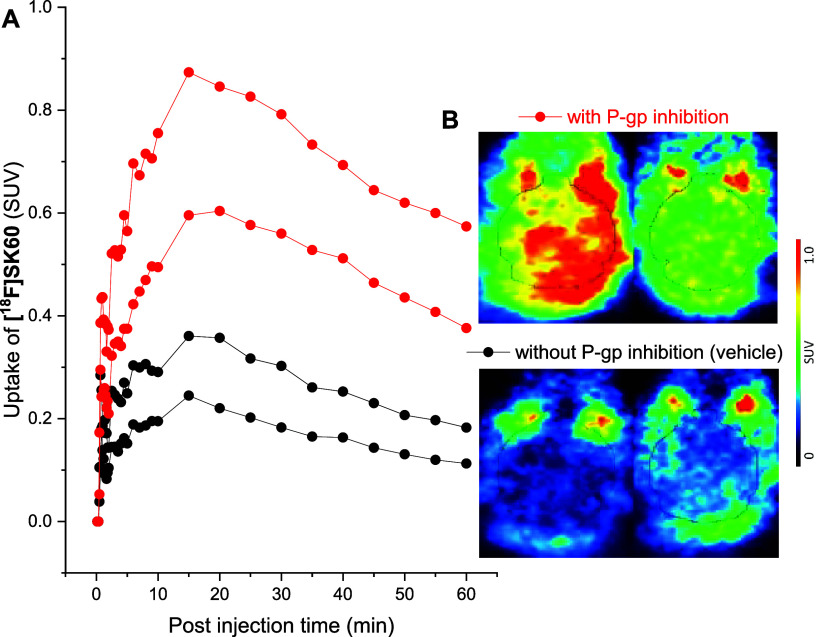
Efflux transporter P-gp substrate study in naïve CD-1 mice. (A) Brain time–activity curves (SUV_mean_) after pretreatment with either vehicle (black circles; *n* = 2) or cyclosporine A (Sandimmune, 50 mg/kg; red circles; *n* = 2) and (B) representative horizontal averaged PET brain images (0–60 min p.i.).

## Conclusions

To develop an IDH1R132H-PET radiotracer for noninvasive molecular imaging, **GSK321** was chosen as the lead compound due to its nanomolar potency and inherent fluorine atom for 18F-radiolabeling with fluorine-18. SAR optimization was performed to improve its selectivity, as **GSK321** showed only 10-fold selectivity for IDH1R132H over that of wtIDH1. Synthesis and evaluation of (*R*,*S*)-**9** (**GSK321**) and its stereoisomers revealed that (*R*,*S*)-**9** and (*R*,*R*)-**9** exhibited significantly higher potency against IDH1R132H compared to those of (*S*,*S*)-**9** and (*S*,*R*)-**9**. Furthermore, this highlights the importance of the (*R*)-configured CH_3_-group at position 1 for the inhibitory potency. To circumvent the challenging separation of stereoisomers, we developed a novel series of dimethylated derivatives lacking the chiral center at position 1, which exhibited potent inhibitory activity against IDH1R132H. Among these compounds, **SK60** was selected based on its excellent potency and selectivity for IDH1R132H, successfully radiolabeled via copper-mediated radiofluorination. The evaluation of **[** ^**18**^ **F]­SK60** uptake in IDH1-U251 and IDH1R132H–U251 cells indicated a high level of nonspecific binding to plastic surfaces. The *in vivo* studies in naïve mice confirmed the high metabolic stability of **[** ^**18**^ **F]­SK60**, without detectable brain-permeable radiometabolites. PET experiments revealed a low brain uptake that moderately increased upon P-gp blocking. These findings indicate that further structural optimizations are necessary to reduce nonspecific binding and improve brain uptake. Nevertheless, the dimethylated compounds developed in this study with low nanomolar inhibitory activity for IDH1R132H and high selectivity over wtIDH1 offer valuable potential for further developments in advancing therapeutic strategies for IDH1R132H-related diseases.

## Experimental Section

### Chemistry

The materials, devices, and the synthesis of all the compounds with their NMR characterization are reported in the Supporting Information (S1, SI). The chemical purity of the final compounds (≥95%) was confirmed by LC-MS and HPLC. LC-MS was performed using a Reprosil-Pur Basic HD column (150 × 3 mm, 3 μm, Dr. Maisch GmbH, Germany) with a linear gradient of MeCN and 20 mM NH_4_OAc_aq_ at a flow rate of 0.7 mL/min. HPLC analysis employed a Reprosil C18-AQ column (250 × 4.5 mm, 5 μm, Dr. Maisch GmbH, Germany) with a similar mobile phase used in gradient mode at a flow rate of 1 mL/min. The HPLC and LC-MS chromatograms are provided as a piece of Supporting Information in a separate PDF.

### X-ray Crystallography

The crystals of **SK60** (25 mg) were grown in MeCN over 4–5 weeks at room temperature, followed by X-ray crystallography studies. The conditions and parameters used for this study are mentioned in the Supporting Information (Table S1, SI).

### Circular Dichroism and Chiral HPLC

The CD spectra were recorded with the CD-4095 detector during the HPLC run in stopped-flow mode, during which the flow through the detector cell was bypassed by using a software-controlled switching valve. Each spectrum was obtained with a scanning speed of 10 nm/sec. The single enantiomers were dissolved in MeCN/water, 2/1 (v/v) for RP-HPLC or *i*-PrOH for NP-HPLC and subjected to chiral separations, which were performed on a CHIRALPAKIA/IB column (250 × 4.6 mm, 5 μm, Daicel—Chiral Technologies Europe, France) in isocratic mode with RP/NP conditions and a flow of 1 mL/min.

### Radiochemistry

No-carrier-added [^18^F]­fluoride was produced via the ^18^O­(*p*,*n*)^18^F nuclear reaction by irradiation of an­[^18^O]­H_2_O target (Hyox 18 enriched water, Rotem Industries Ltd., Israel) on a Cyclone 18/9 (iba RadioPharma Solutions, Belgium) with fixed energy proton beam using Nirta [^18^F]­fluoride XL target.

The anhydrous labeling solvents 1,3-dimethyl-2-imidazolidinone (DMI) and *N*,*N*-dimethylacetamide (DMA), as well as trifluoroacetic acid (TFA) and [Cu­(OTf)_2_(Py)_4_] (abbreviated as [Cu]), were purchased from Sigma-Aldrich Chemie GmbH, Part of Merck, Taufkirchen, Germany. The *n-*tetrabutylammonium hydrogen carbonate (TBAHCO_3_) was used as a 0.075 M solution from ABX advanced biochemical compounds GmbH, Radeberg, Germany. The salt 4-(dimethylamino)­pyridinium trifluoromethanesulfonate (DMAPHOTf) was prepared in-house according to the literature.^60^

Radio thin layer chromatography (radio-TLC) was performed on silica gel (Polygram SIL G/UV254 from Machery-Nagel, Germany) precoated plates with a mixture of ethyl acetate/*n*-hexane 3/1 (v/v) as eluent. The plates were exposed to storage phosphor screens (BAS-IP MS 2025, FUJIFILM Co., Tokyo, Japan) and scanned using the Amersham Typhoon RGB Biomolecular Imager (GE Healthcare Life Sciences). Images were quantified with the ImageQuant TL8.1 software (GE Healthcare Life Sciences).

Radio analytical chromatographic separations were performed on a JASCO LC-2000 system, incorporating a PU-2080Plus pump, AS-2055Plus autoinjector (100 μL sample loop), and a UV-2070Plus detector (Jasco Deutschland GmbH, Pfungstadt, Germany) coupled with a γ radioactivity HPLC detector (Gabi Star, Elysia-raytest GmbH, Straubenhardt, Germany). Data analysis was performed with Galaxie chromatography software (Agilent Technologies).

For radio analytical HPLC, a Reprosil-Pur 120 C18-AQ column (250 × 4.6 mm; 5 μm; Dr. Maisch HPLC GmbH; Germany) with MeCN/20 mM NH_4_OAc_aq_ (pH 6.8) as eluent mixture and a flow of 1.0 mL/min was used (gradient: eluent A 10% MeCN/20 mM NH_4_OAc_aq_.; eluent B 90% MeCN/20 mM NH_4_OAc_aq_.; 0–5 min 100% A, 5–17 min up to 100% B, 17–26 min 100% B, 26–27 min up to 100% A, 27–30 min 100% A). The ammonium acetate concentration is given as 20 mM NH_4_OAc_aq_ and corresponds to the concentration in the aqueous component of an eluent mixture.

Preconditioning of Chromafix 30 PS-HCO3-(45 mg) cartridge (ABX advanced biochemical compounds GmbH, Radeberg, Germany) and Sep-Pak Accell QMA light cartridge (Waters GmbH, Eschborn, Germany) was done using 10 mL of 0.5 M NaHCO3 followed by 10 mL of water. Preconditioning of Sep-Pak C18 light and plus cartridges (Waters GmbH, Eschborn, Germany) was done using 5 mL of EtOH and 20 mL of water.

For the production of [^18^F]­TBAF, n.c.a [^18^F]­fluoride was trapped on the preconditioned cartridge Sep-Pak Accell QMA light cartridge (Waters GmbH, Eschborn, Germany). The loaded [^18^F]­fluoride was eluted with a mixture of 800 μL of MeCN, 100 μL of a 0.075 M solution of TBAHCO_3_ (ABX advanced biochemical compounds GmbH, Radeberg, Germany), 100 μL of water, and 15 μL of an aqueous K_2_CO_3_ solution (20 mg/mL). The eluted aqueous [^18^F]­fluoride was azeotropically dried under vacuum and nitrogen flow within 7–10 min using a single mode microwave (75 W, at 50–60 °C, power cycling mode; Discover PETWave from CEM corporation). Two aliquots of MeCN (2 × 1.0 mL) were added during the drying procedure, and the final complex was obtained as a white solid.

For nonazeotropic drying: no carrier added [^18^F]­fluoride in 1.0 mL of water was trapped on a preconditioned Chromafix 30 PS-HCO_3_-(45 mg) cartridge (ABX advanced biochemical compounds GmbH, Radeberg, Germany). After loading, the cartridge was washed with 2 mL of anhydrous methanol and dried with a stream of nitrogen for 3 min. The activity was then eluted either with 5 mg (13 μmol) TBAOTf or 5 mg (37 μmol) DMAPHOTf dissolved in 500–600 μL of anhydrous methanol, achieving elution efficiency of trapped activity in the range of 80–90%. The methanol was evaporated under a stream of nitrogen at 60 °C.^61^

#### Manual Synthesis

The general procedure for optimization of manual synthesis of **[** ^**18**^ **F]­SK60** began with the azeotropically/nonazeotropically dried [^18^F]­fluoride, which was added to the solvent (300 μL of DMI or DMA), followed by the addition of [Cu] in (300 μL of DMI or DMA), prestirred at r.t. for 2 min (reactions with prestirring). Then, the precursor **64** (dissolved in 300 μL of DMI or DMA or *tert*-BuOH or *n*-BuOH) was added, and the resulting reaction mixture was stirred (at temperatures of 90, 110, or 130 °C) for 15 min. For monitoring of the labeling progress, aliquots were taken at 5, 10, and 15 min and analyzed by radio-TLC and randomly by radio-HPLC.

#### Automated Synthesis

The [^18^F]­fluoride was trapped on a preconditioned Sep-Pak Accell QMA light cartridge (entry 1) (Figure S13). The trapped [^18^F]­fluoride was eluted with the TBAHCO_3_ in the MeCN and water mixture (mentioned above) (entry 2) into the reaction vessel (entry 3) and was dried by azeotropic distillation (entry 4) at 90 °C for 15 min for [^18^F]­TBAF (Figure S13). Then, 15 μmol (10 mg) of [Cu] in 600 μL of DMI was added and stirred for 2 min (entry 5), followed by the addition of 3.5 μmol (2 mg) of precursor **64** in 300 μL of *n*-BuOH (entry 6) to the reactor (entry 3) and stirring for 10 min at 110 °C. The reaction mixture was then cooled to 35 °C, and 0.4% TFA in water (1 mL) was added manually using an externally connected syringe (entry 7) through the inlet 4 and stirred at 110 °C for 5 min. (Figure S13). Afterward, the reaction mixture was diluted with 18 mL of water (external addition via entry 7) (Figure S13). The diluted reaction mixture was loaded on a Sep-Pak C18 Plus cartridge (entry 8) and then eluted with 2.5 mL of 1/1 MeCN/THF (v/v) (entry 10) in the second reactor (entry 9). The resulting mixture containing **[** ^**18**^ **F]­SK60** was further diluted with 2.5 mL of water through entry 10, loaded into the RP-HPLC column (Reprosil-Pur C18-AQ, 250 × 20 mm), and eluted with a mixture of 48% MeCN/THF 1/1 (v/v) and 20 mM NH_4_OAc_aq_ with a flow rate of 7.8 mL/min (entry 11). The radiolabeled product **[** ^**18**^ **F]­SK60** was isolated at a retention time of about 73 min and directed to a collection vial (entry 12) that was previously loaded with 30 mL of water (Figure S13). The final purification step was achieved through SPE on a Sep-Pak C18 light cartridge (trap 4, entry 13), followed by washing of the cartridge with 2 mL of water (entry 14) and successive elution of the radioligand with the 1.2 mL of EtOH (entry 15) into the product vial (entry 16). The EtOH solution was transferred out of the hot cell, and volume was reduced under a gentle argon stream at 70 °C to a final volume of 10–25 μL. Afterward, the radiotracer was formulated in an isotonic saline solution to obtain a final product containing 10% EtOH (v/v).

#### Quality Control

For quality control, the final product of the radiotracer was inspected by analytical radio- and UV-HPLC with and without coinjection of the reference compound in isocratic mode with RP-HPLC using column Nucleodur PFP (250 × 4.6 mm; 5 μm) and a mobile phase consisting of 52% MeCN and 20 mM NH_4_OAc_aq_, with a flow rate of 1 mL/min at maximum absorbing wavelength 268 nm.

For the determination of molar activities of **[** ^**18**^ **F]­SK60**, an aliquot of the tracer solution (50 μL, 5–30 MBq) was analyzed by HPLC under isocratic conditions (mentioned above). The amount of nonradioactive **SK60** was calculated from calibration curves (Figure S11, SI) obtained under the same HPLC conditions.

### Determination of log*D*_7.4_

The apparent distribution coefficient (log*D*_7.4_) value of **[** ^**18**^ **F]­SK60** was determined using a shake-flask method by measuring the distribution of the radiotracer between *n*-octanol and phosphate-buffered saline (PBS, 0.05 mol/L, pH 7.4). The two phases were presaturated with each other. A solution of the radiotracer (10 μL, 1.1 MBq) was added to a 15 mL plastic centrifuge tube containing *n*-octanol (3 mL) and PBS (3 mL). The tube was vortexed for 3 min and centrifuged at 3500 rpm for 5 min (Anke TDL80–2B, China). About 50 μL of the *n*-octanol layer and 50 μL of the buffer layer were pipetted in two tared tubes, and the activity was measured in an automatic γ-counter (Wallac 2480 Wizard). The log*D*_7.4_ was determined as the ratio of cpm/mL of the *n*-octanol layer to that of the buffer layer. Samples from the *n*-octanol layer were redistributed until consistent distribution coefficient values were obtained. The measurement was carried out in triplicate and repeated three times.

### In Vitro Studies

#### Cell Culture

Human U251 glioblastoma cells, stably transfected and overexpressing either wild-type IDH1 (IDH1-U251) or IDH1R132H (IDH1R132H–U251), were obtained from Jaqueline Kessler and Dirk Vordermark (Department of Radiotherapy, Martin Luther University Halle-Wittenberg, Halle/Saale, Germany). Cells were grown in RPMI 1640 medium (10% FCS), supplemented with puromycin, to ensure the cultivation of transfected cells only.

##### IDH1 Enzyme Assay for Determination of Inhibitory Potency

IDH1 enzyme assay is a modified version of the assay used for mIDH1. With the conversion of isocitrate to α-KG, this enzyme stoichiometrically converts NADP to NADPH. It produces NADPH, which directly couples to the diaphorase/resazurin system, and the resorufin production can be measured. The protocols for the determination of inhibition of IDH1 were performed as described by Wang et al. (2013). The IDH1 (ab113858) was purchased from abcam (Cambridge, UK).

Briefly, IDH1 assays were conducted in 50 μL buffer (20 mM TRIS buffer (pH = 7.5), 150 mM NaCl, 10 mM MgCl_2_, 0.05% BSA and 4 mM β-mercaptoethanol) containing 50 μM NADP, 70 μM *DL*-isocitrate, and 0.04 μg/mL IDH1 enzyme (reaction time 1 h at room temperature). For inhibition assays, triplicate samples of the ligands in the concentration range from 10^–4^ M to 10^–10^ M were incubated with the IDH1 for 1 h before the addition of *DL*-isocitrate and NADP to initiate the reaction together with the direct detection system comprised of 20 μg/mL diaphorase and 4 μM resazurin. The reaction was terminated with the addition of 25 μL of 2% SDS and read on a Synergy H1 microplate reader at Ex544/Em590. The data were imported into GraphPad Prism, and the IC_50_ values were calculated with a standard dose–response curve fitting (Figure S2, SI).

##### Mutant IDH1 Enzyme Assay for the Determination of Inhibitory Potency

Determination of the activity and inhibition of mutant IDH1 recombinant protein, IDH1R132H, is based on the reduction of α-KG acid to *D*-2-HG accompanied by a concomitant oxidation of NADPH to NADP. The amount of NADPH remaining at the end of the reaction time is measured in a secondary diaphorase/resazurin reaction, in which the NADPH is consumed in a 1/1 molar ratio with the conversion of resazurin to the highly fluorescent resorufin. For the determination of the inhibitory potential of ligands, the IDH1R132H Assay Kit (BPS-79376, BPS Bioscience, San Diego, CA) was used. The enzyme activity assay was performed in a volume of 100 μL Buffer (20 mM TRIS buffer (pH = 7.5), 150 mM NaCl, 10 mM MgCl_2_, 0.05% bovine serum albumin (BSA), and 4 mM β-mercaptoethanol) containing 0.5 ng/μL IDH1R132H enzyme, 2 mM α-KG, and 12 μM NADPH. For inhibition assays, triplicate samples of the ligands in the concentration range from 10^–5^ M to 10^–10^ M were incubated with the enzyme for 30 min before the addition of α-KG and NADPH to initiate the reaction. The reaction ran for 60 min at room temperature and was terminated with the addition of 25 μL of detection buffer (36 μg/mL diaphorase and 30 mM resazurin) to 50 μL of the reaction solution. The conversion of resazurin to resorufin by diaphorase was measured fluorometrically at Ex544/Em590 (Synergy H1 microplate reader, BioTek, Winooski, VT). The data were imported into GraphPad Prism 4.1 (GraphPad Inc.; La Jolla; CA), and the IC_50_ values were calculated with a standard dose–response curve fitting (Figure S2, SI).

#### *In Vitro* Cell Uptake Studies Using **[** ^**18**^ **F]­SK60** and Stably Transfected U251 Cells

The IDH1-U251 and IDH1R132H–U251 cells were seeded at 4,00,000 cells/mL in a 24-well cell culture plate 1 day before the experiment. The medium was replaced by 400 μL/well, and 4 μL of a 100-x stock solution of the respective inhibitor in 10% DMSO or vehicle (10% DMSO) was added about 2 h before the experiment. The experiment was started with the addition of 100 μL of **[** ^**18**^ **F]­SK60** (0.477 ± 0.002 MBq/mL; 4.86 ± 0.33 nM) diluted in cell culture medium per well, and the well plates were incubated in a humidified-air atmosphere incubator containing 5% CO_2_ at 37 °C for various times. The incubation was stopped by aspiration of the supernatant and washing the cell layers twice with prechilled PBS (500 μL/well). Cell surface-bound activity was released by the addition of acid-glycine buffer (0.2 M glycine, 0.15 M NaCl, pH 3; 500 μL/well) and incubation at room temperature for 10 min. The supernatant was collected and pooled with the supernatant obtained by subsequent washing with PBS (500 μL/well). Finally, the cells were lysed (0.1 M NaOH + 1% SDS; 500 μL/well; 37 °C, 30 min). Activities in the acidic wash and lysis samples, along with aliquots of the radioligand solution, were measured in a γ-counter (Wallac 2480 Wizard, PerkinElmer, Waltham, MA). Cells cultured in an additional well plate and treated as above, except for the addition of radioligand, were used as a control and to determine the protein concentration per well by a BCA assay (Pierce, #23227). The concentrations of surface-bound and internalized activities per well were calculated as a percentage of the applied dose per well and normalized to the protein concentration per well (% AD/μg protein). All experiments were performed in triplicates.

#### Real-Time Binding Experiments

Experimental details of real-time binding experiments are mentioned in the Supporting Information (S3, SI).

### *In Vivo* Studies

#### Animal Studies

All animal experiments have been conducted according to the national legislation on the use of animals for research and were approved by the responsible authorities of Saxony (Landesdirektion Sachsen, Referat 25 - Veterinärwesen and Lebensmittelüberwachung; TVV 18/18, DD24.1–5131/446/19, valid until June 23rd, 2023). CD-1 mice (female, 8–10 weeks, 30–40 g) were obtained from the Medizinisch-Experimentelles Zentrum (MEZ) at the Medical Faculty of Leipzig University (Leipzig, Germany).

#### Biodistribution

**[** ^**18**^ **F]­SK60** (151 ± 37.0 kBq) diluted in 200 μL of sterile isotonic saline was injected into the tail vein of restrained, awake mice. The animals were anesthetized with isoflurane at 5 (*n* = 2), 15 (*n* = 1–2), 30 (*n* = 3), and 60 (*n* = 3) min after injection; blood was collected from the retro-orbital plexus with a heparinized glass Pasteur pipet, and the anesthetized animal was sacrificed by cervical dislocation. Blood plasma was obtained by centrifugation of the whole blood (8000 rpm, 4 °C, 2 min). Organs and tissues of interest were isolated and weighed, and the activity was measured in an automated γ-counter (2480 Wizard; PerkinElmer) together with aliquots of the radioligand solution, the empty syringes, and the remaining tissue. From the measured data, the uptake of the activity in the different tissues and organs at the different time points *p*.*i*. was calculated as a percentage of injected dose per gram of tissue (% ID/g) and standardized uptake values (SUV).

#### Metabolism

**[** ^**18**^ **F]­SK60** (24.3 ± 2.89 MBq) diluted in 200 μL of sterile isotonic saline was injected into the tail vein of restrained, awake mice. Thirty min after injection, animals were anesthetized with isoflurane, blood was collected from the retro-orbital plexus with a heparinized glass Pasteur pipet, and the anesthetized animal was sacrificed by cervical dislocation. Blood plasma was obtained by centrifugation of the whole blood (8000 rpm, 4 °C, 2 min). The brain was isolated, rapidly rinsed with 1 mL isotonic saline to remove superficial blood, and finally homogenized in 1 mL deionized water in an ice–water bath (Potter B. Braun, 1000 rpm, 10 strokes).

##### RP-HPLC

Protein precipitation and extraction were performed by using an ice-cold MeCN/water (9:1 v/v) or MeOH/water (9:1 v/v) mixture at a 4:1 (v/v) solvent-to-sample ratio for plasma or brain homogenates. Samples were vortexed for 3 min, equilibrated on ice for 5 min, and centrifuged at 10,000 rpm for 5 min. The supernatant was separated, and precipitates were washed with 100 μL of the solvent mixture, repeating the process. Combined supernatants were concentrated under nitrogen at 75 °C to ∼100 μL and analyzed via radio-HPLC using a Reprosil-Pur 120 C18-AQ column with a gradient MeCN/20 mM NH_4_OAc_aq_ (pH 6.8) elution (gradient: eluent A 10% MeCN/20 mM NH_4_OAc_aq_.; eluent B 90% MeCN/20 mM NH_4_OAc_aq_.; 0–5 min 100% A, 5–17 min up to 100% B, 17–26 min 100% B, 26–27 min up to 100% A, 27–30 min 100% A). Recovery rates of ≥ 95% for plasma and brain homogenates were confirmed by γ counter analysis.

##### MLC

For the preparation of the MLC samples, mouse plasma (20–50 μL, 2–3 kBq) was dissolved in 200 μL of 200 mM aqueous sodium dodecyl sulfate (SDS) and injected into the MLC system. Homogenized brain material (∼300 μL) was dissolved in 700 μL of 200 mM aqueous SDS, stirred at 75 °C for 5 min, and after cooling to ambient temperature, centrifuged for 5 min at 10.000 rpm. The supernatant was then injected into the MLC system. Separations were performed by using a Reprosil-Pur 120 C18-AQ column (250 × 4.6 +10 mm precolumn, particle size: 10 μm) and an eluent mixture of 1-propanol/100 mM SDS_aq._/25 mM (NH_4_)_2_HPO_4_ in gradient mode with eluent A: 100% 100 mM SDS_aq_/25 mM (NH_4_)_2_HPO_4,aq_ and eluent B: 30% 1-propanol/100 mM SDS_aq_/ 25 mM (NH_4_)_2_HPO_4,aq_ 0–10 min 100% A, 10–15 min up to 100% B, 15–25 min 100% B, 25–35 min up to 100% A, 31–40 min 100% A, flow 1.0 mL/min. The MLC system consisted of a JASCO PU-980 pump, an AS-2055Plus autoinjector with a 2 mL sample loop, and a UV-1575 detector coupled with a γ radioactivity HPLC detector (Gabi Star, raytest Isotopenmessgeräte GmbH; Straubenhardt; Germany). Data analysis was performed with the Galaxie chromatography software (Agilent Technologies).

#### PET Experiments in Naïve CD-1 Mice

For the time of the experiments, female CD-1 mice (*n* = 4; 8–10 weeks; 37–40 g) were kept in a dedicated climatic chamber with free access to water and food under a 12:12 h dark: light cycle at a constant temperature (24 °C). The animals were anesthetized (Anaesthesia Unit U-410, agntho’s, Lidingö, Sweden) with isoflurane (2.0%, 300 mL/min) delivered in a 20% oxygen/80% air mixture (Gas Blender 100 Series, MCQ instruments, Rome, Italy) and their body temperature maintained at 37 °C with a thermal bed system. For P-gp efflux transporter studies, a pretreatment via *i*.*v*. injection of cyclosporine A (*n* = 2; Sandimmune, 50 mg/kg) or of vehicle (*n* = 2; NaCl/DMSO/kolliphor, 7:1:2, v/v) was performed 30 min before **[** ^**18**^ **F]­SK60** (4.6 ± 0.5 MBq; 6.6 ± 2.4 nmol/kg; *A*_m_: 34 GBq/μmol, EOS) *i*.*v*. injection. A dynamic 60 min PET scan (Nanoscan PET/MRT 1 T, Mediso, Hungary) was started 20 s before the radioligand injection. Each PET image was corrected for random coincidences, dead time, scatter, and attenuation (AC) based on a whole body (WB) MR scan. The reconstruction parameters for the list mode data were 3D-ordered subset expectation maximization (OSEM), 4 iterations, 6 subsets, energy window: 400–600 keV, coincidence mode: 1–5, ring difference: 81. The PET data were collected by a continuous WB scan during the entire investigation. Following the 60 min PET scan, a T1 weighted WB gradient echo sequence (TR/TE: 20/6.4 ms, NEX: 1, FA: 25, FOV: 64 × 64 mm, Matrix: 128 × 128, STh: 0.5 mm) was performed for AC and anatomical orientation. Image registration and evaluation of the region of interest (ROI) was done with PMOD (PMOD Technologies LLC, v. 3.9, Zurich, Switzerland). The brain region was identified using the mouse brain atlas template Ma-Benveniste-Mirrione-FDG. The activity data are expressed as the SUV_mean_ of the overall ROI.

## Supplementary Material





## Notes

Chemical synthesis of all compounds; X-ray crystallography of compound **SK60**; inhibitory potency determination for target compounds; real-time radioligand binding assay; radioligand binding to lysates of IDH1-U251 and IDH1R132-U251 cells; PET biodistribution of **[** ^**18**^ **F]­SK60**; synthesis of **8**, (*S*)-**8**, and (*R*)-**8** and separation of stereoisomers of **9**; Formation of protodeboronated side product (**51**) during the radiosynthesis **[** ^**18**^ **F]­SK60**; calibration curve of **SK60**; schematic interface for the automated radiosynthesis; and ^1^H NMR; HPLC and LC-MS chromatograms of final compounds (PDF)
Molecular Formula Strings (CSV)

## Abbreviations Used

Ac: acetyl
AcOH: acetic acid
Am: molar activity
AML: acute myeloid leukemia
Aq.: aqueous
BBB: blood–brain barrier
Bpin: boronic acid pinacol ester
[Cu]: Cu­(Py)_4_(OTf)_2_
c.a: carrier added
CD: circular dichroism
CMRF: copper-mediated radiofluorination
CNS: central nervous system
conc.: concentrated
*D*-2-HG: *D*-2-Hydroxyglutarate
DCM: dichloromethane
DMA: dimethylacetamide
DMAP: 4-dimethylaminopyridine
DMAPHOTf: 4-(dimethylamino)­pyridinium triflate
DMF: dimethylformamide
DMI: 1,3-dimethyl-2-imidazolidinone
DMSO: dimethyl sulfoxide
eq: equivalents
ESI: electron-spray ionization
EtOAc: ethyl acetate
EtOH: ethanol
FDA: Food and Drug Administration
GBM: glioblastoma
Hex: hexane
HGG: high-grade gliomas
HOTf: triflic acid
HPLC: high-performance liquid chromatography
HRMS: high-resolution mass spectrometry
*i*-Pr_2_NEt: *N*,*N*-diisopropylethylamine
*i*-PrOH: isopropanol
IDH: isocitrate dehydrogenase
i.v.: intravenous
K222: Kryptofix222
LCMS: liquid chromatography–mass spectrometry
LDA: lithium diisopropylamide
LGG: low-grade glioma
MeCN: acetonitrile
MeOH: methanol
mIDH: mutant isocitrate dehydrogenase
MLC: micellar liquid chromatography
MRI: magnetic resonance imaging
MRS: magnetic resonance spectroscopy
MS: mass spectrometry
*n*-BuOH: *n*-Butanol
n.c.a.: noncarrier added
nd: not determined
NADP+: nicotinamide adenine dinucleotide phosphate (oxidized form)
NADPH: nicotinamide adenine dinucleotide phosphate (reduced form)
NEt_3_: triethylamine
NH_4_OAc_aq_: ammonium acetate (aqueous)
NMR: nuclear magnetic resonance spectroscopy
NP: normal phase
p.i.: post injection
PBS: phosphate-buffered saline
PET: positron emission tomography
P-gp: P-glycoprotein
PTC: phase transfer catalyst
pyBOP: benzotriazol-1-yloxytripyrrolidinophosphonium hexafluorophosphate
QMA: quaternary methylammonium cartridge
r.t.: room temperature
RCC: radiochemical conversion
RCP: radiochemical purity
RCY: radiochemical yield
RP: reversed-phase
SPE: solid phase extraction
TBAHCO_3_: tetrabutylammonium hydrogen carbonate
TBAOTf: tetrabutylammonium triflate
*t*-BuOH: tert-butanol
TEAHCO_3_: tetraethylammonium hydrogen carbonate
TET2: tet methylcytosine dioxygenase 2
TFA: trifluoroacetic acid
THF: tetrahydrofuran
THPP: tetrahydropyrazolo pyridine
TLC: thin layer chromatography
WHO: World Health Organization
wtIDH: wild-type isocitrate dehydrogenase
α-KG: α-Ketoglutarate
