# Supplementary material for: From Inhibitors to PET: SAR-Based Development of [18F]SK60 for mIDH1 Imaging
Source: J Med Chem. 2025 Jun 29;68(13):13750–71. doi: 10.1021/acs.jmedchem.5c00584 (PMC12257540; doi:10.1021/acs.jmedchem.5c00584)
Supplement: Supplementary file 1 [file jm5c00584_si_001.pdf]

Supporting Information

From inhibitors to PET: SAR-based development of [^{18}F]SK60 for mIDH1 imaging

Sarandeep Kaur*, Sladjana Dukic-Stefanovic, Winnie Deuther-Conrad, Magali Toussaint, Barbara Wenzel, Peter Lönnecke, Cornelius K. Donat, Rareş-Petru Moldovan*, and Klaus Kopka

Helmholtz-Zentrum Dresden-Rossendorf (HZDR), Institute of Radiopharmaceutical Cancer Research, Department of Experimental Neurooncological Radiopharmacy, Research Site Leipzig, 04318 Leipzig, Germany.
Helmholtz-Zentrum Dresden-Rossendorf (HZDR), Institute of Radiopharmaceutical Cancer Research, 01328 Dresden, Germany.
Technische Universität Dresden, School of Science, Faculty of Chemistry and Food Chemistry, 01069 Dresden, Germany.
German Cancer Consortium (DKTK), Partner Site Dresden, 01307 Dresden, Germany.
Univ. Hosp. Carl Gustav Carus, TU Dresden & Helmholtz-Zentrum Dresden-Rossendorf (HZDR), 01307 Dresden, Germany.
University of Leipzig, Faculty of Chemistry and Mineralogy, 04103 Leipzig, Germany.
**Correspondence: Sarandeep Kaur (s.kaur@hzdr.de, Tel.: +49-3412341794639) Rareş-Petru Moldovan (r.moldovan@hzdr.de, Tel.: +49-3412341794634)*
KEYWORDS. Gliomas, IDH1R132H mutation, mIDH1 inhibitor, Copper-mediated radiofluorination (CMRF), 2-hydroxyglutarate (2-HG), Positron Emission Tomography (PET)

Contents

Author names ... 1

S1: CHEMISTRY ... **3**

S2: X-RAY CRYSTALLOGRAPHY ... **18**

S3: THE *IN VITRO* AND *IN VIVO* STUDIES ... **20**

Inhibitory potency determination ... 20

Real-time radioligand binding ... 21

Radioligand binding to lysates of IDH1-U251 and IDH1R132-U251 cells ... 22

PET Biodistribution ... 24

S4: SEPARATION OF STEREOISOMERS OF 9 ... **25**

S5: FORMATION OF PROTODEBORONATED SIDE PRODUCT (51) DURING THE RADIOSYNTHESIS [^{18}F]SK60 ... **28**

S6: CALIBRATION CURVE OF SK60 ... **32**

S7: SCHEMATIC INTERFACE FOR THE AUTOMATED RADIOSYNTHESIS ... **32**

S7: QUALITY CONTROL OF [^{18}F]SK60 ... **33**

S8: NMR OF FINAL COMPOUNDS ... **34**

S9: HPLC/LCMS CHROMATOGRAMS OF FINAL COMPOUNDS ... **67**

REFERENCES ... **80**

S1: Chemistry

All chemicals and reagents were commercially purchased and used without further purification unless otherwise mentioned. Moisture-sensitive reactions were carried out under dry argon. The solvents used were purified and dried according to standard procedures. Distilled solvents of technical grade were used for purification procedures. Unless otherwise stated, the yields correspond to the purified compounds.

^{1}H, ^{13}C and ^{19}F NMR spectra were measured on VARIAN "MERCURY plus" (300 MHz for 1H-NMR, 75 MHz for ^{13}C-NMR, 282 MHz for ^{19}F-NMR) and BRUKER DRX-400 (400 MHz for ^{1}H-NMR, 100 MHz for ^{13}C-NMR, 377 MHz for ^{19}F-NMR). The chemical shifts (*δ*)of signals are reported in ppm units. All spectra were recorded at room temperature followed by calibration on the solvent signal [$CDCl_3$: *δ*(^{1}H-NMR) = 7.26 ppm and *δ*(^{13}C-NMR) = 77.16 ppm, DMSO-d_6: *δ*(^{1}H-NMR) = 2.5 ppm and *δ*(^{13}C-NMR) = 39.52 ppm] and on TMS as an external standard. Multiplicities of NMR (J) signals are indicated as follows: s (singlet), d (doublet), t(triplet), q (quartet), m (multiplet), dd (doublet of doublets), dt (doublet of triplets), td (triplet of doublets), ddd (doublet of doublets of doublets).

High-resolution mass spectra (HRMS) were recorded on an FT-ICR APEX II spectrometer (Bruker Daltonics; Bruker Corporation, Billerica, MA, USA) using electrospray ionization (ESI).

LC-MS was performed on a Dionex Ultimate 3000 system, incorporating an LPG-3400SD pump, an autosampler WPS-3000 TSL, a column compartment TCC-3000SD, a diode array detector DAD3000 (monitoring from 254 to 720 nm) and a low-resolution mass spectrometer MSQ 3000 (Thermo Fisher Scientific Inc., Waltham, USA).

Analytical chromatographic separations were performed on a JASCO LC-2011 system, incorporating a PU-2080Plus pump, AS-2055Plus auto-injector (100 µL sample loop), and a UV-2070Plus detector (Jasco Deutschland GmbH, Pfungstadt, Germany) coupled with an optical activity HPLC detector. Data analysis was performed with the Galaxie chromatography software (Agilent Technologies).

Analytical thin-layer chromatography was performed on silica gel-coated plates (Macherey-Nagel, ALUGRAM SIL G/UV254). The spots were identified by using a UV lamp or by a suitable solution of TLC staining reagents (0.1 % ninhydrin in ethanol/water 1/10, 10 % phosphomolybdic acid in ethanol, $KMNO_4$ solution in water, Hanessian's Stain).

The flash column chromatography was performed on silica gel ZEOsorb 60/40-63 µm from Apollo Scientific Ltd. and silica gel 40-63 µm from VWR Chemicals.

Chemical names, chemical formulas, the exact mass of compounds, total polar surface area (tPSA) and clogP were generated by ChemDraw Professional 17.0 unless otherwise mentioned.

Synthesis of compounds 2-66

2, tert-butyl 3-(2-ethoxy-2-oxoacetyl)-5-methyl-4-oxopiperidine-1-carboxylate, $C_{15}H_{23}NO_6$

The commercially available compound **1** (4 g, 18.7 mmol) was dissolved in THF (20 mL), followed by the addition of a solution of 2 M LDA in THF (10.75 mL, 21.5 mmol) at -78 °C. The reaction mixture was stirred at this temperature for 1 h before diethyl oxalate (3.08 mL, 22.7 mmol) was added with a syringe. The resulting mixture was allowed to warm to r.t. and stirred overnight. The reaction was quenched with ice cubes. The reaction mixture was then extracted with an aqueous solution of 25 % citric acid (~pH 5) and EtOAc (100 mL x 3), and the organic layer was collected, washed with brine (50 mL), dried over $MgSO_4$, and concentrated under reduced pressure to get **2** (4.124 g, 70.2 % yield). ^{1}H NMR (400 MHz, $CDCl_3$) δ ppm 15.45 (br.s, 1H), 4.45 (s, 2H), 4.34 (d, *J* = 5.8 Hz, 2H), 3.80 (s, 1H), 3.22 (s, 1H), 2.66 (s, 1H), 1.45 (s, 9H), 1.37 (t, *J* = 7.1 Hz, 3H), 1.21 (d, *J* = 7.0 Hz, 3H). HRMS (ESI+): *m/z* (%) = 314.1556, calculated 314.1525 for $C_{15}H_{24}NO_6$ $[M+H]^+$

3, 5-(tert-butyl) 3-ethyl 7-methyl-1,4,6,7-tetrahydro-5H-pyrazolo[4,3-c]pyridine-3,5-dicarboxylate, $C_{15}H_{23}N_3O_4$

The compound **2** (2 g, 6.38 mmol) was dissolved in acetic acid (5 mL), followed by the addition of hydrazine hydrate (1.12 mL, 15.31 mmol). After 2 h, the reaction mixture was concentrated under reduced pressure and the residue was poured into ice-cold saturated aqueous $NaHCO_3$ (100 mL) and extracted with three portions of EtOAc (100 mL x 3). The combined extracts were washed with brine (50 mL), dried over $MgSO_4$, filtered, concentrated under reduced pressure, and dried to constant weight to provide **3** (1.86 g, 5.99 mmol, 94 % yield) as a pale yellow solid. ^{1}H NMR (400 MHz, $CDCl_3$) δ ppm 9.82 (s, N*H*), 4.71 (s, 1H), 4.36 (d, *J* = 7.1 Hz, 1H), 3.77 – 3.71 (m, 2H), 3.02 (d, *J* = 5.6 Hz, 1H), 1.88 – 1.81 (m, 2H), 1.48 (s, 9H), 1.36 (t, *J* = 7.1 Hz, 3H), 1.26 (t, *J* = 6.9 Hz, 3H). HRMS (ESI+): *m/z* (%) 310.1713, calculated 310.1689 for $C_{15}H_{24}N_3O_4$ $[M+H]^+$.

4, 5-(tert-butyl) 3-ethyl 1-(4-fluorobenzyl)-7-methyl-1,4,6,7-tetrahydro-5H-pyrazolo[4,3-c]pyridine-3,5-dicarboxylate, $C_{22}H_{28}FN_3O_4$

The compound **3** (3 g, 9.69 mmol) was dissolved in dry THF (20 mL), followed by the addition of Cs2CO3 (9.6 g, 29.07 mmol) with stirring under nitrogen. After 1 h, 1-(bromomethyl)-4-fluorobenzene (1.25 mL, 9.69 mmol) was added and the reaction mixture was stirred at r.t. for 15 h under argon. The reaction mixture was then rotary evaporated to remove THF. The residue was extracted with EtOAc (100 mL x 3) and water (100 mL). The combined extracts were washed with brine (50 mL), dried over $MgSO_4$, filtered, and concentrated under reduced pressure. The residue was purified by chromatography on silica gel (Hex/EtOAc 5/1 → EtOAc). The appropriate fractions were combined, and concentrated under reduced pressure and the residue was dried to constant weight under high vacuum to provide **4** (3.07 g, 7.36 mmol, 76 % yield) as a colorless viscous oil. ^{1}H NMR (400 MHz, $CDCl_3$) δ ppm 7.10 (m, 2H), 7.05 – 6.94 (m, 2H), 5.43 – 5.22 (m, 2H), 5.20 – 4.88 (m, 1H), 4.41 (m, 2H), 4.30 – 3.90 (m, 2H), 3.07 (m, 1H), 2.78 (s, 1H), 1.47 (s, 9H), 1.40 (t, *J* = 7.2 Hz, 3H), 1.09 (d, *J* = 6.9 Hz, 3H). HRMS (ESI+): *m/z* (%) = 418.2152, calculated 418.2064 for $C_{22}H_{29}FN_3O_4$ $[M+H]^+$

5, 3-(ethoxycarbonyl)-1-(4-fluorobenzyl)-7-methyl-4,5,6,7-tetrahydro-1H-pyrazolo[4,3-c]pyridine hydrochloride, $C_{18}H_{23}FN_3O_2Cl$

To a solution of **4** (0.5 g, 1.2 mmol) in 1,4-dioxane (1.4 mL) was added 4 N HCl 1,4-dioxane. The reaction mixture was stirred at r.t. for 2 h. The solvent was removed under reduced pressure and dried to constant weight under a high vacuum to yield golden yellow foam **5** (0.42 g, 98.9 %). This material was used without further purification. ^{1}H NMR (400 MHz, $CDCl_3$) δ ppm 10.53 – 10.27 (m, 1H), 9.87 (s, 1H), 7.07 (dd, *J* = 8.8, 5.2 Hz, 2H), 6.99 (t, *J* = 8.6 Hz, 2H), 5.52 – 5.22 (m, 2H), 4.64 – 4.18 (m, 4H), 3.39 – 3.18 (m, 2H), 3.17 – 3.05 (m, 1H), 1.37 (t, *J* = 7.1 Hz, 6H). ^{19}F NMR (377 MHz, $CDCl_3$) δ ppm -113.36 (dq, *J* = 8.7, 4.3 Hz, 1F).

6, ethyl 1-(4-fluorobenzyl)-7-methyl-5-(1H-pyrrole-2-carbonyl)-4,5,6,7-tetrahydro-1H-pyrazolo[4,3-c]pyridine-3-carboxylate, $C_{22}H_{22}FN_4O_3$

To a solution of 1*H*-pyrrole-2-carboxylic acid (144.4 mg, 1.3 mmol, 1.2 eq) in DMF (10 mL), added pyBOP (687 g, 1.3 mmol, 1.2 eq), and *i*-Pr_2NEt (0.8 mL, 4.4 mmol, 4 eq) and stirred for 1 h under argon. Afterwards, **5** (400 mg, 1.1 mmol, 1 eq) in DMF (8 mL) was added and the reaction mixture was stirred at 25 °C for 18 h. The reaction mixture was rotary evaporated to remove DMF, and the resulting residue was washed with EtOAc (30 mL x 3) and water (10 mL). The combined organic layer was washed with brine (5 mL), dried over $MgSO_4$, filtered, and evaporated under vacuum to give a crude product which was purified on silica gel (PE: EtOAc 5:6 →EtOAc). The combined fractions provided **6** (415 mg, 92 %).

7, 1-(4-fluorobenzyl)-7-methyl-5-(1H-pyrrole-2-carbonyl)-4,5,6,7-tetrahydro-1H-pyrazolo[4,3-c]pyridine-3-carboxylic acid, $C_{20}H_{19}FN_4O_3$

To a solution of **6** (400 g, 0.97 mmol) in EtOH (5 mL) was added aqueous 1 N NaOH (3 mL). The clear solution was stirred at r.t. for 2 h. Afterwards, the reaction was judged complete by LCMS. The EtOH was removed under reduced pressure and the residue was diluted with water and pH adjusted to ~6 with 1 N HCl. The resulting precipitate was collected by filtration and dried overnight to provide **7** as a white solid. The filtrate left from the filtration was washed three times with 20 mL of DCM, and the combined organic layers were washed with brine, dried over $MgSO_4$, filtered, and dried to get the second portion of the product. The residue was dried to constant weight under a high vacuum to provide a second portion of **7** as a white solid. Both portions were combined to get the product (315 mg, 1.6 mmol, 85 %) **7** as a white solid. ^{19}F NMR (377 MHz, DMSO) δ ppm -114.88 (p, *J* = 7.8 Hz, 1F).

8, 1-(3-aminophenyl)ethan-1-ol, $C_8H_{11}NO$

To a solution of 3-amino acetophenone (2 g, 14.7 mmol, 1 eq) in MeOH (60 mL), $NaBH_4$ (0.94 g, 24.9 mmol, 1.7 eq) was added portion-wise at 0 °C and stirred at r.t. for 5 h. After the completion of the reaction, the solvent was evaporated under reduced pressure, and the resulting reaction mixture was extracted with water (50 mL) and EtOAc (70 mL x 3). The combined extracts were washed with brine (50 mL), dried over $MgSO_4$, filtered, and concentrated under reduced pressure to give a light yellowish solid **8** (1.8 g, 13.1 mmol, 89 %). ^1H NMR (400 MHz, $CDCl_3$) δ ppm 7.13 (t, *J* = 7.8 Hz, 1H), 6.80 – 6.67 (m, 2H), 6.59 (ddd, *J* = 7.9, 2.4, 1.0 Hz, 1H), 4.80 (q, *J* = 6.4 Hz, 1H), 3.06 (s, 3H), 1.47 (d, *J* = 6.4 Hz, 3H).

(S)-8, (S)-1-(3-aminophenyl)ethan-1-ol, $C_8H_{11}NO$

Solid 3-amino acetophenone (500 mg, 3.7 mmol, 1 eq) and *(R)*-RUCY-xylBINAP (0.4 mg, 0.4 μmol) were placed in a 100-mL stainless-steel autoclave equipped with a glass inner lining and a magnetic stirring bar. Air present in the autoclave was replaced by argon. Isopropanol (5-8 mL) was added and stirred until the starting material was fully dissolved, and then *t*-BuOK (4.15 mg, 0.037 mmol, 0.1 eq) was added. The air present in the gas inlet tube was removed by flushing with a stream of hydrogen. Hydrogen was initially introduced into the reaction carefully and this process was repeated five times until the vessel was optimally pressurized with H_2. The reaction mixture was vigorously stirred at r.t. for 8 h. After carefully venting the hydrogen gas, the solvent was removed under reduced pressure. The residue was purified by silica gel column chromatography and eluted with 1/1 diethyl ether/hexane to give ***(S)*-8** (yellowish solid, 350 mg, 69 % yield, > 99 % ee). ^1H NMR (400 MHz, $CDCl_3$) δ ppm 7.13 (t, *J* = 7.7 Hz, 1H), 6.80 – 6.68 (m, 2H), 6.65 – 6.51 (m, 1H), 4.80 (q, *J* = 6.5 Hz, 1H), 3.67 (s, 2H), 1.47 (d, *J* = 6.4 Hz, 3H).

(R)-8, (R)-1-(3-aminophenyl)ethan-1-ol, $C_8H_{11}NO$

Same as ***(S)*-8**, with *(S)*-RUCY-xylBINAP, Yield 74 %, > 99 % ee. ^1H NMR (400 MHz, $CDCl_3$) δ ppm 7.12 (t, *J* = 7.7 Hz, 1H), 6.80 – 6.66 (m, 2H), 6.59 (ddd, *J* = 7.9, 2.4, 1.0 Hz, 1H), 4.79 (q, *J* = 6.4 Hz, 1H), 3.03 (s, 2H), 1.46 (d, *J* = 6.5 Hz, 3H).

9, 1-[(4-fluorophenyl)methyl]-N-{3-[1-hydroxyethyl]phenyl}-7- methyl-5-(1H-pyrrol-2-ylcarbonyl)-4,5,6,7-tetrahydro-1H-pyrazolo[4,3-c]pyridine-3- carboxamide, $C_{28}H_{28}FN_5O_3$

To a solution of **7** (50 mg, 0.1 mmol, 1 eq) in anhydrous DMF (3 mL), were added pyBOP (67.5 mg, 0.13 mmol, 1.3 eq), and *i*-Pr_2NEt (53 μL, 0.3 mmol, 3 eq) and stirred for 15 min under argon. Afterwards, **8** or **(*S*)-8** or **(*R*)-8** (41 mg, 0.13 mmol, 1.3 eq) was added and the reaction mixture was stirred at r.t. for 18 h. The reaction mixture was rotary evaporated to remove DMF. The resulting residue was washed with DCM (10 mL x 2) and water (5 mL). The combined organic layer was washed with brine (5 mL), dried over $MgSO_4$, filtered and evaporated under vacuum to give the crude product which was subjected to ChiralPaK

IA (250 x 10 mm) with 50 % of MeCN in 20 mM NH_4OAc_{aq} flow rate 4 mL/min at 268 nm to provide (*R,S*)-**9**, (*R,R*)-**9**, (*S,R*)-**9** and (*S,S*)-**9**.

(R,S)-**9** (12 mg, 0.02 mmol, yield 24 %). ^{1}H NMR (400 MHz, $CDCl_3$) δ ppm 9.45 (s, NH), 8.67 (s, NH), 7.70 – 7.60 (m, 2H), 7.37 (t, *J* = 7.8 Hz, 1H), 7.20 – 7.10 (m, 3H), 7.08 (t, *J* = 8.6 Hz, 2H), 6.99 – 6.92 (m, 1H), 6.89 (s, 1H), 6.33 (d, *J* = 3.7 Hz, 1H), 5.59 (d, *J* = 15.9 Hz, 1H), 5.33 (q, *J* = 15.5 Hz, 2H), 4.94 (dd, *J* = 13.5, 7.9 Hz, 2H), 4.54 (d, *J* = 11.4 Hz, 1H), 3.38 (d, *J* = 11.6 Hz, 1H), 3.07 – 2.96 (m, 1H), 1.55 (d, *J* = 6.4 Hz, 3H), 1.24 (s, 3H). LCMS(ESI)$^+$ m/e 502.3 $[M+H]^+$. HRMS (ESI$^+$): *m/z* (%) = 502.2224, calculated 502.2176 for $C_{28}H_{29}FN_5O_3$ $[M+H]^+$.

(*R,R*)-**9** (11 mg, 0.022 mmol, yield 22 %).^{1}H NMR (300 MHz, $CDCl_3$) δ 9.54 (s, NH), 8.66 (s, NH), 7.70 – 7.55 (m, 2H), 7.33 (t, *J* = 7.8 Hz, 1H), 7.22 – 6.99 (m, 5H), 6.93 (td, *J* = 2.7, 1.2 Hz, 1H), 6.86 (s, 1H), 6.30 (dt, *J* = 3.7, 2.6 Hz, 1H), 5.55 (d, *J* = 16.5 Hz, 1H), 5.43 – 5.17 (m, 2H), 5.01 – 4.81 (m, 2H), 4.50 (dd, *J* = 13.0, 2.7 Hz, 1H), 3.35 (d, *J* = 10.9 Hz, 1H), 2.98 (dt, *J* = 6.4, 3.1 Hz, 1H), 1.51 (d, *J* = 6.4 Hz, 3H), 1.21 (d, *J* = 6.9 Hz, 3H).

(*S,R*)-**9** (15 mg, 0.03 mmol, yield 30 %). ^{1}H NMR (400 MHz, $CDCl_3$) δ ppm 9.45 (s, NH), 8.65 (s, NH), 7.68 – 7.58 (m, 2H), 7.39 – 7.29 (m, 1H), 7.18 – 6.99 (m, 5H), 6.93 (d, *J* = 3.5 Hz, 1H), 6.86 (s, 1H), 6.31 (s, 1H), 5.56 (d, *J* = 15.6 Hz, 1H), 5.29 (m, 2H), 4.93 (d, *J* = 5.6 Hz, 2H), 4.55 – 4.47 (m, 1H), 3.35 (d, *J* = 11.7 Hz, 1H), 2.98 (d, *J* = 7.3 Hz, 1H), 1.52 (d, *J* = 6.4 Hz, 3H), 1.21 (d, *J* = 3.0 Hz, 3H). LCMS(ES)$^+$ m/e 502.3 $[M+H]^{+.}$ HRMS (ESI$^+$): *m/z* (%) = 502.2219, calculated 502.2176 for $C_{28}H_{29}FN_5O_3$ $[M+H]^+$

(*S,S*)-**9** (10 mg, 0.02 mmol, yield 20 %).1H NMR (400 MHz, CDCl3) δ 9.52 (s, NH), 8.66 (s, NH), 7.67 – 7.54 (m, 2H), 7.33 (t, J = 7.8 Hz, 2H), 7.17 – 6.99 (m, 5H), 6.93 (td, J = 2.7, 1.2 Hz, 2H), 6.85 (s, 1H), 6.37 – 6.25 (m, 1H), 5.55 (d, J = 16.0 Hz, 1H), 5.38 – 5.17 (m, 2H), 4.99 – 4.82 (m, 2H), 4.50 (d, J = 10.8 Hz, 1H), 3.35 (d, J = 12.2 Hz, 1H), 3.05 – 2.88 (m, 1H), 1.51 (d, J = 6.4 Hz, 3H), 1.21 (d, J = 7.0 Hz, 3H).

General procedure A:

To the solution of **16** (0.13 mmol,1 eq) in anhydrous DMF, were added pyBOP (0.16 mmol, 1.2 eq), and *i*-Pr_2NEt (0.52 mmol, 4 eq) and stirred for 15 min at 0 °C under argon. Afterwards, respective amine (1.2 eq) was added and the reaction mixture was stirred at r.t. for overnight. The reaction mixture was rotary evaporated to remove anhydrous DMF and then was washed with water (20 mL) and EtOAc (30 mL x 3). The combined organic layer was washed with brine (10 mL), dried over $MgSO_4$, filtered and evaporated under vacuum to give the crude product which was purified on silica gel with an increasing gradient of Hex/EtOAc (5/1 → 1/3).

General procedure B:

To a solution of **46** (0.11 mmol, 1 eq) in dry THF was added Cs_2CO_3 (0.44 mmol, 4 eq) with stirring under argon at r.t. for 1 h. Afterwards, the respective alkyl or aryl bromide (1.2 eq) was added and the reaction mixture was stirred overnight (15 h) under argon. The reaction mixture was rotary evaporated to remove THF and then extracted with two portions of EtOAc (30 mL x 2) and water (15 mL). The combined extracts were washed with brine (10 mL), dried over $MgSO_4$, filtered and concentrated under reduced pressure. The residue was purified by silica gel chromatography with an increasing gradient of Hex/EtOAc (5/1 → 1/2). The appropriate fractions were combined, and concentrated under reduced pressure and the residue was dried to constant weight under vacuum to provide the respective product.

General procedure C:

To a solution of respective carboxylic acid (1.2 eq) in anhydrous DMF, were added pyBOP (0.14 mmol, 1.2 eq), and *i*-Pr_2NEt (0.48 mmol, 4 eq) and stirred for 15 min at 0 °C under Argon. Afterwards, **54** (0.12 mmol, 1.0 eq) was added and the reaction mixture was stirred overnight at r.t. under Argon. The

reaction mixture is rotary evaporated to remove anhydrous DMF and then washed with water (20 mL) and EtOAc (30 mL x 3). The combined organic layer was washed with brine (10 mL), dried over $MgSO_4$, filtered and evaporated under vacuum to give the crude product which was purified on silica gel with an increasing gradient of Hex/EtOAc (5/1 → 1/4).

11, tert-butyl 5-(2-ethoxy-2-oxoacetyl)-3,3-dimethyl-4-oxopiperidine-1-carboxylate, $C_{16}H_{25}NO_6$

To a solution of commercially available **10** (2 g, 8.9 mmol, 1 eq)) in THF (10 mL) was added a solution of 2M LDA in THF (5.1 mL, 10.1 mmol) at –78 °C, the mixture was stirred at –78 °C for 1 h and diethyl oxalate (1.43 mL, 10.6 mmol) was added. The resulting mixture was allowed to warm to r.t. and stirred overnight. The reaction was quenched with ice cubes. The reaction mixture was then extracted with an aqueous solution of 25 % citric acid (~pH 5) and EtOAc (100 mL x 3), and the organic layer was collected, washed with brine (100 mL), dried over $MgSO_4$, and concentrated under reduced pressure to get **11** (2.5 g, 85.2 %). ^1H NMR (400 MHz, $CDCl_3$) δ ppm 4.45 (s, 2H), 4.35 (q, *J* = 7.2 Hz, 2H), 3.39 (s, 2H), 1.47 (s, 9H), 1.38 (t, *J* = 7.2 Hz, 3H), 1.21 (s, 6H). ^{13}C NMR (101 MHz, $CDCl_3$) δ ppm 162.31, 105.18, 80.34, 62.42, 40.89, 28.36, 23.66, 14.08. HRMS (ESI^+): *m/z* (%) = 328.1691, calculated 328.1682 for $C_{16}H_{26}NO_6$ $[M+H]^+$

12, 5-(tert-butyl) 3-ethyl 7,7-dimethyl-1,4,6,7-tetrahydro-5H-pyrazolo[4,3-c]pyridine-3,5-dicarboxylate, $C_{16}H_{25}N_3O_4$

To a solution of **11** (1.5 g, 4.5 mmol) in acetic acid (3 mL) was added hydrazine hydrate (0.5 mL, 10.7 mmol). After 2 h, the reaction mixture was concentrated under reduced pressure and the residue was poured into ice-cold saturated aqueous $NaHCO_3$ (50 mL) and extracted with three portions of EtOAc (50 mL x 3). The combined extracts were washed with brine (30 mL), dried over $MgSO_4$, filtered, concentrated under reduced pressure and dried to constant weight to provide **12** (1.3 g, 4.1 mmol, 92.2 % yield) as a pale yellow solid. ^1H NMR (400 MHz, $CDCl_3$) δ ppm 8.78 (s, NH), 4.67 (s, 2H), 4.35 (q, J = 7.2 Hz, 2H), 3.39 (s, 2H), 1.49 (s, 9H), 1.37 (t, *J* = 7.2 Hz, 3H), 1.30 (s, 6H). HRMS (ESI+): *m/z* (%) = 323.19850, calculated 323.1845 for $C_{16}H_{26}N_3O_4$ $[M+H]^+$

13, 5-(tert-butyl) 3-ethyl 1-(4-fluorobenzyl)-7,7-dimethyl-1,4,6,7-tetrahydro-5H-pyrazolo[4,3-c]pyridine-3,5-dicarboxylate, $C_{23}H_{30}FN_3O_4$

To a solution of **12** (1.3 g, 4.0 mmol) in dry THF (10 mL) was added Cs_2CO_3 (6.2 g, 19.3 mmol) with stirring under argon. After 1 h, 1-(bromomethyl)-4-fluorobenzene (0.6 mL, 8.0 mmol) was added and the reaction mixture was stirred overnight (15 h) under argon. The reaction mixture was extracted with two portions of EtOAc (60 mL x 2) and water (50 mL). The combined extracts were washed with brine (40 mL), dried over $MgSO_4$, filtered and concentrated under reduced pressure. The residue was purified by silica gel chromatography (Hex/EtOAc 5/1→ EtOAc). The appropriate fractions were combined, and concentrated under reduced pressure and the residue was dried to constant weight under high vacuum to provide **13** (0.94 g, 2.2 mmol, 57 % yield) as a light yellow foam. ^1H NMR (400 MHz, $CDCl_3$) δ ppm 7.03-6.93 (d, *J* = 8.1 Hz, 4H, Ar), 5.47 (s, 2H), 4.67 – 4.60 (q, J = 7.2 Hz, 2H), 3.35 (s, 2H), 1.47 (s, 9H), 1.39 (t, *J* = 7.2 Hz, 3H), 1.12 (s, 6H). ^{19}F NMR (377 MHz, $CDCl_3$) δ ppm -114.49. HRMS (ESI+): *m/z* (%) = 431.2233, calculated 431.2220 for $C_{23}H_{31}FN_3O_4$ $[M+H]^+$

14, 3-(ethoxycarbonyl)-1-(4-fluorobenzyl)-7,7-dimethyl-4,5,6,7-tetrahydro-1H-pyrazolo[4,3-c]pyridine hydrochloride, $C_{18}H_{23}FN_3O_2Cl$

To a solution of **13** (0.84 g, 1.94 mmol) in 1,4-dioxane was added 4 N HCl in 1,4-dioxane (5.4 mL). The reaction mixture was stirred at r.t. for 2 h. The solvent was removed under reduced pressure and dried to constant weight under high vacuum to yield **14** as a golden yellow foam (0.71 g, 100 %). This material was used without further purification. ^1H NMR (400 MHz, DMSO) δ ppm 9.80 (s, NH), 7.29 (d, *J* = 8.8 Hz, 1H),

5.67 (s, 2H), 4.40 (q, J = 7.1 Hz, 2H), 4.32 (s, 2H), 3.29 (s, 2H), 1.44 (s, 6H), 1.40 (t, J = 7.1 Hz, 3H). ^{13}C NMR (101 MHz, DMSO) δ ppm 163.19, 161.93, 160.77, 144.50, 138.40, 133.62, 133.59, 129.22, 129.14, 115.98, 115.76, 112.43, 66.83, 61.04, 54.18, 53.18, 31.65, 25.47, 14.66. HRMS (ESI+): *m/z* (%) = 367.1420, calculated 367.1463 for $C_{18}H_{24}FN_3O_2Cl$ [M+H]$^+$.

15, ethyl 1-(4-fluorobenzyl)-7,7-dimethyl-5-(1H-pyrrole-2-carbonyl)-4,5,6,7-tetrahydro-1H-pyrazolo[4,3-c]pyridine-3-carboxylate, $C_{23}H_{25}FN_4O_3$

To the solution of 1*H*-pyrrole-2-carboxylic acid (344 mg, 3.1 mmol) in DMF (10 mL), were added pyBOP (1.6 g, 3.1 mmol), and *i*-Pr_2NEt (1.5 mL, 8.4 mmol) and stirred for 1 h under argon. Afterwards, **14** (760 mg, 2.1 mmol) in DMF (5 mL) was added and the reaction mixture was stirred at r.t. for 18 h. The reaction mixture was rotary evaporated to remove DMF. The resulting residue was washed with EtOAc (60 mL x 3) water (60 mL). The combined organic layer was washed with brine (40 mL), dried over $MgSO_4$, filtered and evaporated under vacuum to give a crude product which was purified on silica gel (Hex/EtOAc 5:6 →EtOAc). The combined fractions provided 850 mg of **15** (95.3 %). ^{1}H NMR (400 MHz, $CDCl_3$) δ ppm 9.75 (s, NH), 7.09 (m, 4H) – 6.94 (m, 1H), 6.79 (s, 1H), 6.33 (dt, J = 3.8, 2.7 Hz, 1H), 5.51 (s, 2H), 5.16 (s, 2H), 4.46 (q, J = 7.1 Hz, 2H), 3.75 (s, 2H), 1.46 (t, J = 7.1 Hz, 3H), 1.21 (s, 6H). ^{13}C NMR (101 MHz, $CDCl_3$) δ ppm 163.56, 162.34, 161.11, 146.09, 139.98, 138.19, 132.17, 132.14, 128.45, 128.08, 127.99, 126.63, 126.59, 124.27, 121.62, 116.84, 116.64, 115.85, 115.64, 113.23, 111.41, 110.08, 61.10, 54.85, 33.94, 29.71, 24.95, 14.47. HRMS (ESI+): *m/z* (%) = 425.1921, calculated 425.1911 for $C_{23}H_{26}FN_4O_3$ [M+H]$^+$.

16, 1-(4-fluorobenzyl)-7,7-dimethyl-5-(1H-pyrrole-2-carbonyl)-4,5,6,7-tetrahydro-1H-pyrazolo[4,3-c]pyridine-3-carboxylic acid, $C_{21}H_{21}FN_4O_3$

To a solution of **15** (850 g, 2.0 mmol) in EtOH (10 mL) was added 1N NaOH (6 mL, 4 mmol). The clear solution was stirred at r.t. for 2 h, afterwards, the reaction was judged complete by LCMS. The EtOH was removed under reduced pressure and the residue was diluted with water and pH adjusted to ~6 with aqueous 1 N HCl. The resulting precipitate was collected by filtration and dried overnight to provide **16** as a white solid. The filtrate left from the filtration was washed three times with 20 mL of DCM, and the combined organic layers were washed with brine (10 mL), dried over $MgSO_4$, filtered and dried to get the second portion of the product. The residue was dried to constant weight under vacuum to provide a second portion of **16** as a white solid. Both portions were combined to get the product **16** (634 mg, 1.6 mmol, 80 %) as a white solid. ^{1}H NMR (400 MHz, DMSO) δ ppm 11.63 (s, 1H), 7.34 – 7.17 (m, 4H), 7.01 (td, J = 2.7, 1.4 Hz, 1H), 6.65 (s, 1H), 6.26 (dd, J = 4.3, 1.8 Hz, 1H), 5.60 (s, 2H), 5.01 (s, 2H), 3.78 (s, 2H), 1.30 (s, 6H). HRMS (ESI+): *m/z* (%) = 397.1601, calculated 397.1598 for $C_{21}H_{22}FN_4O_3$ [M+H]$^+$

17, 1-(4-fluorobenzyl)-N-(4-methoxy-3,5-dimethylphenyl)-7,7-dimethyl-5-(1H-pyrrole-2-carbonyl)-4,5,6,7-tetrahydro-1H-pyrazolo[4,3-c]pyridine-3-carboxamide, $C_{30}H_{32}FN_5O_3$

General procedure A: Yield 67 %. ^{1}H NMR (400 MHz, $CDCl_3$) δ ppm 9.48 (s, NH), 8.47 (s, NH), 7.29 (s, 2H), 7.03 (d, J = 6.8 Hz, 4H), 6.93 (d, J = 3.0 Hz, 1H), 6.88 (s, 1H), 6.30 (q, J = 2.9 Hz, 1H), 5.42 (s, 2H), 5.25 (s, 2H), 3.74 (s, 2H), 3.70 (s, 3H), 2.29 (s, 6H), 1.26 (s, 6H). ^{13}C NMR (101 MHz, $CDCl_3$) δ ppm 162.38 (d, J_{C-F} = 246.9 Hz), 162.05, 160.00, 153.61, 146.80, 140.77, 133.06, 132.34 (d, J_{C-F} = 4 Hz), 128.05 (d, J_{C-F} = 8.2 Hz), 124.56, 121.13, 120.42, 115.96, 115.85 (d, J_{C-F} = 21.7 Hz), 113.44, 110.27, 59.84, 54.45, 33.99, 29.71, 25.12, 16.21. ^{19}F NMR (377 MHz, $CDCl_3$) δ ppm -114.12 (d, J = 8.1 Hz). HRMS (ESI+): *m/z* (%) = 530.2559, calculated 530.2489 for $C_{30}H_{33}FN_5O_3$ [M+H]$^+$

18, 1-(4-fluorobenzyl)-N-(3-(fluoromethyl)phenyl)-7,7-dimethyl-5-(1H-pyrrole-2-carbonyl)-4,5,6,7-tetrahydro-1H-pyrazolo[4,3-c]pyridine-3-carboxamide, $C_{28}H_{27}F_2N_5O_2$

General procedure A: Yield 62 %. ^{1}H NMR (400 MHz, $CDCl_3$) δ ppm 9.55 (s, NH), 8.67 (s, NH), 7.72 (d, *J* = 2.0 Hz, 1H), 7.66 – 7.61 (m, 1H), 7.41 – 7.35 (m, 1H), 7.14 (d, *J* = 7.6 Hz, 1H), 7.04 (d, *J* = 6.8 Hz, 4H), 6.93 (td, *J* = 2.7, 1.2 Hz, 1H), 6.87 (s, 1H), 6.31 (dt, *J* = 3.8, 2.6 Hz, 1H), 5.45 (s, 1H), 5.45 – 5.41 (m, 2H), 5.33 (s, 1H), 5.25 (s, 2H), 3.75 (s, 2H), 1.26 (s, 6H). ^{13}C NMR (101 MHz, $CDCl_3$) δ ppm 162.37 (d, $J_{C\text{-}F}$ = 247.0 Hz), 162.05, 160.14, 146.94, 140.52, 137.97, 137.39, 137.22, 132.22 (d, $J_{C\text{-}F}$ = 3.3 Hz), 129.35, 128.04 (d, $J_{C\text{-}F}$ = 8.1 Hz), 124.51, 122.92, 122.86, 121.18, 119.92, 119.89, 118.51, 118.45, 115.86 (d, $J_{C\text{-}F}$ = 21.7 Hz), 115.63, 113.35, 110.25, 85.12, 83.46, 54.48, 33.99, 29.68, 25.09. ^{19}F NMR (376 MHz, $CDCl_3$) δ ppm 20.74, 20.61, 20.48, -114.02 (p, *J* = 7.1 Hz).

SK60 (19), 1-(4-fluorobenzyl)-7,7-dimethyl-5-(1H-pyrrole-2-carbonyl)-N-(m-tolyl)-4,5,6,7-tetrahydro-1H-pyrazolo[4,3-c]pyridine-3-carboxamide, $C_{28}H_{28}FN_5O_2$

General procedure A: Yield 88 %, ^{1}H NMR (400 MHz, $CDCl_3$) δ ppm 9.51 (s, NH), 8.57 (s, NH), 7.51 (d, *J* = 2.1 Hz, 1H), 7.43 (dd, *J* = 8.0, 2.4 Hz, 1H), 7.22 (d, *J* = 7.8 Hz, 1H), 7.04 (d, *J* = 6.9 Hz, 4H), 6.93 (t, *J* = 1.4 Hz, 1H), 6.88 (s, 1H), 6.31 (dt, *J* = 3.8, 2.7 Hz, 1H), 5.43 (d, *J* = 1.1 Hz, 2H), 5.26 (s, 2H), 3.75 (s, 2H), 2.37 (s, 3H), 1.26 (s, 9H). ^{13}C NMR (76 MHz, $CDCl_3$) δ ppm 162.38 (d, $J_{C\text{-}F}$ = 247 Hz), 162.05, 160.08, 146.84, 140.75, 139.00, 137.57, 132.33 (d, $J_{C\text{-}F}$ = 3.4 Hz), 128.04 (d, $J_{C\text{-}F}$ = 8.2 Hz), 127.99, 125.01, 124.54, 121.16, 120.40, 116.91, 116.01, 115.86 (d, $J_{C\text{-}F}$ = 22 Hz), 113.43, 110.29, 54.47, 34.00, 29.71, 25.11, 21.54. ^{19}F NMR (377 MHz, $CDCl_3$) δ ppm -113.99. HRMS (ESI+): *m/z* (%) = 486.2282, calculated 486.2227 for $C_{28}H_{29}FN_5O_2$ $[M+H]^+$

20, 1-(4-fluorobenzyl)-N-(3-hydroxyphenyl)-7,7-dimethyl-5-(1H-pyrrole-2-carbonyl)-4,5,6,7-tetrahydro-1H-pyrazolo[4,3-c]pyridine-3-carboxamide, $C_{27}H_{26}FN_5O_3$

General procedure A: Yield 70 %. ^{1}H NMR (300 MHz, $CDCl_3$) δ ppm 9.59 (s, NH), 8.66 (s, NH), 7.65 (s, 1H), 7.18 (t, *J* = 8.1 Hz, 1H), 7.03 (d, *J* = 6.8 Hz, 4H), 6.90 (s, 2H), 6.86 (d, *J* = 7.4 Hz, 1H), 6.63 (d, *J* = 7.4 Hz, 1H), 6.19 (d, *J* = 3.5 Hz, 1H), 5.43 (s, 2H), 5.26 (s, 2H), 3.75 (s, 2H), 1.34 (s, 6H). ^{13}C NMR (101 MHz, $CDCl_3$) δ ppm 162.10, 160.19, 156.57, 138.76, 129.97, 128.06 (d, $J_{C\text{-}F}$ = 8.1 Hz), 124.78, 124.48, 121.22, 115.89 (d, $J_{C\text{-}F}$ = 21.6 Hz), 111.68, 111.36, 110.29, 107.05, 54.52, 31.94, 29.71, 25.10, 22.70. ^{19}F NMR (282 MHz, $CDCl_3$) δ ppm -114.01 (p, *J* = 7.1 Hz). HRMS (ESI+): *m/z* (%) = 488.2106, calculated 488.2020 for $C_{27}H_{27}FN_5O_3$ $[M+H]^+$

21, (S)-1-(4-fluorobenzyl)-N-(3-(1-hydroxyethyl)phenyl)-7,7-dimethyl-5-(1H-pyrrole-2-carbonyl)-4,5,6,7-tetrahydro-1H-pyrazolo[4,3-c]pyridine-3-carboxamide, $C_{29}H_{30}FN_5O_3$

General procedure A: Yield 63 %, ^{1}H NMR (400 MHz, $CDCl_3$) δ ppm 9.49 (s, NH), 8.64 (s, NH), 7.72 – 7.55 (m, 2H), 7.33 (t, *J* = 7.8 Hz, 1H), 7.13 (d, *J* = 7.6 Hz, 1H), 7.03 (s, 4H), 6.93 (dt, *J* = 3.6, 1.8 Hz, 1H), 6.87 (s, 1H), 6.31 (q, *J* = 2.9 Hz, 1H), 5.43 (s, 2H), 5.24 (s, 2H), 4.92 (q, *J* = 6.5 Hz, 1H), 3.74 (s, 2H), 1.51 (d, *J* = 6.4 Hz, 3H), 1.25 (s, 6H). ^{3}C NMR (101 MHz, $CDCl_3$) δ ppm 162.37 (d, $J_{C\text{-}F}$ = 246.8 Hz), 162.03, 160.09, 147.03, 146.88, 140.63, 137.82, 132.25 (d, $J_{C\text{-}F}$ = 3.2 Hz), 129.23, 128.03 (d, $J_{C\text{-}F}$ = 8.2 Hz), 124.52, 121.12, 118.84, 116.74, 115.85 (d, $J_{C\text{-}F}$ = 21.8 Hz), 115.57, 113.38, 110.26, 70.23, 54.46, 33.98, 29.69, 25.23, 25.09. ^{19}F NMR (376 MHz, $CDCl_3$) δ ppm -114.05 (p, *J* = 7.1 Hz).

22, N-(3-ethylphenyl)-1-(4-fluorobenzyl)-7,7-dimethyl-5-(1H-pyrrole-2-carbonyl)-4,5,6,7-tetrahydro-1H-pyrazolo[4,3-c]pyridine-3-carboxamide, $C_{29}H_{30}FN_5O_2$

General procedure A: Yield 80 %, ^{1}H NMR (400 MHz, $CDCl_3$) δ ppm 9.64 (s, NH), 8.60 (s, NH), 7.48 (m, 2H), 7.28 (s, 1H), 7.25 (s, 1H), 7.03 (d, *J* = 7.0 Hz, 4H), 6.93 (dt, *J* = 2.7, 1.4 Hz, 1H), 6.88 (s, 1H), 6.30 (dt, *J* = 3.8, 2.7 Hz, 1H), 5.43 (s, 2H), 5.26 (s, 2H), 3.75 (s, 2H), 2.66 (q, *J* = 7.6 Hz, 2H), 1.27-1.25 (s, 9H). ^{13}C NMR (101 MHz, $CDCl_3$) δ ppm 163.60, 162.13, 161.15, 160.08, 146.84, 145.38, 140.79, 137.65, 132.37, 132.33, 128.98, 128.07, 127.99, 124.51, 123.79, 121.24, 119.31, 117.23, 115.97, 115.75, 115.56, 113.47, 110.23,

54.46, 34.00, 29.71, 28.92, 25.12, 15.59. ^{19}F NMR (377 MHz, $CDCl_3$) δ ppm -114.11. HRMS (ESI+): *m/z* (%) = 500.2437, calculated 500.2384 for $C_{29}H_{31}FN_5O_2$ $[M+H]^+$

23, 1-(4-fluorobenzyl)-7,7-dimethyl-N-phenyl-5-(1H-pyrrole-2-carbonyl)-4,5,6,7-tetrahydro-1H-pyrazolo[4,3-c]pyridine-3-carboxamide, $C_{27}H_{26}FN_5O_2$

General procedure A: Yield 74 %. ^1H NMR (400 MHz, $CDCl_3$) δ ppm 9.54 (s, NH), 8.61 (s, NH), 7.65 (d, *J* = 7.9 Hz, 2H), 7.35 (t, *J* = 8.0 Hz, 2H), 7.12 (t, *J* = 7.4 Hz, 1H), 7.04 (d, *J* = 6.9 Hz, 4H), 6.91 (d, *J* = 22.1 Hz, 2H), 6.30 (q, *J* = 2.8 Hz, 1H), 5.44 (s, 2H), 5.26 (s, 2H), 3.75 (s, 2H), 1.26 (s, 6H). ^{13}C NMR (101 MHz, $CDCl_3$) δ ppm 163.62, 162.06, 161.16, 160.13, 146.88, 140.71, 137.65, 132.33, 132.30, 129.07, 128.09, 128.01, 124.56, 124.19, 121.17, 119.85, 115.98, 115.76, 115.62, 113.41, 110.28, 54.48, 34.01, 29.71, 25.11. ^{19}F NMR (377 MHz, $CDCl_3$) δ ppm -114.06. HRMS (ESI+): *m/z* (%) = 472.2119, calculated 472.2071 for $C_{27}H_{27}FN_5O_2$ $[M+H]^+$

24, 1-(4-fluorobenzyl)-N-(3-(1-hydroxyethyl)phenyl)-7,7-dimethyl-5-(1H-pyrrole-2-carbonyl)-4,5,6,7-tetrahydro-1H-pyrazolo[4,3-c]pyridine-3-carboxamide, $C_{29}H_{30}FN_5O_3$

General procedure A: Yield 70 %, ^1H NMR (400 MHz, $CDCl_3$) δ ppm 9.45 (s, NH), 8.64 (s, NH), 7.68 – 7.55 (m, 2H), 7.34 (t, *J* = 7.8 Hz, 1H), 7.14 (d, *J* = 7.7 Hz, 1H), 7.04 (d, J = 6.9 Hz, 4H), 6.94 (td, *J* = 2.7, 1.2 Hz, 1H), 6.88 (s, 1H), 6.31 (dt, *J* = 3.8, 2.6 Hz, 1H), 5.46 – 5.39 (m, 2H), 5.25 (s, 2H), 4.92 (q, *J* = 6.5 Hz, 1H), 3.75 (s, 2H), 1.51 (d, *J* = 6.4 Hz, 3H), 1.26 (s, 6H). HRMS (ESI+): *m/z* (%) = 516.2397, calculated 516.2333 for $C_{29}H_{31}FN_5O_3$ $[M+H]^+$

25, 1-(4-fluorobenzyl)-7,7-dimethyl-5-(1H-pyrrole-2-carbonyl)-N-(3-(trifluoromethyl)phenyl)-4,5,6,7-tetrahydro-1H-pyrazolo[4,3-c]pyridine-3-carboxamide, $C_{28}H_{25}F_4N_5O_2$

General procedure A: Yield 53 %. ^1H NMR (400 MHz, $CDCl_3$) δ ppm 9.47 (s, NH), 8.72 (s, NH), 7.97 – 7.84 (m, 2H), 7.47 (t, *J* = 7.9 Hz, 1H), 7.37 (d, *J* = 7.8 Hz, 1H), 7.04 (d, *J* = 7.5 Hz, 4H), 6.94 (td, *J* = 2.7, 1.2 Hz, 1H), 6.87 (s, 1H), 6.33 (q, *J* = 3.0 Hz, 1H), 5.44 (s, 2H), 5.25 (s, 2H), 3.76 (s, 2H), 1.27 (s, 6H). ^{13}C NMR (101 MHz, $CDCl_3$) δ ppm 163.62, 162.01, 160.16, 147.09, 140.26, 138.23, 132.11, 129.58, 128.05, 127.97, 124.49, 122.72, 121.18, 120.63, 116.37, 116.00, 115.78, 113.30, 110.30, 54.52, 34.02, 29.68, 25.08. ^{19}F NMR (376 MHz, $CDCl_3$) δ ppm -62.68, -113.93 (p, *J* = 6.9 Hz).

26, 1-(4-fluorobenzyl)-N-(3-fluorophenyl)-7,7-dimethyl-5-(1H-pyrrole-2-carbonyl)-4,5,6,7-tetrahydro-1H-pyrazolo[4,3-c]pyridine-3-carboxamide, $C_{27}H_{25}F_2N_5O_2$

General procedure A: Yield 85 %, ^1H NMR (400 MHz, $CDCl_3$) δ ppm 9.55 (s, NH), 8.63 (s, NH), 7.59 (d, *J* = 11.9 Hz, 1H), 7.25 (d, J =10.3, 1H), 7.04-6.82 (m, 8H), 6.30 (s, 1H), 5.41 (d, *J* = 1.1 Hz, 2H), 5.23 (s, 2H), 3.73 (s, 2H), 1.24 (s, 6H). ^{13}C NMR (101 MHz, $CDCl_3$) δ ppm 164.31, 163.63, 162.08, 161.88, 161.18, 160.10, 147.02, 140.41, 139.26, 139.15, 132.22, 132.19, 130.16, 130.06, 128.08, 128.00, 124.49, 121.25, 116.01, 115.79, 115.73, 115.04, 115.01, 113.39, 110.95, 110.74, 110.31, 107.33, 107.07, 54.52, 34.03, 29.71, 25.10. ^{19}F NMR (377 MHz, $CDCl_3$) δ ppm -111.46, -113.95. HRMS (ESI+): *m/z* (%) = 490.2013, calculated 490.1976 for $C_{27}H_{26}F_2N_5O_2$ $[M+H]^+$

27, 1-(4-fluorobenzyl)-N-(2-fluorophenyl)-7,7-dimethyl-5-(1H-pyrrole-2-carbonyl)-4,5,6,7-tetrahydro-1H-pyrazolo[4,3-c]pyridine-3-carboxamide, $C_{27}H_{25}F_2N_5O_2$

General procedure A: Yield 26 %. ^1H NMR (400 MHz, $CDCl_3$) δ ppm 9.46 (s, NH), 8.87 (s, NH), 8.42 (t, *J* = 8.0 Hz, 1H), 7.23 – 6.98 (m, 7H), 6.94 (s, 1H), 6.87 (s, 1H), 6.35 – 6.27 (m, 1H), 5.45 (s, 2H), 5.25 (s, 2H), 3.75 (s, 2H), 1.26 (s, 6H). ^{13}C NMR (76 MHz, $CDCl_3$) δ ppm 164.05, 162.03, 160.78, 160.14, 154.29, 151.06, 146.82, 140.39, 132.23, 132.19, 128.27, 128.16, 126.31, 126.17, 124.56, 124.26, 124.16, 121.61, 121.15, 115.98, 115.70, 115.65, 115.08, 114.82, 113.32, 110.28, 54.61, 31.94, 34.01, 29.71, 25.10. ^{19}F NMR (377 MHz, $CDCl_3$) δ ppm -114.07, -130.81. HRMS (ESI+): *m/z* (%) = 490.2059, calculated 490.1976 for $C_{27}H_{26}F_2N_5O_2$ $[M+H]^+$

28, 1-(4-fluorobenzyl)-N-(4-fluorophenyl)-7,7-dimethyl-5-(1H-pyrrole-2-carbonyl)-4,5,6,7-tetrahydro-1H-pyrazolo[4,3-c]pyridine-3-carboxamide, $C_{27}H_{25}F_2N_5O_2$

General procedure A: Yield 82 %. ^{1}H NMR (400 MHz, $CDCl_3$) δ ppm 9.52 (s, NH), 8.58 (s, NH), 7.60 (dd, *J* = 8.9, 4.8 Hz, 2H), 7.03 (d, *J* = 7.1 Hz, 6H), 6.94 (d, *J* = 3.0 Hz, 1H), 6.86 (s, 1H), 6.30 (q, *J* = 3.0 Hz, 1H), 5.43 (s, 2H), 5.24 (s, 2H), 3.75 (s, 2H), 1.26 (s, 6H). ^{13}C NMR (75 MHz, $CDCl_3$) δ ppm 162.02, 160.06, 146.90, 133.61, 132.23, 128.06, 127.95, 124.50, 121.65, 121.55, 121.17, 116.01, 115.83, 115.72, 115.55, 113.34, 110.26, 54.47, 44.17, 33.99, 29.69, 25.09. ^{19}F NMR (377 MHz, $CDCl_3$) δ ppm -114.00 (t, *J* = 7.2 Hz), -118.19. HRMS (ESI+): *m/z* (%) = 490.2060, calculated 490.1976 for $C_{27}H_{26}F_2N_5O_2$ $[M+H]^+$

29, N-(3-fluoro-4-methylphenyl)-1-(4-fluorobenzyl)-7,7-dimethyl-5-(1H-pyrrole-2-carbonyl)-4,5,6,7-tetrahydro-1H-pyrazolo[4,3-c]pyridine-3-carboxamide, $C_{28}H_{27}F_2N_5O_2$

General procedure A: Yield 77 %. ^{1}H NMR (400 MHz, $CDCl_3$) δ ppm 9.51 (s, NH), 8.58 (s, NH), 7.54 (dd, *J* = 11.5, 2.1 Hz, 1H), 7.19 (dd, *J* = 8.2, 2.1 Hz, 1H), 7.12 (t, *J* = 8.3 Hz, 1H), 7.03 (d, *J* = 7.6 Hz, 4H), 6.94 (td, *J* = 2.7, 1.2 Hz, 1H), 6.87 (s, 1H), 6.31 (q, *J* = 2.7 Hz, 1H), 5.43 (s, 2H), 5.24 (s, 2H), 3.75 (s, 2H), 2.24 (d, *J* = 2.0 Hz, 3H), 1.26 (s, 6H). ^{13}C NMR (101 MHz, $CDCl_3$) δ ppm 163.62, 162.41, 162.04, 161.17, 160.02, 159.99, 146.95, 140.52, 136.78, 136.68, 132.27, 132.24, 131.41, 131.34, 128.08, 127.99, 124.54, 121.18, 120.43, 120.26, 115.99, 115.78, 115.65, 114.95, 114.91, 113.36, 110.31, 107.21, 106.94, 54.49, 34.01, 29.71, 25.11, 14.13, 14.10. ^{19}F NMR (377 MHz, $CDCl_3$) δ ppm -114.01 (p, *J* = 7.1 Hz), -115.47 (t, *J* = 10.0 Hz).

30, 1-(4-fluorobenzyl)-7,7-dimethyl-N-(pyridin-3-yl)-5-(1H-pyrrole-2-carbonyl)-4,5,6,7-tetrahydro-1H-pyrazolo[4,3-c]pyridine-3-carboxamide, $C_{26}H_{25}FN_6O_2$

General procedure A: Yield 75 %. ^{1}H NMR (400 MHz, $CDCl_3$) δ ppm 9.52 (s, NH), 8.68 (s, NH), 8.65 (s, 1H), 8.37 (d, *J* = 4.7 Hz, 1H), 8.30 (d, *J* = 8.2 Hz, 1H), 7.30 (dd, *J* = 8.4, 4.8 Hz, 1H), 7.04 (d, *J* = 6.8 Hz, 4H), 6.94 (d, *J* = 3.2 Hz, 1H), 6.86 (s, 1H), 6.31 (q, *J* = 3.0 Hz, 1H), 5.45 (s, 2H), 5.24 (s, 2H), 3.76 (s, 2H), 1.25 (s, 6H). ^{13}C NMR (101 MHz, $CDCl_3$) δ ppm 163.63, 162.02, 161.17, 160.43, 147.08, 145.17, 141.18, 140.16, 134.48, 132.11, 132.08, 128.06, 127.98, 126.91, 124.48, 123.67, 121.21, 116.01, 115.79, 115.76, 113.27, 110.27, 54.55, 34.02, 30.30, 29.68, 25.07. ^{19}F NMR (377 MHz, $CDCl_3$) δ ppm -113.87. HRMS (ESI+): *m/z* (%) = 473.2096, calculated 473.2023 for $C_{26}H_{26}FN_6O_2$ $[M+H]^+$

31, 1-(4-fluorobenzyl)-N-(3-methoxyphenyl)-7,7-dimethyl-5-(1H-pyrrole-2-carbonyl)-4,5,6,7-tetrahydro-1H-pyrazolo[4,3-c]pyridine-3-carboxamide, $C_{28}H_{28}FN_5O_3$

General procedure A: Yield 92 %. ^{1}H NMR (400 MHz, $CDCl_3$) δ ppm 9.58 (s, NH), 8.60 (s, NH), 7.36 (t, *J* = 2.3 Hz, 1H), 7.23 (d, *J* = 8.1 Hz, 1H), 7.15 (ddd, *J* = 8.0, 2.1, 1.0 Hz, 1H), 7.03 (d, *J* = 6.9 Hz, 4H), 6.94 (td, *J* = 2.7, 1.2 Hz, 1H), 6.87 (s, 1H), 6.68 (ddd, *J* = 8.2, 2.5, 1.0 Hz, 1H), 6.31 (dt, *J* = 3.8, 2.6 Hz, 1H), 5.43 (s, 2H), 5.25 (s, 2H), 3.83 (s, 3H), 3.74 (s, 2H), 1.25 (s, 6H). ^{13}C NMR (101 MHz, $CDCl_3$) δ ppm 163.62, 162.19, 161.16, 160.25, 160.09, 146.85, 140.69, 138.84, 132.29, 132.26, 129.75, 128.08, 128.00, 126.74, 125.68, 124.40, 121.34, 117.95, 115.98, 115.77, 115.55, 113.56, 112.11, 110.69, 110.28, 110.09, 105.54, 55.39, 54.48, 34.02, 29.71, 25.10. ^{19}F NMR (377 MHz, $CDCl_3$) δ ppm -114.03 (t, *J* = 7.0 Hz). HRMS (ESI+): *m/z* (%) = 502.2246, calculated 502.2176 for $C_{28}H_{29}FN_5O_3$ $[M+H]^+$

32, 1-(4-fluorobenzyl)-N-(6-fluoropyridin-3-yl)-7,7-dimethyl-5-(1H-pyrrole-2-carbonyl)-4,5,6,7-tetrahydro-1H-pyrazolo[4,3-c]pyridine-3-carboxamide, $C_{26}H_{24}F_2N_6O_2$

General procedure A: Yield 51 %. ^{1}H NMR (400 MHz, $CDCl_3$) δ ppm 9.45 (s, NH), 8.64 (s, NH), 8.44 – 8.23 (m, 2H), 7.04 (d, *J* = 7.9 Hz, 4H), 6.94 (m, 2H), 6.85 (s, 1H), 6.31 (s, 1H), 5.44 (s, 2H), 5.23 (s, 2H), 3.76 (s, 2H), 1.35 (s, 6H). ^{13}C NMR (101 MHz, $CDCl_3$) δ ppm 163.65, 161.99, 161.20, 160.30, 147.17, 139.99, 138.54, 138.39, 132.95, 132.88, 132.36, 132.06, 128.07, 127.99, 124.52, 121.21, 116.05, 115.83, 113.24, 110.32, 109.66, 109.28, 54.58, 34.06, 29.72, 25.10, 22.71. ^{19}F NMR (377 MHz, $CDCl_3$) δ ppm -72.77, -113.82.

33, N-(adamantan-1-yl)-1-(4-fluorobenzyl)-7,7-dimethyl-5-(1H-pyrrole-2-carbonyl)-4,5,6,7-tetrahydro-1H-pyrazolo[4,3-c]pyridine-3-carboxamide, $C_{31}H_{36}FN_5O_2$

General procedure A: Yield 64 %. ^{1}H NMR (400 MHz, $CDCl_3$) δ ppm 9.52 (s, NH), 7.07 – 6.94 (m, 4H), 6.92 (s, 1H), 6.88 (s, 1H), 6.59 (s, 1H), 6.29 (q, *J* = 2.9 Hz, 1H), 5.37 (s, 2H), 5.18 (s, 2H), 3.70 (s, 2H), 2.11 (s, 8H), 1.70 (t, *J* = 9.6 Hz, 6H), 1.20 (s, 6H). ^{13}C NMR (101 MHz, $CDCl_3$) δ ppm 163.52, 162.05, 161.42, 161.07, 146.30, 141.64, 132.68, 132.65, 128.00, 127.92, 124.64, 121.04, 115.85, 115.63, 114.87, 113.48, 110.20, 54.26, 51.71, 41.83, 36.39, 33.90, 29.71, 29.50, 25.10. ^{19}F NMR (377 MHz, $CDCl_3$) δ ppm -114.42. HRMS (ESI+): *m/z* (%) = 530.2921, calculated 530.2853 for $C_{31}H_{37}FN_5O_2$ $[M+H]^+$

34, N-cyclohexyl-1-(4-fluorobenzyl)-7,7-dimethyl-5-(1H-pyrrole-2-carbonyl)-4,5,6,7-tetrahydro-1H-pyrazolo[4,3-c]pyridine-3-carboxamide, $C_{27}H_{32}FN_5O_2$

General procedure A: Yield 98 %. ^{1}H NMR (400 MHz, $CDCl_3$) δ ppm 9.52 (s, NH), 7.01 (d, *J* = 8.6 Hz, 4H), 6.89 (d, *J* = 20.5 Hz, 1H), 6.69 (d, *J* = 8.2 Hz, 1H), 6.28 (s, 1H), 5.38 (s, 2H), 5.21 (s, 2H), 3.92 (s, 1H), 3.71 (s, 2H), 1.99 (d, *J* = 12.4 Hz, 2H), 1.83 – 1.53 (m, 4H), 1.34 (d, *J* = 67.4 Hz, 10H). ^{13}C NMR (101 MHz, $CDCl_3$) δ ppm 163.55, 162.10, 161.40, 161.10, 146.29, 140.84, 132.53, 132.50, 128.00, 127.92, 124.37, 121.30, 115.90, 115.69, 115.10, 113.72, 110.37, 54.34, 47.89, 33.91, 33.28, 29.71, 25.57, 25.07, 25.01. ^{19}F NMR (377 MHz, $CDCl_3$) δ ppm -114.31. HRMS (ESI+): *m/z* (%) = 478.2610, calculated 478.2540 for $C_{27}H_{33}FN_5O_2$ $[M+H]^+$

35, (1-(4-fluorobenzyl)-7,7-dimethyl-3-(piperidine-1-carbonyl)-1,4,6,7-tetrahydro-5H-pyrazolo[4,3-c]pyridin-5-yl)(1H-pyrrol-2-yl)methanone, $C_{26}H_{30}FN_5O_2$

General procedure A: Yield 78 %. ^{1}H NMR (400 MHz, $CDCl_3$) δ ppm 9.49 (s, NH), 7.04-6.8 (m, 6H), 6.28 (s, 1H), 5.37 (s, 2H), 5.10 (s, 2H), 3.96 (s, 2H), 3.71 (s, 4H), 1.65 -1.3 (m, 12H). ^{13}C NMR (101 MHz, $CDCl_3$) δ ppm 163.50, 162.31, 162.08, 161.05, 145.05, 141.59, 132.78, 128.31, 128.23, 124.65, 121.05, 116.30, 115.75, 115.54, 113.28, 110.16, 54.23, 48.05, 43.72, 33.78, 29.70, 25.23, 24.75, 14.12. ^{19}F NMR (377 MHz, $CDCl_3$) δ ppm -114.50. HRMS (ESI+): *m/z* (%) = 464.2475, calculated 464.2384 for $C_{26}H_{31}FN_5O_2$ $[M+H]^+$

36, N-cyclopentyl-1-(4-fluorobenzyl)-7,7-dimethyl-5-(1H-pyrrole-2-carbonyl)-4,5,6,7-tetrahydro-1H-pyrazolo[4,3-c]pyridine-3-carboxamide, $C_{26}H_{30}FN_5O_2$

General procedure A: Yield 75%. ^{1}H NMR (400 MHz, $CDCl_3$) δ ppm 9.52 (s, NH), 7.16 – 6.55 (m, 6H), 6.28 (s, 1H), 5.38 (s, 2H), 5.21 (s, 2H), 4.35 (s, 1H), 3.71 (s, 2H), 2.05– 1.25 (m, 14H). ^{13}C NMR (101 MHz, $CDCl_3$) δ ppm 163.54, 162.03, 161.93, 161.09, 146.31, 140.82, 132.58, 132.55, 127.97, 127.89, 124.61, 121.08, 115.89, 115.68, 115.12, 113.47, 110.29, 54.32, 50.66, 33.90, 33.18, 29.71, 25.08, 23.82, 14.13. ^{19}F NMR (377 MHz, $CDCl_3$) δ ppm -114.32. HRMS (ESI+): *m/z* (%) = 464.2454, calculated 464.2384 for $C_{26}H_{31}FN_5O_2$ $[M+H]^+$

37, (1-(4-fluorobenzyl)-7,7-dimethyl-3-(morpholine-4-carbonyl)-1,4,6,7-tetrahydro-5H-pyrazolo[4,3-c]pyridin-5-yl)(1H-pyrrol-2-yl)methanone, $C_{25}H_{28}FN_5O_3$

General procedure A: Yield 91%, ^{1}H NMR (400 MHz, $CDCl_3$) δ ppm 9.54 (s, NH), 7.00 (d, *J* = 6.9 Hz, 4H), 6.93 (d, *J* = 2.9 Hz, 1H), 6.82 (s, 1H), 6.29 (q, *J* = 3.0 Hz, 1H), 5.38 (s, 2H), 5.14 (s, 2H), 4.19 (s, 2H), 3.84 – 3.61 (m, 8H), 1.28 (s, 6H). ^{13}C NMR (101 MHz, $CDCl_3$) δ ppm 163.54, 162.17, 162.04, 161.09, 145.32, 141.06, 132.58, 132.55, 128.23, 128.14, 124.64, 121.11, 117.12, 115.84, 115.62, 113.23, 110.16, 67.23, 67.05, 54.34, 47.50, 33.79, 29.71, 25.20. ^{19}F NMR (377 MHz, $CDCl_3$) δ ppm -114.15 – -114.32 (m). HRMS (ESI+): *m/z* (%) = 466.2256, calculated 466.2176 for $C_{25}H_{29}FN_5O_3$ $[M+H]^+$

38, 1-(4-fluorobenzyl)-N-(2-hydroxyethyl)-7,7-dimethyl-5-(1H-pyrrole-2-carbonyl)-4,5,6,7-tetrahydro-1H-pyrazolo[4,3-c]pyridine-3-carboxamide, $C_{23}H_{26}FN_5O_3$

General procedure A: Yield 88 %. ^{1}H NMR (400 MHz, $CDCl_3$) δ ppm 9.49 (s, 1H), 7.23 (t, *J* = 6.2 Hz, 1H), 7.02 – 6.97 (m, 4H), 6.92 (td, *J* = 2.7, 1.2 Hz, 1H), 6.83 (s, 1H), 6.28 (dt, *J* = 3.8, 2.6 Hz, 1H), 5.37 (s, 2H), 5.18 (s, 2H), 3.79 (q, *J* = 4.4 Hz, 2H), 3.71 (s, 2H), 3.56 (td, *J* = 5.7, 4.4 Hz, 2H), 1.25 (s, 6H). ^{13}C NMR (101 MHz, $CDCl_3$) δ ppm 163.56, 163.32, 162.01, 161.11, 146.39, 140.39, 132.39, 132.36, 128.09, 128.01, 124.58, 121.10, 115.91, 115.69, 115.20, 113.34, 110.28, 62.45, 54.37, 46.32, 46.28, 42.09, 33.91, 29.71, 26.47, 26.39, 25.10. ^{19}F NMR (377 MHz, $CDCl_3$) δ ppm -114.20 (t, *J* = 7.4 Hz).

39, 1-(4-fluorobenzyl)-7,7-dimethyl-N-(2-propoxyethyl)-5-(1H-pyrrole-2-carbonyl)-4,5,6,7-tetrahydro-1H-pyrazolo[4,3-c]pyridine-3-carboxamide, $C_{26}H_{32}FN_5O_3$

General procedure A: Yield 95 %, ^{1}H NMR (400 MHz, $CDCl_3$) δ ppm 9.51 (s, NH), 7.20 – 6.68 (m, 6H), 6.28 (s, 1H), 5.37 (s, 2H), 5.20 (s, 2H), 3.57 (m, 8H), 1.69-1.25 (m, 8H) 0.89 (s, 3H). ^{13}C NMR (101 MHz, $CDCl_3$) δ ppm 163.59, 162.31, 161.14, 146.12, 140.50, 132.33, 128.23, 128.15, 115.89, 115.68, 110.50, 72.81, 69.26, 54.41, 38.82, 33.97, 31.94, 29.71, 25.10, 22.80, 22.70, 14.13, 10.53. ^{19}F NMR (377 MHz, $CDCl_3$) δ ppm -114.29. HRMS (ESI+): *m/z* (%) = 482.2560, calculated 482.2489 for $C_{26}H_{33}FN_5O_3$ $[M+H]^+$

40, 1-(4-fluorobenzyl)-N,7,7-trimethyl-5-(1H-pyrrole-2-carbonyl)-4,5,6,7-tetrahydro-1H-pyrazolo[4,3-c]pyridine-3-carboxamide, $C_{22}H_{24}FN_5O_2$

General procedure A: Yield 88 %. ^{1}H NMR (400 MHz, $CDCl_3$) δ ppm 9.52 (s, NH), 7.07 – 6.96 (m, 4H), 6.92 (s, 1H), 6.86 (s, 1H), 6.78 (d, *J* = 5.7 Hz, 1H), 6.33 – 6.23 (m, 1H), 5.37 (s, 2H), 5.21 (s, 2H), 3.72 (s, 2H), 2.95 (d, *J* = 5.0 Hz, 3H), 1.24 (s, 6H). ^{13}C NMR (75 MHz, $CDCl_3$) δ ppm 163.94, 162.91, 162.01, 160.67, 146.32, 140.66, 132.39, 128.06, 127.96, 124.52, 121.11, 115.91, 115.63, 114.94, 113.42, 110.28, 54.29, 33.88, 29.68, 25.53, 25.10.^{19}F NMR (377 MHz, $CDCl_3$) δ ppm -114.24. HRMS (ESI+): *m/z* (%) = 410.1992, calculated 410.1914 for $C_{22}H_{25}FN_5O_2$ $[M+H]^+$

41, 1-(4-fluorobenzyl)-N,N,7,7-tetramethyl-5-(1H-pyrrole-2-carbonyl)-4,5,6,7-tetrahydro-1H-pyrazolo[4,3-c]pyridine-3-carboxamide, $C_{23}H_{26}FN_5O_2$

General procedure A: Yield 79 %. ^{1}H NMR (400 MHz, $CDCl_3$) δ ppm 9.49 (s, NH), 7.04 – 6.98 (m, 4H), 6.92 (td, *J* = 2.7, 1.2 Hz, 1H), 6.83 (s, 1H), 6.28 (dt, *J* = 3.8, 2.7 Hz, 1H), 5.39 (s, 2H), 5.13 (s, 2H), 3.73 (s, 2H), 3.39 (s, 3H), 3.09 (s, 3H), 1.25 (s, 6H). ^{13}C NMR (101 MHz, $CDCl_3$) δ ppm 163.75, 163.48, 162.01, 161.03, 145.09, 141.60, 132.73, 128.14, 128.05, 124.65, 120.99, 116.51, 115.77, 115.56, 113.27, 110.15, 54.24, 38.97, 36.25, 33.74, 31.91, 31.42, 30.30, 30.18, 29.68, 25.20. ^{19}F NMR (377 MHz, $CDCl_3$) δ ppm -114.47 (t, *J* = 7.4 Hz). HRMS (ESI+): *m/z* (%) = 424.2144, calculated 424.2071 for $C_{23}H_{27}FN_5O_2$ $[M+H]^+$

42, 1-(4-fluorobenzyl)-7,7-dimethyl-5-(1H-pyrrole-2-carbonyl)-4,5,6,7-tetrahydro-1H-pyrazolo[4,3-c]pyridine-3-carboxamide, $C_{21}H_{22}FN_5O_2$

General procedure A: Yield 79 %. ^{1}H NMR (400 MHz, $CDCl_3$) δ ppm 9.58 (s, NH), 7.02 (d, *J* = 6.8 Hz, 4H), 6.93 (s, 1H), 6.83 (s, 1H), 6.68 (s, 2H), 6.34 – 6.26 (m, 1H), 5.39 (s, 2H), 5.18 (s, 2H), 3.73 (s, 2H), 1.24 (s, 6H). ^{13}C NMR (101 MHz, $CDCl_3$) δ ppm 164.28, 163.72, 162.20, 161.26, 146.60, 140.16, 132.46, 128.28, 128.20, 124.68, 121.30, 116.05, 115.83, 115.65, 113.44, 110.34, 54.55, 34.09, 30.46, 29.84, 25.26. ^{19}F NMR (377 MHz, $CDCl_3$) δ ppm -114.17. HRMS (ESI+): *m/z* (%) = 396.1849, calculated 396.1758 for $C_{21}H_{23}FN_5O_2$ $[M+H]^+$

43, 3-(ethoxycarbonyl)-7,7-dimethyl-4,5,6,7-tetrahydro-1H-pyrazolo[4,3-c]pyridin-5-ium chloride, $C_{11}H_{18}ClN_3O_2$

Same procedure as **14**, Quantitative, LCMS $(ESI)^+$ m/e 260.5 $[M+H]^+$.

44, ethyl 7,7-dimethyl-5-(1H-pyrrole-2-carbonyl)-4,5,6,7-tetrahydro-1H-pyrazolo[4,3-c]pyridine-3-carboxylate, $C_{16}H_{20}N_4O_3$

General procedure C: Yield 79 %. ^{1}H NMR (400 MHz, DMSO) δ ppm 13.41 (s, NH), 11.51 (s, NH), 6.89 (td, *J* = 2.7, 1.3 Hz, 1H), 6.61 – 6.51 (m, 1H), 6.14 (dt, *J* = 3.6, 2.4 Hz, 1H), 4.90 (s, 2H), 4.25 (q, *J* = 6.9 Hz, 2H), 3.69 (s, 2H), 1.28 (t, *J* = 7.1 Hz, 3H), 1.24 (s, 6H). ^{13}C NMR (101 MHz, DMSO) δ ppm 162.51, 151.15, 124.80, 124.28, 123.30, 121.78, 115.32, 112.26, 108.89, 14.63.

45, 7,7-dimethyl-5-(1H-pyrrole-2-carbonyl)-4,5,6,7-tetrahydro-1H-pyrazolo[4,3-c]pyridine-3-carboxylic acid, $C_{14}H_{16}N_4O_3$

Same procedure as **16**, Yield 97 %. LCMS (ESI)$^+$ m/e 289.5 [M+H]$^+$.

46, 7,7-dimethyl-5-(1H-pyrrole-2-carbonyl)-N-(m-tolyl)-4,5,6,7-tetrahydro-1H-pyrazolo[4,3-c]pyridine-3-carboxamide, $C_{21}H_{23}N_5O_2$

General procedure A: Yield 61 %. ^{1}H NMR (400 MHz, Acetone) δ ppm 12.51 (s, NH), 10.66 (s, NH), 9.13 (s, NH), 7.73 (d, *J* = 2.2 Hz, 1H), 7.66 (d, *J* = 8.4 Hz, 1H), 7.24 (t, *J* = 7.8 Hz, 1H), 7.02 (td, *J* = 2.7, 1.3 Hz, 1H), 6.94 (ddt, *J* = 7.6, 1.8, 0.9 Hz, 1H), 6.76 (ddd, *J* = 3.8, 2.5, 1.3 Hz, 1H), 6.27 (dt, *J* = 3.7, 2.5 Hz, 1H), 5.15 (s, 2H), 3.83 (s, 2H), 2.36 (s, 3H), 1.40 (s, 6H).

47, 1-(4-fluorobutyl)-7,7-dimethyl-5-(1H-pyrrole-2-carbonyl)-N-(m-tolyl)-4,5,6,7-tetrahydro-1H-pyrazolo[4,3-c]pyridine-3-carboxamide, $C_{25}H_{30}FN_5O_2$

General procedure B: Yield 81 %, ^{1}H NMR (400 MHz, $CDCl_3$) δ ppm 9.88 (s, NH), 8.87 (s, NH), 7.83 (s, 1H), 7.75 (d, *J* = 8.4 Hz, 1H), 7.60 – 7.50 (m, 2H), 7.24 (s, 1H), 7.18 (s, 1H), 6.61 (q, *J* = 2.8 Hz, 1H), 5.53 (s, 2H), 4.84 (dt, *J* = 47.3, 5.7 Hz, 2H), 4.52 (t, *J* = 7.5 Hz, 2H), 4.08 (s, 2H), 2.69 (s, 3H), 2.44 (p, *J* = 7.6 Hz, 2H), 1.60-1.57 (m, 8H), ^{13}C NMR (101 MHz, $CDCl_3$) δ ppm 162.08, 160.22, 146.26, 140.28, 138.98, 137.66, 128.87, 124.92, 124.53, 121.16, 120.39, 116.90, 114.75, 113.43, 110.25, 84.39, 82.74, 50.99, 33.96, 29.71, 27.80, 27.61, 26.65, 26.61, 25.29, 21.54. ^{19}F NMR (377 MHz, $CDCl_3$) δ ppm -218.57 (tt, *J* = 47.2, 26.7 Hz). HRMS (ESI+): *m/z* (%) = 452.2464, calculated 452.5464 for $C_{25}H_{31}FN_5O_2$ [M+H]$^+$

48, 1-(4-fluorobenzoyl)-7,7-dimethyl-5-(1H-pyrrole-2-carbonyl)-N-(m-tolyl)-4,5,6,7-tetrahydro-1H-pyrazolo[4,3-c]pyridine-3-carboxamide, $C_{28}H_{26}FN_5O_3$

p-fluorobenzoyl chloride (15.4 µL, 0.13 mmol, 1.2 eq) was added dropwise to the solution of **46** (40 mg, 0.11 mmol, 1.0 eq), NEt_3 (46 µL, 0.33 mmol, 3.0 eq), DMAP (1.2 mg, 0.01 mmol, 0.1 eq) in DCM (5 mL) at 0°C and stirred at r.t. for overnight. The reaction mixture was diluted with DCM (30 mL), washed with 1 M HCl (2 mL), and saturated with aqueous $NaHCO_3$ (10 mL) and brine (10 mL). The organic phase was dried over $MgSO_4$ and rotary evaporated to get the crude product. The crude product was purified by flash chromatography Hex/EtOAc 5/4 → 5/10. The appropriate fractions were collected and dried to get the white solid **48**, Yield 55 % .^{1}H NMR (400 MHz, $CDCl_3$) δ ppm 9.62 (s, NH), 8.41 (s, NH), 8.15 – 7.95 (m, 2H), 7.45 (s, 1H), 7.41 – 7.35 (m, 1H), 7.32 – 7.21 (m, 3H), 6.99 (m, 2H), 6.91 (s, 1H), 6.35 (q, *J* = 2.9 Hz, 1H), 5.30 (s, 2H), 3.85 (s, 2H), 2.39 (s, 3H), 1.55 (s, 6H). ^{13}C NMR (75 MHz, Acetone) δ ppm 205.50, 205.24, 204.98, 138.80, 138.30, 128.52, 125.13, 124.13, 121.02, 120.10, 116.70, 113.15, 112.06, 108.88, 32.87, 25.12, 20.66. ^{19}F NMR (377 MHz, $CDCl_3$) δ ppm -103.16. HRMS (ESI+): *m/z* (%) = 500,2121, calculated 500.2020 for $C_{28}H_{27}FN_5O_3$ [M+H]$^+$

49, 1-(4-(3-fluoropropoxy)benzyl)-7,7-dimethyl-5-(1H-pyrrole-2-carbonyl)-N-(m-tolyl)-4,5,6,7-tetrahydro-1H-pyrazolo[4,3-c]pyridine-3-carboxamide, $C_{31}H_{34}FN_5O_3$

General procedure B: Yield 37 % (Note: Reaction was not complete even after heating at 60 °C for 12 h and the additional 1 eq of **66**, therefore the reaction was interrupted after 2 days). ^{1}H NMR (400 MHz, $CDCl_3$) δ ppm 9.55 (s, NH), 8.60 (s, NH), 7.51 (s, 1H), 7.43 (d, *J* = 8.3 Hz, 1H), 7.31 (d, *J* = 8.0 Hz, 2H), 7.23 (t,

J = 7.8 Hz, 2H), 7.11 (d, *J* = 7.7 Hz, 1H), 6.97 – 6.91 (m, 1H), 6.91 – 6.83 (m, 1H), 6.74 (d, *J* = 3.6 Hz, 1H), 6.30 (dq, *J* = 5.0, 2.7 Hz, 1H), 5.40 (s, 2H), 5.25 (s, 2H), 4.63 (dt, *J* = 47.0, 5.8 Hz, 2H), 4.08 (t, *J* = 6.1 Hz, 2H), 3.74 (s, 2H), 2.34 (s, 3H), 2.16 (dp, *J* = 26.4, 6.0 Hz, 2H), 1.26 (s, 6H). ^{13}C NMR (76 MHz, $CDCl_3$) δ ppm 162.08, 161.30, 160.23, 158.52, 157.16, 146.71, 140.50, 139.19, 138.96, 137.65, 135.64, 131.46, 129.00, 128.86, 128.76, 127.69, 127.57, 124.94, 124.64, 124.61, 121.15, 121.10, 120.43, 116.94, 115.40, 114.82, 113.42, 112.83, 110.26, 109.95, 108.00, 89.42, 81.73, 79.55, 63.64, 63.57, 54.72, 37.16, 34.59, 34.02, 30.53, 30.26, 29.70, 26.21, 26.12, 25.08, 22.70, 21.52, 14.11. ^{19}F NMR (377 MHz, $CDCl_3$) δ ppm -222.34 (tt, *J* = 47.2, 26.1 Hz). HRMS (ESI+): *m/z* (%) = 544.2706, calculated 544.2646 for $C_{31}H_{35}FN_5O_3$ [M+H]$^+$

50, 1-(4-fluorophenethyl)-7,7-dimethyl-5-(1H-pyrrole-2-carbonyl)-N-(m-tolyl)-4,5,6,7-tetrahydro-1H-pyrazolo[4,3-c]pyridine-3-carboxamide, $C_{29}H_{30}FN_5O_2$

General procedure B: Yield 66 %. ^{1}H NMR (400 MHz, $CDCl_3$) δ ppm 9.80 (s, NH), 8.84 (s, NH), 7.79 (s, 1H), 7.72 (d, *J* = 8.1 Hz, 1H), 7.53 (t, *J* = 4.0 Hz, 2H), 7.37 (dd, *J* = 8.2, 5.3 Hz, 2H), 7.31 – 7.16 (m, 3H), 7.13 (s, 1H), 6.56 (q, *J* = 3.1 Hz, 1H), 5.47 (s, 2H), 4.59 (t, *J* = 7.5 Hz, 2H), 3.98 (s, 2H), 3.51 (t, *J* = 7.5 Hz, 2H), 2.65 (s, 3H), 1.55 (s, 6H). ^{13}C NMR (101 MHz, $CDCl_3$) δ ppm 163.13, 162.05, 160.69, 160.19, 146.61, 140.44, 139.02, 137.66, 133.41, 133.38, 130.35, 130.27, 128.91, 124.98, 124.52, 121.18, 120.37, 116.88, 115.78, 115.57, 114.70, 113.42, 110.25, 85.05, 83.36, 74.89, 74.81, 63.56, 63.52, 52.74, 35.88, 33.88, 29.71, 25.22, 21.55. ^{19}F NMR (377 MHz, $CDCl_3$) δ ppm -115.57. HRMS (ESI+): *m/z* (%) = 500.2457, calculated 500.2384 for $C_{29}H_{31}FN_5O_2$ [M+H]$^+$

51, 1-benzyl-7,7-dimethyl-5-(1H-pyrrole-2-carbonyl)-N-(m-tolyl)-4,5,6,7-tetrahydro-1H-pyrazolo[4,3-c]pyridine-3-carboxamide, $C_{28}H_{29}N_5O_2$

General procedure B: Yield 76 %. ^{1}H NMR (400 MHz, $CDCl_3$) δ ppm 9.54 (s, NH), 8.59 (s, NH), 7.76 – 6.68 (m, 11H), 6.29 (s, 1H), 5.46 (s, 2H), 5.25 (s, 2H), 3.73 (s, 2H), 2.35 (s, 3H), 1.24 (s, 6H). ^{13}C NMR (101 MHz, $CDCl_3$) δ ppm 162.08, 160.17, 146.84, 140.58, 138.94, 137.60, 136.61, 128.85, 127.90, 126.21, 124.93, 121.14, 120.40, 116.90, 115.42, 113.45, 110.22, 55.13, 33.99, 29.68, 25.02, 21.50. HRMS (ESI+): *m/z* (%) = 468.2392, calculated 468.2321 for $C_{28}H_{30}N_5O_2$ [M+H]$^+$

52, 5-(tert-butoxycarbonyl)-1-(4-fluorobenzyl)-7,7-dimethyl-4,5,6,7-tetrahydro-1H-pyrazolo[4,3-c]pyridine-3-carboxylic acid, $C_{21}H_{26}FN_3O_4$

Same procedure as **16**, Yield 96 %, LCMS (ESI)$^+$ m/e 404.5 [M+H]$^+$.

53, tert-butyl 1-(4-fluorobenzyl)-7,7-dimethyl-3-(m-tolylcarbamoyl)-1,4,6,7-tetrahydro-5H-pyrazolo[4,3-c]pyridine-5-carboxylate, $C_{28}H_{33}FN_4O_3$

General procedure A: Yield 79 %. ^{1}H NMR (400 MHz, $CDCl_3$) δ ppm 8.57 (s, NH), 7.53 (s, 1H), 7.41 (d, *J* = 8.0 Hz, 1H), 7.22 (t, *J* = 7.8 Hz, 1H), 7.04 (d, *J* = 7.0 Hz, 4H), 6.93 (d, *J* = 7.5 Hz, 1H), 5.42 (d, *J* = 1.2 Hz, 2H), 4.75 (s, 2H), 3.39 (s, 2H), 2.36 (s, 3H), 1.48 (s, 9H), 1.19 (s, 6H). ^{13}C NMR (75 MHz, $CDCl_3$) δ ppm 163.96, 160.69, 138.92, 137.71, 132.41, 128.80, 128.10, 127.99, 124.81, 120.25, 116.72, 115.93, 115.65, 54.38, 33.71, 28.44, 24.97, 21.50. ^{19}F NMR (282 MHz, $CDCl_3$) δ ppm -114.22.

54, 1-(4-fluorobenzyl)-7,7-dimethyl-3-(m-tolylcarbamoyl)-4,5,6,7-tetrahydro-1H-pyrazolo[4,3-c]pyridin-5-ium chloride, $C_{23}H_{26}ClFN_4O$

Same procedure as **14**, Yield 98 %, LCMS (ESI)$^+$ m/e 429.9 [M+H]$^+$.

55, 1-(4-fluorobenzyl)-5-(furan-2-carbonyl)-7,7-dimethyl-N-(m-tolyl)-4,5,6,7-tetrahydro-1H-pyrazolo[4,3-c]pyridine-3-carboxamide, $C_{28}H_{27}FN_4O_3$

General procedure C: Yield 62 %. ^{1}H NMR (400 MHz, $CDCl_3$) δ ppm 8.57 (s, NH), 7.53 (d, *J* = 17.3 Hz, 2H), 7.41 (d, *J* = 8.0 Hz, 1H), 7.23 (t, *J* = 9.0 Hz, 1H), 7.10 (d, *J* = 3.5 Hz, 1H), 7.04 (d, *J* = 6.8 Hz, 4H), 6.93 (d, *J* = 7.6 Hz, 1H), 6.54 – 6.44 (m, 1H), 5.43 (s, 2H), 5.19 (s, 2H), 3.74 (s, 2H), 2.36 (s, 3H), 1.28 (s, 6H). ^{13}C NMR

(101 MHz, $CDCl_3$) δ ppm 163.59, 161.14, 160.07, 159.79, 138.97, 137.54, 132.26, 132.23, 128.84, 128.09, 128.01, 124.99, 120.35, 116.84, 115.95, 115.73, 115.47, 111.33, 54.43, 34.08, 31.42, 30.30, 30.19, 29.68, 29.65, 25.17, 21.49. ^{19}F NMR (377 MHz, $CDCl_3$) δ ppm -114.06. HRMS (ESI+): *m/z* (%) = 487.2144, calculated 487.2067 for $C_{28}H_{28}FN_4O_3$ $[M+H]^+$

56, 1-(4-fluorobenzyl)-7,7-dimethyl-5-(thiophene-2-carbonyl)-N-(m-tolyl)-4,5,6,7-tetrahydro-1H-pyrazolo[4,3-c]pyridine-3-carboxamide, $C_{28}H_{27}FN_4O_2S$

General procedure C: Yield 52 %. ^1H NMR (400 MHz, $CDCl_3$) δ ppm 8.55 (s, NH), 7.49-7.46 (m, 3H), 7.39 (d, *J* = 8.0 Hz, 1H), 7.22 (t, *J* = 7.8 Hz, 1H), 7.09-7.04 (m, 5H), 6.93 (d, *J* = 7.5 Hz, 1H), 5.43 (s, 2H), 5.15 (s, 2H), 3.74 (s, 2H), 2.35 (s, 3H), 1.28 (s, 6H). ^{13}C NMR (101 MHz, $CDCl_3$) δ ppm 163.98, 163.63, 161.18, 160.04, 146.88, 140.71, 139.00, 137.50, 137.43, 132.24, 132.20, 129.48, 129.36, 128.87, 128.13, 128.05, 127.13, 125.05, 120.37, 116.87, 116.00, 115.78, 115.33, 54.47, 34.19, 29.71, 25.27, 21.52. ^{19}F NMR (377 MHz, $CDCl_3$) δ ppm -114.01. HRMS (ESI+): *m/z* (%) = 503.1913, calculated 503.1839 for $C_{28}H_{28}FN_4O_2S$ $[M+H]^+$

57, 1-(4-fluorobenzyl)-7,7-dimethyl-5-(tetrahydrofuran-2-carbonyl)-N-(m-tolyl)-4,5,6,7-tetrahydro-1H-pyrazolo[4,3-c]pyridine-3-carboxamide, $C_{28}H_{31}FN_4O_3$

General procedure C: Yield 68 %. ^1H NMR (400 MHz, $CDCl_3$) δ ppm 8.58 (s, NH), 7.53 (s, 1H), 7.40 (d, *J* = 8.1 Hz, 1H), 7.23 (t, *J* = 7.9 Hz, 1H), 7.05 (d, *J* = 6.9 Hz, 4H), 6.94 (d, *J* = 7.7 Hz, 1H), 5.41 (s, 2H), 5.05 – 4.73 (m, 3H), 3.94 (dq, *J* = 44.6, 7.4 Hz, 2H), 3.71 – 3.45 (m, 2H), 2.36 (s, 3H), 2.14 – 1.8 (m, 4H), 1.20 (d, *J* = 2.8 Hz, 6H). ^{13}C NMR (101 MHz, $CDCl_3$) δ ppm 171.78, 163.62, 161.17, 160.18, 147.19, 140.58, 139.02, 137.51, 132.17, 128.87, 128.17, 125.07, 120.33, 116.81, 115.98, 115.76, 115.15, 75.81, 69.27, 54.45, 52.91, 42.89, 33.94, 29.20, 26.93, 25.68, 25.18, 21.53. ^{19}F NMR (377 MHz, $CDCl_3$) δ ppm -114.01. HRMS (ESI+): *m/z* (%) = 491.2458, calculated 491.2380 for $C_{28}H_{32}FN_4O_3$ $[M+H]^+$

58, 1-(4-fluorobenzyl)-5-(1H-imidazole-4-carbonyl)-7,7-dimethyl-N-(m-tolyl)-4,5,6,7-tetrahydro-1H-pyrazolo[4,3-c]pyridine-3-carboxamide, $C_{27}H_{27}FN_6O_2$

General procedure C: Yield 51 %. ^1H NMR (400 MHz, $CDCl_3$) δ ppm 8.58 (s, NH), 7.76 – 7.44 (m, 3H), 7.41 (d, *J* = 8.1 Hz, 1H), 7.22 (t, *J* = 7.8 Hz, 1H), 7.04 (d, *J* = 6.9 Hz, 4H), 6.94 (d, *J* = 7.5 Hz, 1H), 5.42 (s, 2H), 3.74 (s, 2H), 3.16 (td, *J* = 6.7, 3.6 Hz, 2H), 2.35 (s, 3H), 1.25 (s, 6H). ^{13}C NMR (101 MHz, $CDCl_3$) δ ppm 163.58, 161.13, 160.17, 138.99, 132.27, 128.87, 128.10, 128.01, 125.11, 115.93, 115.71, 54.42, 46.34, 34.09, 25.15, 21.48. ^{19}F NMR (376 MHz, $CDCl_3$) δ ppm -114.11. HRMS (ESI+): *m/z* (%) = 487.2254, calculated 487.2180 for $C_{27}H_{28}FN_6O_2$ $[M+H]^+$

59, 1-(4-fluorobenzyl)-5-(1H-indazole-3-carbonyl)-7,7-dimethyl-N-(m-tolyl)-4,5,6,7-tetrahydro-1H-pyrazolo[4,3-c]pyridine-3-carboxamide, $C_{31}H_{29}FN_6O_2$

General procedure C: Yield 54 %. ^1H NMR (400 MHz, $CDCl_3$) δ ppm 11.68 (s, NH), 8.81 (s, NH), 7.86 – 6.99 (m, H), 6.23 (s, 11H), 5.66 (s, 2H), 5.47 (s, 2H), 5.06 (s, 2H), 2.60 (s, 3H), 1.38 (s, 6H). ^{13}C NMR (101 MHz, $CDCl_3$) δ ppm 163.57, 161.11, 159.99, 159.22, 146.42, 145.24, 144.85, 141.29, 138.94, 137.69, 132.28, 128.82, 127.98, 124.84, 120.22, 116.66, 115.93, 115.71, 114.96, 55.77, 54.41, 54.14, 41.70, 34.38, 25.24, 25.00, 21.52. ^{19}F NMR (376 MHz, $CDCl_3$) δ ppm -114.12. HRMS (ESI+): *m/z* (%) = 534.2411, calculated 537.2336 for $C_{31}H_{30}FN_6O_2$ $[M+H]^+$

60, 5-(3-fluoro-2,2-dimethylpropanoyl)-1-(4-fluorobenzyl)-7,7-dimethyl-N-(m-tolyl)-4,5,6,7-tetrahydro-1H-pyrazolo[4,3-c]pyridine-3-carboxamide, $C_{28}H_{32}F_2N_4O_2$

3-fluoro-2,2-dimethylpropanoyl chloride was added to the solution of **54**, NEt_3, DMAP in DCM at 0°C and afterwards, stirred overnight at r.t.. The reaction mixture was diluted with DCM, washed with 1 M HCl (2 mL), and saturated with aqueous $NaHCO_3$ (10 mL) and brine (10 mL). The organic phase was dried over $MgSO_4$ and rotary evaporated to get the crude product. The crude product was purified by flash

chromatography Hex/EtOAc 4/1→ 4/7. The appropriate fractions were collected and dried to get the white solid **60**, Yield 58 %. ^{1}H NMR (400 MHz, $CDCl_3$) δ ppm 8.58 (s, NH), 7.50 (s, 1H), 7.42 (d, *J* = 7.9 Hz, 1H), 7.22 (d, *J* = 7.8 Hz, 1H), 7.05 (d, *J* = 6.8 Hz, 4H), 6.94 (d, *J* = 7.5 Hz, 1H), 5.41 (s, 2H), 5.00 (s, 2H), 4.47 (d, *J* = 48.0 Hz, 2H), 3.60 (s, 2H), 2.37 (s, 3H), 1.39 (s, 6H), 1.21 (s, 6H). ^{13}C NMR (101 MHz, $CDCl_3$) δ ppm 174.39, 163.60, 161.14, 160.16, 147.49, 140.45, 139.01, 137.42, 132.14, 132.11, 128.89, 128.14, 128.06, 125.11, 120.42, 116.93, 115.95, 115.73, 115.24, 90.17, 88.44, 54.33, 54.10, 44.04, 43.06, 42.88, 34.23, 29.68, 25.28, 21.49, 21.42, 21.37. ^{19}F NMR (376 MHz, $CDCl_3$) δ ppm -114.05 (p, *J* = 7.0 Hz), -224.26, -224.45 (d, *J* = 47.9 Hz). HRMS (ESI+): *m/z* (%) = 495.2457, calculated 495.2493 for $C_{28}H_{33}F_2N_4O_2$ [M+H]$^+$

61, 5-acetyl-1-(4-fluorobenzyl)-7,7-dimethyl-N-(m-tolyl)-4,5,6,7-tetrahydro-1H-pyrazolo[4,3-c]pyridine-3-carboxamide, $C_{25}H_{27}FN_4O_2$

General procedure C: Yield 90 %. ^{1}H NMR (400 MHz, $CDCl_3$) δ ppm 8.62 (s, NH), 7.75 – 6.74 (m, 8H), 5.45 (s, 2H), 4.83 (s, 2H), 3.60 (s, 2H), 2.39-2.25 (m, 6H), 1.28 (s, 6H). ^{13}C NMR (101 MHz, $CDCl_3$) δ ppm 170.25, 163.61, 161.15, 160.20, 147.23, 140.52, 139.01, 137.51, 132.15, 128.85, 128.14, 128.06, 125.05, 124.78, 120.27, 116.74, 115.96, 115.74, 115.28, 54.46, 52.30, 43.91, 33.78, 31.42, 30.30, 30.19, 29.68, 25.06, 21.88, 21.50. ^{19}F NMR (377 MHz, $CDCl_3$) δ ppm -113.98. HRMS (ESI+): *m/z* (%) = 435.2206, calculated 435.2118 for $C_{25}H_{28}FN_4O_2$ [M+H]$^+$

62, 1-(4-fluorobenzyl)-7,7-dimethyl-5-propionyl-N-(m-tolyl)-4,5,6,7-tetrahydro-1H-pyrazolo[4,3-c]pyridine-3-carboxamide, $C_{26}H_{29}FN_4O_2$

Same procedure as **60**. The crude product was purified by flash chromatography Hex/EtOAc 4/8 → 4/12. The appropriate fractions were collected and dried to get the white solid **62**, Yield 45 %. ^{1}H NMR (400 MHz, $CDCl_3$) δ ppm 8.58 (s, 1H), 7.54 (s, 1H), 7.40 (d, *J* = 8.1 Hz, 1H), 7.22 (d, *J* = 7.8 Hz, 1H), 7.05 (d, *J* = 6.9 Hz, 5H), 6.94 (d, *J* = 7.8 Hz, 1H), 5.42 (s, 2H), 4.80 (s, 2H), 3.59 (s, 2H), 2.50 (q, *J* = 7.4 Hz, 2H), 2.36 (s, 3H), 1.25 (s, 4H), 1.16 (d, *J* = 7.4 Hz, 4H). ^{13}C NMR (101 MHz, $CDCl_3$) δ ppm 173.50, 163.60, 161.14, 160.22, 147.34, 140.51, 139.00, 137.53, 132.17, 128.85, 128.05, 125.02, 120.25, 116.72, 115.95, 115.73, 115.38, 54.43, 52.50, 43.01, 33.79, 29.68, 26.94, 25.10, 21.50, 9.40.^{19}F NMR (376 MHz, $CDCl_3$) δ ppm -114.04 (p, *J* = 7.1 Hz). HRMS (ESI+): *m/z* (%) = 449.2368, calculated 449.2275 for $C_{26}H_{30}FN_4O_2$ [M+H]$^+$

63, 5-ethyl-1-(4-fluorobenzyl)-7,7-dimethyl-N-(m-tolyl)-4,5,6,7-tetrahydro-1H-pyrazolo[4,3-c]pyridine-3-carboxamide, $C_{25}H_{29}FN_4O$

General procedure B: Yield 30 %. ^{1}H NMR (400 MHz, $CDCl_3$) δ ppm 8.57 (s, NH), 7.57 (s, 1H), 7.39 (dd, *J* = 8.0, 2.2 Hz, 1H), 7.20 (t, *J* = 7.8 Hz, 1H), 7.02 (d, *J* = 6.9 Hz,4H), 6.91 (d, *J* = 7.5 Hz, 1H), 5.41 (d, *J* = 1.2 Hz, 2H), 3.79 (s, 2H), 2.70 – 2.56 (m, 2H), 2.39 (s, 2H), 2.35 (s, 3H), 1.24 (s, 6H), 1.16 (t, *J* = 7.1 Hz, 3H). ^{13}C NMR (75 MHz, $CDCl_3$) δ ppm 160.46, 147.12, 140.77, 138.86, 137.90, 132.73, 130.86, 128.72, 128.06, 127.95, 125.02, 124.61, 120.15, 117.96, 116.56, 115.81, 115.52, 68.15, 65.74, 54.03, 51.36, 50.31, 33.35, 29.69, 26.23. ^{19}F NMR (282 MHz, $CDCl_3$) δ ppm -114.58 – -114.74 (m). HRMS (ESI+): *m/z* (%) = 421.2395, calculated 421.2325 for $C_{25}H_{30}FN_4O$ [M+H]$^+$

64, 7,7-dimethyl-5-(1H-pyrrole-2-carbonyl)-1-(4-(4,4,5,5-tetramethyl-1,3,2-dioxaborolan-2-yl)benzyl)-N-(m-tolyl)-4,5,6,7-tetrahydro-1H-pyrazolo[4,3-c]pyridine-3-carboxamid, $C_{34}H_{40}BN_5O_4$

General procedure A: Yield 48 %. ^{1}H NMR (400 MHz, $CDCl_3$) δ ppm 9.47 (s, NH), 8.59 (s, NH), 7.77 (d, *J* = 8.0 Hz, 2H), 7.50 (s, 1H), 7.43 (dd, *J* = 8.0, 2.2 Hz, 1H), 7.23 (t, *J* = 7.8 Hz, 1H), 7.03 (d, *J* = 7.8 Hz, 2H), 6.96 – 6.90 (m, 2H), 6.88 (s, 1H), 6.30 (q, *J* = 3.0 Hz, 1H), 5.48 (s, 2H), 5.26 (s, 2H), 3.74 (s, 2H), 2.36 (s, 3H), 1.33 (s, 12H), 1.24 (s, 6H). ^{13}C NMR (101 MHz, $CDCl_3$) δ ppm 162.03, 160.20, 140.67, 139.63, 138.96, 137.62, 135.30, 128.86, 125.55, 124.95, 124.62, 121.05, 120.42, 116.93, 115.41, 113.39, 110.30, 83.95, 75.05,

55.23, 34.00, 29.71, 25.09, 24.87, 21.53. ^{11}B NMR (128 MHz, $CDCl_3$) δ ppm 31.81. HRMS (ESI+): *m/z* (%) = 594.3248, calculated 594.3173 for $C_{34}H_{41}BN_5O_4$ $[M+H]^+$

65, 1-(3-fluoropropoxy)-4-methylbenzene, $C_{10}H_{13}FO$

To a solution of *p*-cresol (200 mg, 1.9 mmol, 1 eq) in anhydrous DMF was added K_2CO_3 (1050 mg, 7.6 mmol, 4 eq) and stirred at r.t. for 1 h. Afterwards, 3-fluoropropyl iodide (360 µL, 3.7 mmol, 2 eq) was added and stirred overnight at 50 °C. The reaction mixture was rotary evaporated to remove anhydrous DMF and then extracted with EtOAc (30 mL x 3) and water (10 mL). The organic phase was washed with brine (10 mL) and then dried over the $MgSO_4$. The crude reaction mixture was then purified by flash chromatography Hex/EtOAc → 8/1 × 3 (100 mL). The appropriate fraction was collected to get **65**, yield 51 %. ^1H NMR (400 MHz, $CDCl_3$) δ ppm 7.08 (dt, *J* = 8.1, 0.7 Hz, 2H), 6.86 – 6.77 (m, 2H), 4.65 (dt, *J* = 47.1, 5.8 Hz, 2H), 4.07 (t, *J* = 6.1 Hz, 2H), 2.29 (s, 3H), 2.16 (dp, *J* = 25.9, 6.0 Hz, 2H). ^{19}F NMR (377 MHz, $CDCl_3$) δ ppm -222.14 (tt, *J* = 47.1, 25.9 Hz).

66, 1-(bromomethyl)-4-(3-fluoropropoxy)benzene, $C_{10}H_{12}BrFO$

A solution of **65** in CCl_4 was treated with *N*-Bromo succinimide (NBS) (238 mg, 1.4 mmol, 1.5 eq) and Azobisisobutyronitrile (AIBN) (26.6 mg, 0.36 mmol, 0.4 eq) for 7 h at 80 °C. The reaction mixture was filtered, and the supernatant was rotary evaporated. Then it was washed with EtOAc (30 mL x 3) and water (10 mL). The combined organic phase was washed with brine (10 mL) and then dried over anhydrous $MgSO_4$. It was filtered and dried to get the crude product. The crude product was purified by flash chromatography Hex/EtOAc 10/1 → 7/1 (100 mL) to obtain the product **66**, Yield 55 %. ^1H NMR (400 MHz, $CDCl_3$) δ ppm 9.89 (s, 1H), 7.90 – 7.75 (m, 2H), 7.06 – 6.96 (m, 2H), 4.66 (dt, *J* = 47.0, 5.7 Hz, 2H), 4.19 (t, *J* = 6.1 Hz, 2H), 3.11 – 2.72 (m, 1H), 2.21 (dp, *J* = 26.2, 5.9 Hz, 2H). ^{13}C NMR (75 MHz, $CDCl_3$) δ ppm 190.77, 163.77, 132.00, 130.08, 114.74, 81.46, 79.28, 63.92, 63.85, 31.71, 31.42, 30.36, 30.18, 30.09, 29.68, 29.57, 28.45, 25.16. ^{19}F NMR (377 MHz, $CDCl_3$) δ ppm -222.62 (tt, *J* = 47.2, 26.2 Hz).

S2: X-ray Crystallography

X-ray crystallography studies were performed by Dr. Peter Lönnecke at the University of Leipzig. The crystals of **SK60** (25 mg) were grown in MeCN at r.t. over 4-5 weeks. The data were collected on a Gemini diffractometer (Rigaku Oxford Diffraction) using Mo-Kα radiation and ω-scan rotation. Data reduction was performed with CrysAlisPro (Rigaku Oxford Diffraction, (1995-2023), CrysAlisPro Software system, Rigaku Corporation, Wroclaw, Poland) including the program SCALE3 ABSPACK for empirical absorption correction. The structure was solved by dual space methods with SHELXT[1] and the refinement was performed with SHELXL[2]. All non-hydrogen atoms were refined with anisotropic displacement parameters. Hydrogen atoms were calculated on idealized positions using the riding model. Structure figures were generated with DIAMOND-4 (DIAMOND 4; K. Brandenburg, Crystal Impact GbR, Bonn, Germany). The CCDC deposition number given in Table S1 contains the supplementary crystallographic data for this paper. The data can be obtained free of charge via https://www.ccdc.cam.ac.uk/structures/ (or from the Cambridge Crystallographic Data Centre, 12 Union Road, Cambridge CB2 1EZ, UK; fax: (+44)1223-336-033; or deposit@ccdc.cam.uk).

*Table S1: Crystal data and structure refinement for **SK60**.*

Empirical formula	$C_{28}H_{28}FN_5O_2$	
Formula weight	485.55	
Temperature	299(2) K	
Wavelength	71.073 pm	

Crystal system	Monoclinic	
Space group	P $2_1/c$	
Unit cell dimensions	a = 1019.49(3) pm	α = 90°.
	b = 1520.46(4) pm	β = 90.854(2)°.
	c = 1648.39(4) pm	γ = 90°.
Volume	2.5549(1) nm^3	
Z	4	
Density (calculated)	1.262 Mg/m^3	
Absorption coefficient	0.087 mm^{-1}	
F(000)	1024	
Crystal size	0.199 x 0.150 x 0.052 mm^3	
Theta range for data collection	2.471 to 28.414°.	
Index ranges	-13 ≤ h ≤ 13<br>-19 ≤ k ≤ 19<br>-21 ≤ l ≤ 21	
Reflections collected	43870	
Independent reflections	5828 [R(int) = 0.0578]	
Completeness to theta = 25.350°	99.9 %	
Absorption correction	Semi-empirical from equivalents	
Max. and min. transmission	1.00000 and 0.99925	
Refinement method	Full-matrix least-squares on F^2	
Restraints/parameters	0 / 328	
Goodness-of-fit on F^2	1.001	
Final R indices [I>2sigma(I)]	R1 = 0.0614, wR2 = 0.1293	
R indices (all data)	R1 = 0.1551, wR2 = 0.1656	
Residual electron density	0.140 and -0.173 e·Å^{-3}	
CCDC Number	2405785	

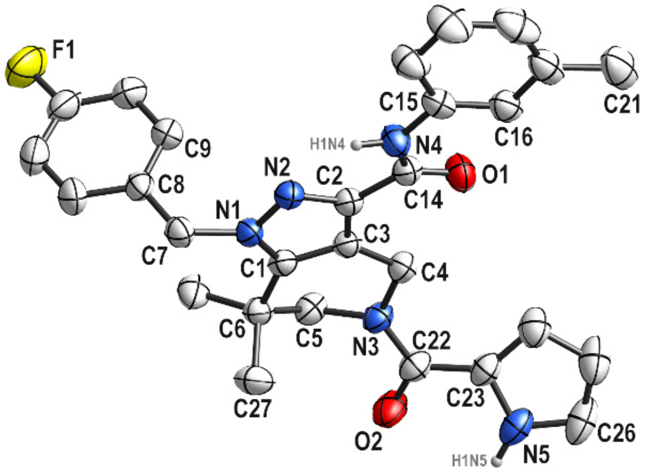

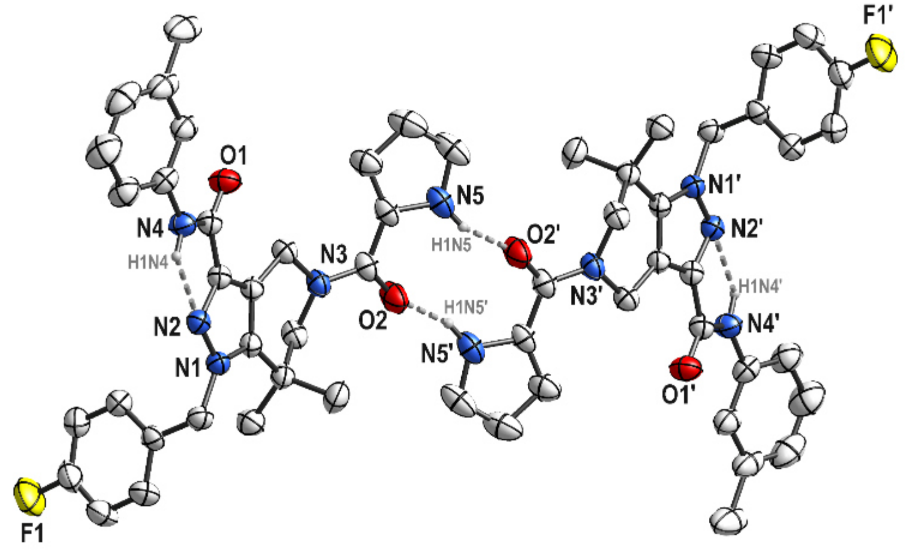

*Figure S1: Molecular structure and labeling scheme of **SK60** (top). For H1N4 an intramolecular contact to N2 is detectable (N4-N2: 271.9(3) and H1N4-N2: 229 pm). With intermolecular NH···O hydrogen donor-acceptor bonds (N5-O2': 277.2(3) and H1N5-O2': 199 pm) dimers are formed (bottom). Hydrogen atoms, except NH, were omitted for clarity, and displacement ellipsoids were drawn at the 30% probability level. Symmetry operator ('): -x+1,-y+2,-z+1.*

S3: The *in vitro* and *in vivo* studies

Inhibitory potency determination

The inhibitory potential of **SK60** for the IDH1 and IDH1R123H recombinant enzymes was evaluated. The IC_{50} values obtained were 472 ± 180 nM and 14.5 ± 3.3 nM for IDH1 and IDH1R132H, respectively.

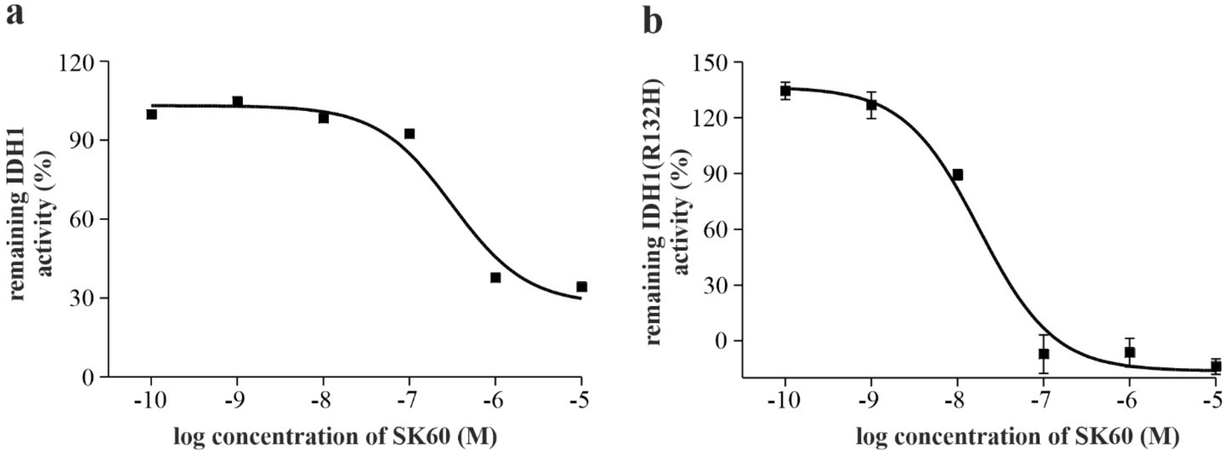

*Figure S2: Representative **SK60** inhibition curves for (**a**) IDH1 and (**b**) IDH1R123H. Recombinant IDH1 and IDH1R132H proteins were preincubated with different concentrations (10^{-5} to 10^{-10}) of **SK60**. One hour after the addition of the substrate the conversion of resazurin to resorufin by diaphorase was measured fluorometrically at Ex544/Em590. The IC_{50} values were calculated with a standard dose−response curve fitting: All experiments are performed in triplicates (n = 3).*

Real-time radioligand binding

Methodology

Real-time radioligand binding using LigandTracer®

The LigandTracer (LigandTracer Yellow, Ridgeview Instruments AB, Uppsala, Sweden) system allows assessment of binding kinetics (association rate constant k_a and dissociation rate constant k_d) and therefore dissociation constant (K_D).

Briefly, target-positive/-negative cells are seeded to different quadrants of a petri dish (Thermo Scientific Nunclon Delta #150350). Here, approximately $5x10^4$ target-positive cells (IDH1R132H-U251 cells) and target-negative cells (IDH1-U251 cells) were seeded in a ~20 mm round spot (0.5 mL of a solution of $1x10^5$ cells/mL), three days before the experiment.

For the determination of kinetic parameters, cultured cells in a petri dish are placed in the instrument. The culture medium is removed and replaced with 3 mL CO_2-independent medium (Gibco #18045088, ThermoFisher Germany) without or with bovine serum albumin (BSA) (5 %; #3737.3, Carl Roth GmbH, Karlsruhe, Germany). In the instrument, the dish rotates on an inclined base, where cells in medium (3 mL) are located on one side, while radioactivity is detected on the opposite side. This allows continuous measurements of two or more alternating parts of the dish for bound radioactivity (e.g. target-positive) against background (e.g. no cells) or target-negative cells.

All experiments were performed at room temperature. Association of **[^{18}F]SK60** was observed following incubation with two or more increasing concentrations of the tracer. This was followed by replacement with fresh medium (3 mL) and observation of dissociation for at least 90 min. Previously, this approach was found to yield reliable kinetic measurements when a 1:1 interaction is likely.[3] After the experiment, petri dishes were inspected for cell detachment under a microscope.

Data was acquired in decay-corrected counts per second (CPS) and subsequently analyzed using TraceDrawer (1.9.2, Ridgeview Instruments AB, Uppsala, Sweden). For that purpose, traces were loaded into TraceDrawer and inspected for potential spikes (>100 % sudden increases in CPS over the previous data point). Typically, data would then be fitted to yield kinetic parameters.

Results

Reliable binding of **[^{18}F]SK60** could not be observed. Real-time radioligand binding to target-positive (IDH1R132H-U251) and negative (IDH1-U251) live cells indicated a lack of substantial specific binding of **[^{18}F]SK60**. Using a CO_2-independent medium without additives (Figure S3, A), only a marginal difference in bound tracer over time was found for target-positive and negative cells. This indicates only little binding at the employed tracer concentrations, hinting towards a lack of specific binding and/or low affinity. More importantly, radioactivity in the background quadrant (polystyrene of the petri dish) increased in a pattern consistent with that exhibited by cells, albeit at lower levels. This indicates a pronounced nonspecific binding component of the tracer, possibly caused by its rather lipophilic nature. Bovine serum albumin (BSA) is often used to prevent such nonspecific binding. Adding 5 % (*w/v*) BSA to the incubation medium indeed effectively reduced nonspecific binding, especially in the lower concentration range (Figure S3, B). However, this also resulted in a nearly identical binding pattern exhibited by both target-positive and negative cells. The latter again supports the conclusion of little to no specific binding to the target.

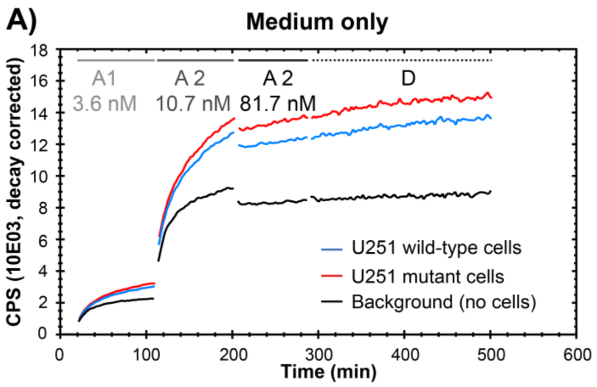

*Figure S3: Real-time binding of escalating concentrations of **[¹⁸F]SK60** to U251 wild-type/mutant cells in the absence/presence of bovine serum albumin. Curves show radioactivity (CPS, counts per second) in quadrants of a petri dish containing U251 wild-type (blue) or mutant (red) cells and the background (black, no cells). Association phases (A) with corresponding tracer concentrations are indicated, along with the dissociation phase (D).*

Radioligand binding to lysates of IDH1-U251 and IDH1R132-U251 cells

Methodology

Radioligand binding using the cytosolic fraction of stably transfected cells

IDH1 is mainly located in the cytoplasm. Therefore, the cytosolic fraction of IDH1-U251 and IDH1R132H-U251 cells was used to determine the affinity of **[^{18}F]SK60** for the respective enzyme. First, whole cell lysates of IDH1-U251 and IDH1R132H-U251 cells were prepared by scraping the cells from the cell culture flask with PBS, centrifuging the resulting suspension (800 rpm, 3 min, r.t.), resuspending the pellet in hypotonic buffer (50 mM TRIS-HCl, pH 7.4 at 4°C) and storing the suspensions at -30°C. The suspensions were thawed on ice, homogenized by two passes through a grey 27G cannula, and centrifuged at 15,000 rpm for 15 min at 4°C. The protein concentration of the supernatant, containing the cytosolic fraction of the cells and used in the radioligand binding experiment, was determined by a BCA assay according to the supplier's protocol (Pierce™ BCA Protein Assay; # 23225, Thermo Fisher Scientific Inc.). The supernatants of the IDH1-U251 and IDH1R132H-U251 cells were diluted 1:10 with incubation buffer (PBS without Ca^{2+} and Mg^{2+}(#9143.1, Carl Roth GmbH, Karlsruhe, Germany) supplemented with 10 mM $MgCl_2$). To 800 µl of the resulting suspension, 100 µl of **[^{18}F]SK60** (1.21 MBq/mL incubation buffer with a molar activity of 77 MBq/mL at the start of the experiment; resulting in a final chemical concentration of 1.6 nM) and 100 µl of incubation buffer (to determine total binding) or 10^{-4} M solutions of mIDH inhibitors (pan-inhibitor **BAY-1436032**, mIDH1 inhibitor **AG-120**, here developed **SK60**) were added. To determine the filter binding of **[^{18}F]SK60**, to 100 µl of ligand 900 µl of only incubation buffer was added. Suspensions were incubated in glass vials on a shaker (300 rpm) at room temperature. Incubation was stopped after 120 min by filtration through a glass fiber filter (GF75; Hahnemühle FineArt GmbH, Dassel, Germany), using a cell harvester (Brandel M-48 Cell Harvester; Brandel Inc., Gaithersburg, Maryland, USA) followed by three 1 mL wash steps with refrigerated washing buffer (50 mM TRIS-HCl, pH 7.4 at 4°C). The filter discs were excised, and the filter-bound radioactivity was measured along with 10 µl aliquots of the original radiotracer solution as calibration standards in an automated gamma counter (WIZARD 2470; Revvity Germany Diagnostics GmbH, Lübeck, Germany).

Results

Specific binding of **[^{18}F]SK60** to IDH1R132H could not be observed. A comparison of the total filter-bound activity obtained with the cytosolic fractions of IDH1-U251 or IDH1R132H-U251 cells revealed no higher

signal for the latter one (Figure S4). In addition, neither the co-incubation of 1.6 nM **[^{18}F]SK60** with high concentrations (10 µM) of the established mIDH inhibitors **BAY-1436032** and **AG-120** nor with **SK60** itself reduced the amount of activity bound to the IDH1-containing supernatants of either IDH1-U251 or IDH1R132H-U251 cells. Altogether, the results confirm a lack of substantial specific binding of **[^{18}F]SK60**.
In addition, the activity concentrations of aliquots taken from the original radioligand solution (stored in a 50 mL plastic tube) at the time of the start of the experiment and 2 h later differed significantly (118222 CPM/10 µl and 50749 CPM/10 µl, resp.) indicating binding of **[^{18}F]SK60** to plastic. The binding of **[^{18}F]SK60** to the glass fiber filter accounted for about 30 % of the originally applied dose.

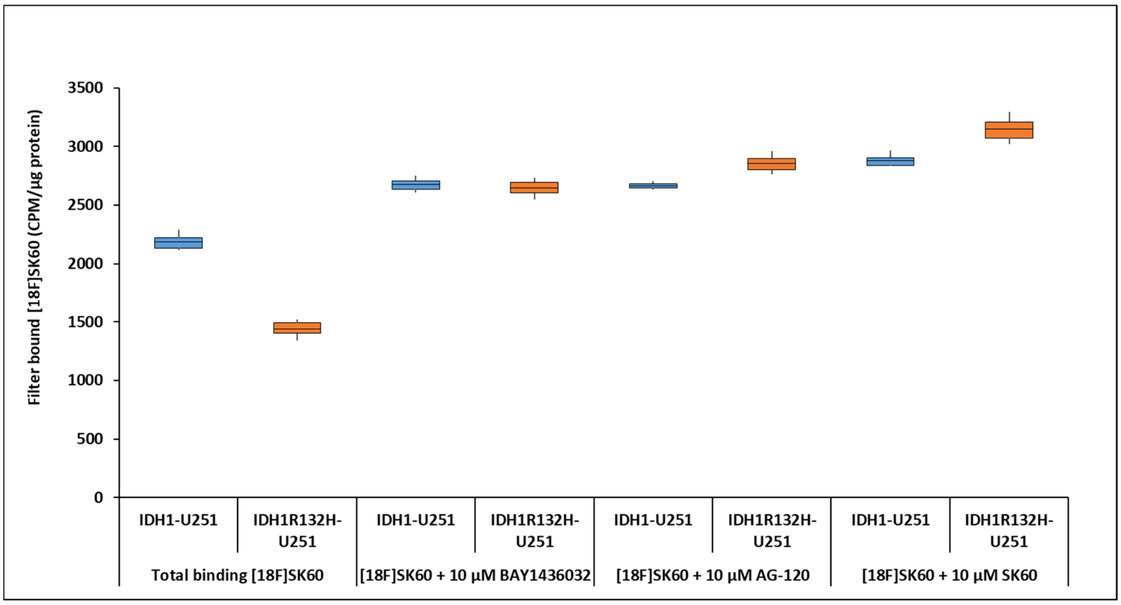

*Figure S4: Binding of **[^{18}F]SK60** to the cytosolic fractions of whole cell lysates from IDH1-U251 (blue) and IDH1R132H-U251 (orange) cells in the absence (total binding) or presence of mIDH inhibitors (**BAY-1436032**, **AG-120**, **SK60**). Data show radioactivity bound to the filter after separation of free and protein-bound **[^{18}F]SK60** by filtration, normalized to microgram protein (CPM/µg protein). Data were obtained in a single experiment performed in technical triplicate.*

PET Biodistribution

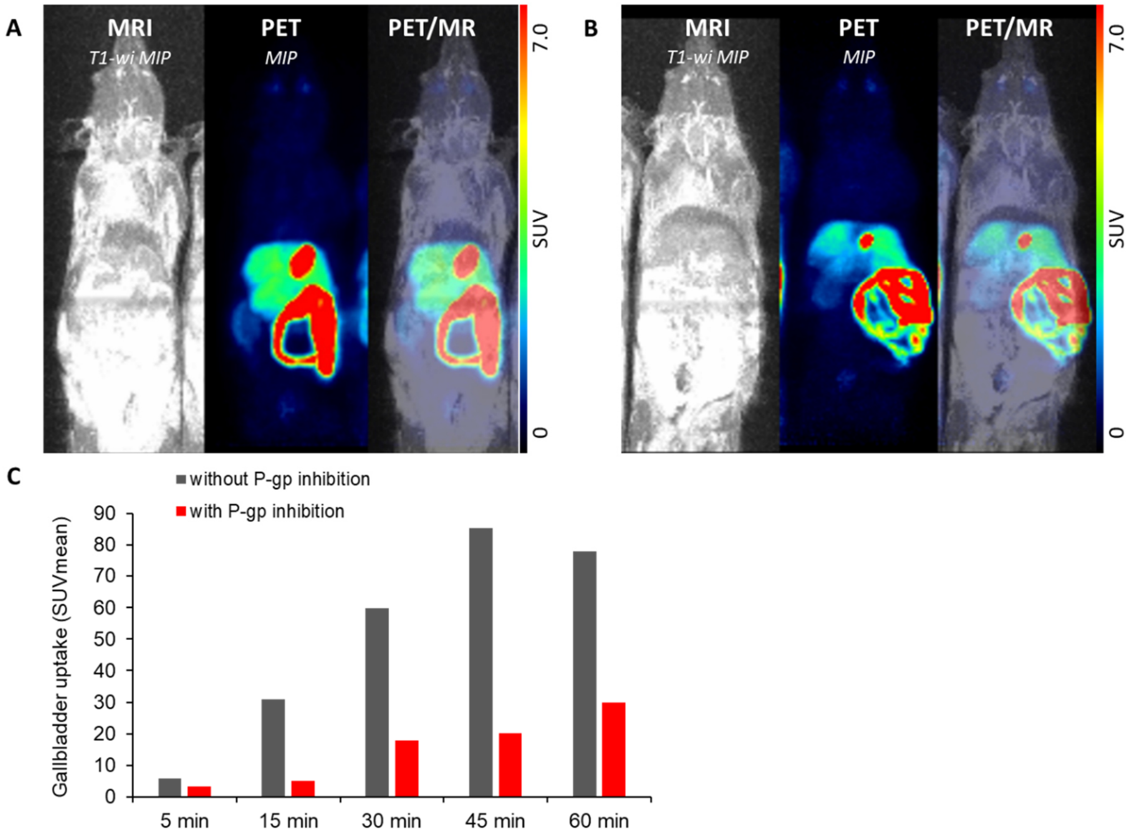

*Figure S5: A) Representative images of **[^{18}F]SK60** in naive female CD-1 mouse, **A** after pretreatment with either vehicle or **B** cyclosporine A (Sandimmune®, 50 mg/kg) revealed hepato-biliary excretion pathway. **C [^{18}F]SK60** gallbladder uptake without P-gp inhibition (vehicle, black histogram) or upon P-gp inhibition (Cyclosporine A, red histogram) (MRI: T1-weighted image; PET: maximal intensity projection from 0-60 minutes; PET/MRI: fusion of both modalities). Gb: Gallbladder; L: Liver; sI: small intestine.*

S4: Separation of stereoisomers of 9

*Table S2: Different HPLC conditions were tested to separate all four stereoisomers of **9** in stereoisomeric mixture I.*

Column	Mobile phase compositions (isocratic mode)[b]
ChiralPak IA[a]	-MeCN/20 mM NH_4OAc_{aq}<br>-*n*-hexane/*i*-PrOH
ChiralPak IB[a]	-*n*-hexane/*i*-PrOH<br>-*n*-hexane/EtOH<br>-*n*-hexane/THF<br>-*n*-hexane/EtOH/DCM
[a]: 250*4.6 mm, 5 µm particle size, b: Flow 1 mL/min	

UV and CD chromatogram of stereoisomeric mixture I

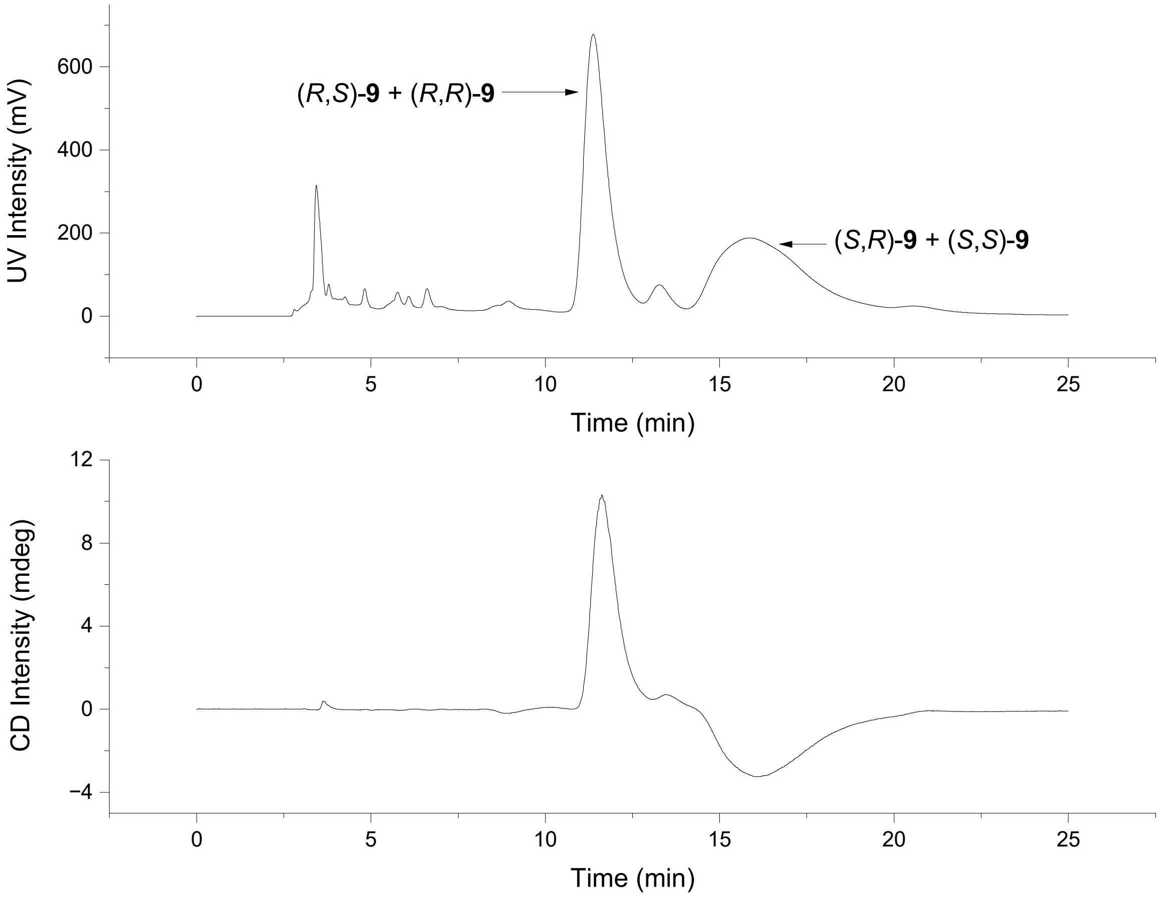

*Figure S6: Chromatograms of the chiral HPLC separation of stereoisomeric mixture **I** using CHIRALPAK®IA (250 x 4.6 mm) with 62 % of MeCN in aqueous 20 mM NH_4OAc at a flow rate of 1 mL/min with CD detection at 268 nm.*

UV and CD chromatograms of (*R*,*S*)-9, (*S*,*S*)-9, (*R*,*R*)-9 and (*S*,*R*)-9

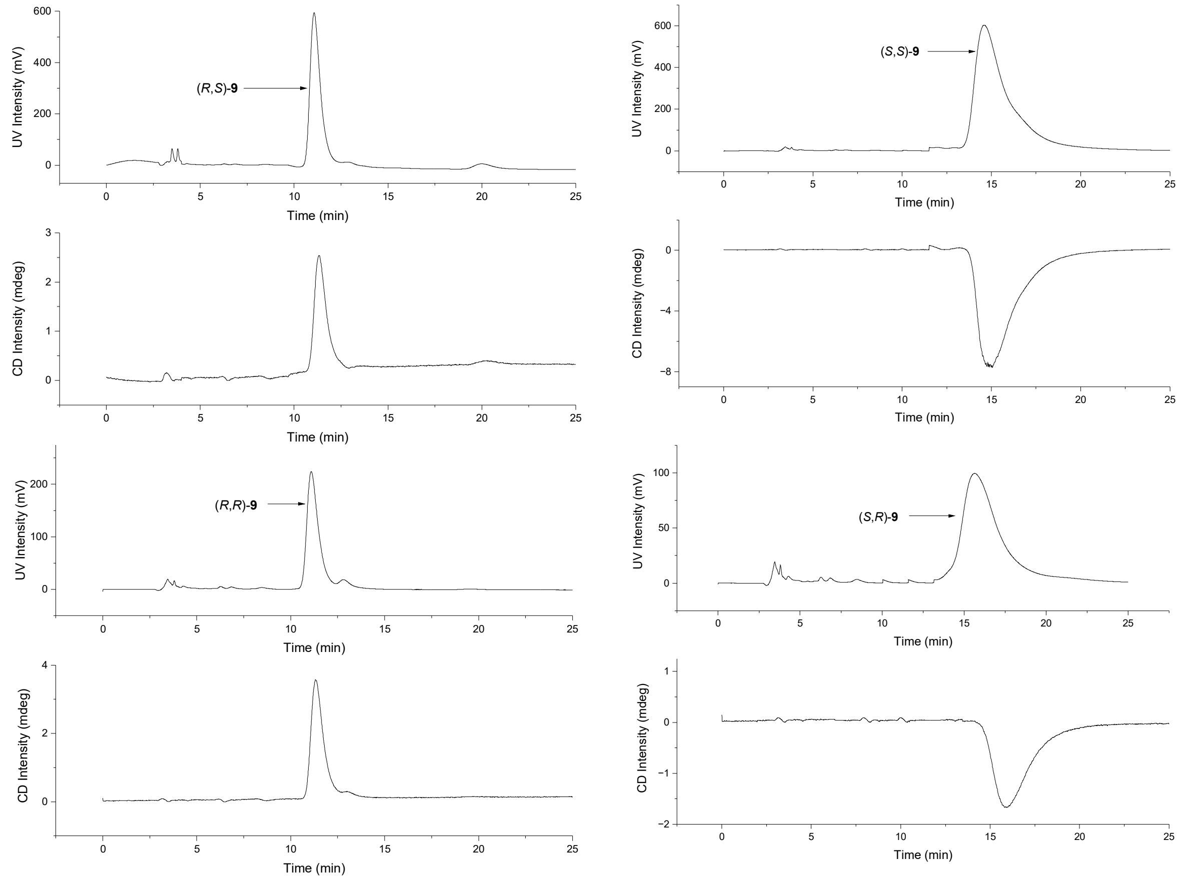

*Figure S7: Chromatograms of (R,S)-**9**, (S,S)-**9**, (R,R)-**9** and (S,R)-**9** using CHIRALPAK®IA (250 x 4.6 mm) with 62 % of MeCN in aqueous 20 mM NH4OAc at a flow rate of 1 mL/min with CD detection at 268 nm.*

Synthesis of 8, (*S*)-8, and (*R*)-8

The enantiopure (*S*)-and (*R*)-**8** were synthesized through asymmetric reduction of 1-(3-aminophenyl)ethan-1-one with catalyst (*R*)- and (*S*)- RUCY-xylBINAP respectively (Scheme S1).[4] The HPLC analysis using a ChiralPaK IB column with 80 % of *n*-Hex in *i*-PrOH showed that both compounds (*S*)-and (*R*)-**8** were enantiomerically pure (e.e. > 99 %).

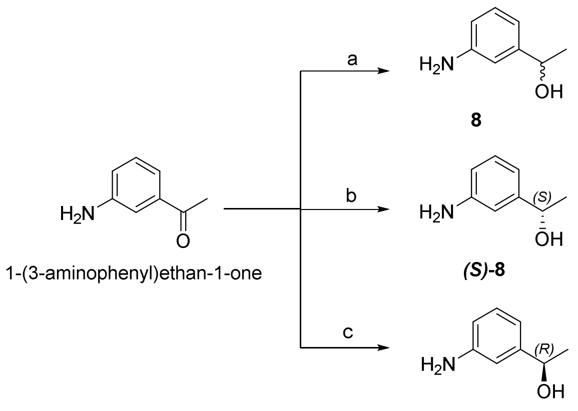

*Scheme S1: Synthesis of **8**, **(S)-8** and **(R)-8**. Reagents and conditions: a) $NaBH_4$, MeOH, r.t, 5 h (yield 89 %); b) H_2, (R)-RUCY-xylBINAP, t-BuOK, i-PrOH, r.t, 16 h (yield 69 %, e.e > 99 %); c) H_2, (S)-RUCY-xylBINAP, t-BuOK, i-PrOH, r.t, 16 h (yield 74 %, e.e > 99 %).*

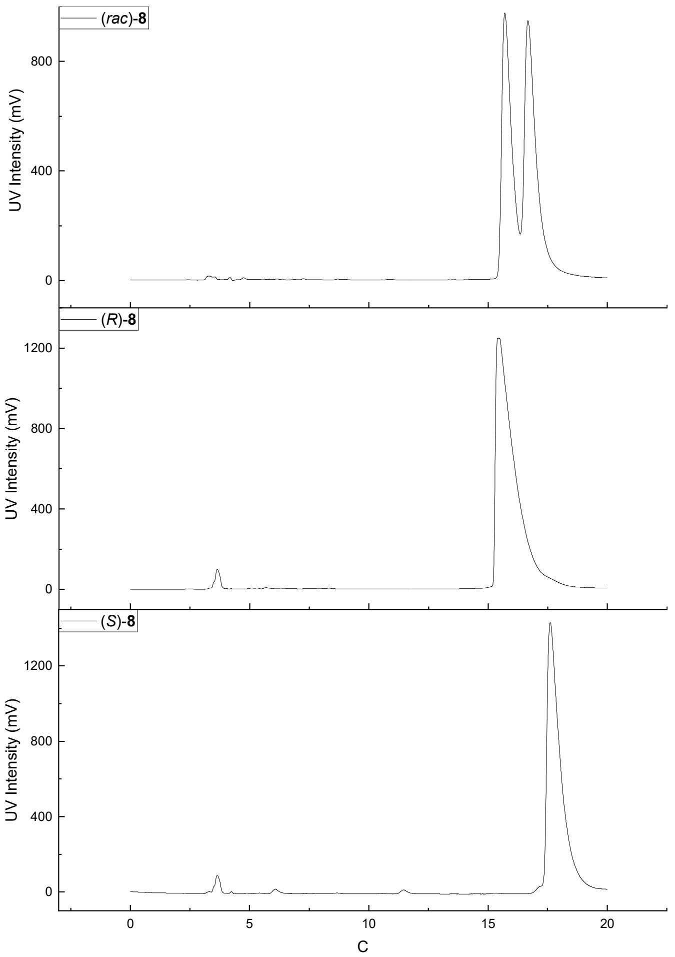

*Figure S8: Chromatograms of rac-**8**, (R)-**8** and (S)-**8** using CHIRALPAK®IB (250 x 4.6 mm) with 80 % of hexane i-PrOH at a flow rate of 1 mL/min at 230 nm.*

S5: Formation of protodeboronated side product (51) during the radiosynthesis [^{18}F]SK60

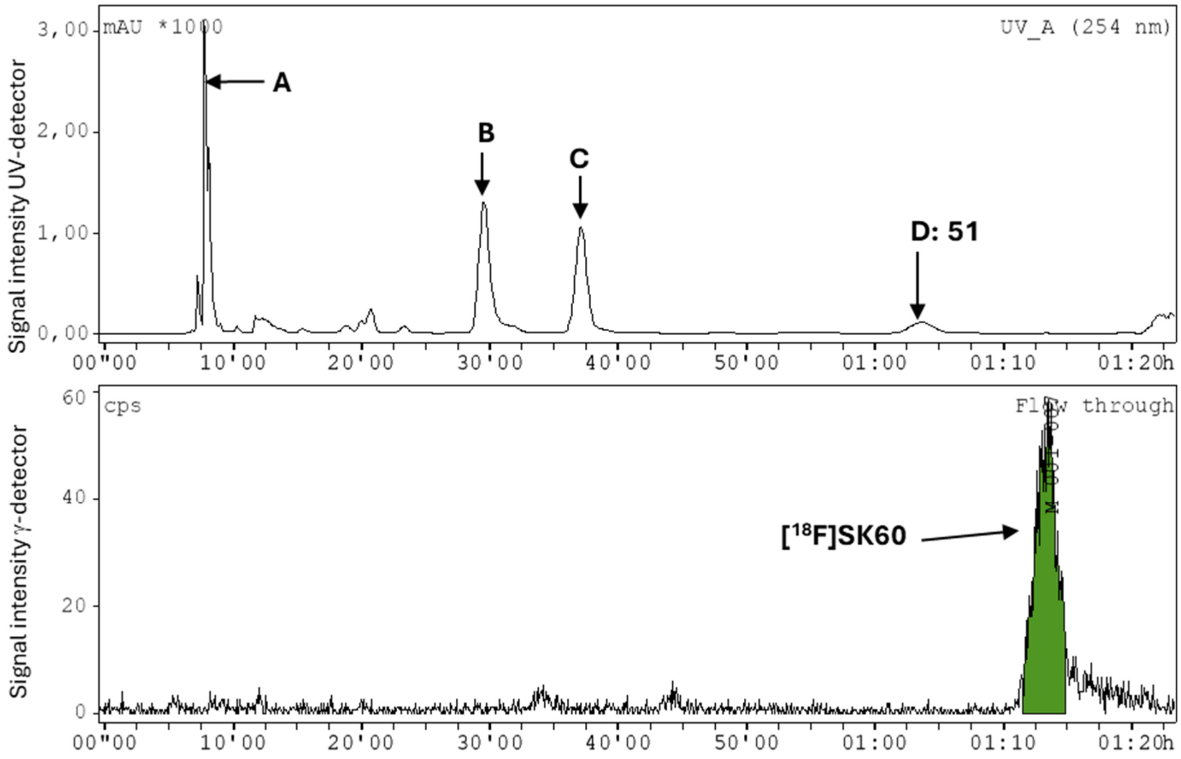

*Figure S9: Representative radio- and UV chromatogram obtained for the isolation of **[^{18}F]SK60** by semi-preparative HPLC with a Reprosil-Pur C18-AQ, 250 × 20 mm column from Dr. Maisch GmbH using a mixture of 48 % MeCN: THF 1:1 (v/v) and 20 mM NH_4OAc_{aq}, flow 7.8 mL/min. In the chromatogram, **A** results from [Cu] catalyst; **B** is the boronic acid resulting from the hydrolysis of the precursor 64; **C** is the unidentified impurity and **D** is the protodeboronated side product (**51**).*

The formation of the protodeboronated side product (**51**, Figure S9) during the radiosynthesis of **[^{18}F]SK60** was confirmed by analyzing the HPLC fraction containing the product by LCMS. The chromatogram of the decayed isolated HPLC isolated fraction of the reaction mixture containing **[^{18}F]SK60** and by-product (chromatogram A, Figure S10) was compared with reference **51** (chromatogram B, Figure S10) and **SK60** (chromatogram C, Figure S10) using LCMS. In chromatogram A, a peak with the same retention time (t_R = 11.650 min) and mass (468) as reference **51** (t_R = 11.653 min and 468).

Following the confirmation of the protodeboronated side product, a mixture of reference **51** and **SK60** was analytically investigated with different RP- HPLC columns and eluent mixtures to determine the most suitable separating conditions (Table S3). Among the evaluated stationary phases, the pentafluorophenyl propyl (PFP) column (Nucleodur PFP) with 52 % MeCN and 20 mM NH_4OAc_{aq} provided efficient separation. Additionally, mobile phases containing THF with 20 mM NH_4OAc_{aq}, as well as a mixture of THF/MeCN 1/1 (*v/v*) with 20 mM NH_4OAc_{aq}, achieved separation on other stationary phases, including Reprosil-Pur C18-AQ, Reprosil-Gold C18, and Reprosil-Pur CN (some representative chromatograms are shown below, Figure S11. These analytical HPLC findings were then investigated on the semi-preparative HPLC of **[^{18}F]SK60**. The use of a Reprosil-Pur C18-AQ, 250 × 20 mm column with a mobile phase comprising 48 % MeCN/THF 1/1 (*v/v*) and 20 mM NH4OAc$_{aq}$ with a flow of 7.8 mL/min resulted in the most efficient separation of **51** from the **[^{18}F]SK60**.

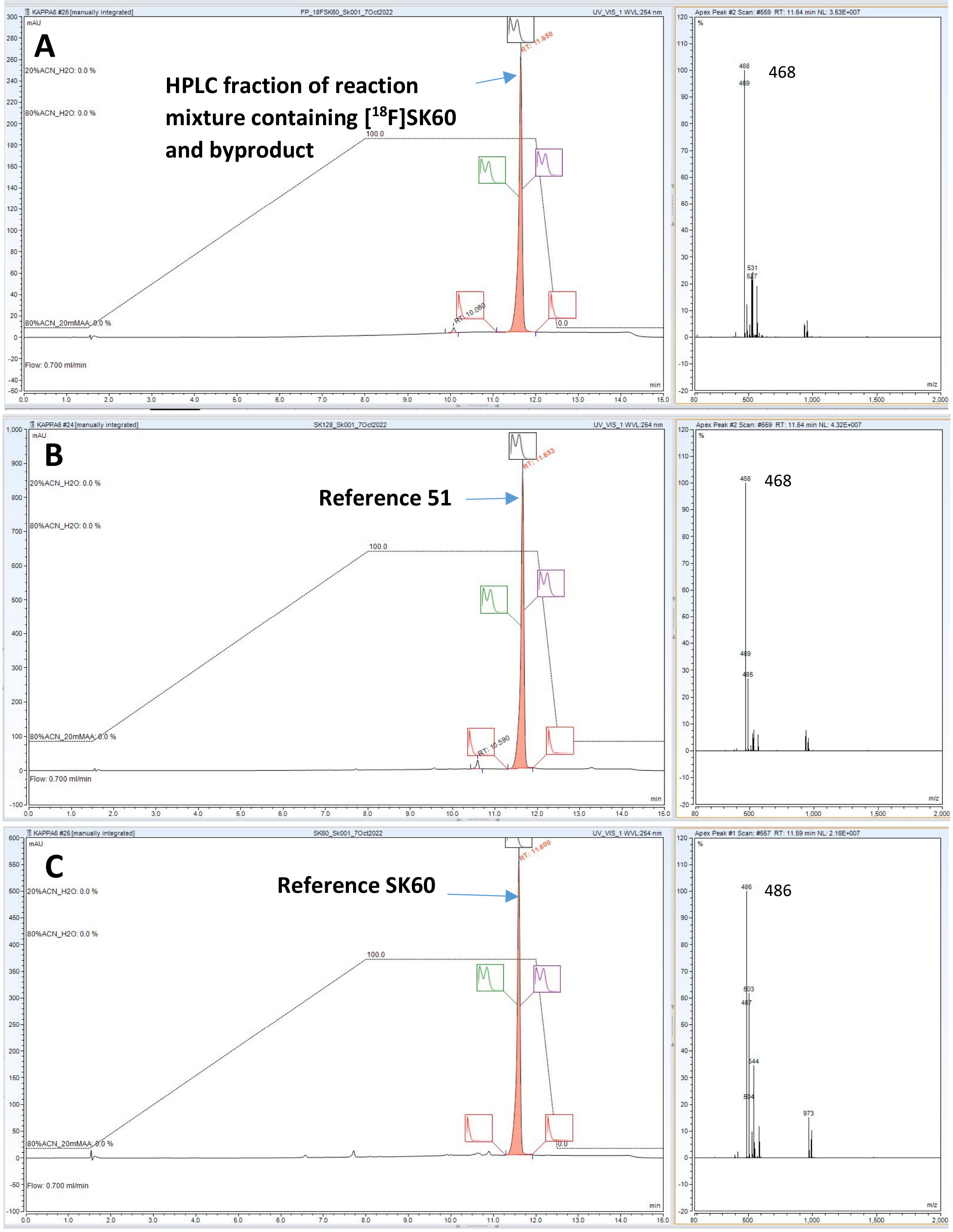

*Figure S10: LCMS chromatograms of the HPLC isolated fraction of reaction mixture containing **[18F]SK60** (decayed) and byproduct **[18F]SK60**) (A), reference **51** (B) and **SK60** (C) in [M+H]$^+$ mode, confirming the formation of protodeboronated side product (**51**) during the radiosynthesis of **[18F]SK60**.*

Analytical HPLC investigation of a mixture of 51 and SK60

*Table S3: Analytical investigation of a mixture of **51** and **SK60** with different RP- HPLC columns and eluent mixtures to determine the most suitable separating conditions.*

Column	Stationary phase type	Mobile phase (isocratic mode)
Reprosil-Pur 120 C18-AQ[a]	hydrophobic, polar	-MeCN/20 mM NH_4OAc_{aq}; 1 mL/min<br>-MeCN/THF/20 mM NH_4OAc_{aq}; 1 mL/min
Reprosil-Gold 120 C18[a]	strong hydrophobic	-MeCN/20 mM NH_4OAc_{aq}; 1 mL/min<br>-MeCN/THF/20 mM NH_4OAc_{aq}; 1 mL/min<br>-THF/20 mM NH_4OAc_{aq} ; 0.7 mL/min
Reprosil-Pur Basic-C18-HD[a]	strong hydrophobic	-MeCN/20 mM NH_4OAc_{aq}; 1 mL/min
Reprosil-Pur 120 Phenyl[a]	hydrophobic, polar	-MeCN/20 mM NH_4OAc_{aq}; 1 mL/min<br>-MeOH/20 mM NH_4OAc_{aq}; 0.7 mL/min<br>-MeOH/0.05 % TFA_{aq}; 0.7 mL/min
Reprosil-Pur 120 CN[a]	hydrophobic, polar	-MeCN/20 mM NH_4OAc_{aq}; 1 mL/min<br>-MeCN/0.05 % TFA_{aq}; 1 mL/min<br>-MeOH/20 mM NH_4OAc_{aq}; 0.7 mL/min<br>-MeOH/0.05 % TFA_{aq}; 0.7 mL/min<br>-MeCN/THF/20 mM NH_4OAc_{aq}; 1 mL/min<br>-THF/20 mM NH_4OAc_{aq}; 0.7 mL/min
Nucleoshell[b]	hydrophobic	-MeCN/20 mM NH_4OAc_{aq}; 0.4 mL/min
X Bridge[c]	n/a	-MeCN/20 mM NH_4OAc_{aq}; 0.4 mL/min
Nucleodur sphrinx[a]	hydrophobic, polar	-MeCN/20 mM NH_4OAc_{aq}; 1 mL/min
Nucleodur PFP[a]	hydrophobic, polar, steric selectivity	-MeCN/20 mM NH_4OAc_{aq}; 1 mL/min

[a]250*4.6 mm, 5 µm particle size
[b]150*3.0 mm, 3.5 µm particle size
[c]150*3.0 mm, 3.5 µm particle size

Representative chromatograms showing the separation of 51 and SK60

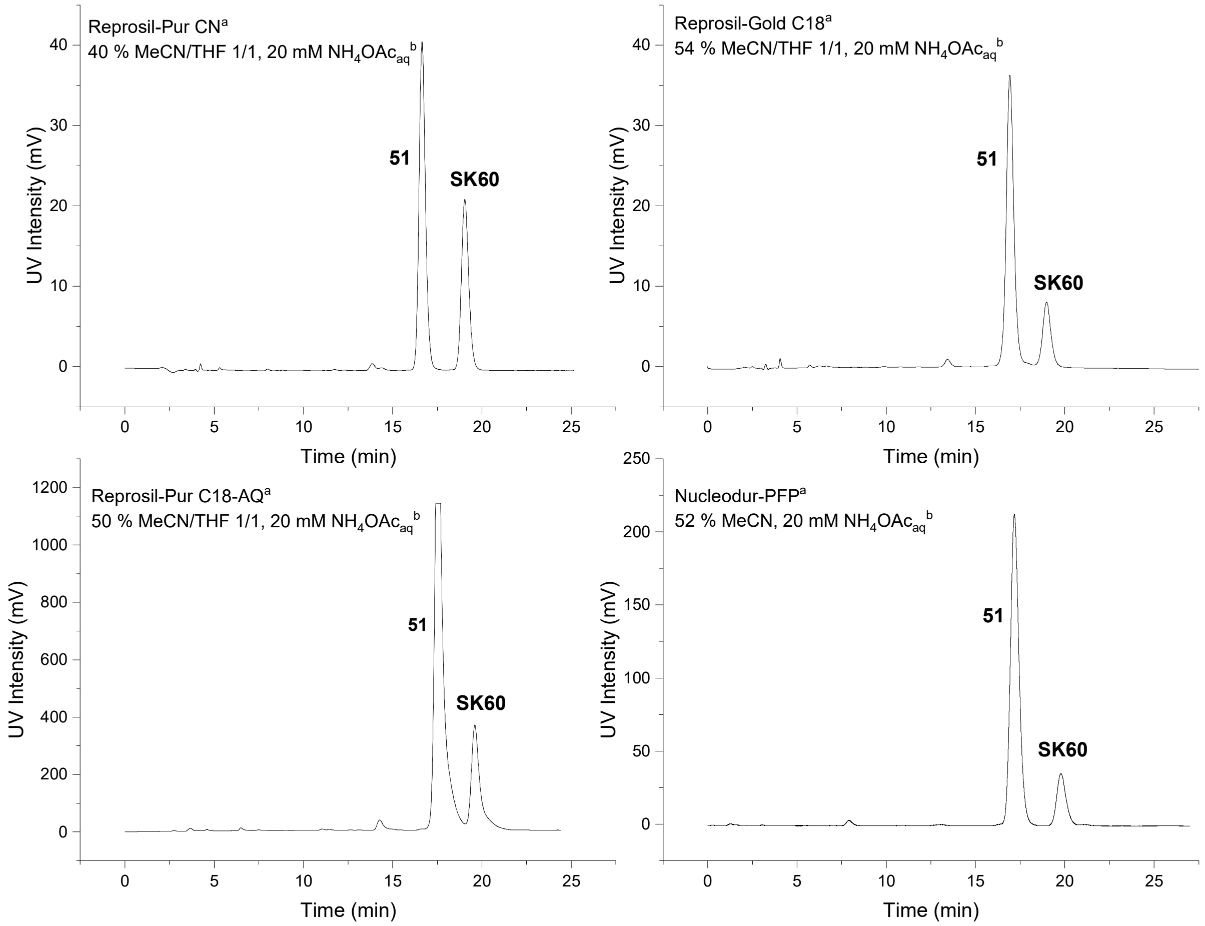

*Figure S11: Representative chromatograms for the separation of **51** from **SK60,** [a]250*4.6 mm, 5 µm particle size; [b]flow 1 mL/min.*

S6: Calibration curve of SK60

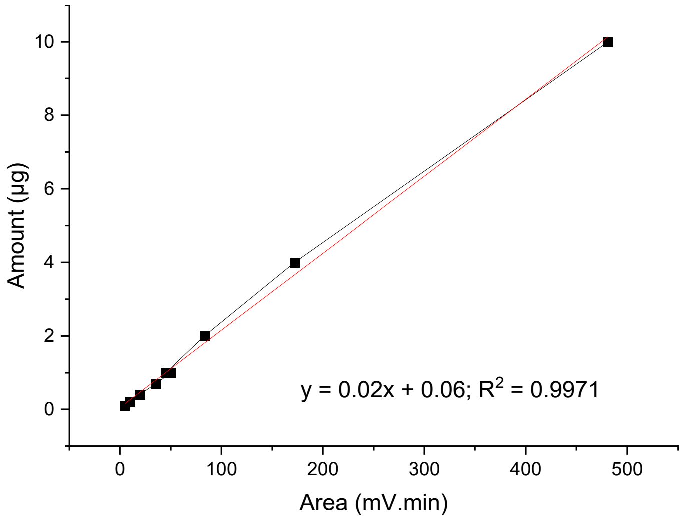

*Figure S12: Calibration curve of **SK60** for the determination of molar activity. HPLC conditions: Nucleodur PFP (250 × 4.6 mm; 5 µm), isocratic mode 52 % MeCN/20 mM NH_4OAc_{aq}, 1 mL/min, 268 nm.*

S7: Schematic interface for the automated radiosynthesis

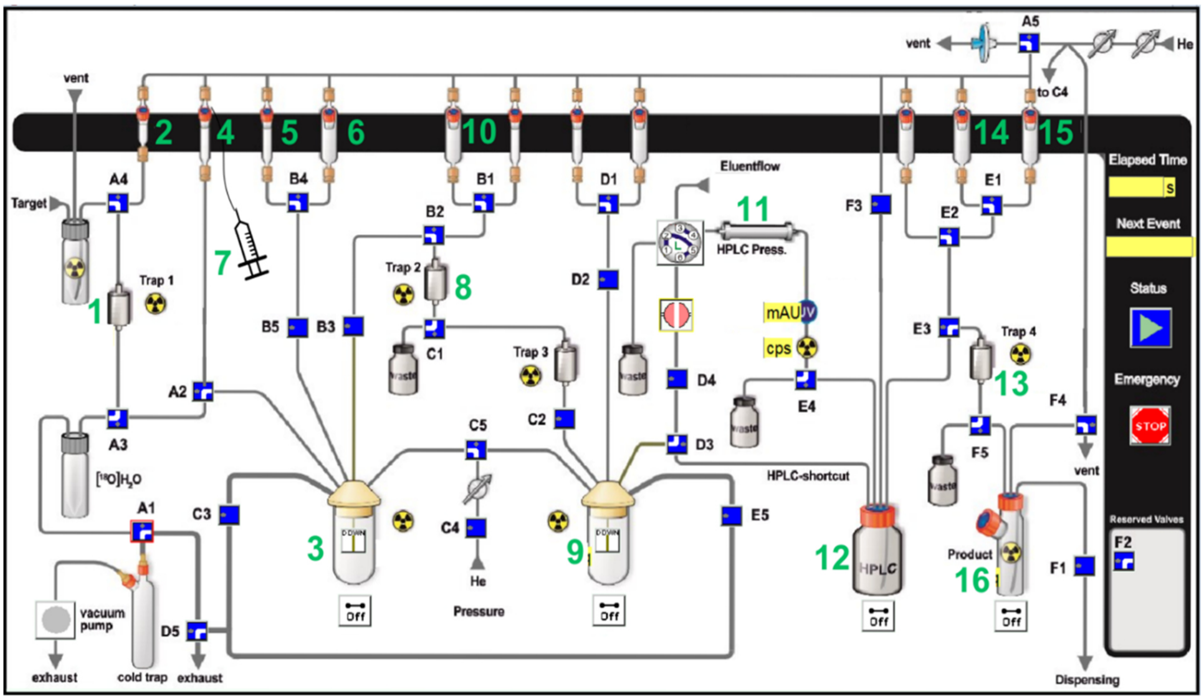

*Figure S13: Scheme of the Raytest SynChrom R&D synthesis module for the automated radiosynthesis of **[^{18}F]SK60**. 1) Sep-Pak® Accell Plus QMA Plus Light cartridge; 2) 100 µL $TBAHCO_3$, 800 µL MeCN, and 200 µL water mixture; 3) Reactor 1; 4) 1.5 mL MeCN; 5) 15 µmol [Cu] in 600 µL DMI; 6) 3.5 µmol of **64** in 300 µL n-BuOH; c) 2.5 mL of MeCN/THF 1/1; 7.external) a) 0.4 % of TFA in water (1 mL); b) 18 mL water; 8) Sep-Pak® C18 Plus cartridge: 9) Reactor 2; 10) 2.5 mL water; 11) Reprosil-Pur C18-AQ, 250 × 20 mm column from Dr. Maisch GmbH using a mixture of 48 % MeCN/THF 1/1 20 mM NH_4OAc_{aq} flow 7.8 mL/min; 12) 30 mL water; 13) Sep-Pak® C18 light; 14) 2 mL water; 15) 1.2 mL EtOH; 16) product vial.*

S7: Quality control of [^{18}F]SK60

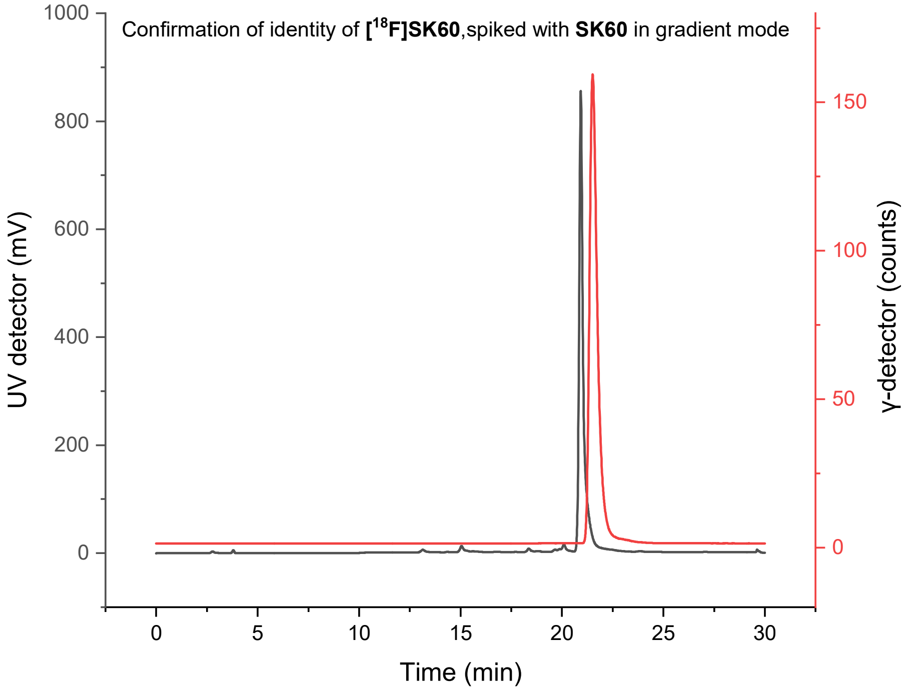

*Figure S14: HPLC analysis of the formulated product **[^{18}F]SK60** spiked with **SK60**, the difference between the retention times of **SK60** (t_R = 20.9 min) and **[^{18}F]SK60** (t_R = 21.4 min) corresponds to the delay between UV and radio detection. HPLC conditions: Reprosil C18-AQ, 250 x 4.6 mm, 5 µm, linear gradient system with a mixture of MeCN/20 mM NH_4OAc_{aq}, flow 1 mL/min, 268 nm.*

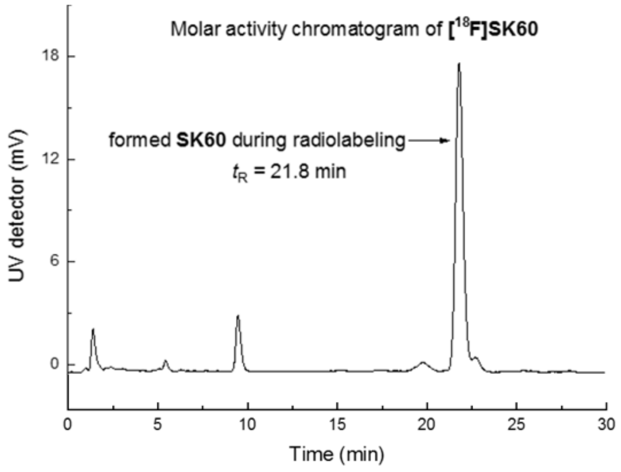

*Figure S15: Molar activity chromatogram of 50 µL of formulated product **[^{18}F]SK60.** HPLC conditions: Nucleodur PFP from Macherey-Nagel, 250 x 4.6 mm, 5 µm, 52 % MeCN/20 mM NH_4OAc_{aq}, flow 1 mL/min at 268 nm.*

S8: NMR of final compounds

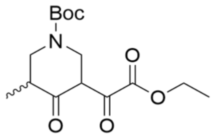

2

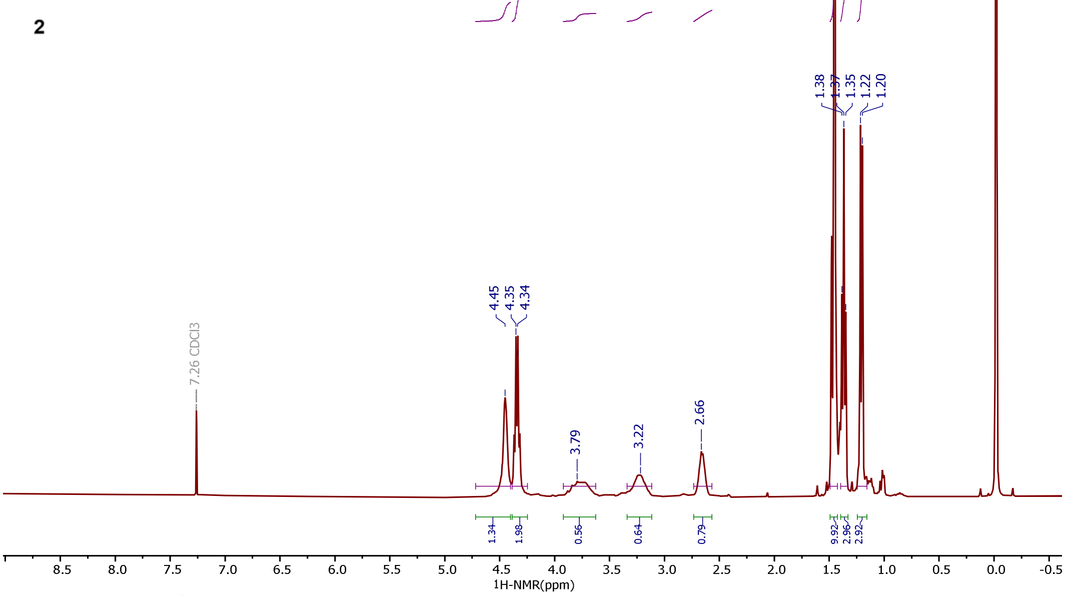

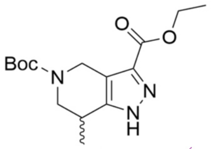

3

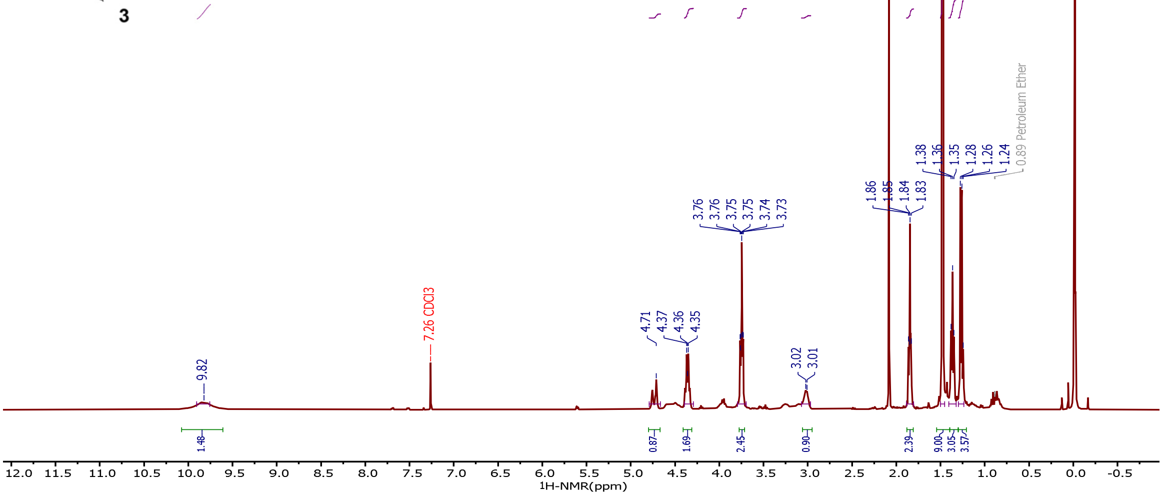

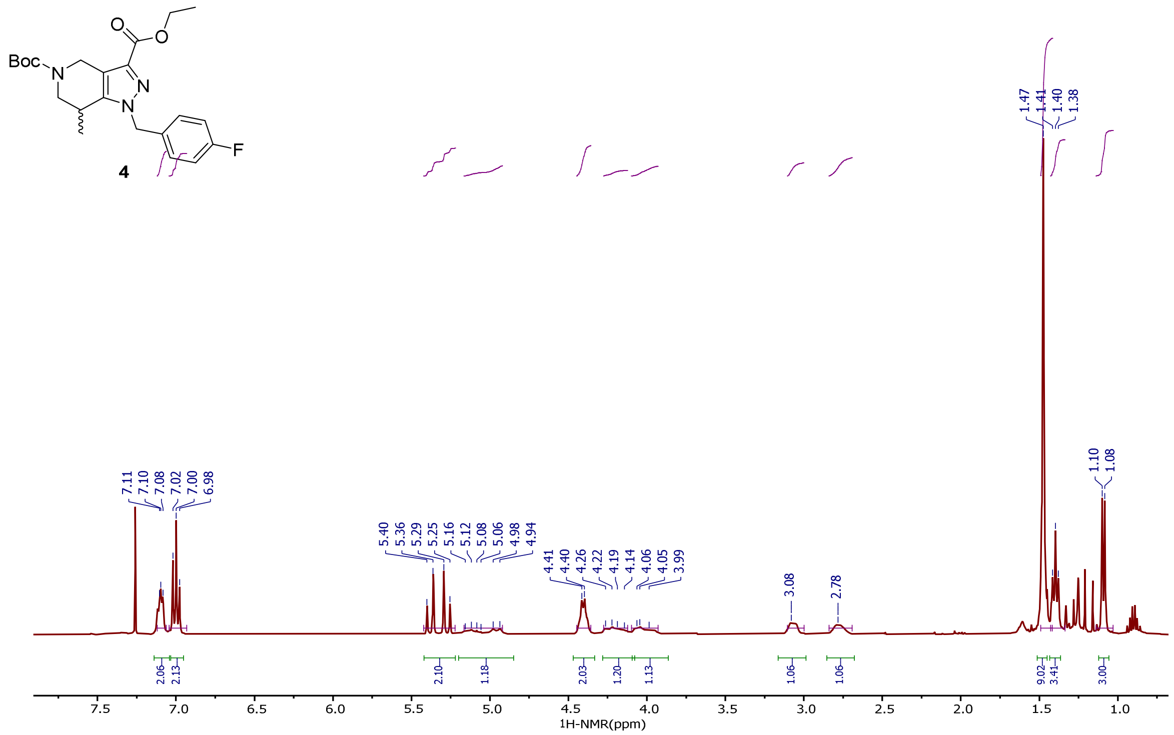
4
1H-NMR(ppm)

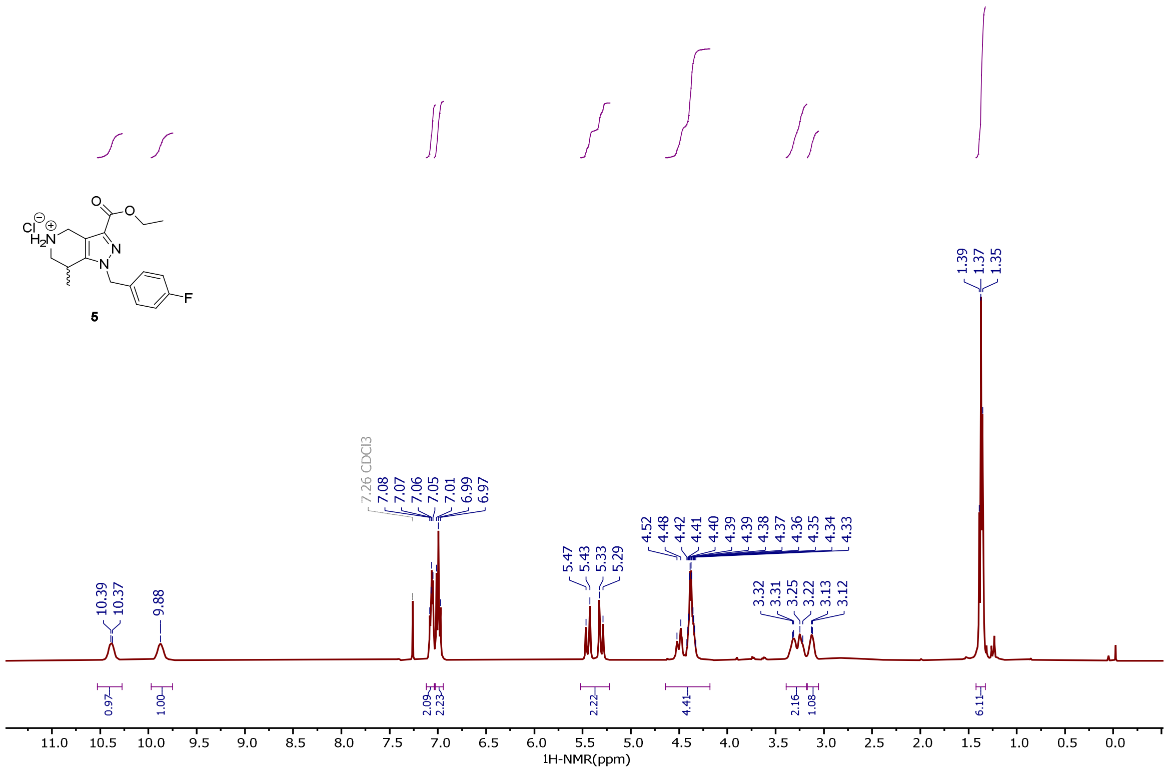
5
7.26 CDCl3
1H-NMR(ppm)

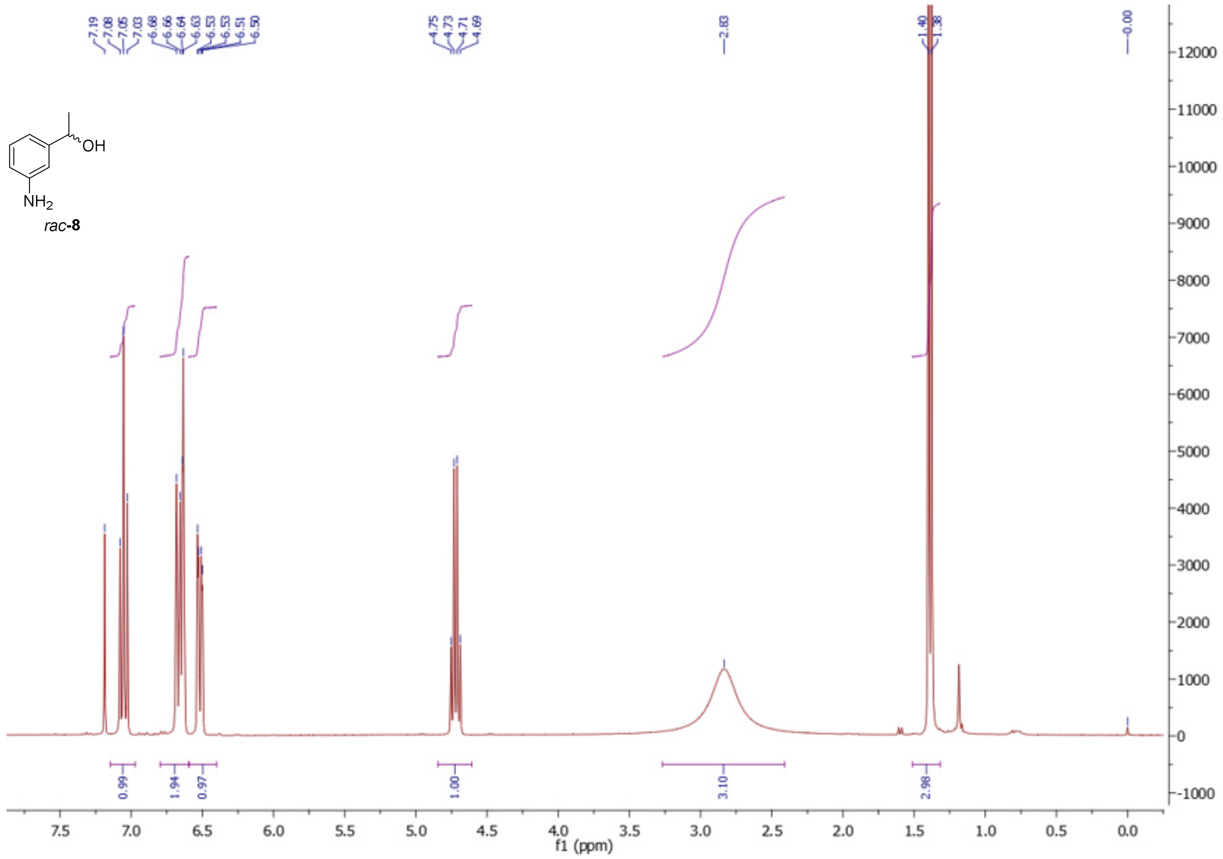

rac-**8**

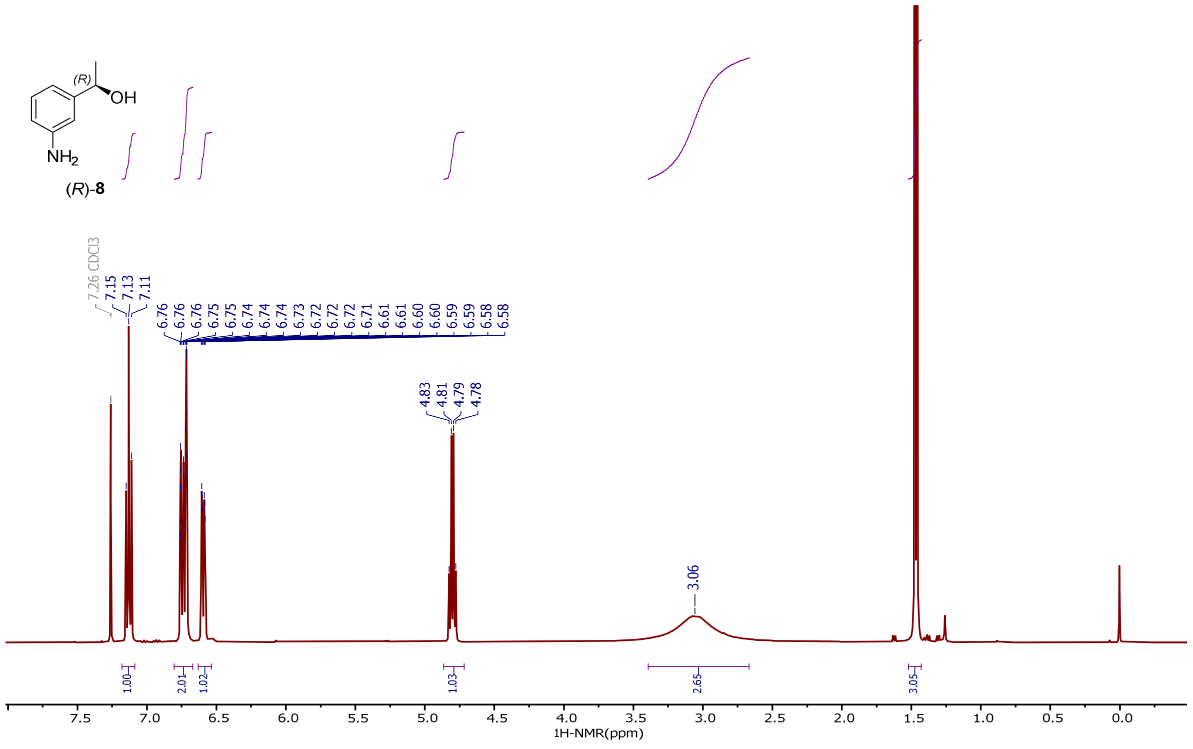

(*R*)-**8**

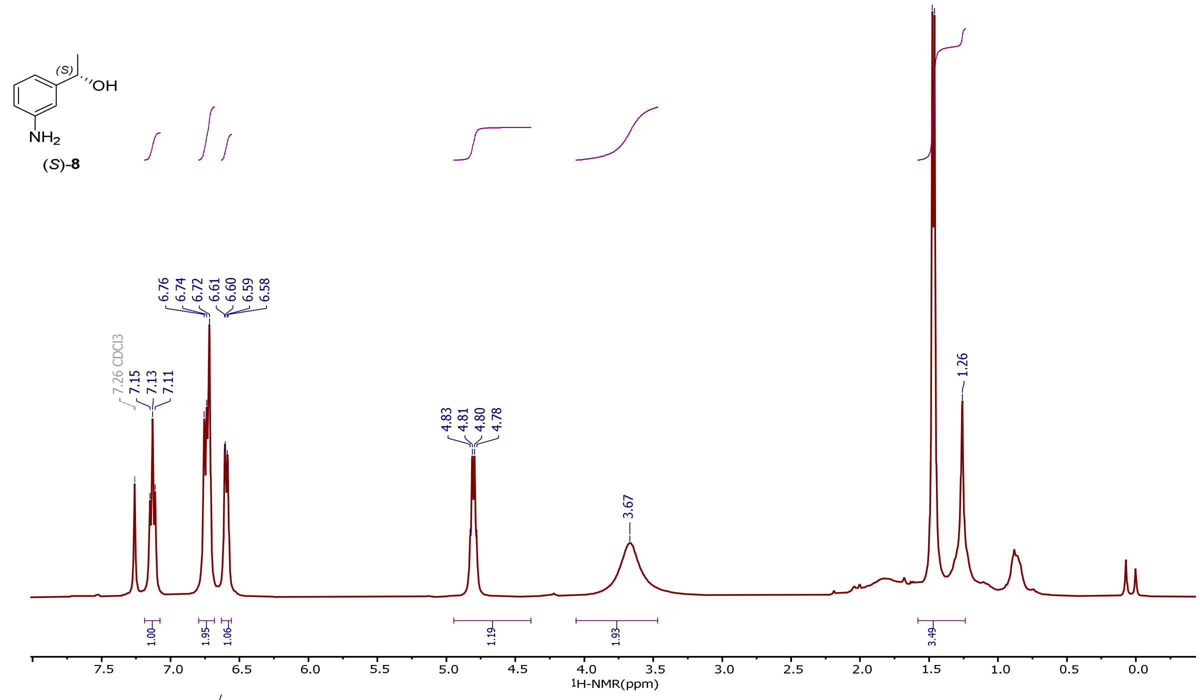
(S)-8
7.26 CDCl3
1H-NMR(ppm)

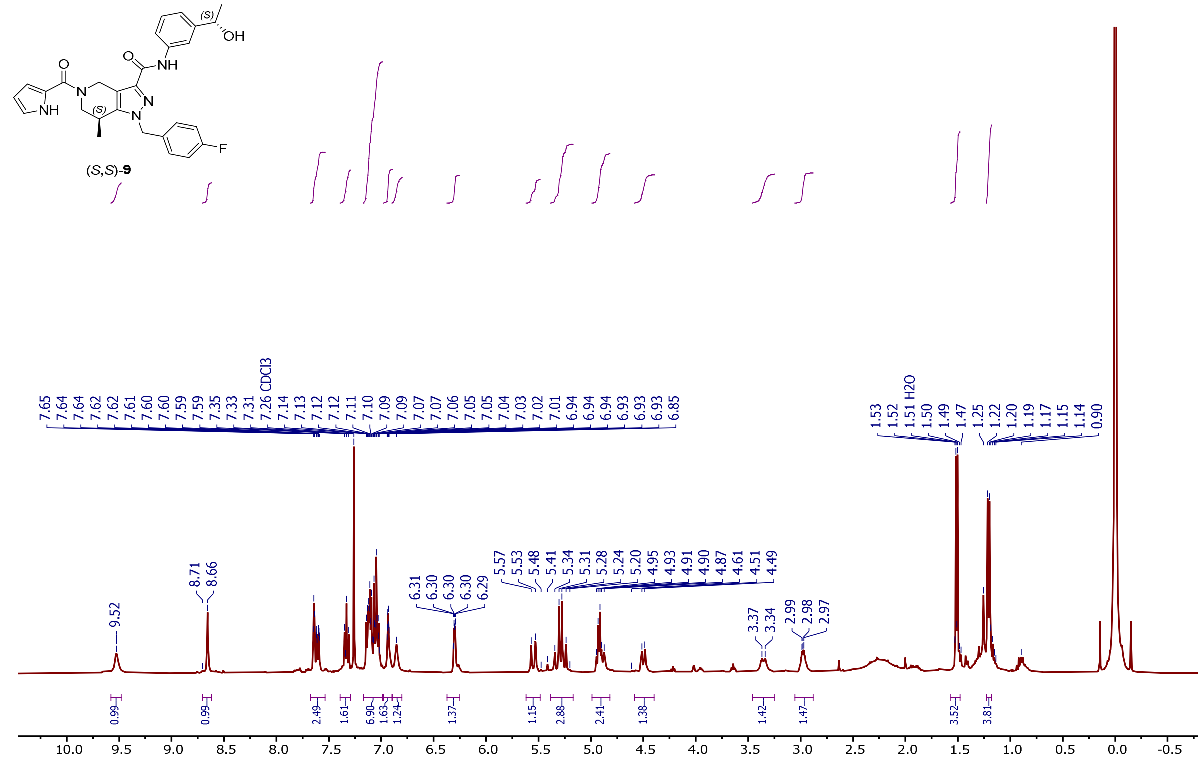
(S,S)-9
7.26 CDCl3
1.51 H2O
1H-NMR(ppm)

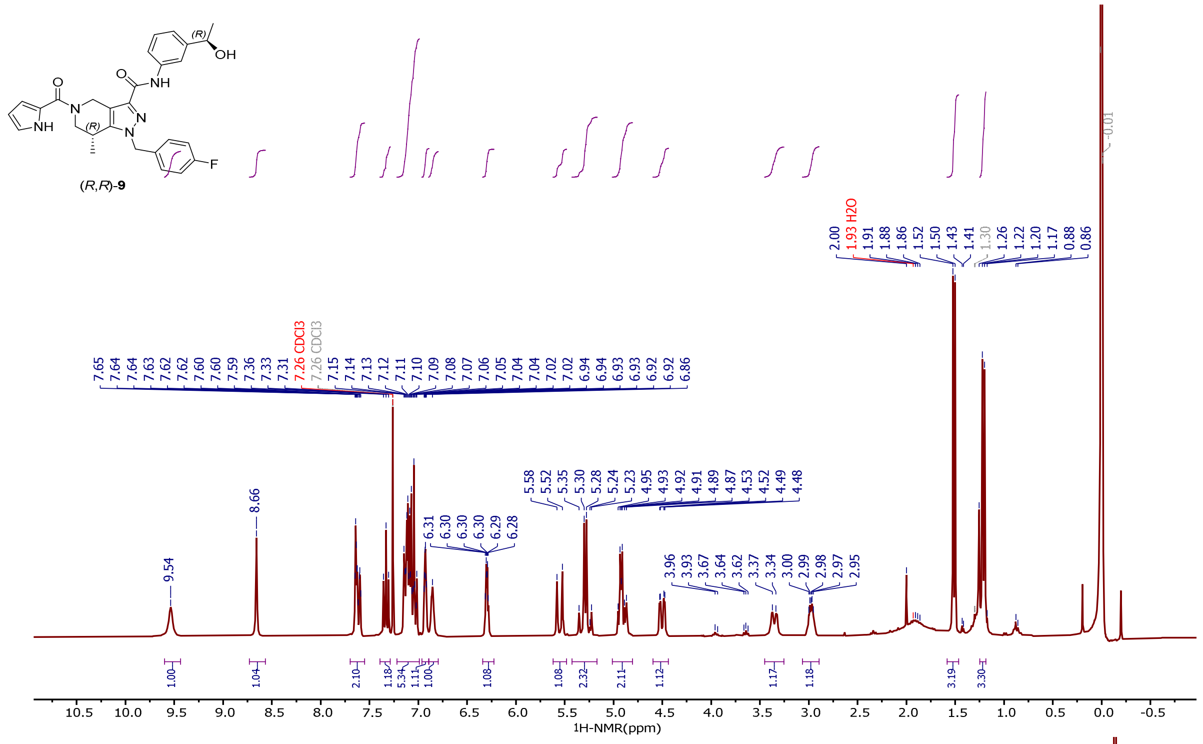
(R,R)-9
1H-NMR(ppm)

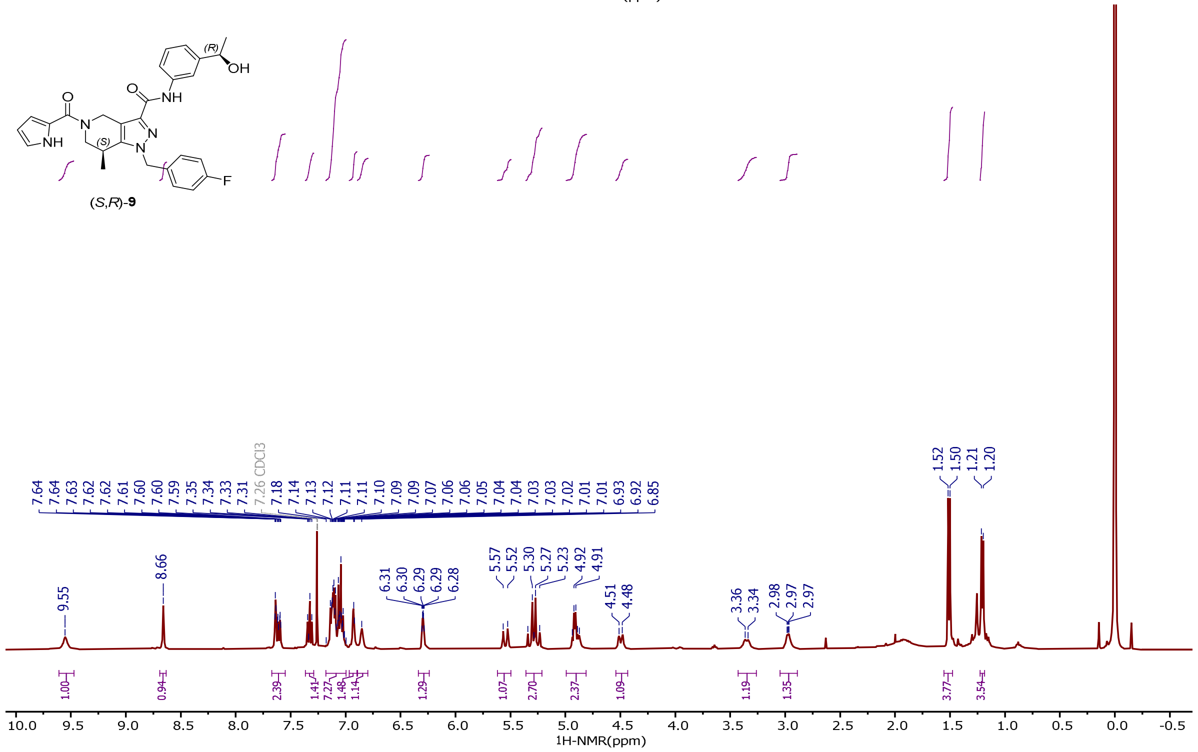
(S,R)-9
1H-NMR(ppm)

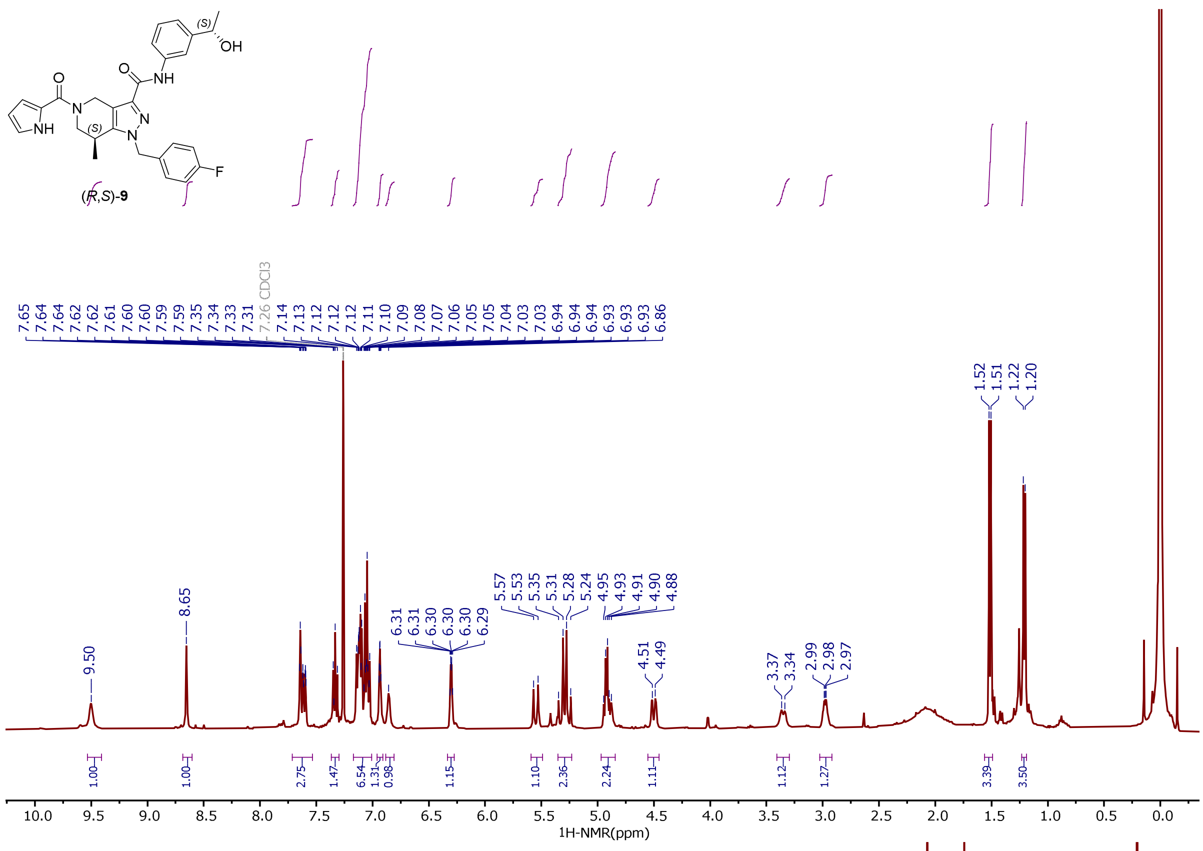

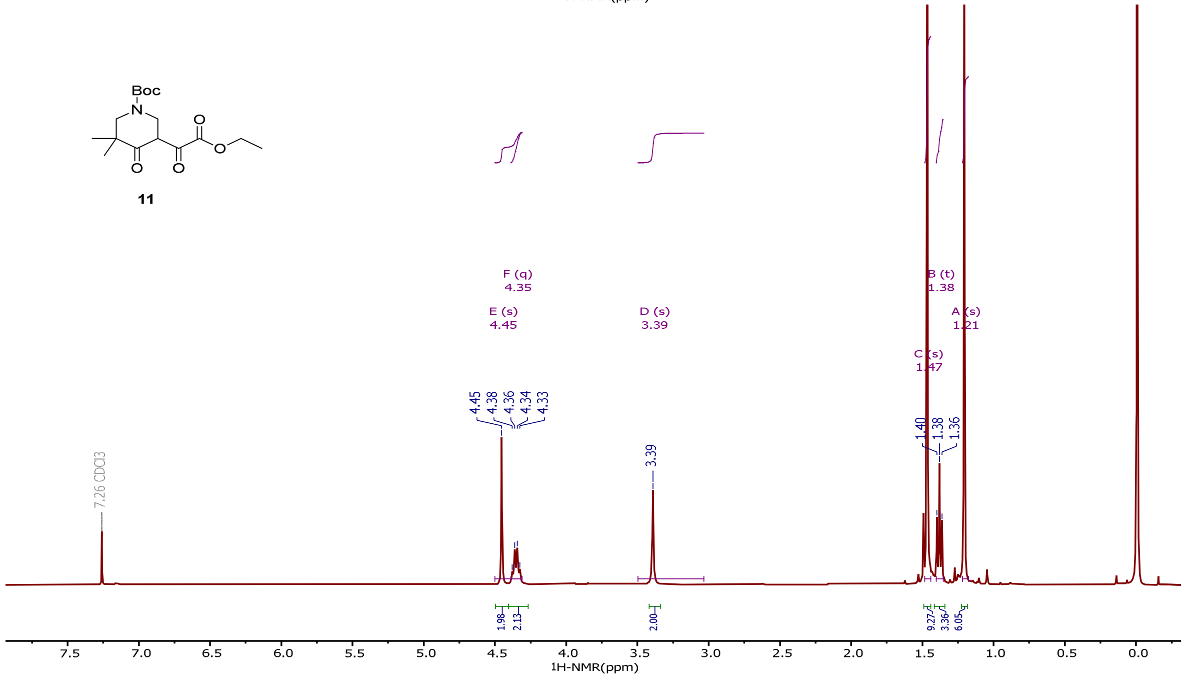

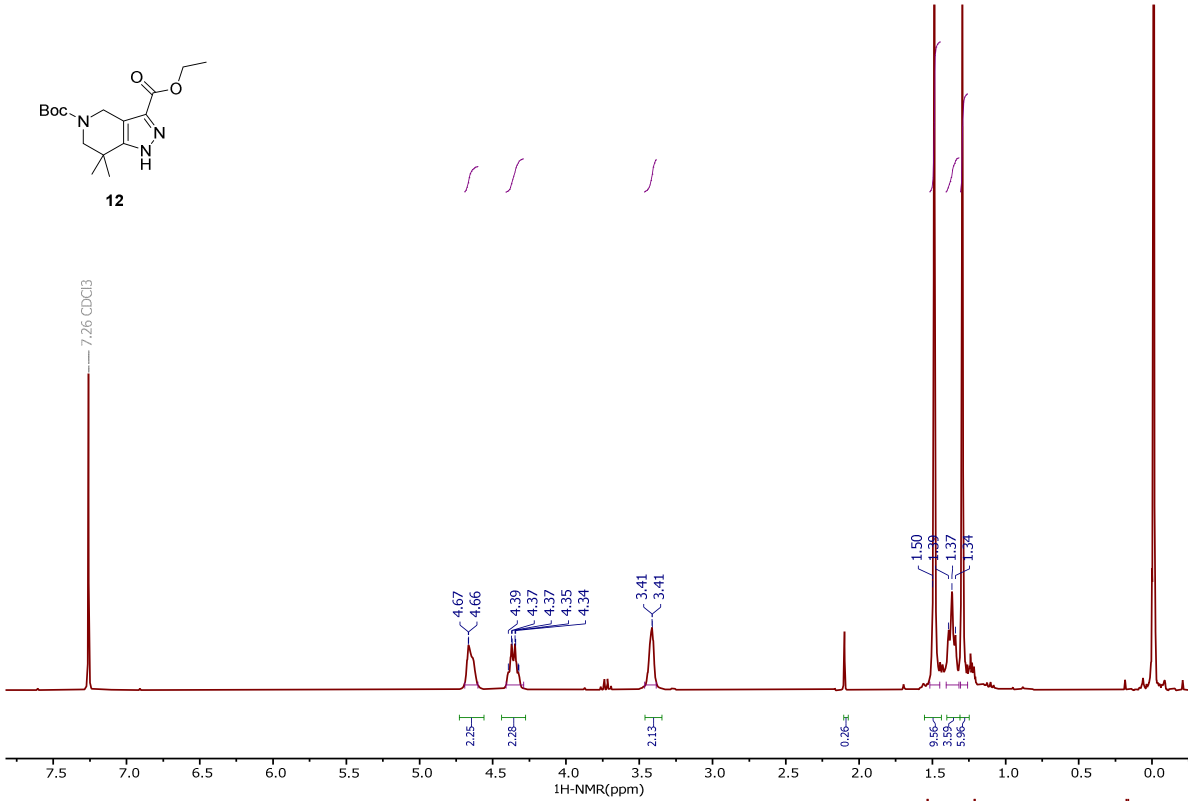
Boc
O
O
N
N
N
H
7.26 CDCl3
4.67
4.66
4.39
4.37
4.37
4.35
4.34
3.41
3.41
1.50
1.39
1.37
1.34
2.25
2.28
2.13
0.26
9.56
3.59
5.96
7.5 7.0 6.5 6.0 5.5 5.0 4.5 4.0 3.5 3.0 2.5 2.0 1.5 1.0 0.5 0.0
1H-NMR(ppm)

12

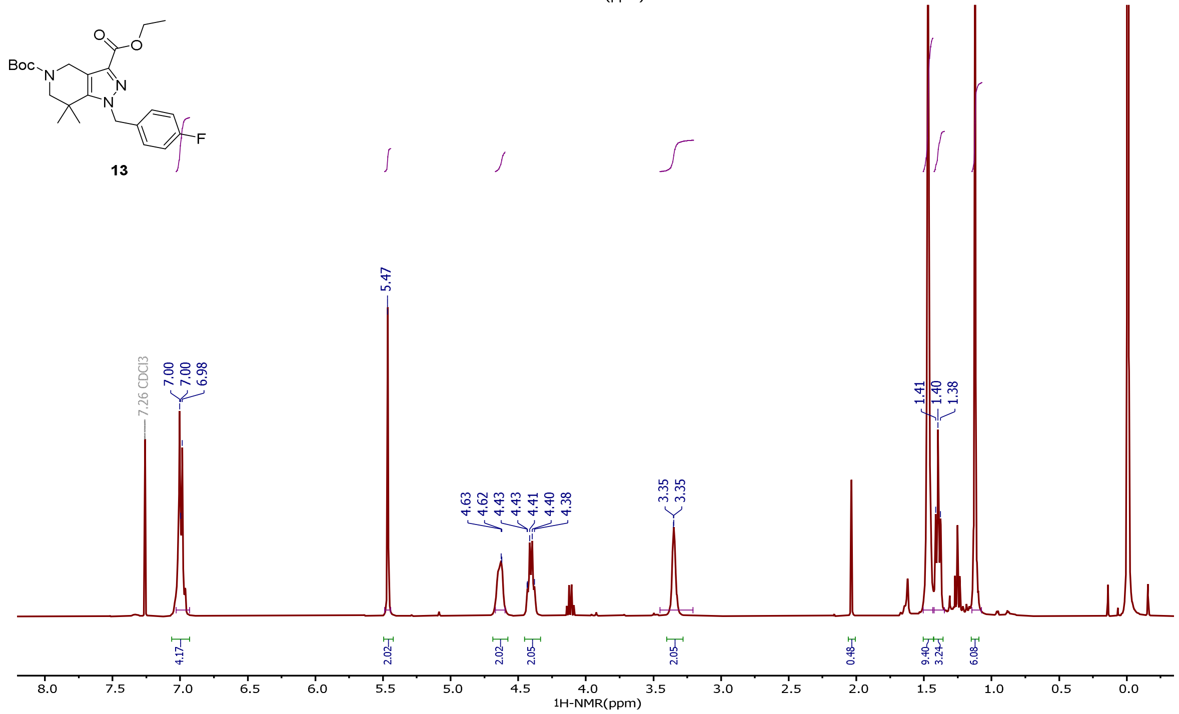
Boc
O
O
N
N
N
F
7.26 CDCl3
7.00
7.00
6.98
5.47
4.63
4.62
4.43
4.43
4.41
4.40
4.38
3.35
3.35
1.41
1.40
1.38
4.17
2.02
2.02
2.05
2.05
0.48
9.40
3.24
6.08
8.0 7.5 7.0 6.5 6.0 5.5 5.0 4.5 4.0 3.5 3.0 2.5 2.0 1.5 1.0 0.5 0.0
1H-NMR(ppm)

13

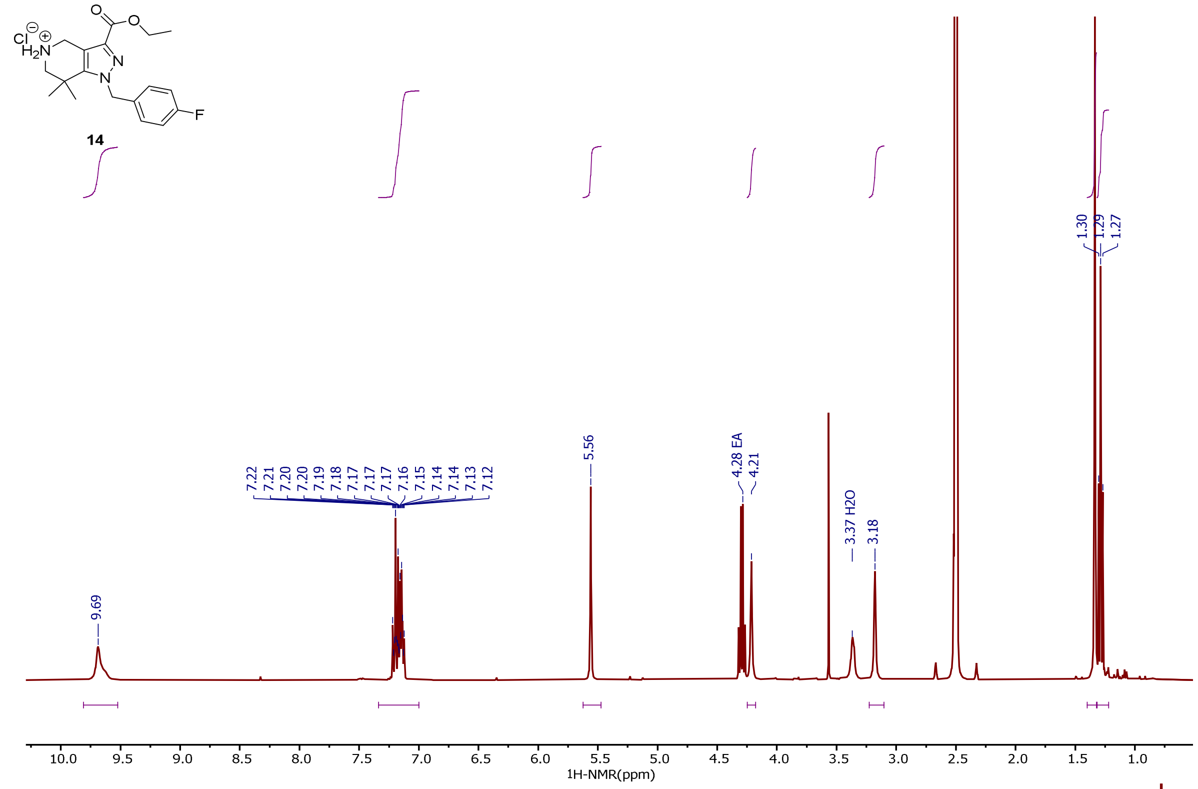

14
9.69
7.22 7.21 7.20 7.20 7.19 7.18 7.17 7.17 7.17 7.16 7.15 7.14 7.14 7.13 7.12
5.56
4.28 EA
4.21
3.37 H2O
3.18
1.30 1.29 1.27
1H-NMR(ppm)

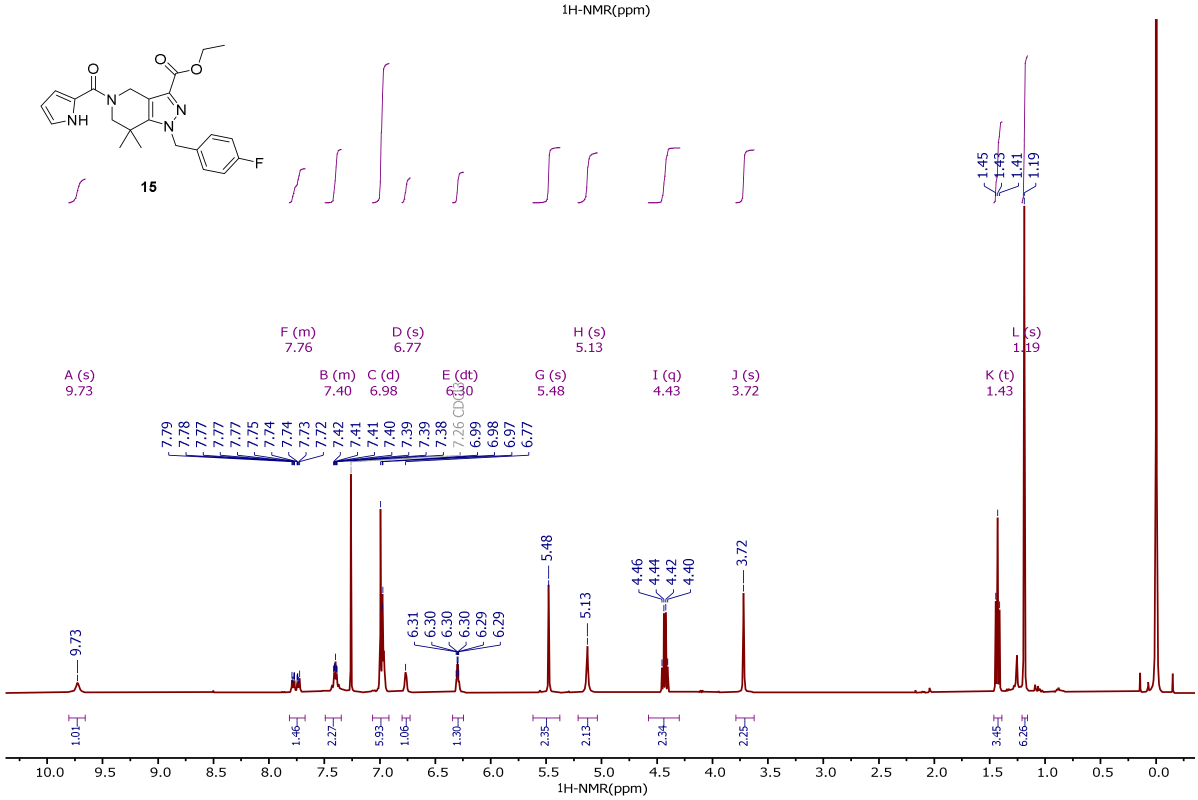

15
A (s) 9.73
F (m) 7.76
B (m) 7.40
C (d) 6.98
D (s) 6.77
E (dt) 6.30
G (s) 5.48
H (s) 5.13
I (q) 4.43
J (s) 3.72
K (t) 1.43
L (s) 1.19
9.73
7.79 7.78 7.77 7.77 7.77 7.75 7.74 7.74 7.73 7.72 7.42 7.41 7.41 7.40 7.39 7.39 7.38 7.26 CDCl3 6.99 6.98 6.97 6.77
6.31 6.30 6.30 6.30 6.29 6.29
5.48
5.13
4.46 4.44 4.42 4.40
3.72
1.45 1.43 1.41 1.19
1.01 1.46 2.27 5.93 1.06 1.30 2.35 2.13 2.34 2.25 3.45 6.26
1H-NMR(ppm)

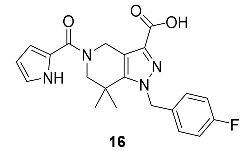
O
OH
N
N
N
NH
F

16

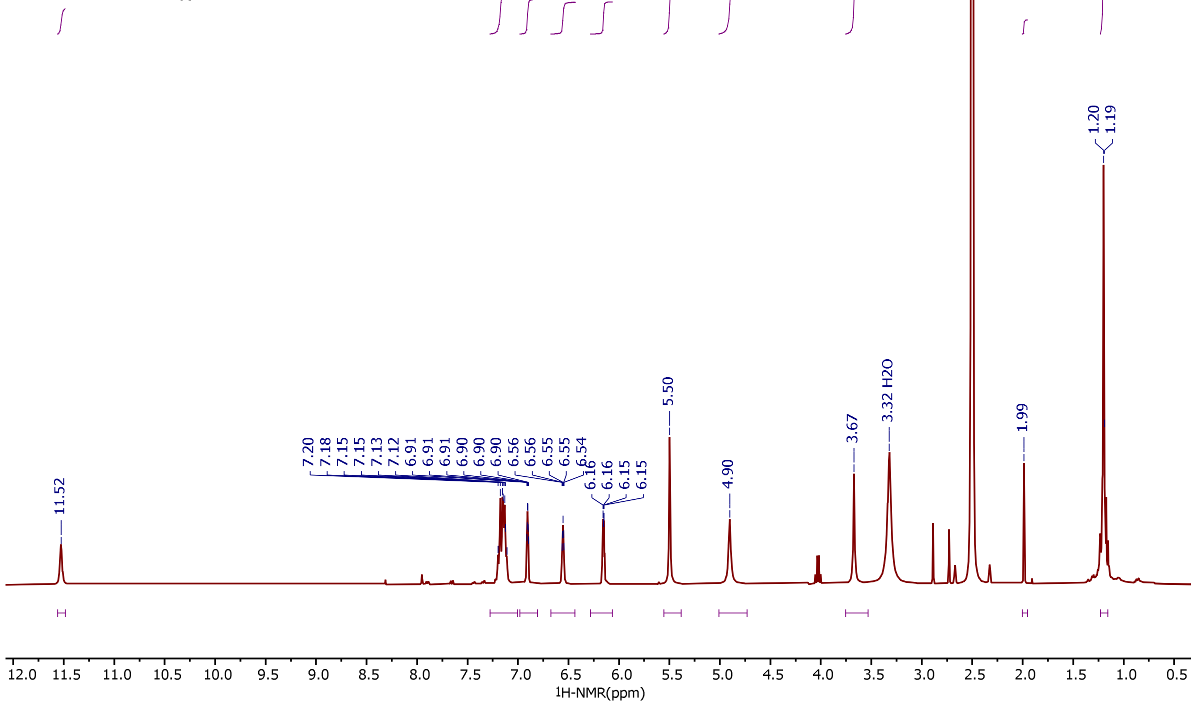
11.52
7.20
7.18
7.15
7.15
7.13
7.12
6.91
6.91
6.91
6.90
6.90
6.90
6.56
6.56
6.55
6.55
6.54
6.16
6.16
6.15
6.15
5.50
4.90
3.67
3.32 H2O
1.99
1.20
1.19
12.0 11.5 11.0 10.5 10.0 9.5 9.0 8.5 8.0 7.5 7.0 6.5 6.0 5.5 5.0 4.5 4.0 3.5 3.0 2.5 2.0 1.5 1.0 0.5
1H-NMR(ppm)

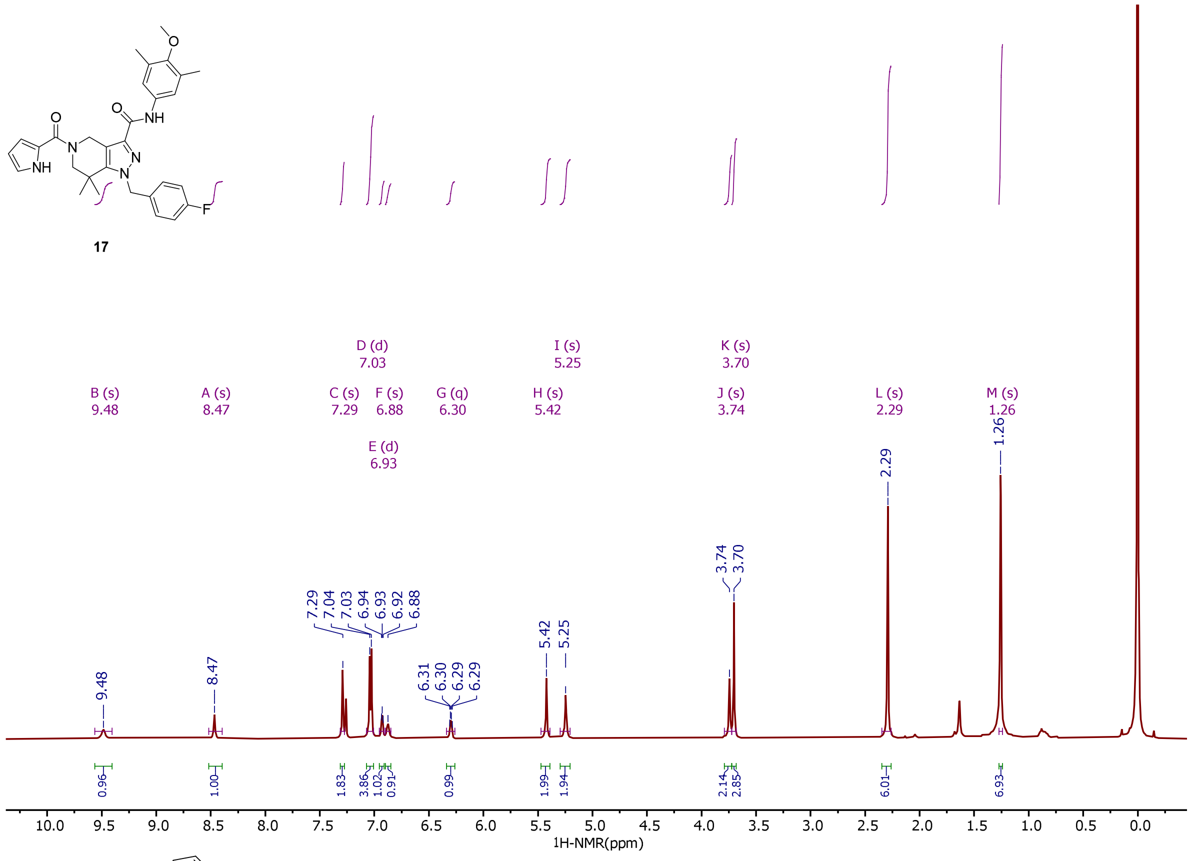
17
B (s) 9.48
A (s) 8.47
C (s) 7.29
D (d) 7.03
E (d) 6.93
F (s) 6.88
G (q) 6.30
H (s) 5.42
I (s) 5.25
J (s) 3.74
K (s) 3.70
L (s) 2.29
M (s) 1.26
1H-NMR(ppm)

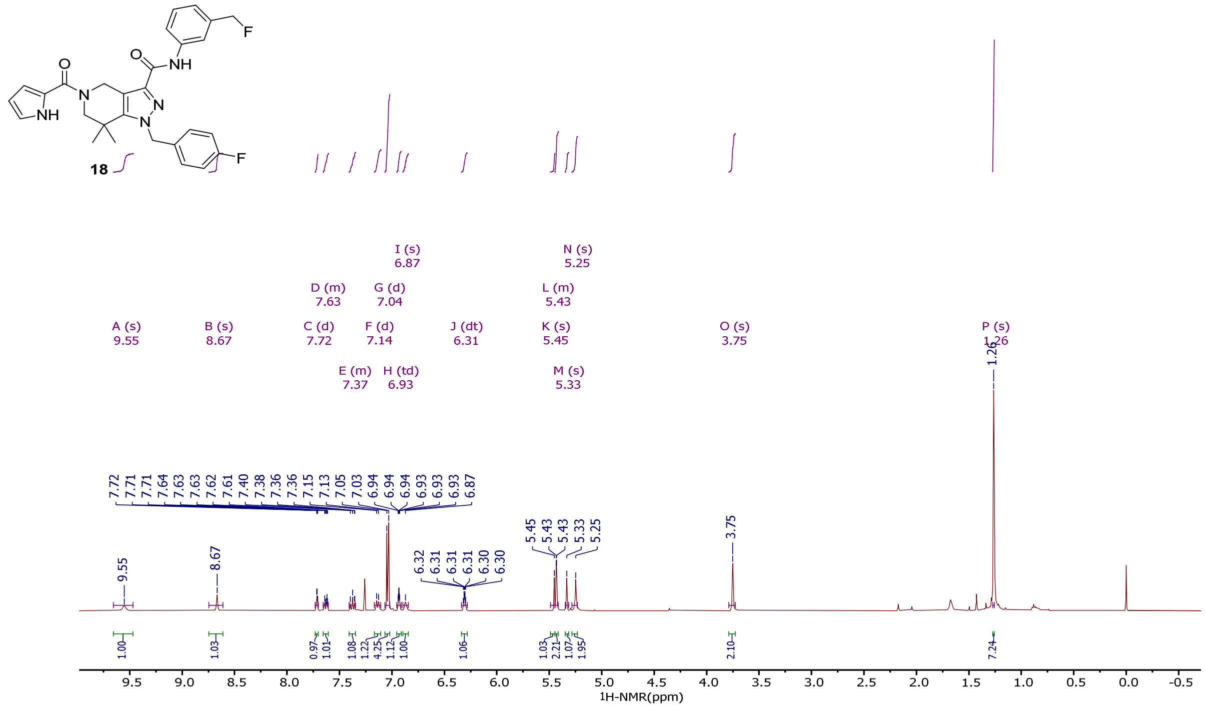
18
A (s) 9.55
B (s) 8.67
C (d) 7.72
D (m) 7.63
E (m) 7.37
F (d) 7.14
G (d) 7.04
H (td) 6.93
I (s) 6.87
J (dt) 6.31
K (s) 5.45
L (m) 5.43
M (s) 5.33
N (s) 5.25
O (s) 3.75
P (s) 1.26
1H-NMR(ppm)

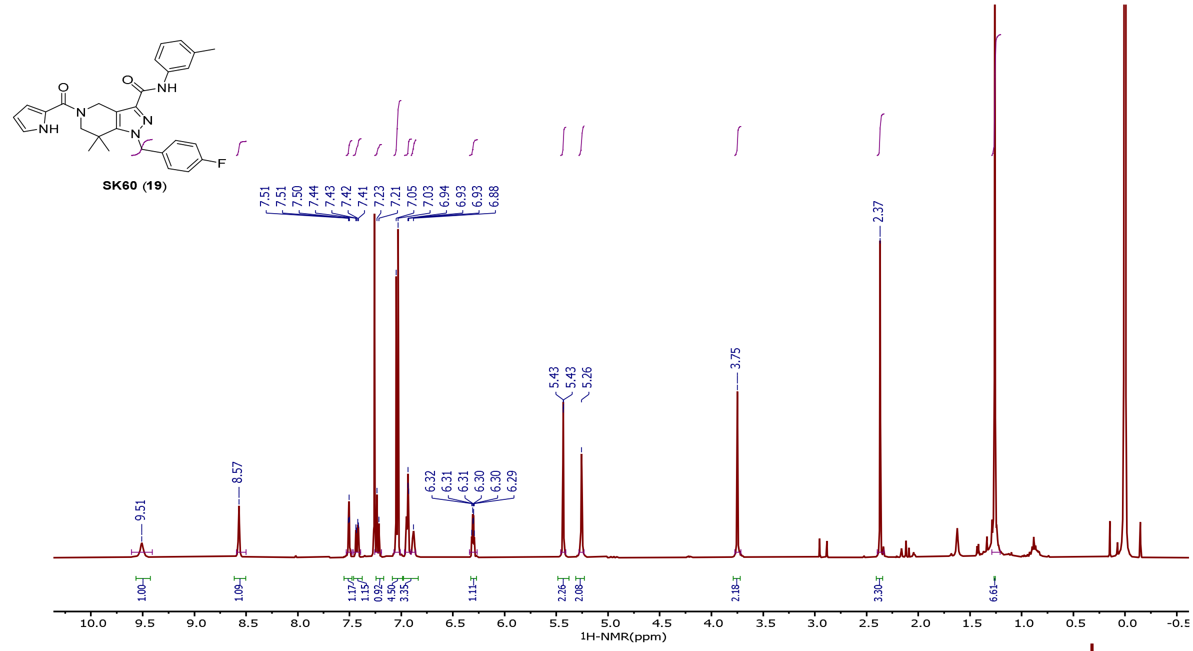

1H-NMR(ppm)
9.51
8.57
5.43
5.26
3.75
2.37

SK60 (19)

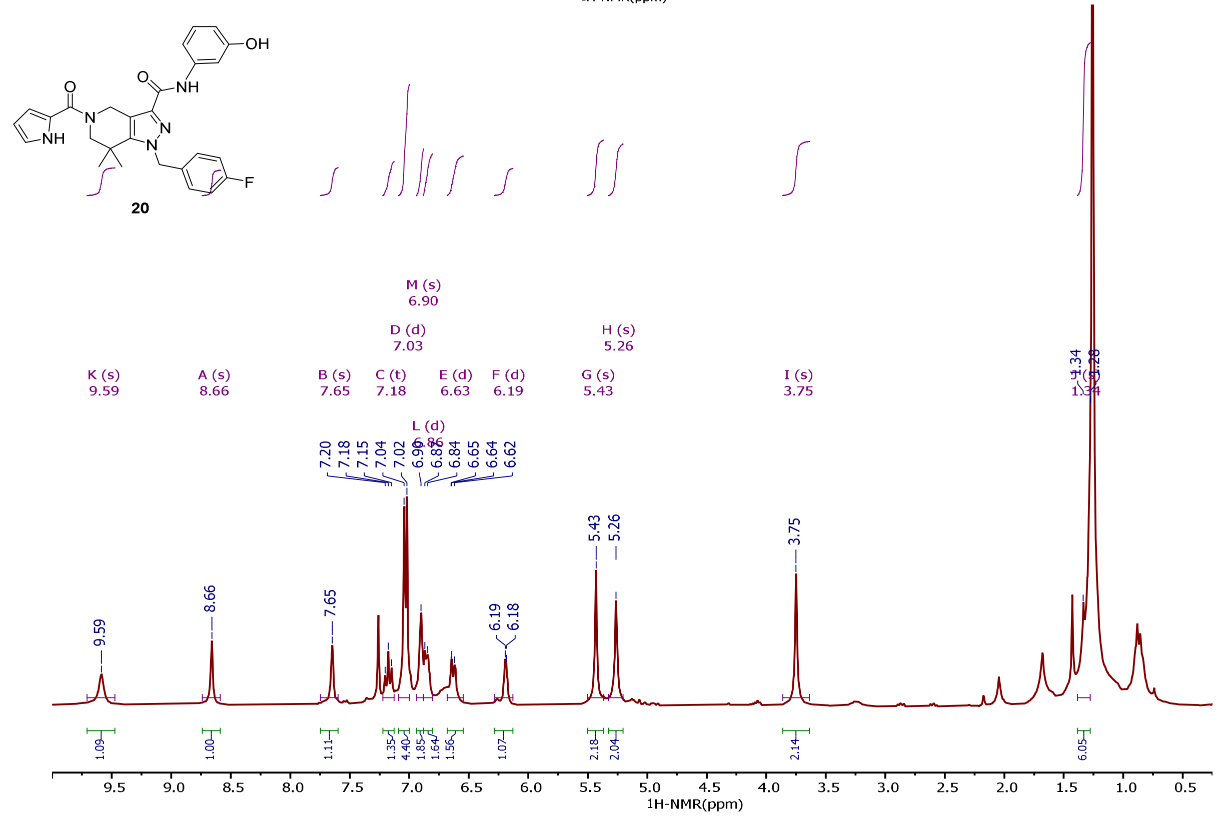

K (s)
9.59
A (s)
8.66
B (s)
7.65
C (t)
7.18
D (d)
7.03
M (s)
6.90
L (d)
E (d)
6.63
F (d)
6.19
G (s)
5.43
H (s)
5.26
I (s)
3.75
1H-NMR(ppm)

20

21

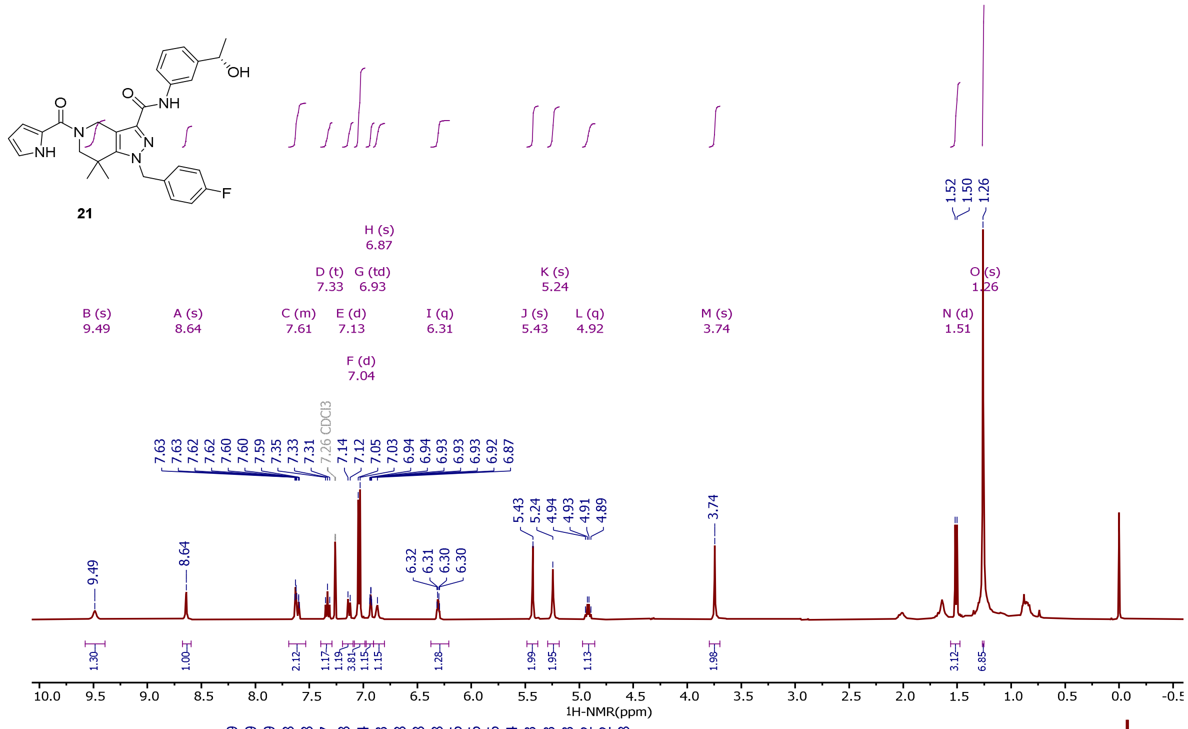

22

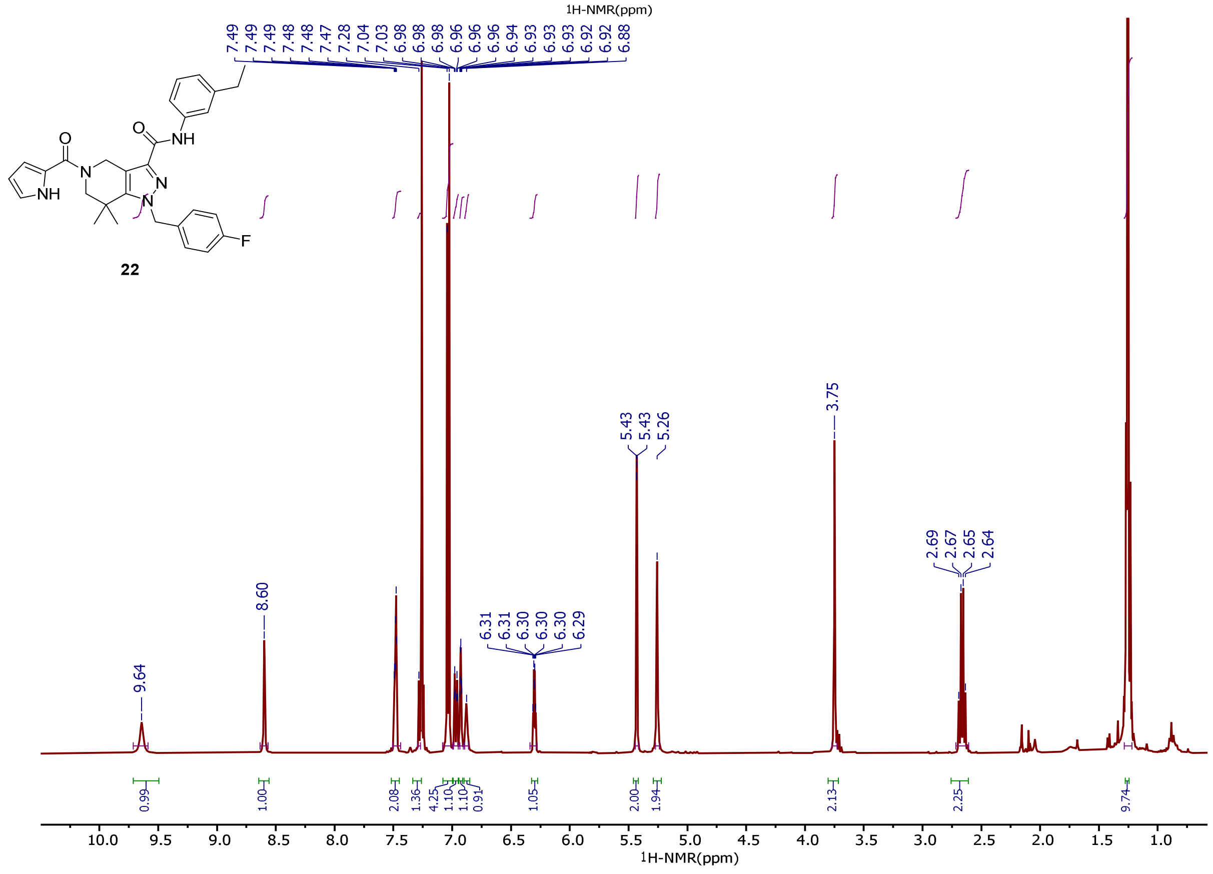

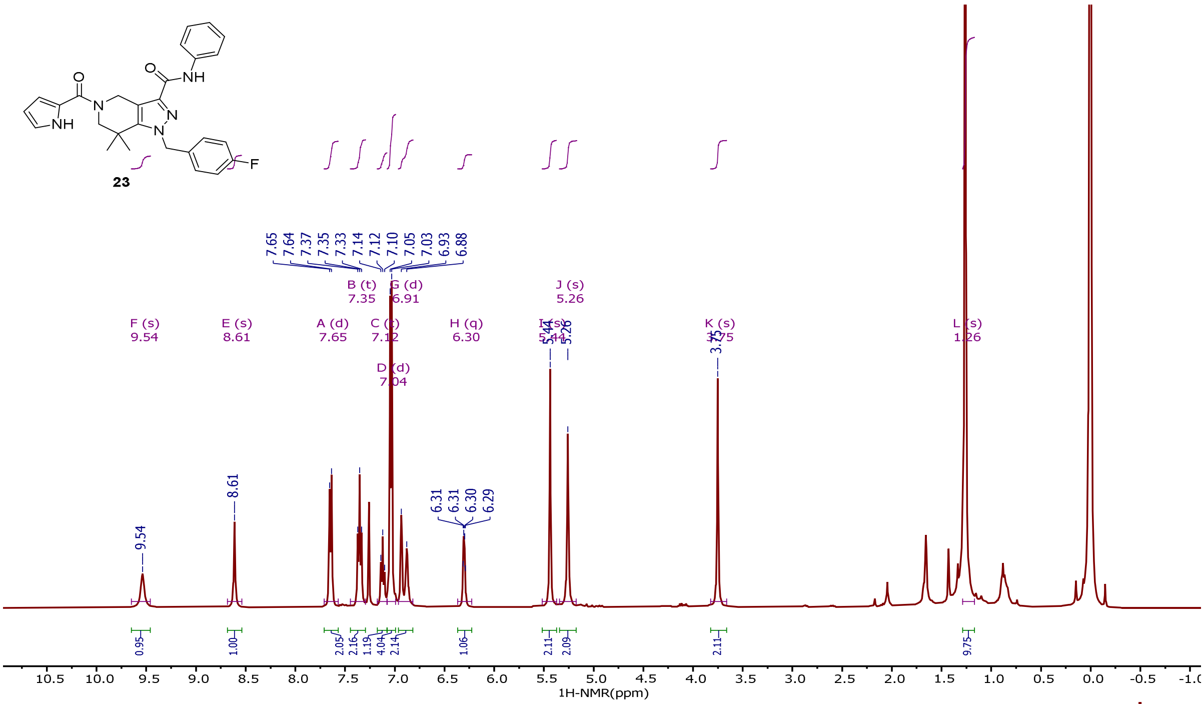

23
7.65 7.64 7.37 7.35 7.33 7.14 7.12 7.10 7.05 7.03 6.93 6.88
B (t) 7.35
G (d) 6.91
J (s) 5.26
F (s) 9.54
E (s) 8.61
A (d) 7.65
C (t) 7.12
H (q) 6.30
I (s) 5.44
K (s) 3.75
L (s) 1.26
D (d) 7.04
9.54
8.61
6.31 6.31 6.30 6.29
5.44
5.26
3.75
1H-NMR(ppm)

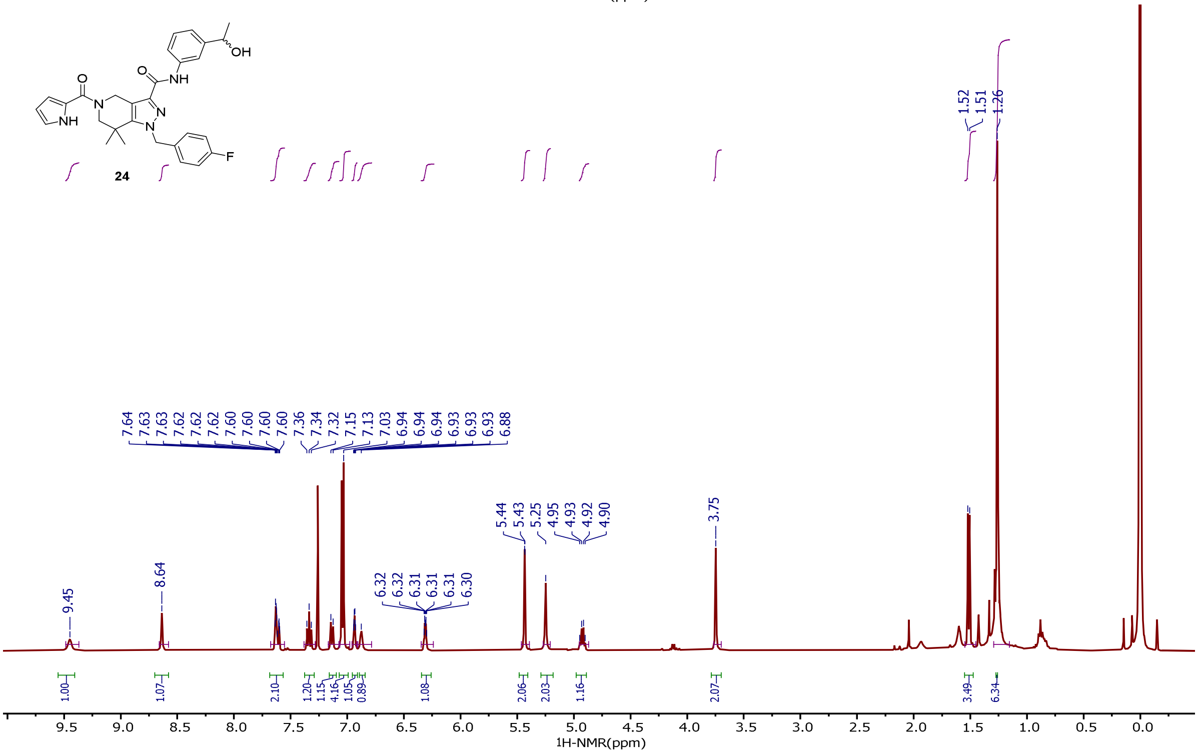

24
1.52 1.51 1.26
7.64 7.63 7.63 7.62 7.62 7.62 7.60 7.60 7.60 7.60 7.36 7.34 7.32 7.15 7.13 7.03 6.94 6.94 6.94 6.93 6.93 6.93 6.88
5.44 5.43 5.25 4.95 4.93 4.92 4.90
3.75
9.45
8.64
6.32 6.32 6.31 6.31 6.31 6.31 6.30
1H-NMR(ppm)

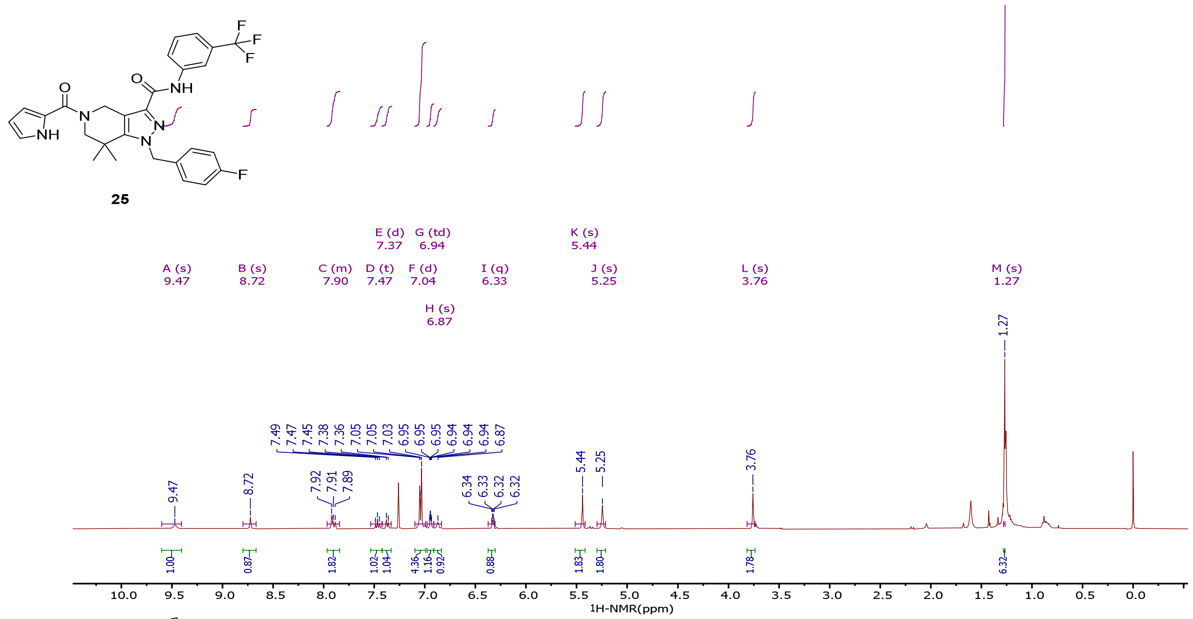
A (s) 9.47
B (s) 8.72
C (m) 7.90
D (t) 7.47
E (d) 7.37
F (d) 7.04
G (td) 6.94
H (s) 6.87
I (q) 6.33
K (s) 5.44
J (s) 5.25
L (s) 3.76
M (s) 1.27
1H-NMR(ppm)

25

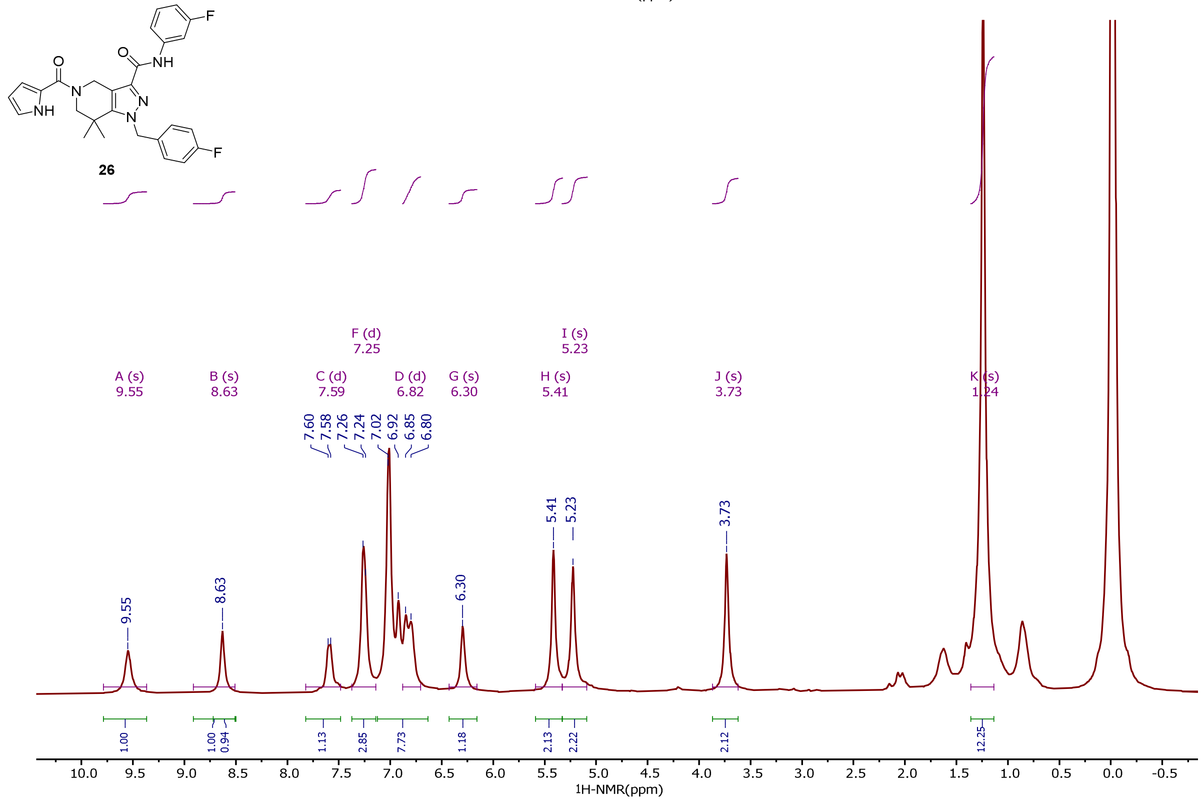
A (s) 9.55
B (s) 8.63
C (d) 7.59
F (d) 7.25
D (d) 6.82
G (s) 6.30
H (s) 5.41
I (s) 5.23
J (s) 3.73
K (s) 1.24
1H-NMR(ppm)

26

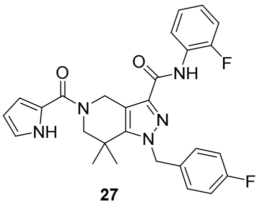
O
NH
F
N
N
N
NH
F

27

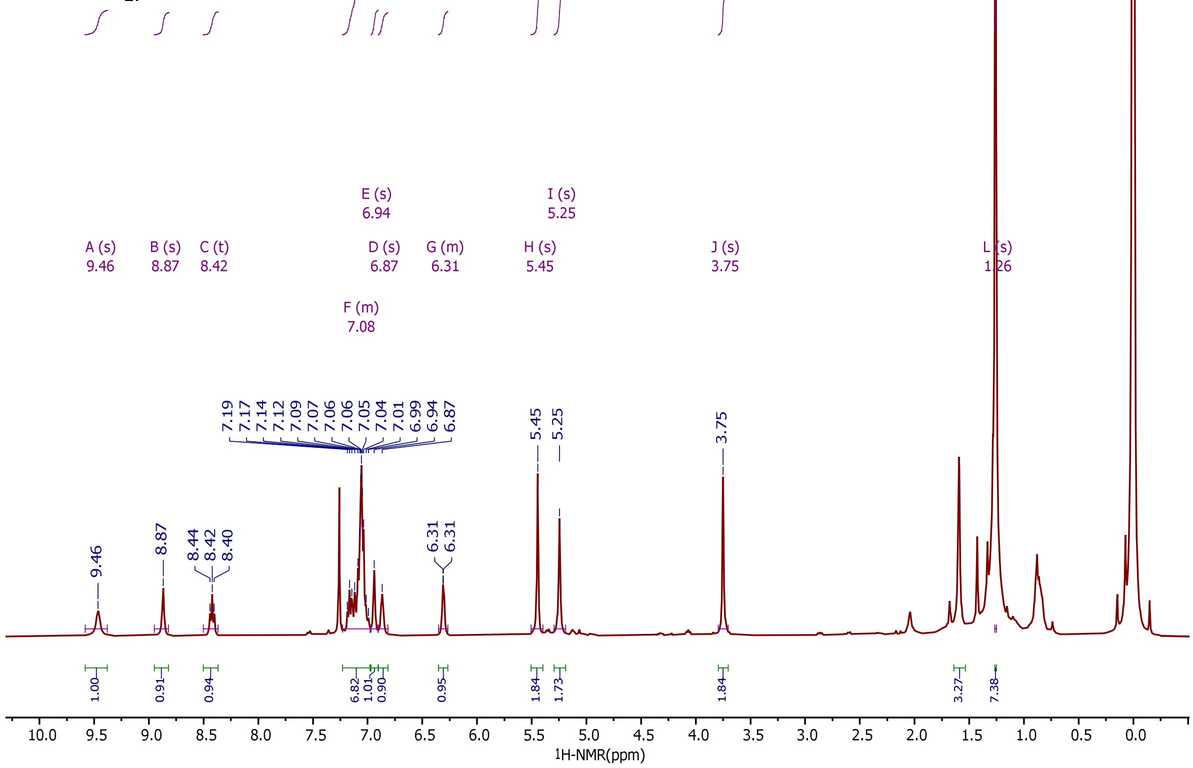
A (s) 9.46
B (s) 8.87
C (t) 8.42
E (s) 6.94
D (s) 6.87
F (m) 7.08
G (m) 6.31
I (s) 5.25
H (s) 5.45
J (s) 3.75
L (s) 1.26
1H-NMR(ppm)

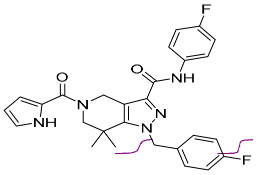
F
O
NH
O
N
N
N
NH
F

28

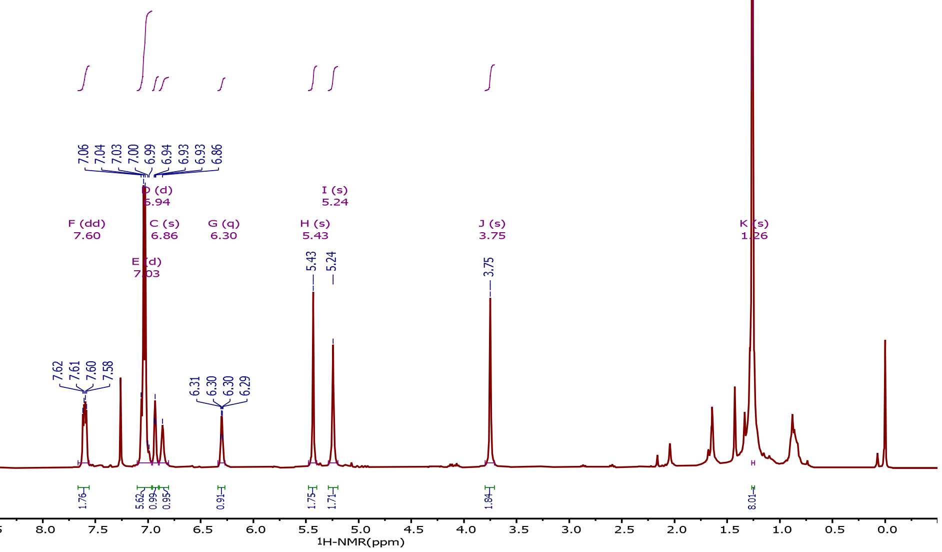
A (s) 9.52
B (s) 8.58
F (dd) 7.60
D (d) 6.94
C (s) 6.86
E (d) 7.03
G (q) 6.30
I (s) 5.24
H (s) 5.43
J (s) 3.75
K (s) 1.26
1H-NMR(ppm)

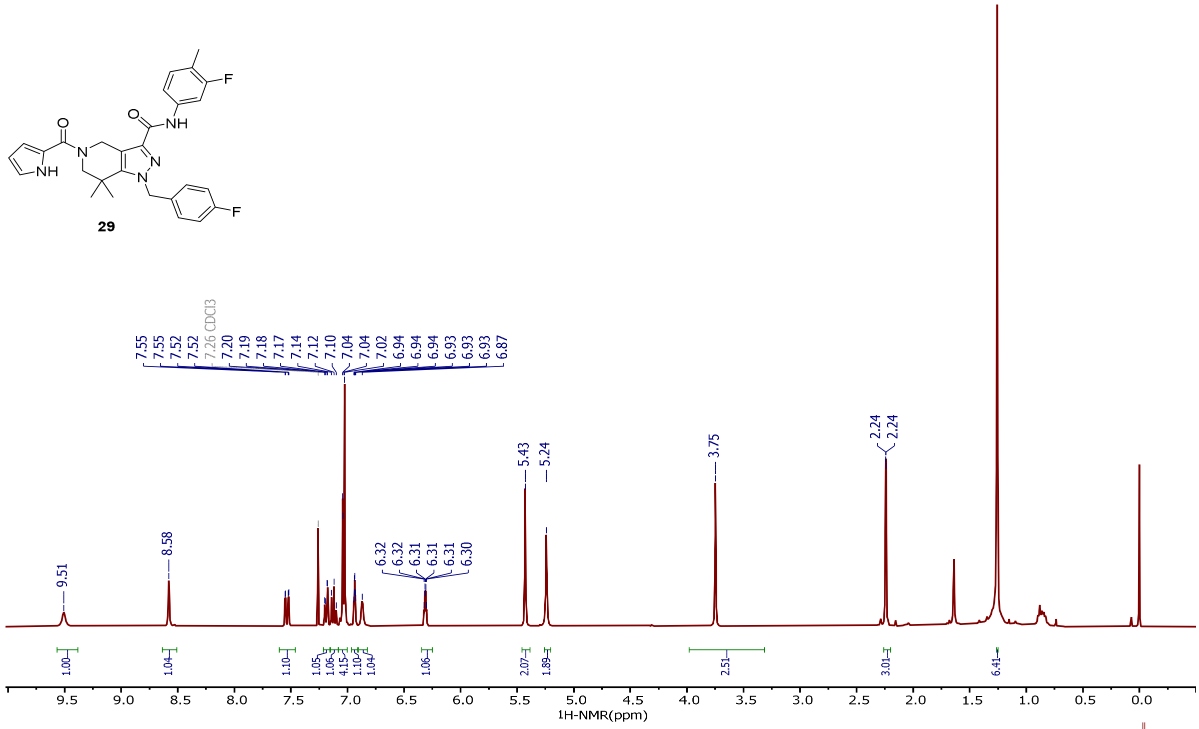
29
7.26 CDCl3
1H-NMR(ppm)

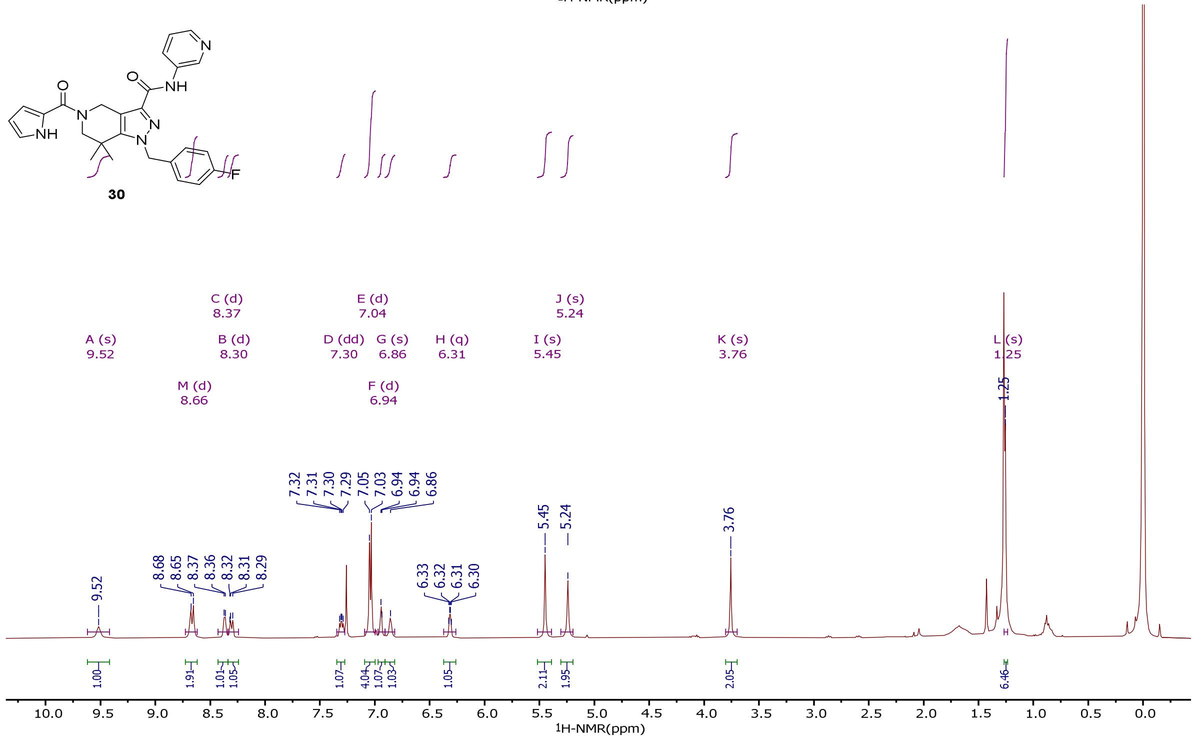
30
A (s) 9.52
B (d) 8.30
C (d) 8.37
D (dd) 7.30
E (d) 7.04
F (d) 6.94
G (s) 6.86
H (q) 6.31
I (s) 5.45
J (s) 5.24
K (s) 3.76
L (s) 1.25
M (d) 8.66
1H-NMR(ppm)

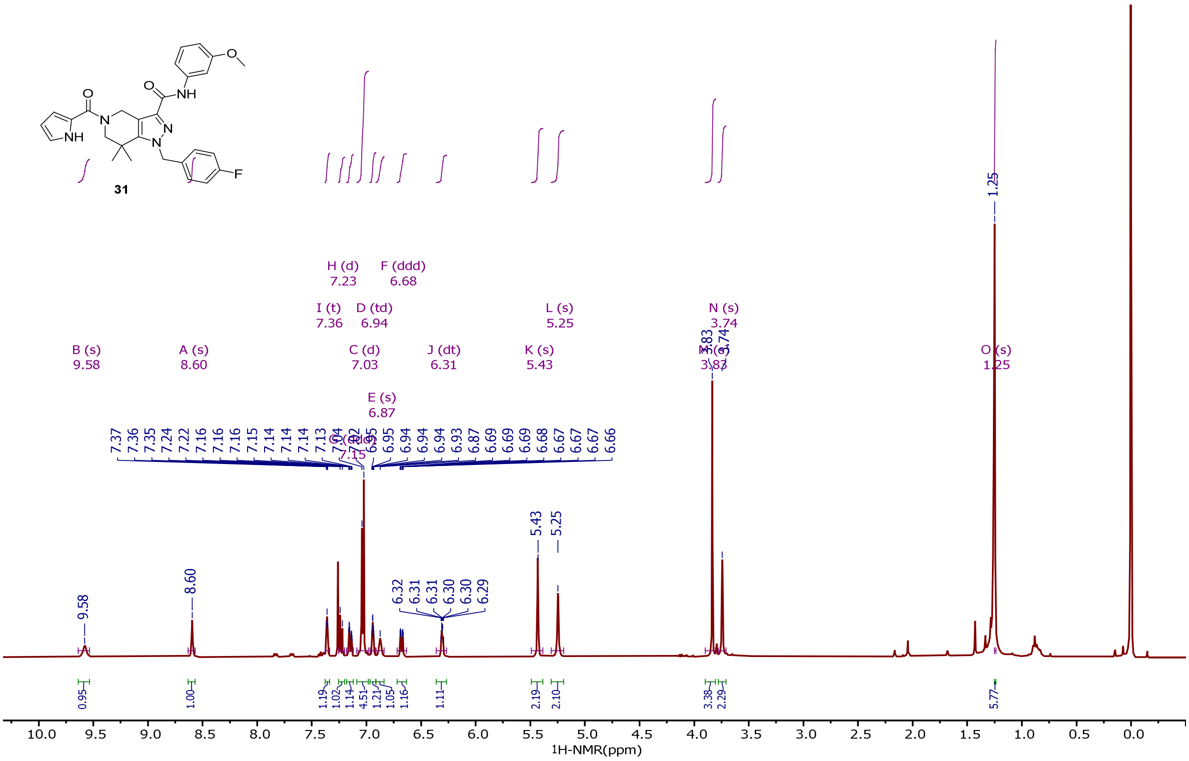
31
H (d) 7.23
F (ddd) 6.68
I (t) 7.36
D (td) 6.94
L (s) 5.25
N (s) 3.74
B (s) 9.58
A (s) 8.60
C (d) 7.03
J (dt) 6.31
K (s) 5.43
O (s) 1.25
E (s) 6.87
1H-NMR(ppm)

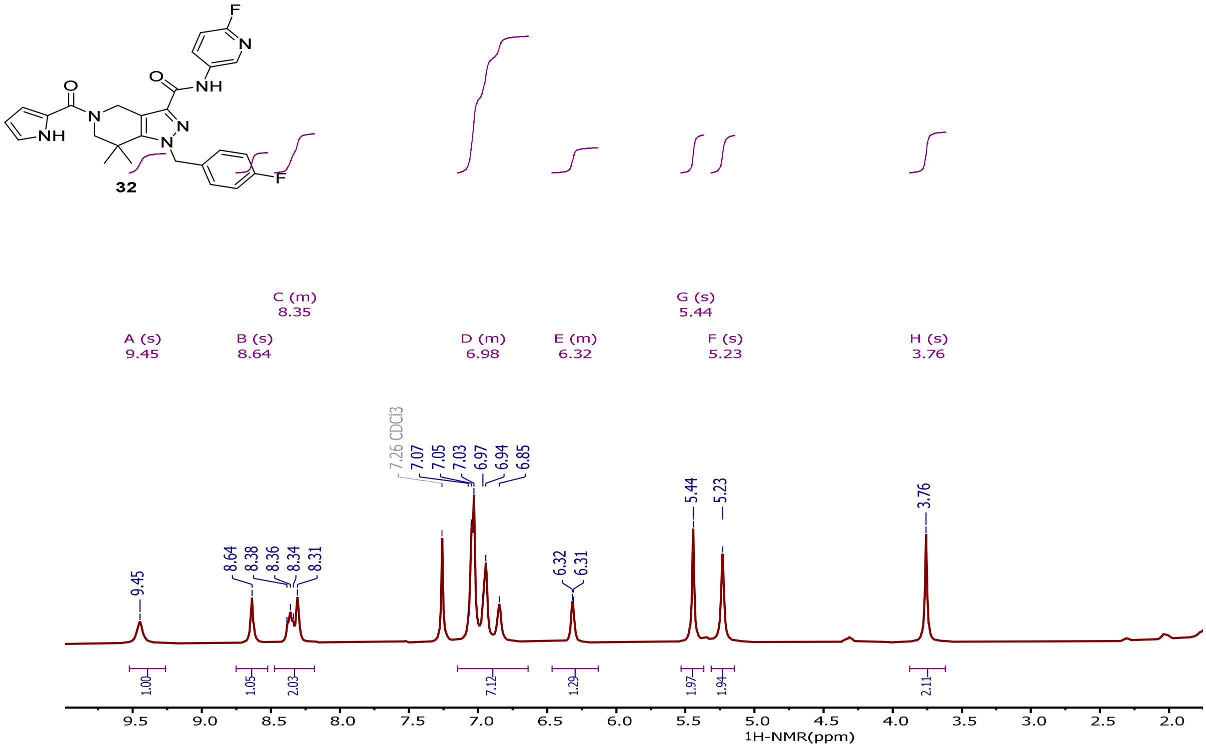
32
C (m) 8.35
G (s) 5.44
A (s) 9.45
B (s) 8.64
D (m) 6.98
E (m) 6.32
F (s) 5.23
H (s) 3.76
7.26 CDCl3
1H-NMR(ppm)

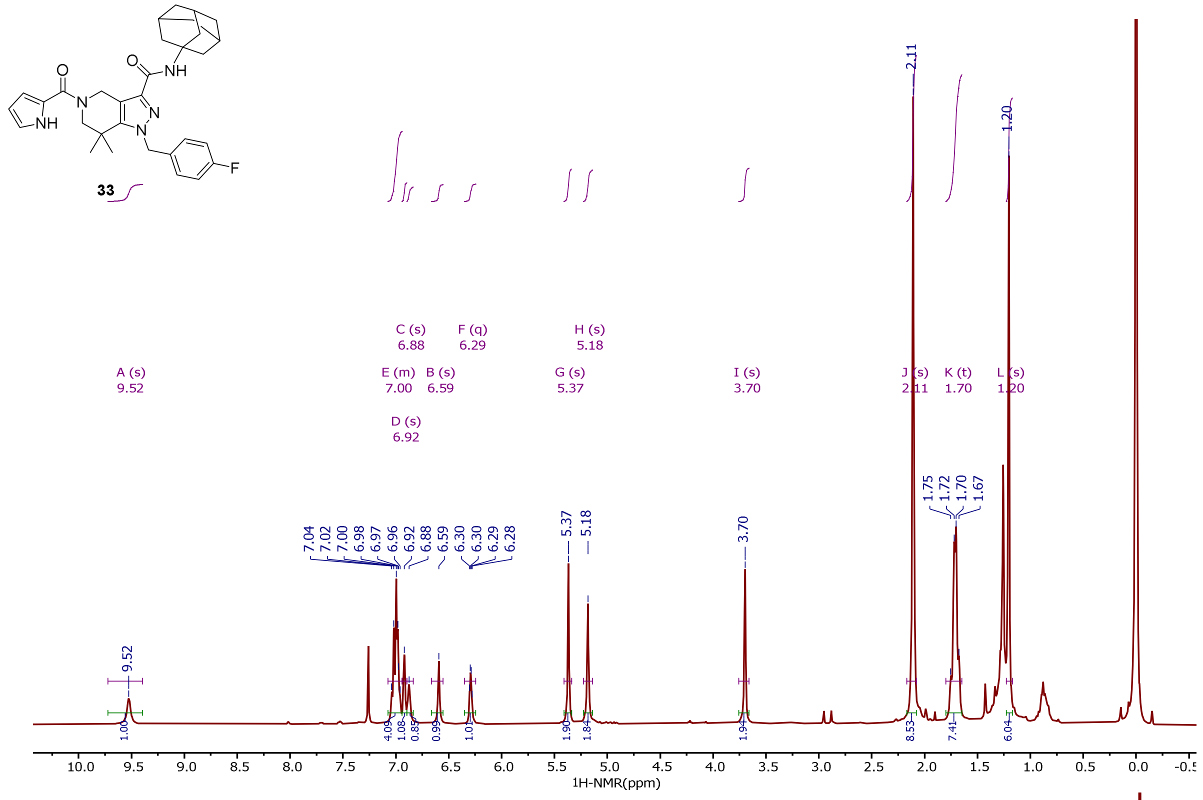
33
A (s) 9.52
B (s) 6.59
C (s) 6.88
D (s) 6.92
E (m) 7.00
F (q) 6.29
G (s) 5.37
H (s) 5.18
I (s) 3.70
J (s) 2.11
K (t) 1.70
L (s) 1.20
1H-NMR(ppm)

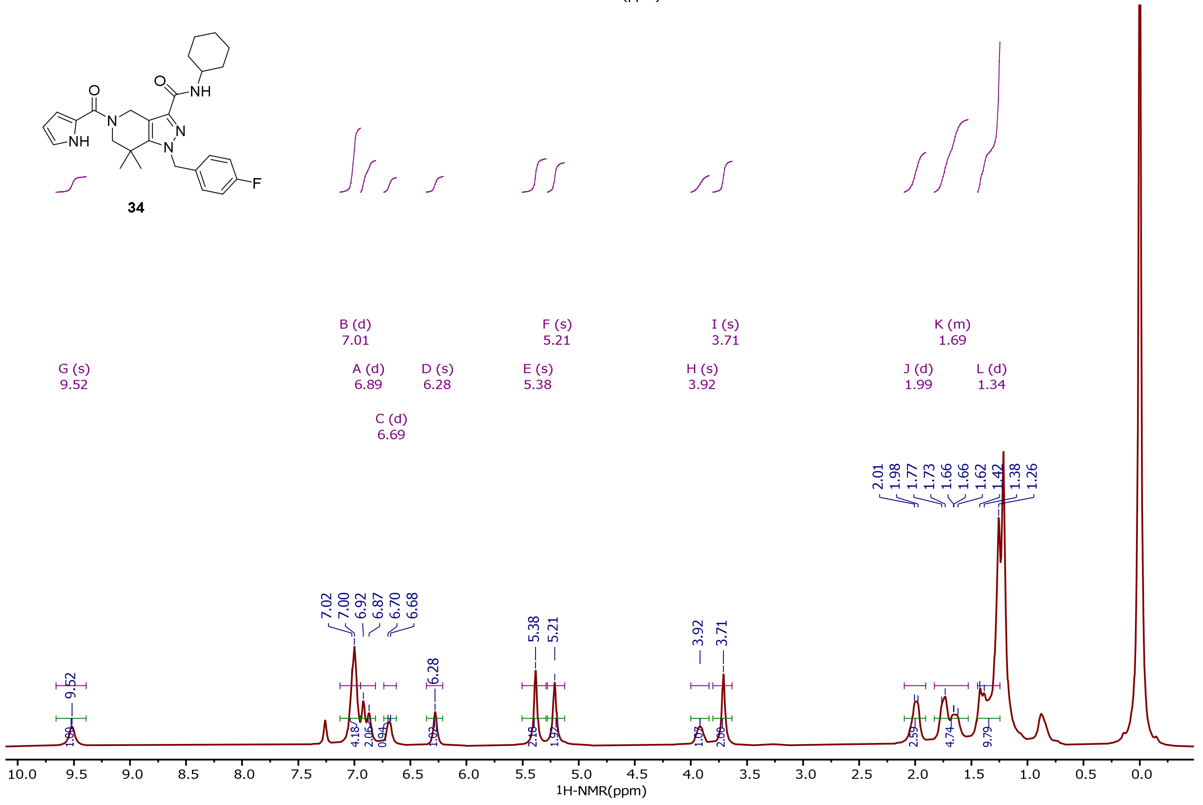
34
A (d) 6.89
B (d) 7.01
C (d) 6.69
D (s) 6.28
E (s) 5.38
F (s) 5.21
G (s) 9.52
H (s) 3.92
I (s) 3.71
J (d) 1.99
K (m) 1.69
L (d) 1.34
1H-NMR(ppm)

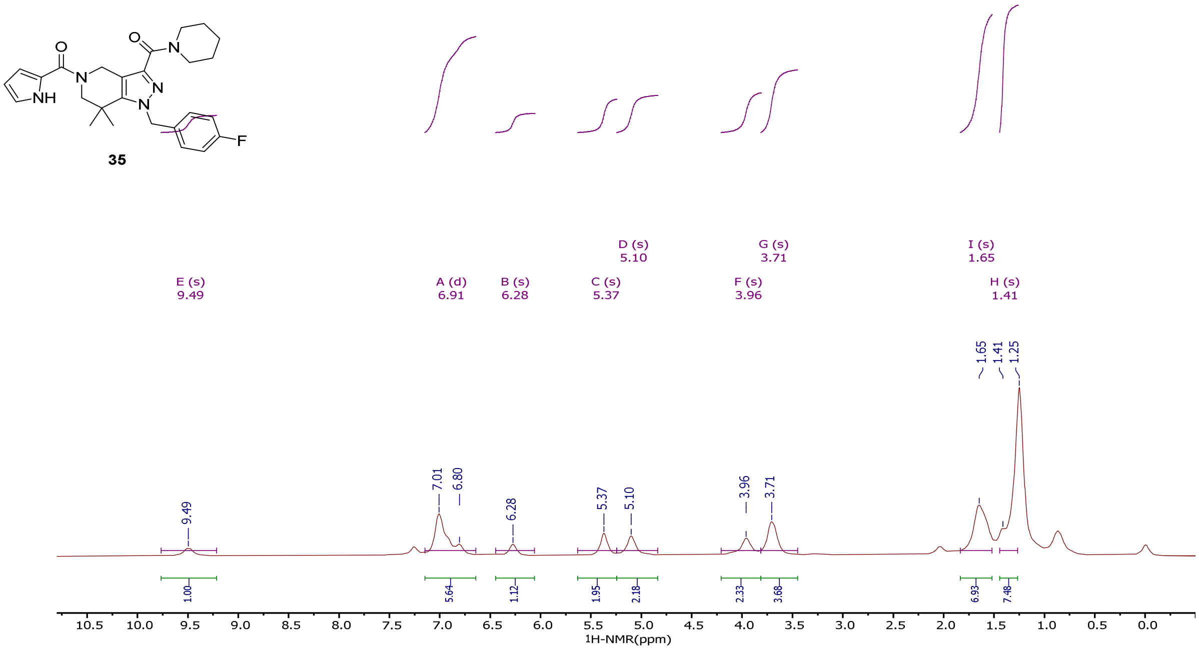
O
N
NH
N
N
N
F
D (s) 5.10
G (s) 3.71
I (s) 1.65
E (s) 9.49
A (d) 6.91
B (s) 6.28
C (s) 5.37
F (s) 3.96
H (s) 1.41
9.49
7.01
6.80
6.28
5.37
5.10
3.96
3.71
1.65
1.41
1.25
1.00
5.64
1.12
1.95
2.18
2.33
3.68
6.93
7.48
1H-NMR(ppm)

35

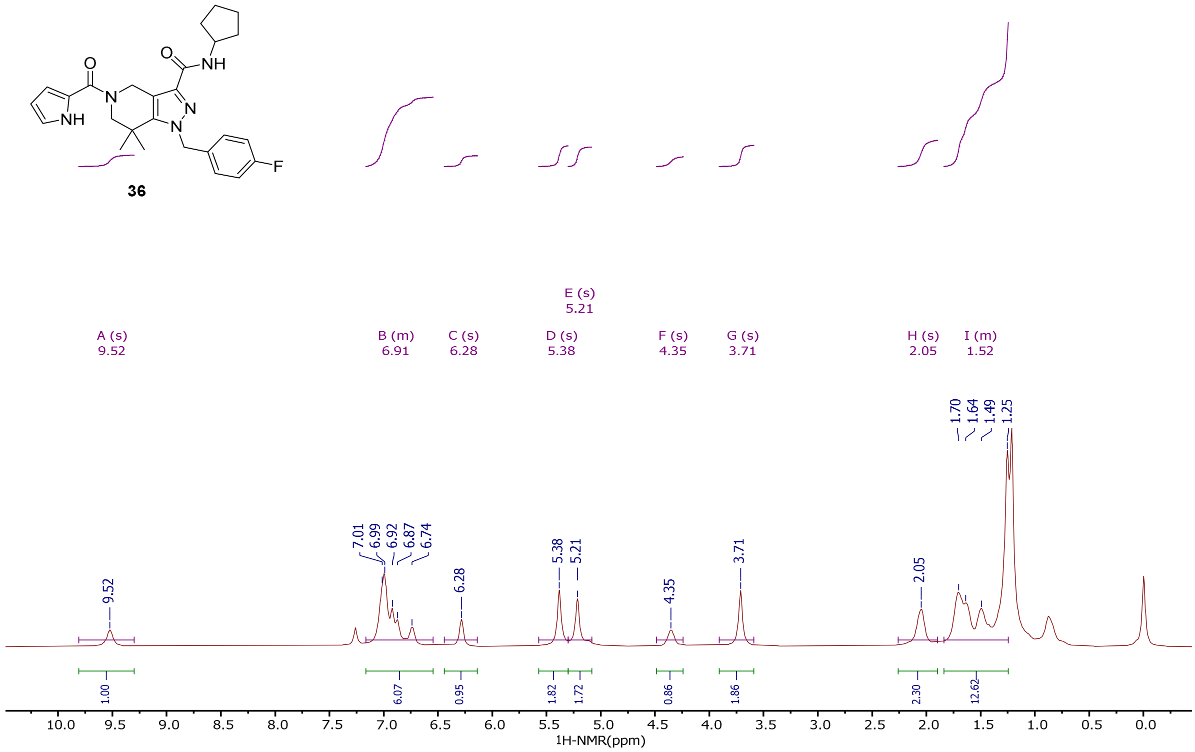
O
NH
N
NH
N
N
F
E (s) 5.21
A (s) 9.52
B (m) 6.91
C (s) 6.28
D (s) 5.38
F (s) 4.35
G (s) 3.71
H (s) 2.05
I (m) 1.52
9.52
7.01
6.99
6.92
6.87
6.74
6.28
5.38
5.21
4.35
3.71
2.05
1.70
1.64
1.49
1.25
1.00
6.07
0.95
1.82
1.72
0.86
1.86
2.30
12.62
1H-NMR(ppm)

36

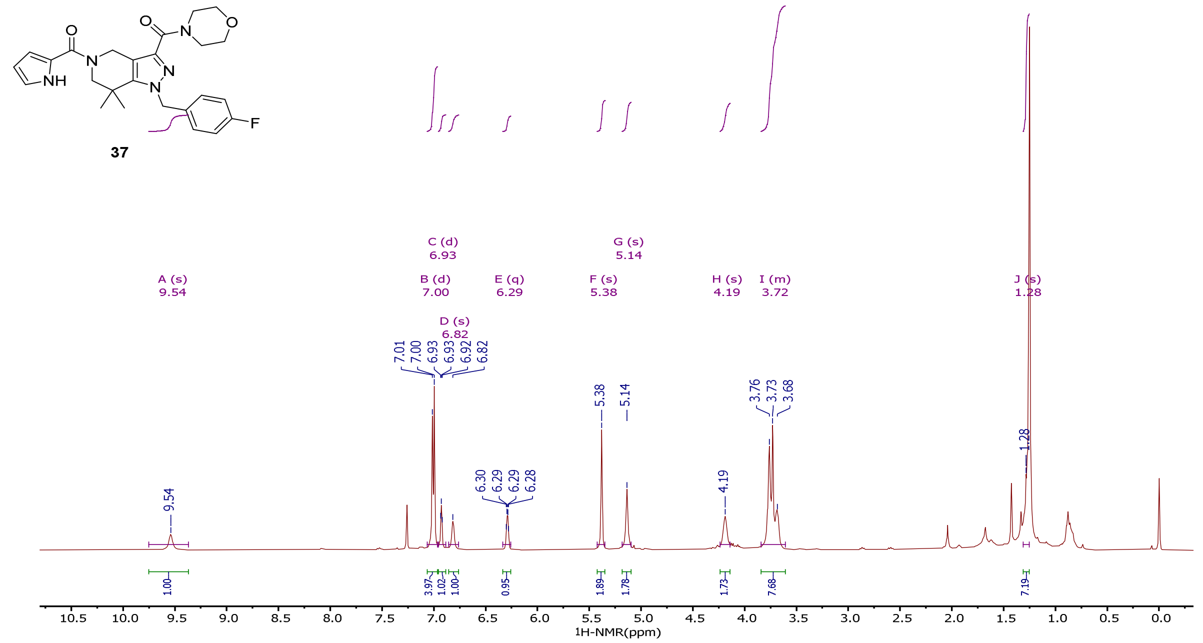
A (s) 9.54
B (d) 7.00
C (d) 6.93
D (s) 6.82
E (q) 6.29
F (s) 5.38
G (s) 5.14
H (s) 4.19
I (m) 3.72
J (s) 1.28
1H-NMR(ppm)
37

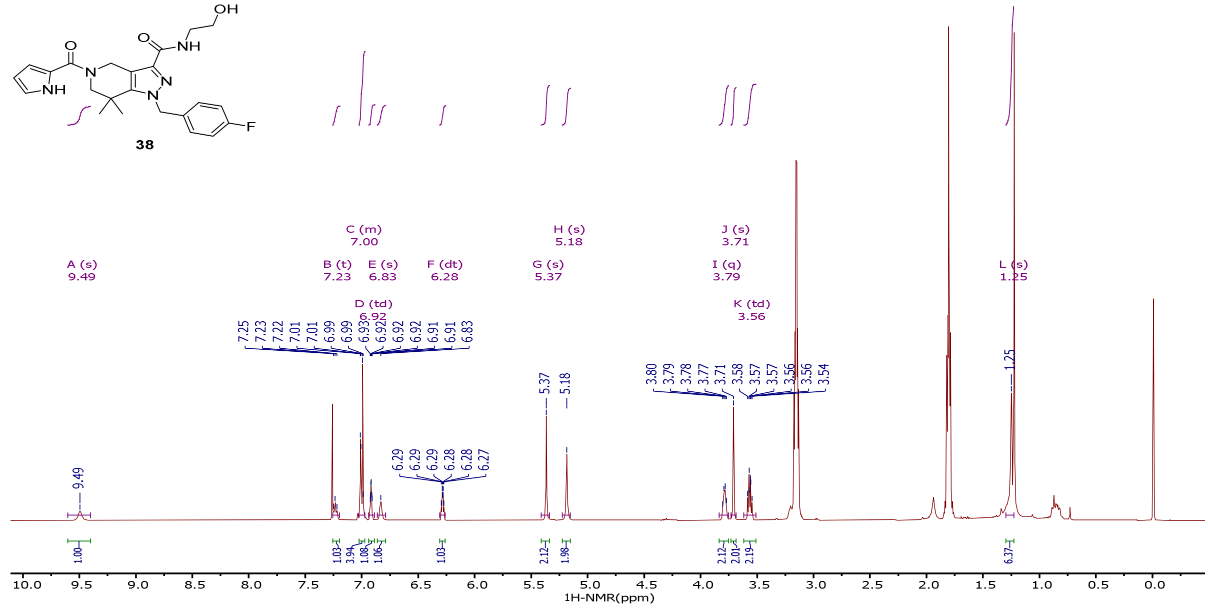
A (s) 9.49
B (t) 7.23
C (m) 7.00
D (td) 6.92
E (s) 6.83
F (dt) 6.28
G (s) 5.37
H (s) 5.18
I (q) 3.79
J (s) 3.71
K (td) 3.56
L (s) 1.25
1H-NMR(ppm)
38

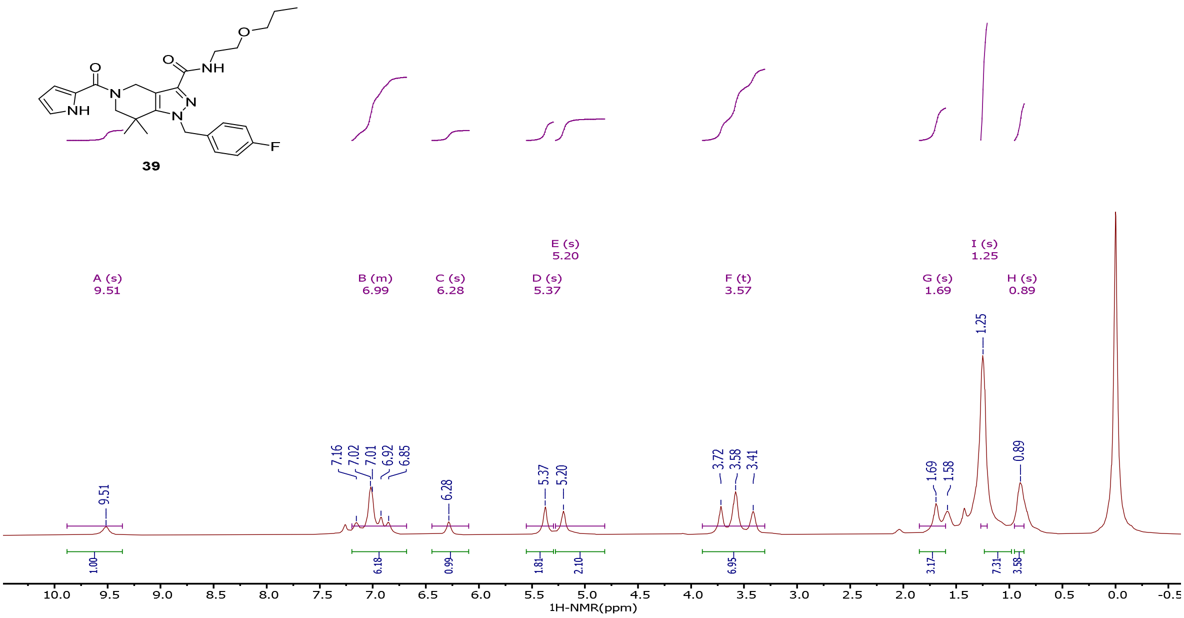
39
1H-NMR(ppm)

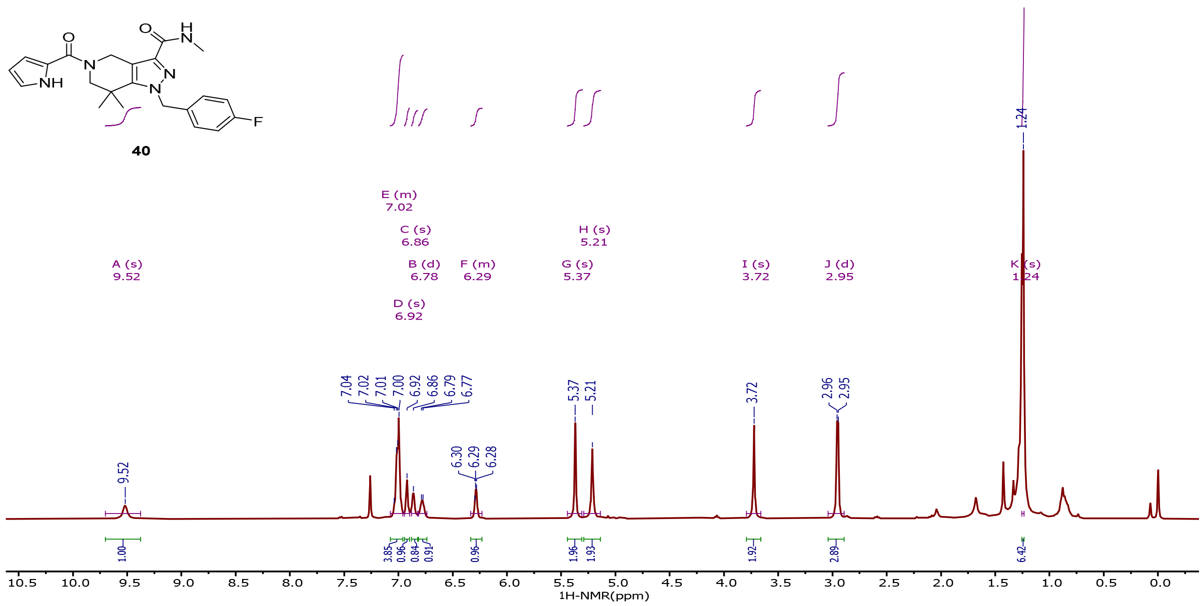
40
1H-NMR(ppm)

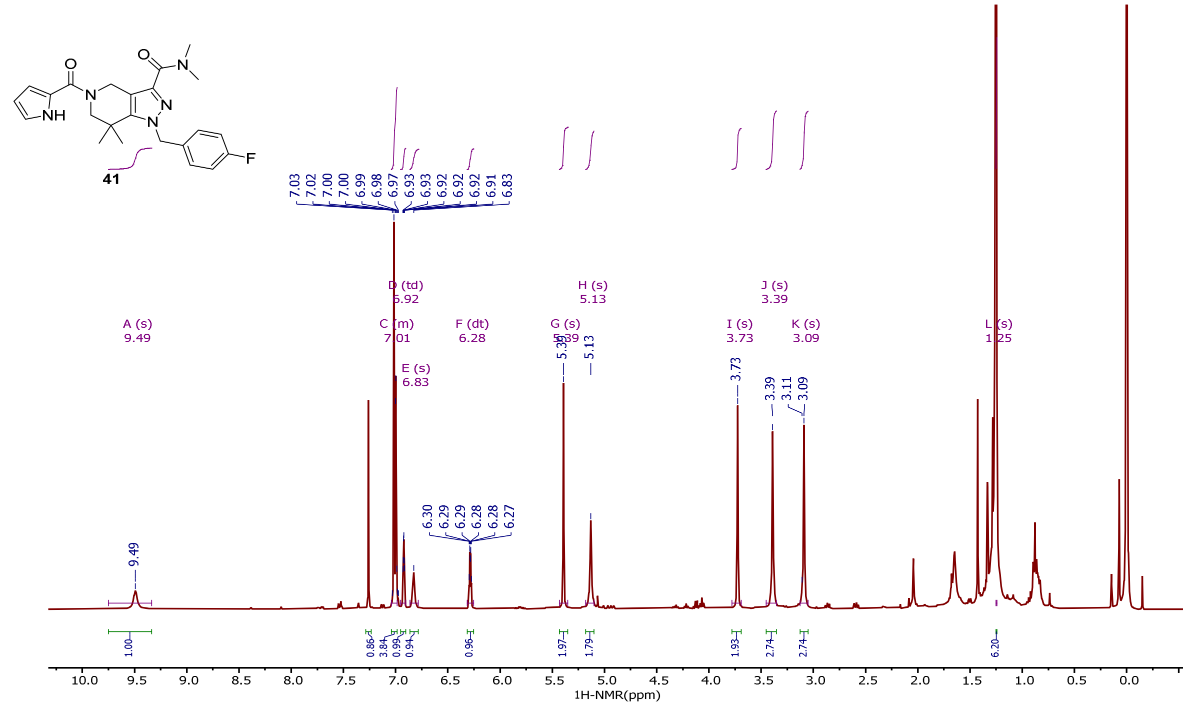
41
A (s) 9.49
C (m) 7.01
D (td) 6.92
E (s) 6.83
F (dt) 6.28
G (s) 5.39
H (s) 5.13
I (s) 3.73
J (s) 3.39
K (s) 3.09
L (s) 1.25
1H-NMR(ppm)

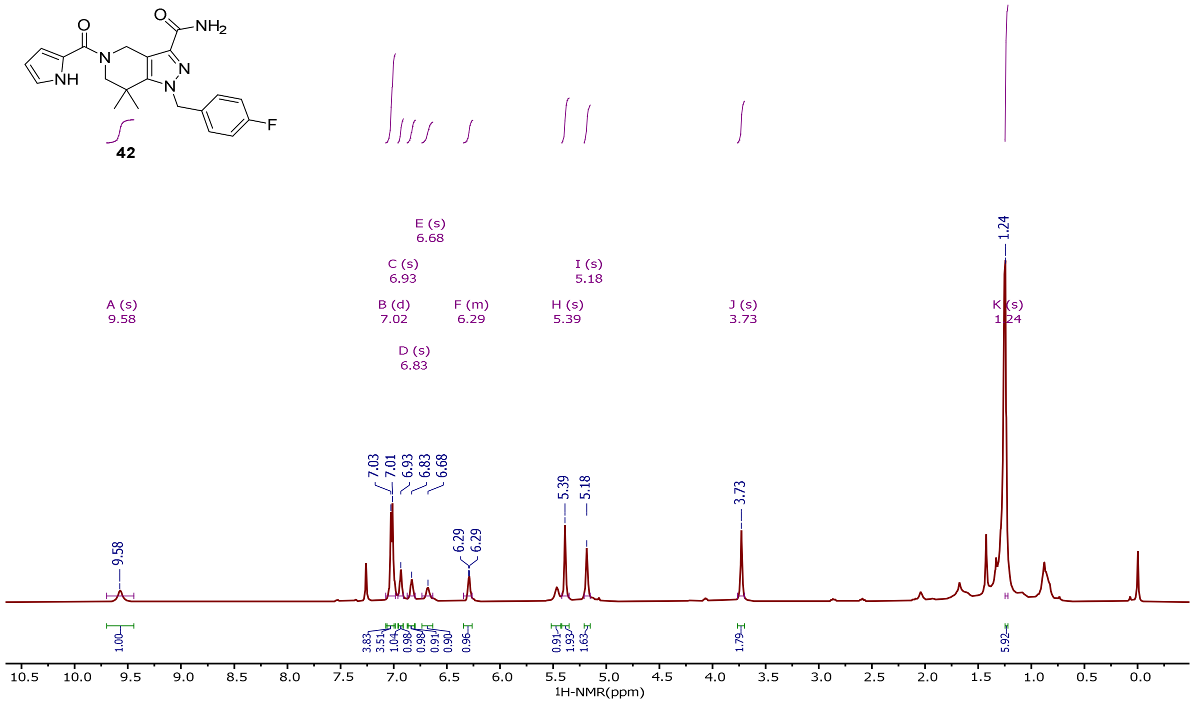
42
A (s) 9.58
B (d) 7.02
C (s) 6.93
D (s) 6.83
E (s) 6.68
F (m) 6.29
H (s) 5.39
I (s) 5.18
J (s) 3.73
K (s) 1.24
1H-NMR(ppm)

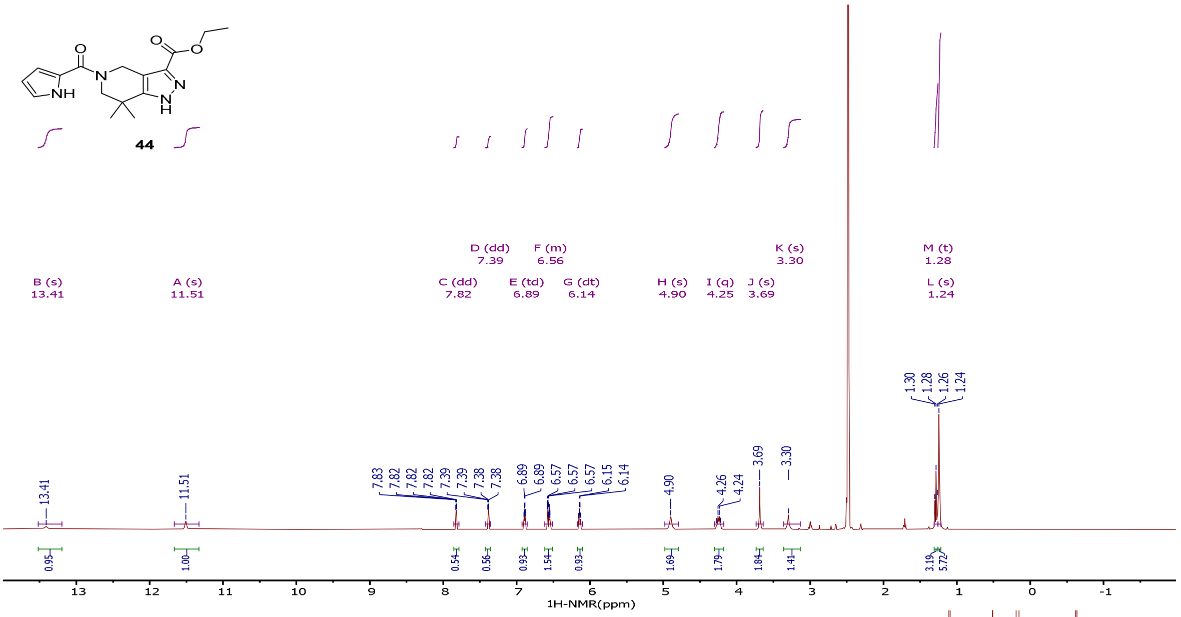
44
B (s) 13.41
A (s) 11.51
C (dd) 7.82
D (dd) 7.39
E (td) 6.89
F (m) 6.56
G (dt) 6.14
H (s) 4.90
I (q) 4.25
J (s) 3.69
K (s) 3.30
M (t) 1.28
L (s) 1.24
1H-NMR(ppm)

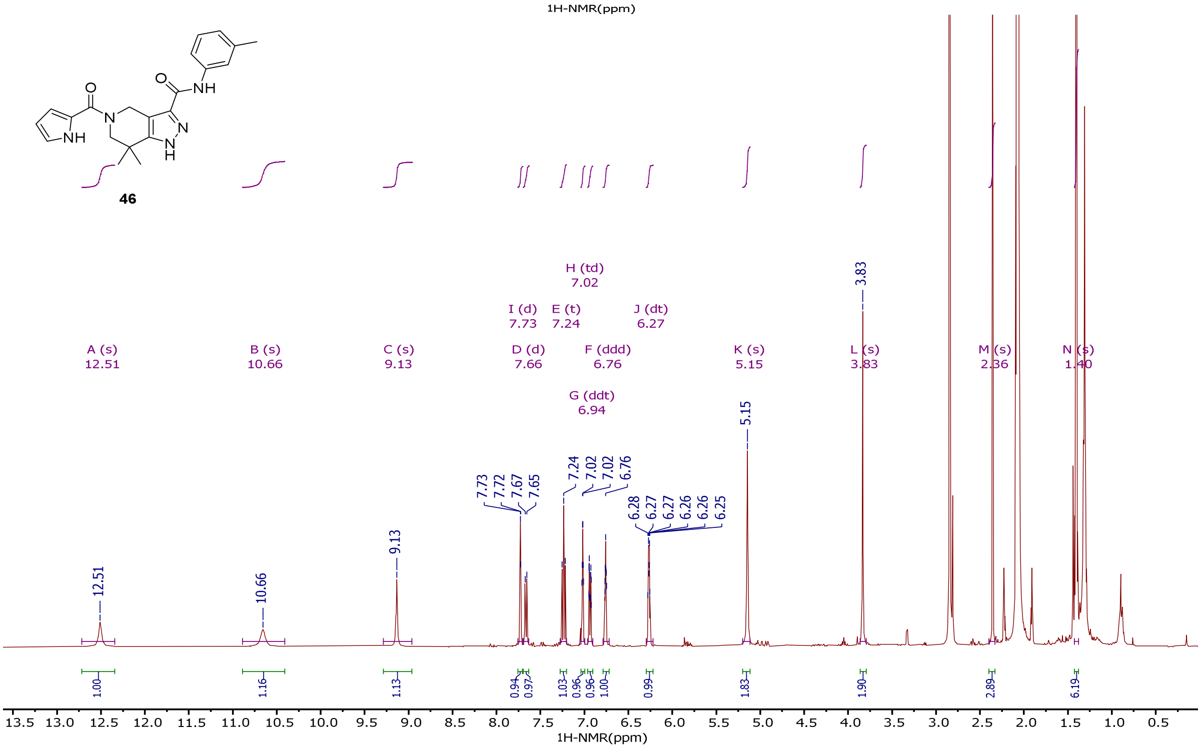
46
A (s) 12.51
B (s) 10.66
C (s) 9.13
I (d) 7.73
D (d) 7.66
E (t) 7.24
H (td) 7.02
G (ddt) 6.94
F (ddd) 6.76
J (dt) 6.27
K (s) 5.15
L (s) 3.83
M (s) 2.36
N (s) 1.40
1H-NMR(ppm)

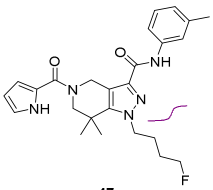

47

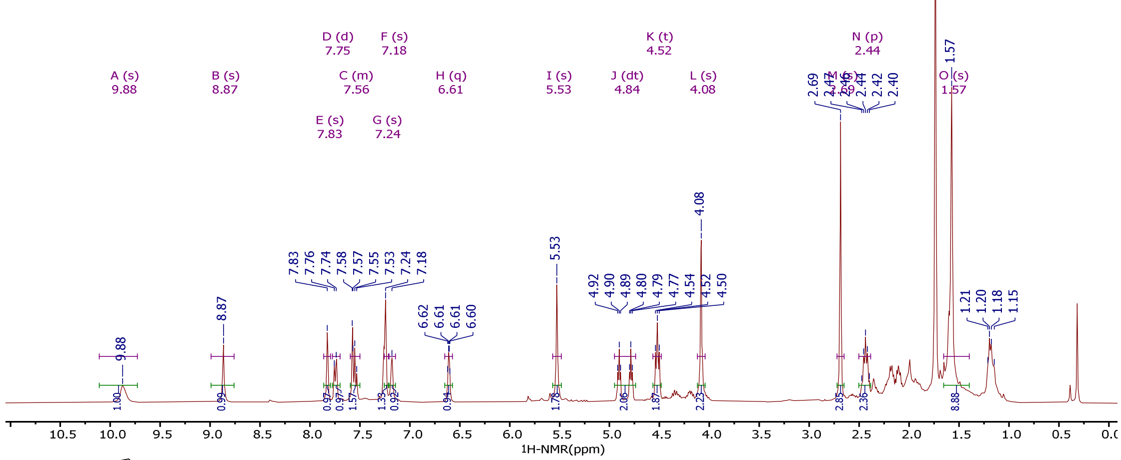
A (s) 9.88
B (s) 8.87
D (d) 7.75
C (m) 7.56
E (s) 7.83
F (s) 7.18
G (s) 7.24
H (q) 6.61
I (s) 5.53
J (dt) 4.84
K (t) 4.52
L (s) 4.08
N (p) 2.44
O (s) 1.57
1H-NMR(ppm)

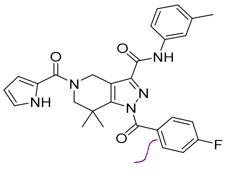

48

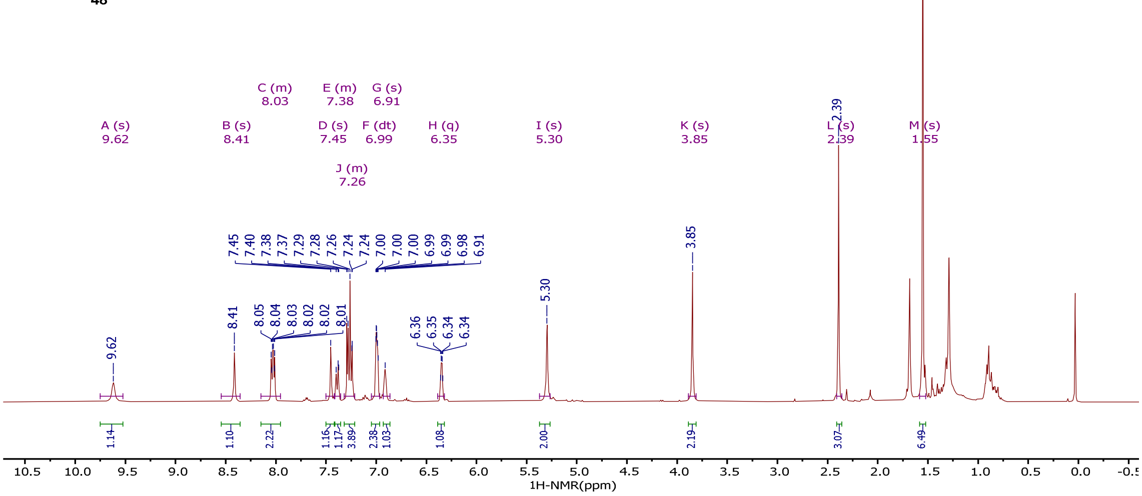
A (s) 9.62
B (s) 8.41
C (m) 8.03
D (s) 7.45
E (m) 7.38
F (dt) 6.99
G (s) 6.91
J (m) 7.26
H (q) 6.35
I (s) 5.30
K (s) 3.85
L (s) 2.39
M (s) 1.55
1H-NMR(ppm)

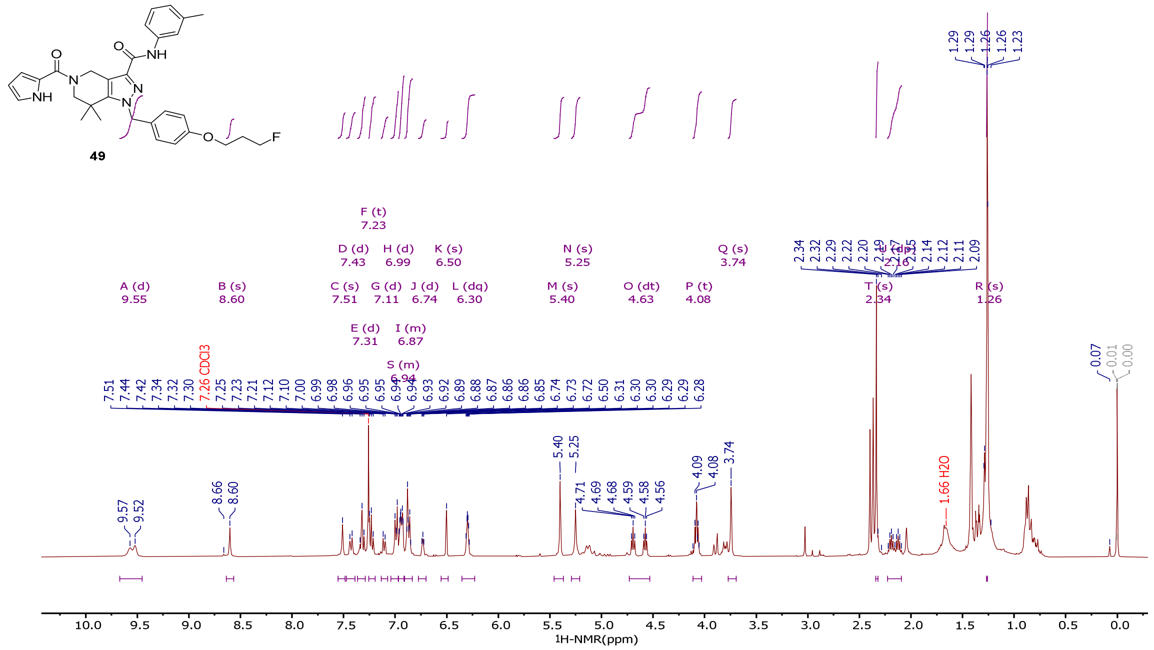

49

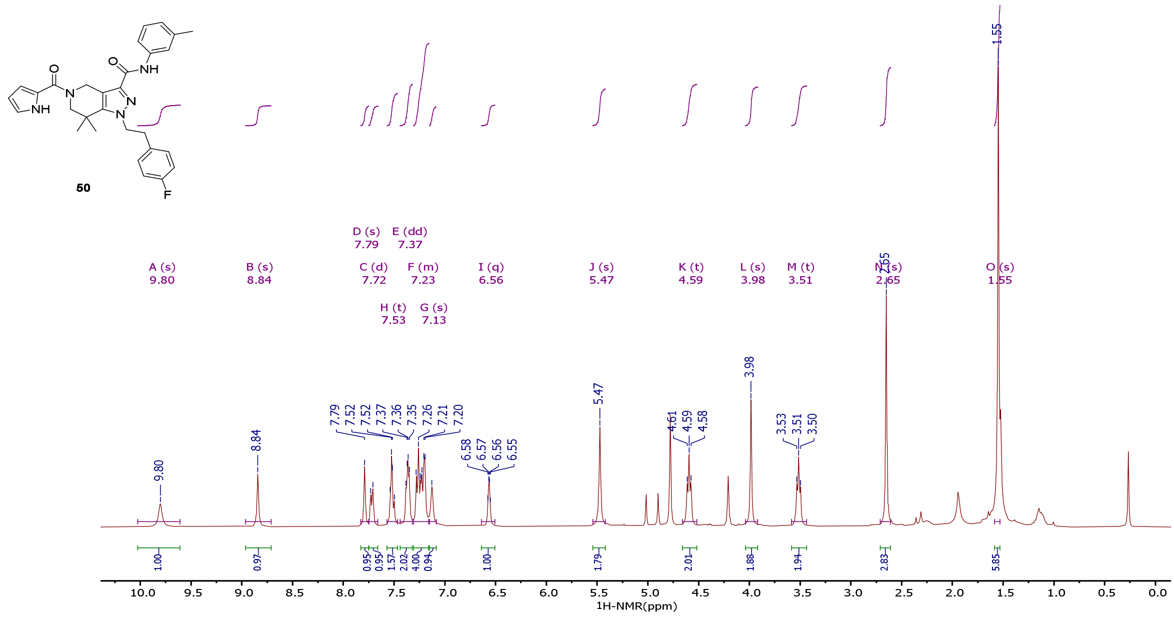

50

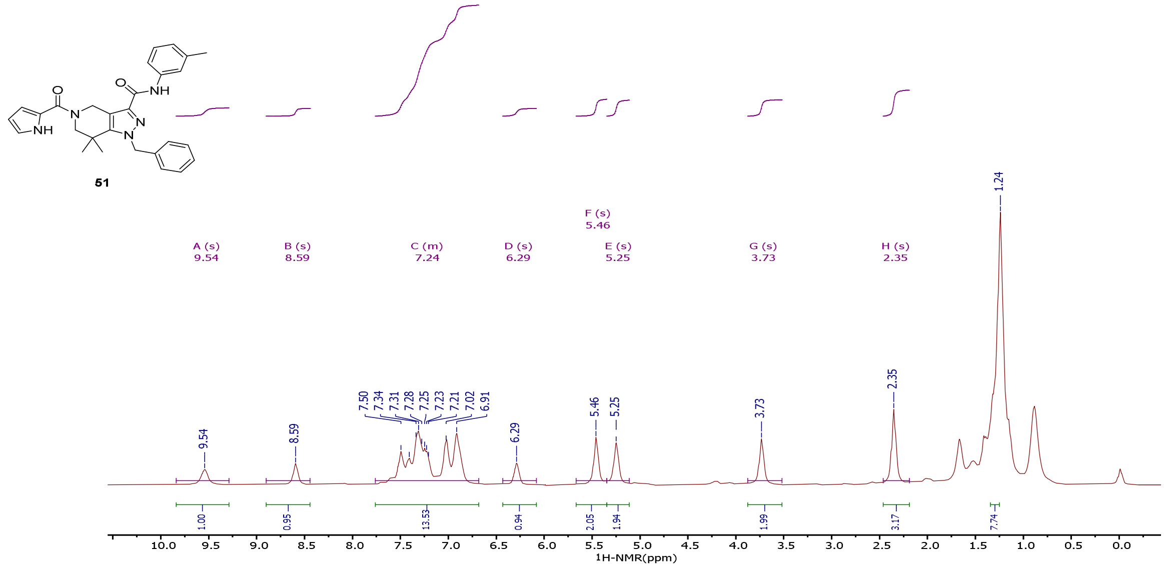
A (s) 9.54
B (s) 8.59
C (m) 7.24
D (s) 6.29
F (s) 5.46
E (s) 5.25
G (s) 3.73
H (s) 2.35
1H-NMR(ppm)

51

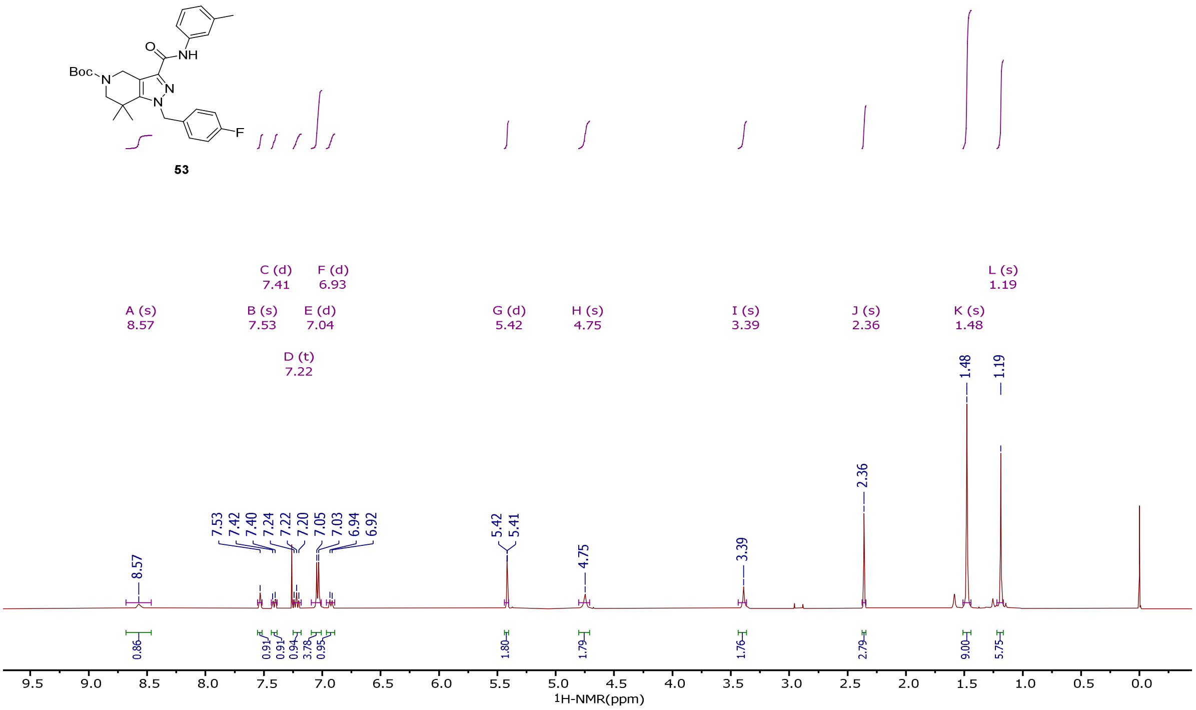
Boc
F
A (s) 8.57
B (s) 7.53
C (d) 7.41
D (t) 7.22
E (d) 7.04
F (d) 6.93
G (d) 5.42
H (s) 4.75
I (s) 3.39
J (s) 2.36
K (s) 1.48
L (s) 1.19
1H-NMR(ppm)

53

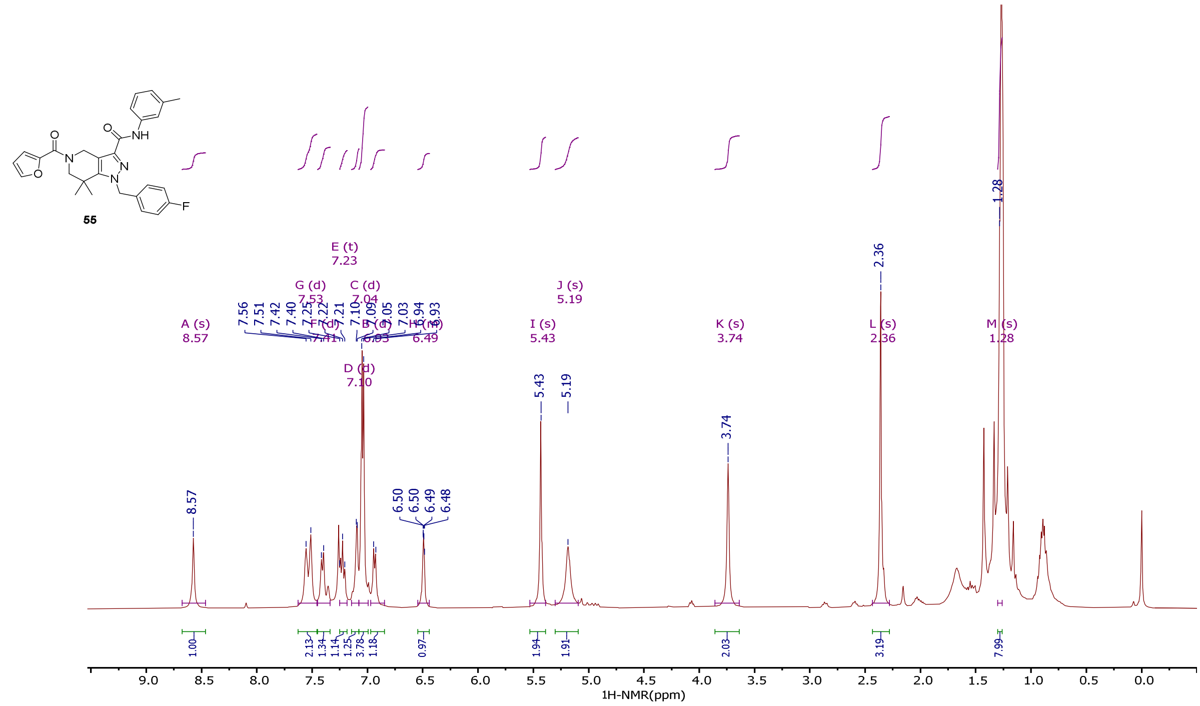
55
1H-NMR(ppm)

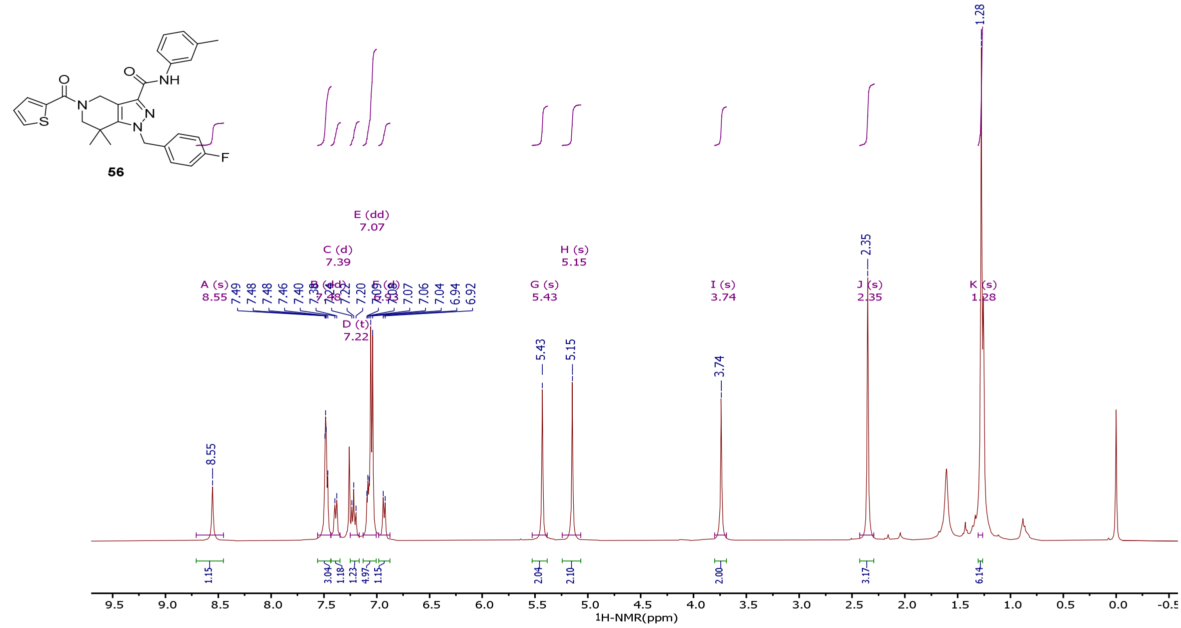
56
1H-NMR(ppm)

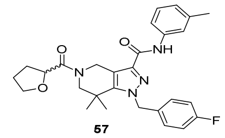

57

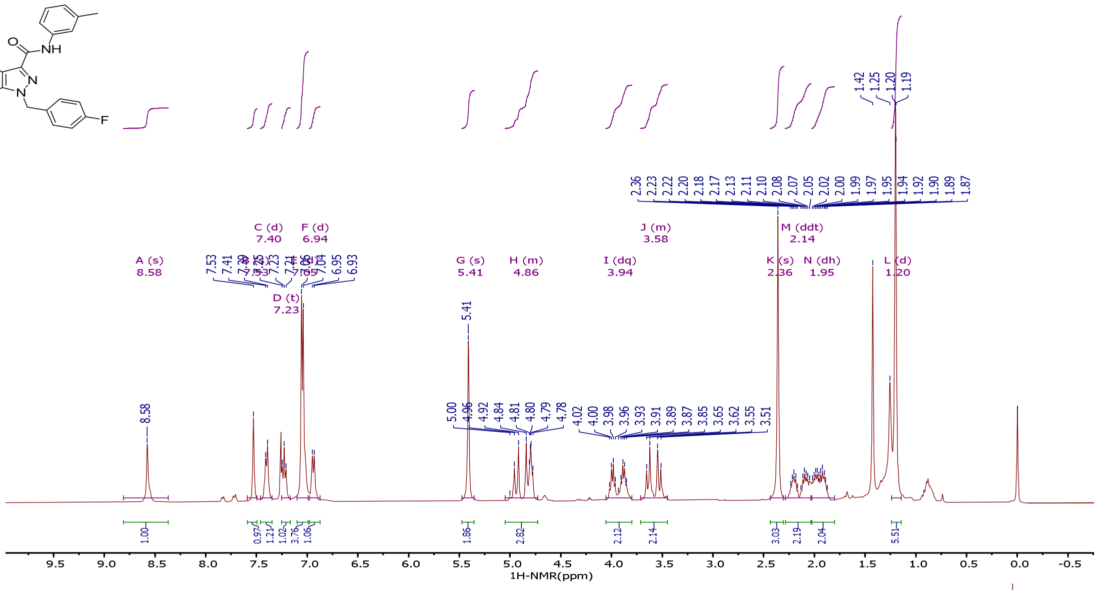

A (s) 8.58
C (d) 7.40
D (t) 7.23
F (d) 6.94
G (s) 5.41
H (m) 4.86
I (dq) 3.94
J (m) 3.58
K (s) 2.36
M (ddt) 2.14
N (dh) 1.95
L (d) 1.20
1H-NMR(ppm)

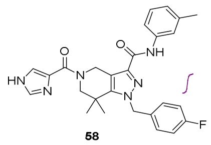

58

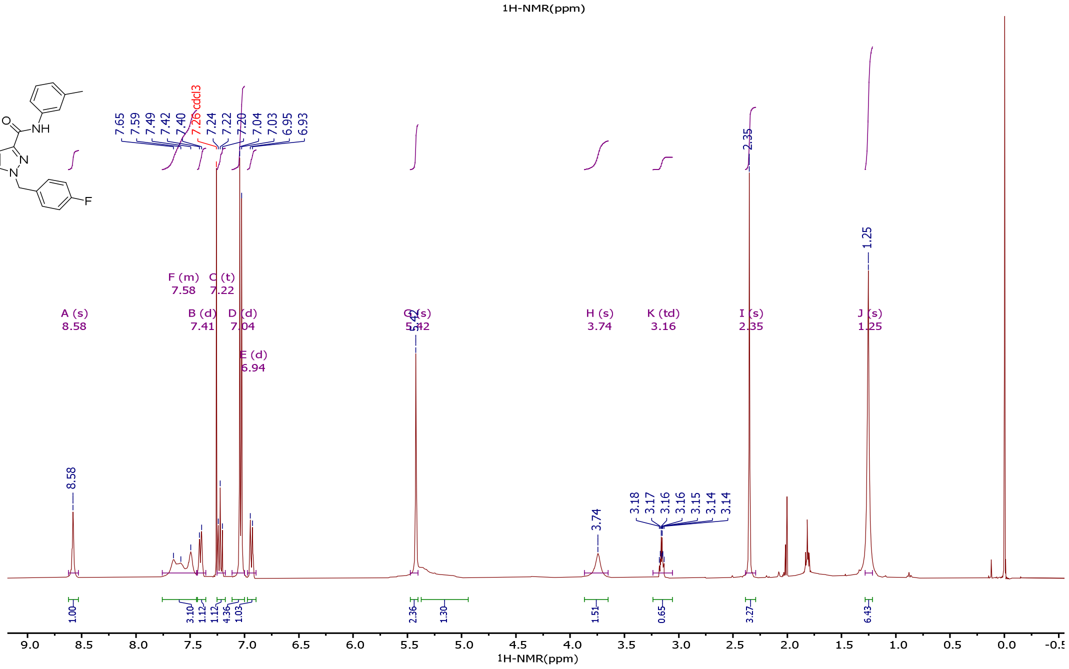

7.26 cdcl3
A (s) 8.58
F (m) 7.58
C (t) 7.22
B (d) 7.41
D (d) 7.04
E (d) 6.94
G (s) 5.42
H (s) 3.74
K (td) 3.16
I (s) 2.35
J (s) 1.25
1H-NMR(ppm)

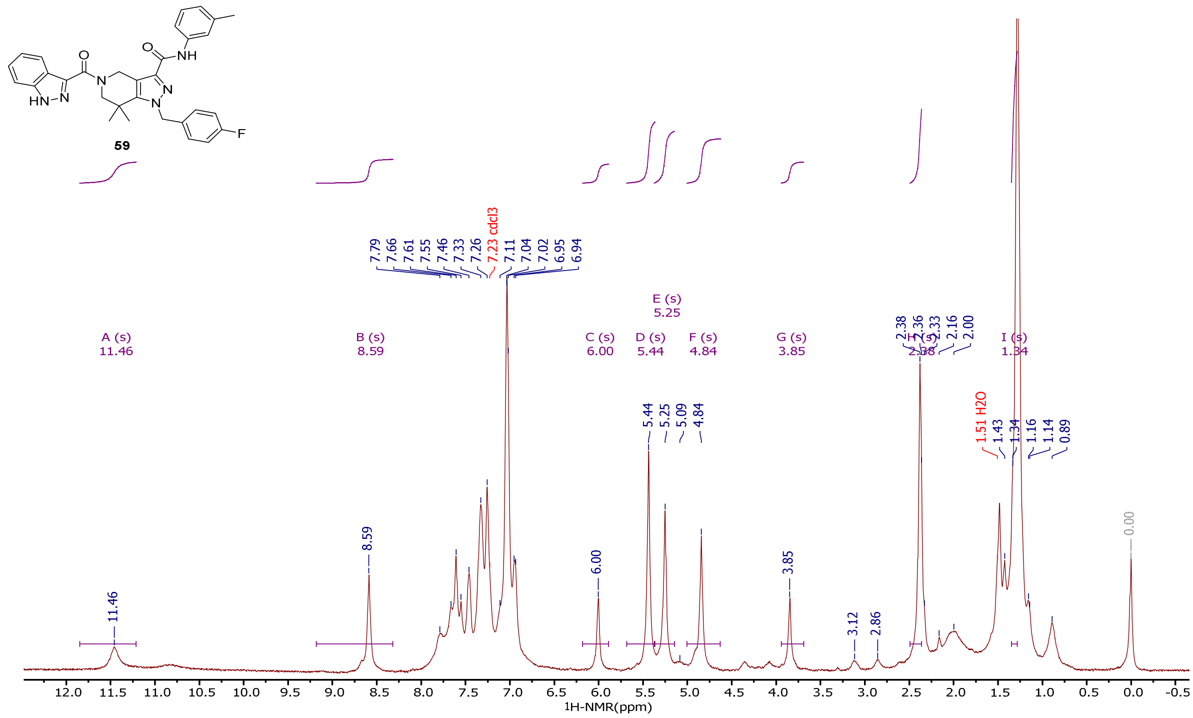

A (s) 11.46
B (s) 8.59
C (s) 6.00
D (s) 5.44
E (s) 5.25
F (s) 4.84
G (s) 3.85
I (s) 1.34
7.23 cdcl3
1.51 H2O
1H-NMR(ppm)

59

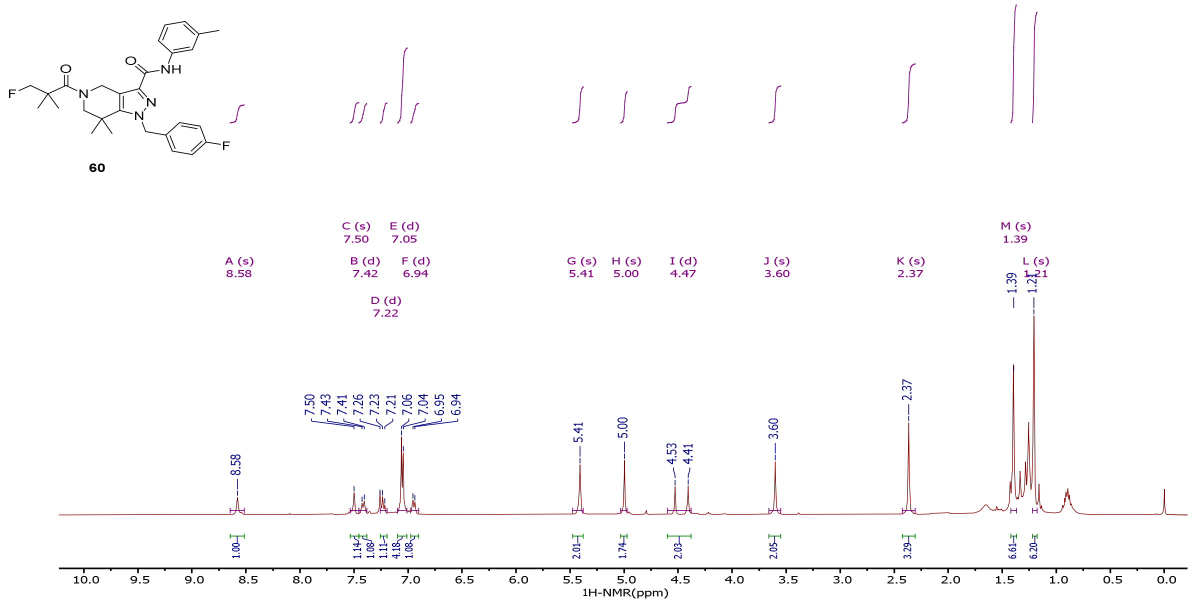

A (s) 8.58
B (d) 7.42
C (s) 7.50
D (d) 7.22
E (d) 7.05
F (d) 6.94
G (s) 5.41
H (s) 5.00
I (d) 4.47
J (s) 3.60
K (s) 2.37
L (s) 1.21
M (s) 1.39
1H-NMR(ppm)

60

61

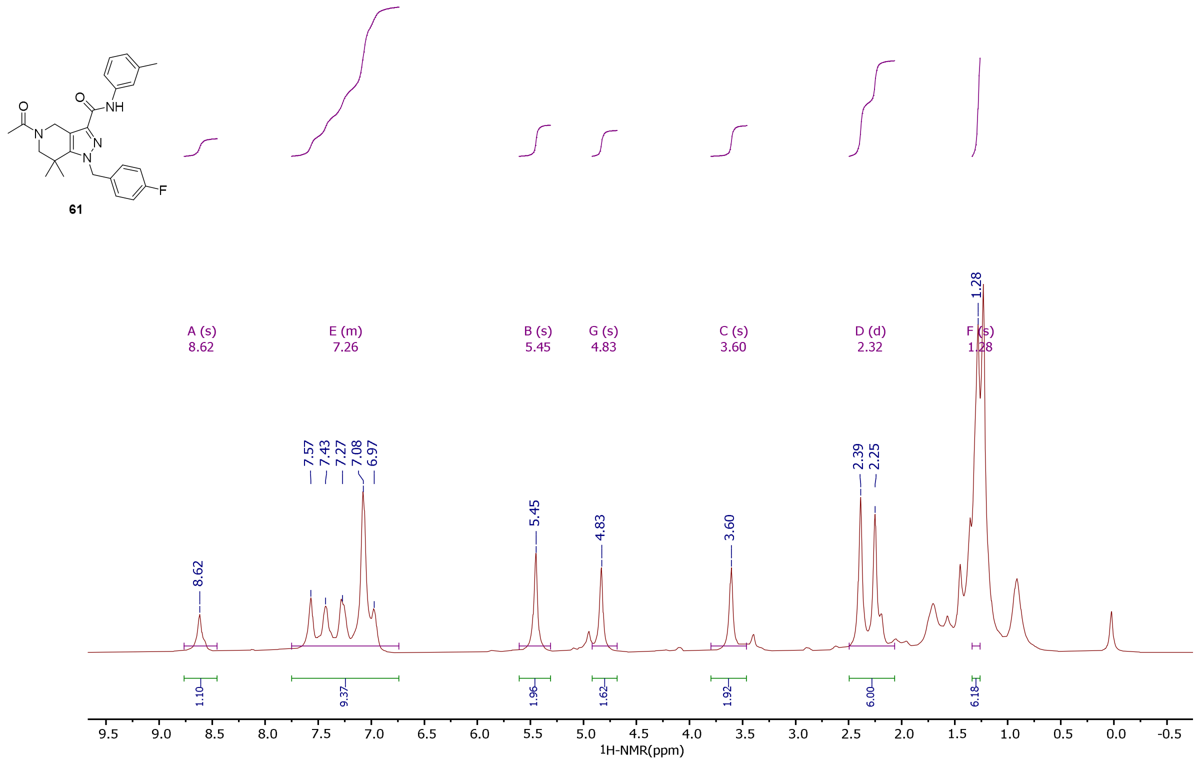
A (s) 8.62
E (m) 7.26
B (s) 5.45
G (s) 4.83
C (s) 3.60
D (d) 2.32
F (s) 1.28
1H-NMR(ppm)

62

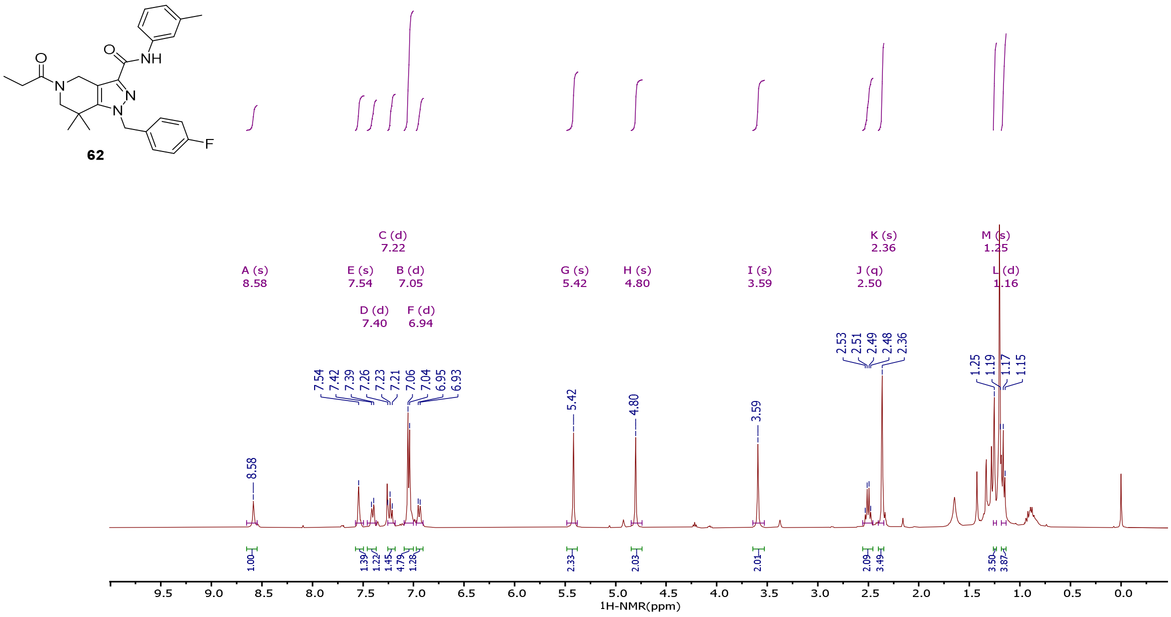
A (s) 8.58
C (d) 7.22
E (s) 7.54
B (d) 7.05
D (d) 7.40
F (d) 6.94
G (s) 5.42
H (s) 4.80
I (s) 3.59
J (q) 2.50
K (s) 2.36
M (s) 1.25
L (d) 1.16
1H-NMR(ppm)

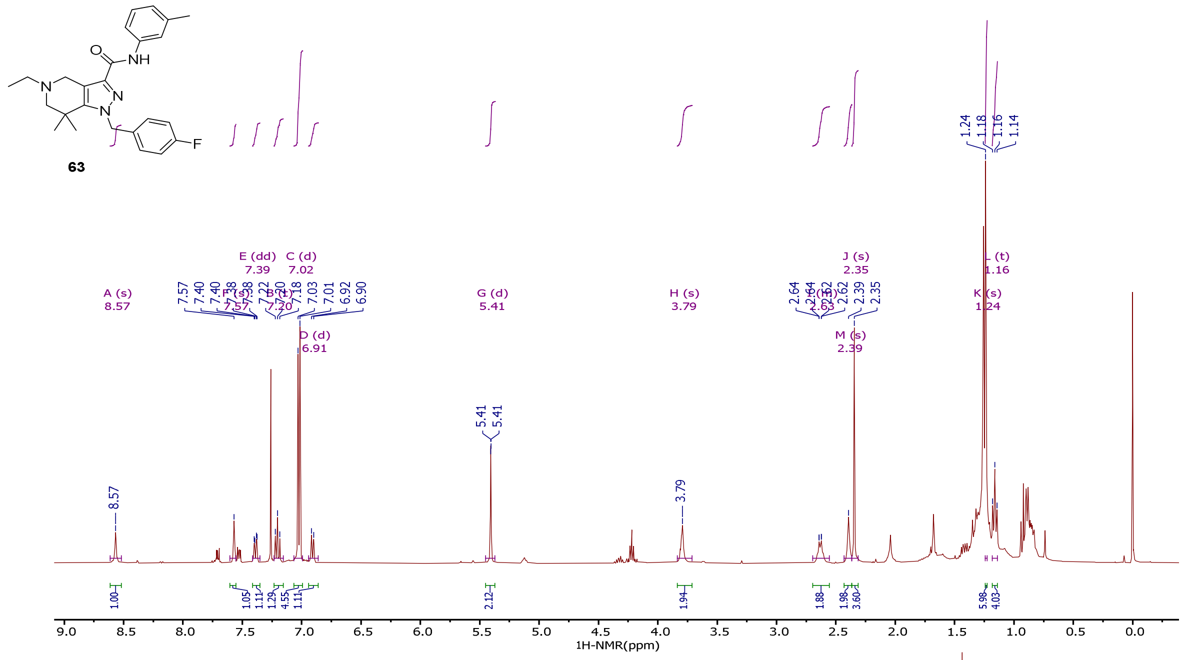

63

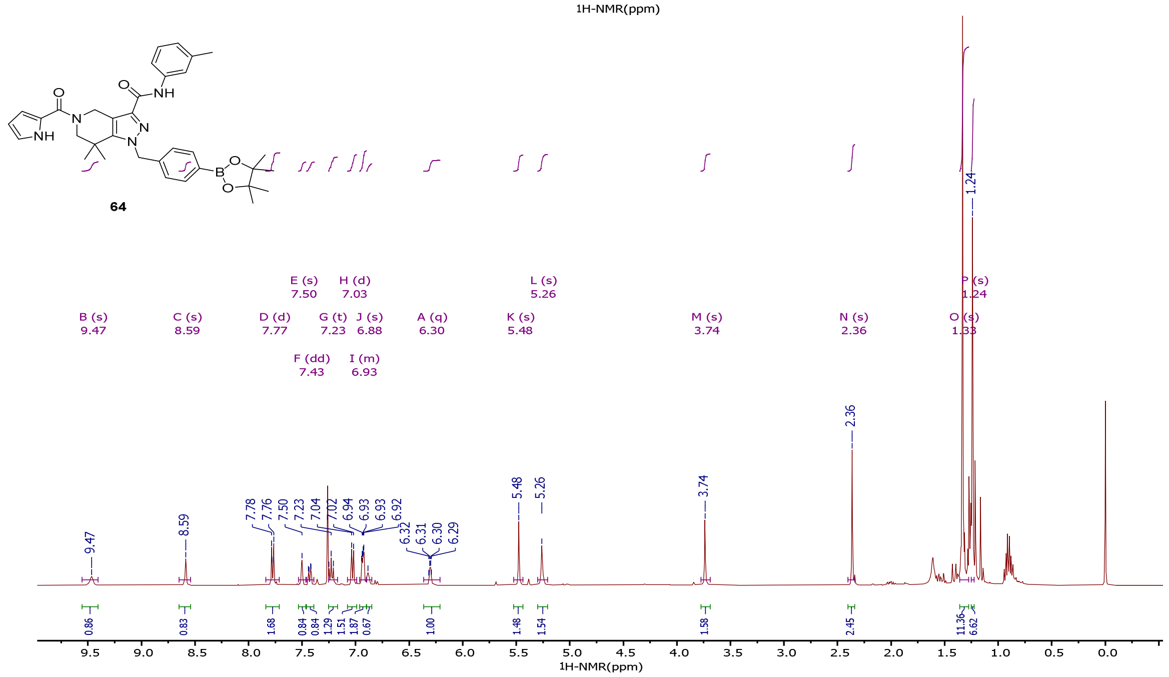

64

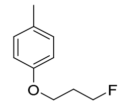
O
F

65

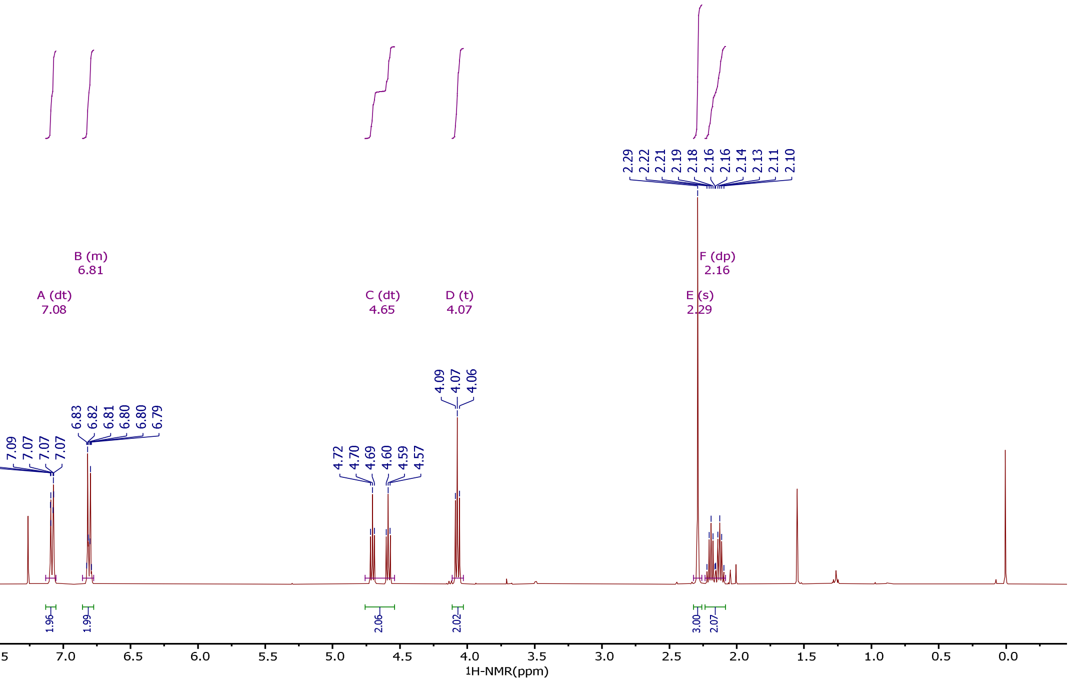
A (dt)
7.08
B (m)
6.81
C (dt)
4.65
D (t)
4.07
E (s)
2.29
F (dp)
2.16
1H-NMR(ppm)

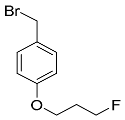
Br
O
F

66

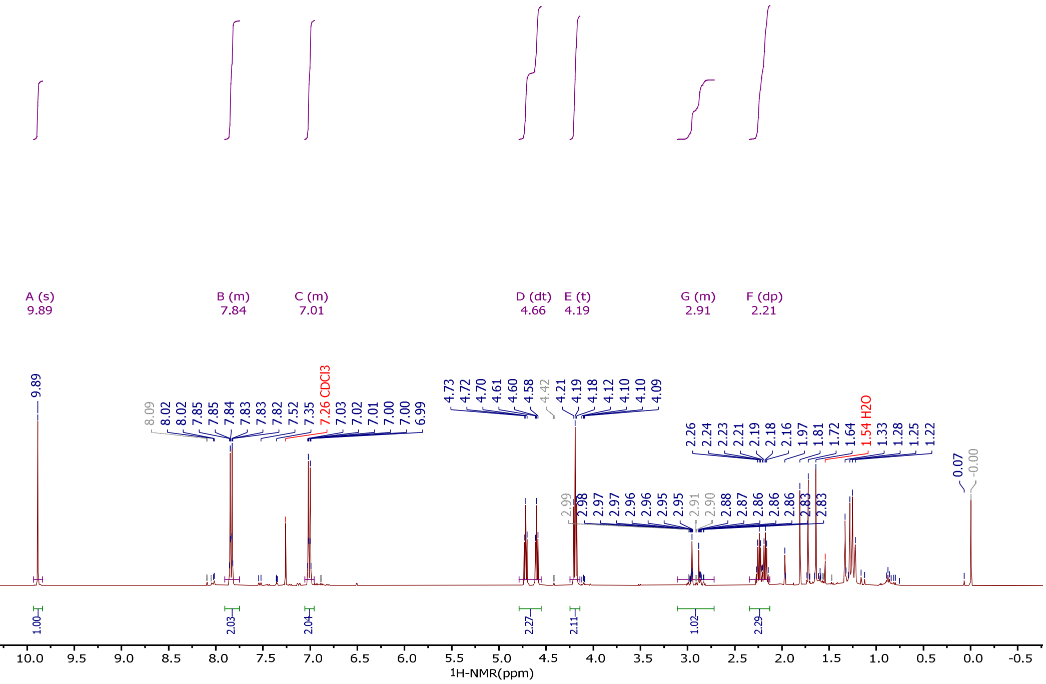
A (s)
9.89
B (m)
7.84
C (m)
7.01
D (dt)
4.66
E (t)
4.19
G (m)
2.91
F (dp)
2.21
7.26 CDCl3
1.54 H2O
1H-NMR(ppm)

S9: HPLC/LCMS CHROMATOGRAMS OF FINAL COMPOUNDS

- The purity of the final compounds (**17-42**, **47-51**, **55-63**) was additionally controlled by HPLC/LCMS.
- Purity ≥95 %
- HPLC system: HPLC was performed on a JASCO LC-2011 system, incorporating a PU-2080Plus pump, AS-2055Plus auto-injector (100 µL sample loop), and a UV-4075 detector (Jasco Deutschland GmbH, Pfungstadt, Germany) coupled with an optical activity HPLC detector. Data analysis was performed with the ChromNAV Ver.2 software. Stationary phase: Reprosil-Pur C18-AQ (250×4.5 mm, 5 µm, Dr. Maisch GmbH, Germany). Mobile phase: A mixture of MeCN and 20 mM NH_4OAc_{aq} was used as eluent in a linear gradient system with a flow of 1 mL/min. (eluent A 10 % MeCN/20 mM NH_4OAc_{aq}; eluent B 90 % MeCN/20 mM NH_4OAc_{aq}; **gradient A:** 0–17 min 0–100 % B, 17–26 min 100 % B, 26–30 min 100 % A; **gradient B:** 0–5 min 0–50 % B, 5–26 min 50–100 % B, 26–34 min 100 % B, 34–35 min 0 –100 % A, 35–40 100 % A).
- LCMS system: LC-MS was performed on a Dionex Ultimate 3000 system, incorporating an LPG-3400SD pump, an autosampler WPS-3000 TSL, a column compartment TCC-3000SD, a diode array detector DAD3000 (monitoring from 254 to 720 nm) and a low-resolution mass spectrometer MSQ 3000 (Thermo Fisher Scientific Inc., Waltham, USA). Stationary phase: Reprosil-Pur Basic HD column (150×3 mm, 3 µm, Dr. Maisch GmbH, Germany). Mobile phase: A mixture of MeCN and 20 mM NH_4OAc_{aq} was used as eluent in a linear gradient system with a flow of 0.7 mL/min. (gradient: eluent A 5 % MeCN/20 mM NH_4OAc_{aq}; eluent B 80 % MeCN/20 mM NH_4OAc_{aq}.; 0–1.5 min 100 % A, 1.5–10 min up to 100 % B, 10–12 min 100 % B, 12-15 min 100 % A).

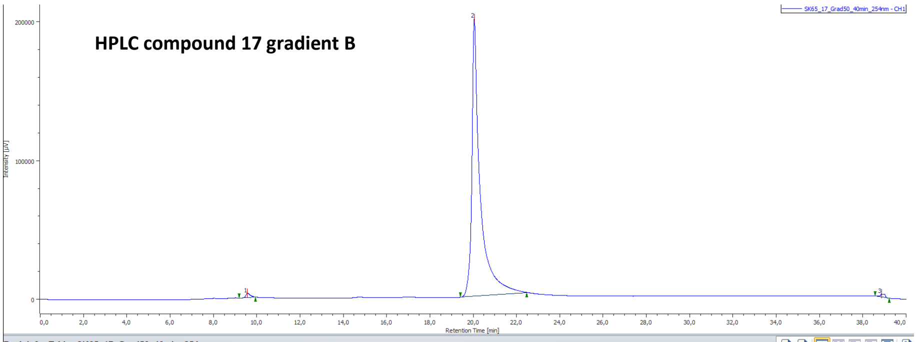

Peak Info. Table: SK65_17_Grad50_40min_254nm

#	Peak Name	CH	tR	Area	Height	Area%	Height%	Quantity	NTP	Resolution	Symmetry Factor	Warning
1	Unknown	1	9,567	43932	3261	0,815	1,586	N/A	13208	25,288	1,501	
2	Unknown	1	20,042	5310107	199978	98,519	97,276	N/A	25947	41,930	2,206	
3	Unknown	1	38,833	35870	2339	0,666	1,138	N/A	150021	N/A	1,017	

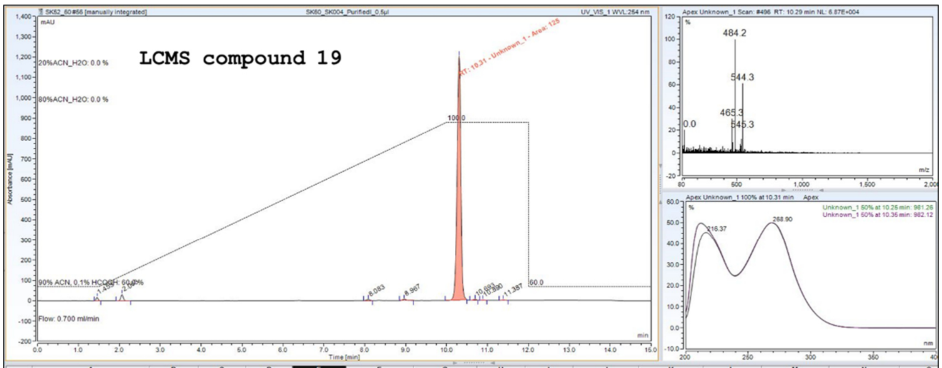

	A	B	C	D	E	F	G	H	I	J	K	L	M	N	O
1–2	Peak Name	Ret. Time min	Area mAU*min	Rel Area %	Amount	Overall Ion Ratio Confirmation	Quant. Ion m/z	Conf. Ion #1 m/z	Ion Ratio #1 (Expected)	Ion Ratio #1 (Observed)	Ion Ratio #1 Within Window	Conf.Ion #2 m/z	Ion Ratio #2 (Expected)	Ion Ratio #2 (Observed)	Ion Ra Within W
4		1.45	1	0.73	n.a.	Not confirmed	n.a.	n.a.	n.a.	n.a.	n.a.	n.a.	n.a.	n.a.	n.a
5		2.06	2	1.63	n.a.	Not confirmed	n.a.	n.a.	n.a.	n.a.	n.a.	n.a.	n.a.	n.a.	n.a
6		8.08	1	0.39	n.a.	Not confirmed	n.a.	n.a.	n.a.	n.a.	n.a.	n.a.	n.a.	n.a.	n.a
7		8.97	1	0.60	n.a.	Not confirmed	n.a.	n.a.	n.a.	n.a.	n.a.	n.a.	n.a.	n.a.	n.a
8	Unknown_1	10.31	125	96.19	n.a.	Not confirmed	n.a.	n.a.	n.a.	n.a.	n.a.	n.a.	n.a.	n.a.	n.a
9		10.69	0	0.21	n.a.	Not confirmed	n.a.	n.a.	n.a.	n.a.	n.a.	n.a.	n.a.	n.a.	n.a
10		10.89	0	0.11	n.a.	Not confirmed	n.a.	n.a.	n.a.	n.a.	n.a.	n.a.	n.a.	n.a.	n.a
11		11.39	0	0.13	n.a.	Not confirmed	n.a.	n.a.	n.a.	n.a.	n.a.	n.a.	n.a.	n.a.	n.a

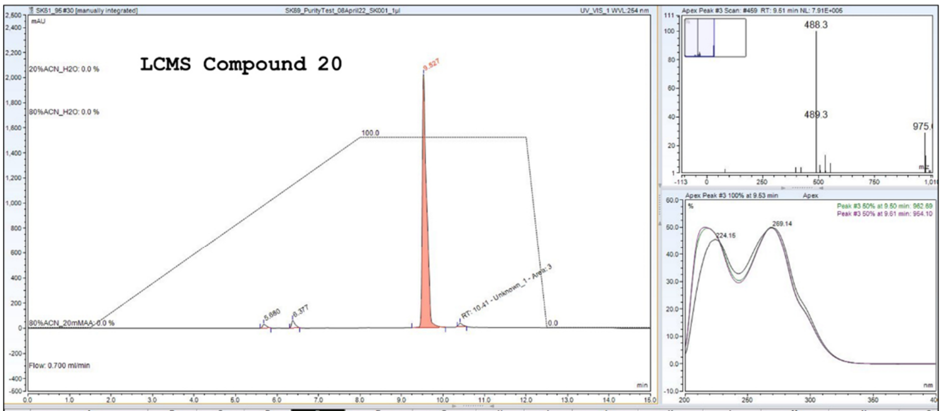

	A	B	C	D	E	F	G	H	I	J	K	L	M	N	O
1–2	Peak Name	Ret. Time min	Area mAU*min	Rel Area %	Amount	Overall Ion Ratio Confirmation	Quant. Ion m/z	Conf. Ion #1 m/z	Ion Ratio #1 (Expected)	Ion Ratio #1 (Observed)	Ion Ratio #1 Within Window	Conf.Ion #2 m/z	Ion Ratio #2 (Expected)	Ion Ratio #2 (Observed)	Ion Ra Within W
4		5.68	3	1.29	n.a.	Not confirmed	n.a.	n.a.	n.a.	n.a.	n.a.	n.a.	n.a.	n.a.	n.a
5		6.38	6	2.38	n.a.	Not confirmed	n.a.	n.a.	n.a.	n.a.	n.a.	n.a.	n.a.	n.a.	n.a
6		9.53	238	95.28	n.a.	Not confirmed	n.a.	n.a.	n.a.	n.a.	n.a.	n.a.	n.a.	n.a.	n.a
7	Unknown_1	10.41	3	1.06	n.a.	Not confirmed	n.a.	n.a.	n.a.	n.a.	n.a.	n.a.	n.a.	n.a.	n.a
8															

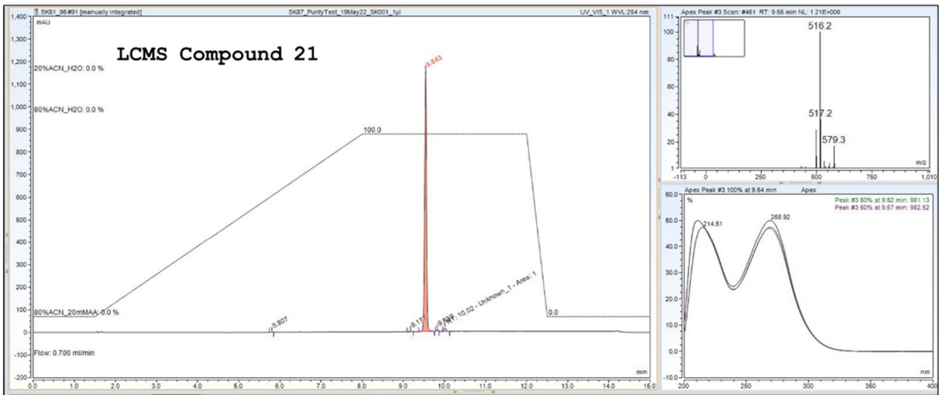

	A	B	C	D	E	F	G	H	I	J	K	L	M	N	O
1–2	Peak Name	Ret. Time min	Area mAU*min	Rel Area %	Amount	Overall Ion Ratio Confirmation	Quant. Ion m/z	Conf. Ion #1 m/z	Ion Ratio #1 (Expected)	Ion Ratio #1 (Observed)	Ion Ratio #1 Within Window	Conf.Ion #2 m/z	Ion Ratio #2 (Expected)	Ion Ratio #2 (Observed)	Ion Ra Within W
5		9.18	0	0.41	n.a.	Not confirmed	n.a.	n.a.	n.a.	n.a.	n.a.	n.a.	n.a.	n.a.	n.a
6		9.54	59	97.41	n.a.	Not confirmed	n.a.	n.a.	n.a.	n.a.	n.a.	n.a.	n.a.	n.a.	n.a
7		9.83	0	0.62	n.a.	Not confirmed	n.a.	n.a.	n.a.	n.a.	n.a.	n.a.	n.a.	n.a.	n.a
8	Unknown_1	10.02	1	1.34	n.a.	Not confirmed	n.a.	n.a.	n.a.	n.a.	n.a.	n.a.	n.a.	n.a.	n.a
9															

HPLC compound 22 gradient B

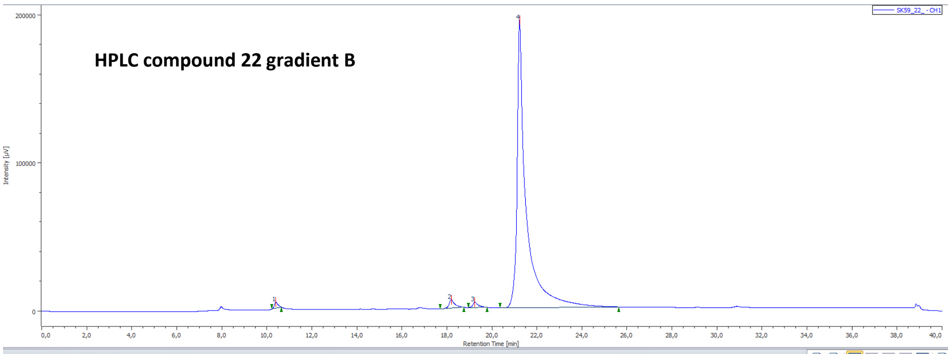

Peak Info. Table: SK59_22_

#	Peak Name	CH	tR	Area	Height	Area%	Height%	Quantity	NTP	Resolution	Symmetry Factor	Warning
1	Unknown	1	10,392	45224	4089	0,798	1,973	N/A	20741	22,960	1,273	
2	Unknown	1	18,183	99378	5735	1,753	2,767	N/A	34444	2,722	1,445	
3	Unknown	1	19,233	60337	3648	1,064	1,760	N/A	40657	4,680	1,592	
4	Unknown	1	21,225	5464484	193809	96,385	93,501	N/A	32379	N/A	2,348	

HPLC compound 23 gradient B

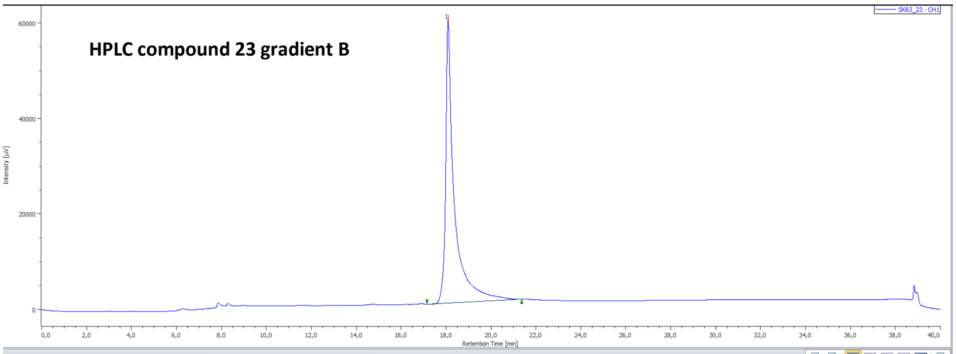

Peak Info. Table: SK63_23

#	Peak Name	CH	tR	Area	Height	Area%	Height%	Quantity	NTP	Resolution	Symmetry Factor	Warning
1	Unknown	1	18,083	1582014	59241	100,000	100,000	N/A	23315	N/A	2,382	

HPLC compound 24 gradient B

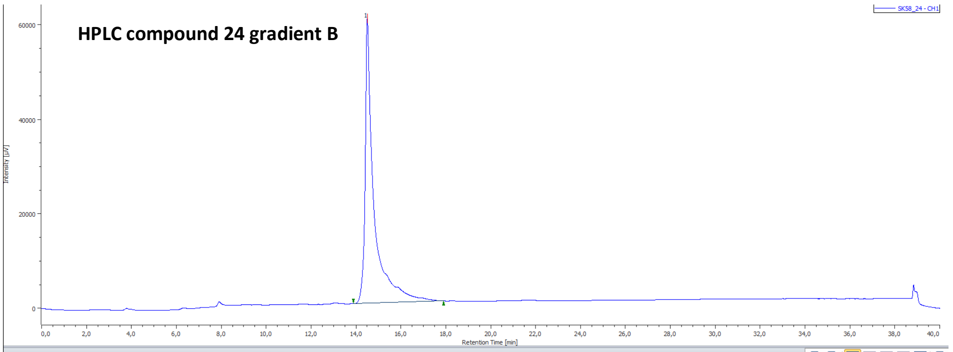

Peak Info. Table: SK58_24

#	Peak Name	CH	tR	Area	Height	Area%	Height%	Quantity	NTP	Resolution	Symmetry Factor	Warning
1	Unknown	1	14,500	1669907	60179	100,000	100,000	N/A	13774	N/A	2,948	

HPLC compound 18 gradient A

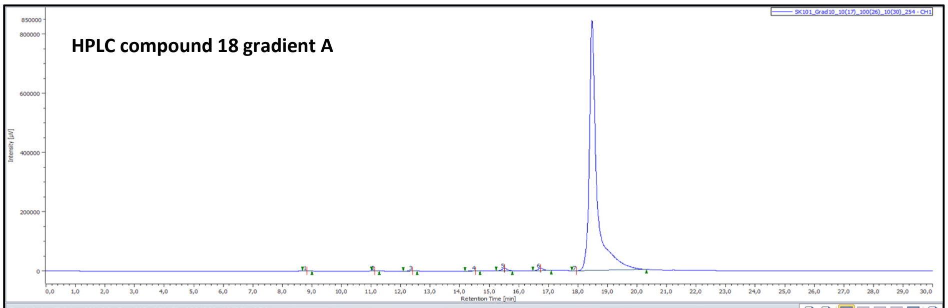

Peak Info. Table: SK101_Grad10_10(17)_100(26)_10(30)_254

#	Peak Name	CH	tR	Area	Height	Area%	Height%	Quantity	NTP	Resolution	Symmetry Factor	Warning
1	Unknown	1	8,825	4195	354	1,710	1,772	N/A	10247	7,762	1,093	
2	Unknown	1	11,117	4336	506	1,767	2,535	N/A	33390	4,625	1,167	
3	Unknown	1	12,400	10065	865	4,102	4,332	N/A	25104	7,012	0,802	
4	Unknown	1	14,525	8686	805	3,540	4,033	N/A	38878	3,246	0,886	
5	Unknown	1	15,508	104000	8429	42,383	42,230	N/A	39317	3,856	0,967	
6	Unknown	1	16,725	109190	8447	44,498	42,321	N/A	43731	4,251	1,220	
7	Unknown	1	17,942	4908	554	2,000	2,777	N/A	79795	N/A	0,802	

HPLC compound 25 gradient A

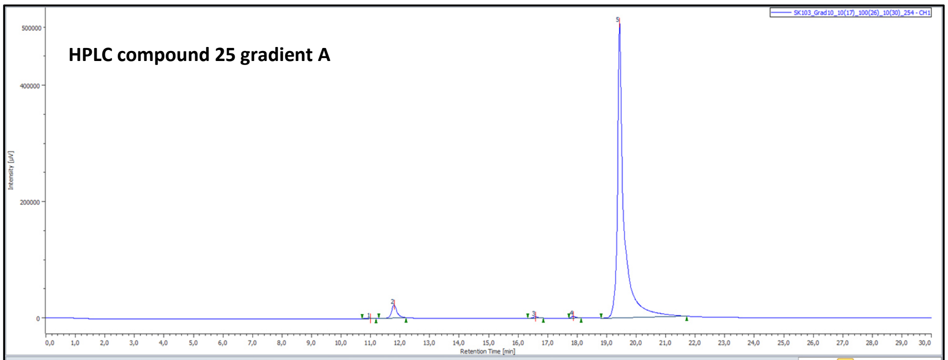

Peak Info. Table: SK103_Grad10_10(17)_100(26)_10(30)_254

#	Peak Name	CH	tR	Area	Height	Area%	Height%	Quantity	NTP	Resolution	Symmetry Factor	Warning
1	Unknown	1	10,983	13025	856	0,162	0,160	N/A	10756	2,236	0,843	
2	Unknown	1	11,792	283700	22578	3,531	4,214	N/A	24519	18,055	1,209	
3	Unknown	1	16,575	31965	3157	0,398	0,589	N/A	83063	5,613	1,103	
4	Unknown	1	17,858	26424	2838	0,329	0,530	N/A	97744	6,519	1,469	
5	Unknown	1	19,425	7678882	506384	95,580	94,508	N/A	93990	N/A	1,959	

HPLC compound 26 gradient B

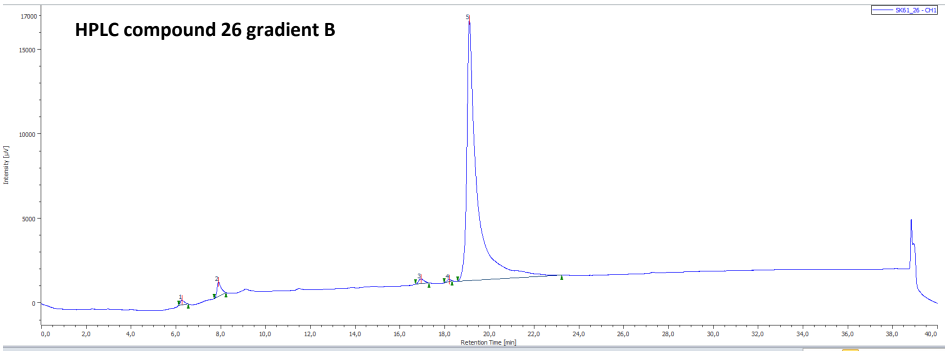

Peak Info. Table: SK61_26

#	Peak Name	CH	tR	Area	Height	Area%	Height%	Quantity	NTP	Resolution	Symmetry Factor	Warning
1	Unknown	1	6,267	3575	285	0,705	1,683	N/A	5552	4,972	1,403	
2	Unknown	1	7,900	10813	851	2,133	5,030	N/A	9611	22,921	1,583	
3	Unknown	1	16,933	4977	286	0,982	1,692	N/A	20947	3,176	1,278	
4	Unknown	1	18,183	1256	108	0,248	0,641	N/A	51251	2,054	0,804	
5	Unknown	1	19,100	486442	15381	95,933	90,954	N/A	17723	N/A	2,741	

HPLC compound 27 gradient B

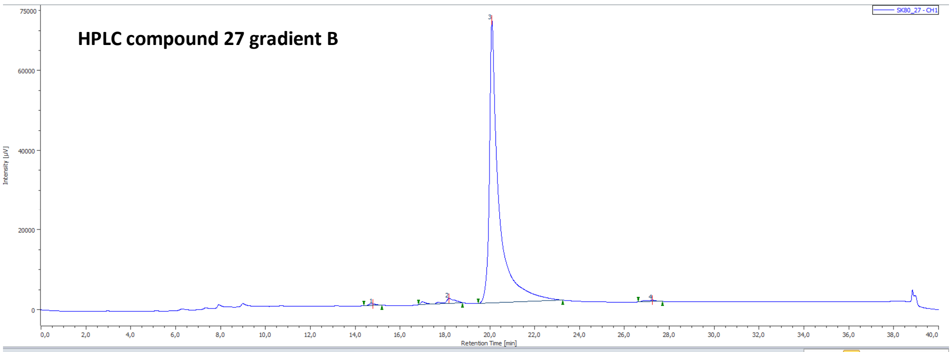

Peak Info. Table: SK80_27

#	Peak Name	CH	tR	Area	Height	Area%	Height%	Quantity	NTP	Resolution	Symmetry Factor	Warning
1	Unknown	1	14,783	9867	415	0,461	0,569	N/A	8470	2,127	1,041	
2	Unknown	1	18,175	46904	1265	2,190	1,734	N/A	810	1,239	0,710	
3	Unknown	1	20,083	2072005	70888	96,749	97,201	N/A	22531	8,853	2,582	
4	Unknown	1	27,217	12859	362	0,600	0,496	N/A	10150	N/A	0,873	

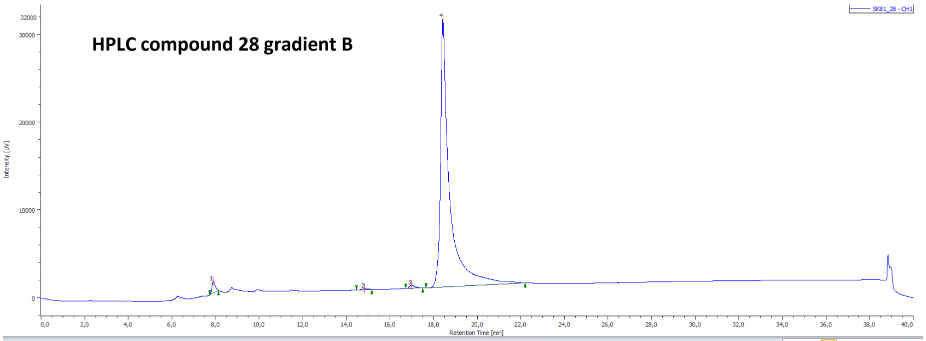

Peak Info. Table: SK81_28

#	Peak Name	CH	tR	Area	Height	Area%	Height%	Quantity	NTP	Resolution	Symmetry Factor	Warning
1	Unknown	1	7,892	13949	1333	1,544	4,114	N/A	13789	19,153	1,365	
2	Unknown	1	14,825	2395	139	0,265	0,429	N/A	16829	4,695	1,055	
3	Unknown	1	16,983	6519	355	0,722	1,096	N/A	21371	2,910	1,551	
4	Unknown	1	18,408	880415	30580	97,469	94,362	N/A	20268	N/A	2,613	

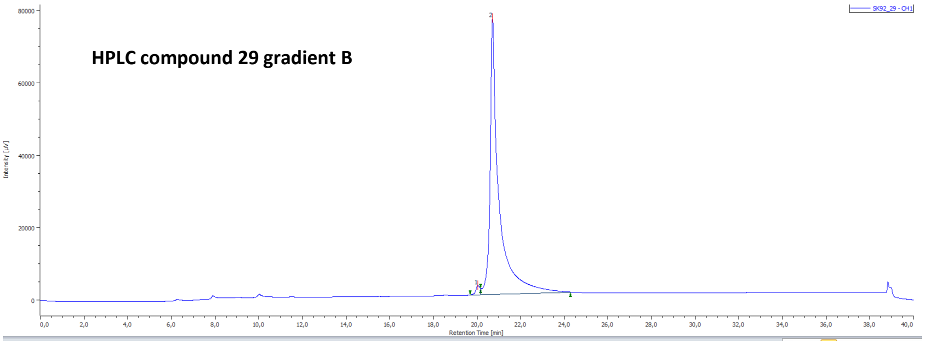

Peak Info. Table: SK92_29

#	Peak Name	CH	tR	Area	Height	Area%	Height%	Quantity	NTP	Resolution	Symmetry Factor	Warning
1	Unknown	1	20,033	33477	2569	1,536	3,264	N/A	N/A	N/A	N/A	
2	Unknown	1	20,700	2145586	76143	98,464	96,736	N/A	31696	N/A	2,379	

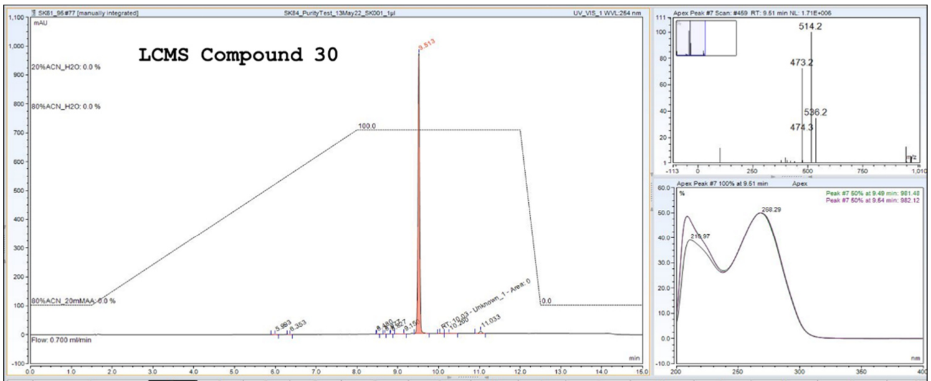

	A	B	C	D	E	F	G	H	I	J	K	L	M	N	O
1 / 2	Peak Name	Ret. Time min	Area mAU*min	Rel Area %	Amount	Overall Ion Ratio Confirmation	Quant. Ion m/z	Conf. Ion #1 m/z	Ion Ratio #1 (Expected)	Ion Ratio #1 (Observed)	Ion Ratio #1 Within Window	Conf.Ion #2 m/z	Ion Ratio #2 (Expected)	Ion Ratio #2 (Observed)	Ion Ra Within W
8		8.83	0	0.02	n.a.	Not confirmed	n.a.	n.a.	n.a.	n.a.	n.a.	n.a.	n.a.	n.a.	n.a
9		9.15	0	0.12	n.a.	Not confirmed	n.a.	n.a.	n.a.	n.a.	n.a.	n.a.	n.a.	n.a.	n.a
10		9.51	47	98.22	n.a.	Not confirmed	n.a.	n.a.	n.a.	n.a.	n.a.	n.a.	n.a.	n.a.	n.a
11	Unknown_1	10.03	0	0.17	n.a.	Not confirmed	n.a.	n.a.	n.a.	n.a.	n.a.	n.a.	n.a.	n.a.	n.a
12		10.26	0	0.18	n.a.	Not confirmed	n.a.	n.a.	n.a.	n.a.	n.a.	n.a.	n.a.	n.a.	n.a
13		11.03	1	1.15	n.a.	Not confirmed	n.a.	n.a.	n.a.	n.a.	n.a.	n.a.	n.a.	n.a.	n.a

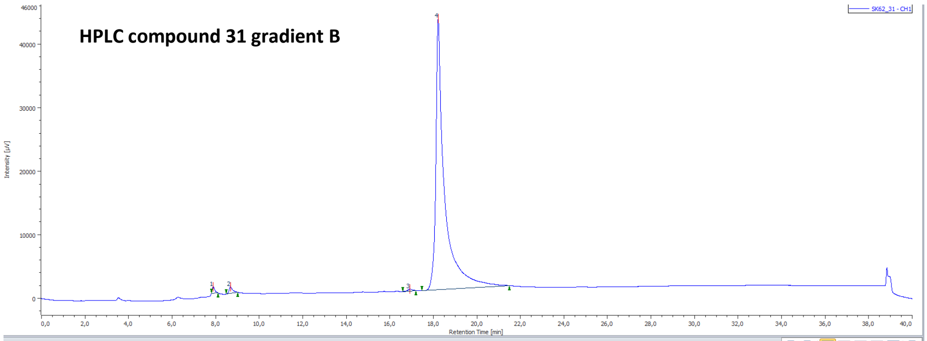

Peak Info. Table: SK62_31

#	Peak Name	CH	tR	Area	Height	Area%	Height%	Quantity	NTP	Resolution	Symmetry Factor	Warning
1	Unknown	1	7,883	8844	1045	0,729	2,324	N/A	20880	3,183	1,818	
2	Unknown	1	8,675	11921	1016	0,983	2,261	N/A	15308	25,539	1,516	
3	Unknown	1	16,917	3905	281	0,322	0,625	N/A	34054	3,017	1,198	
4	Unknown	1	18,208	1188244	42607	97,966	94,790	N/A	21922	N/A	2,560	

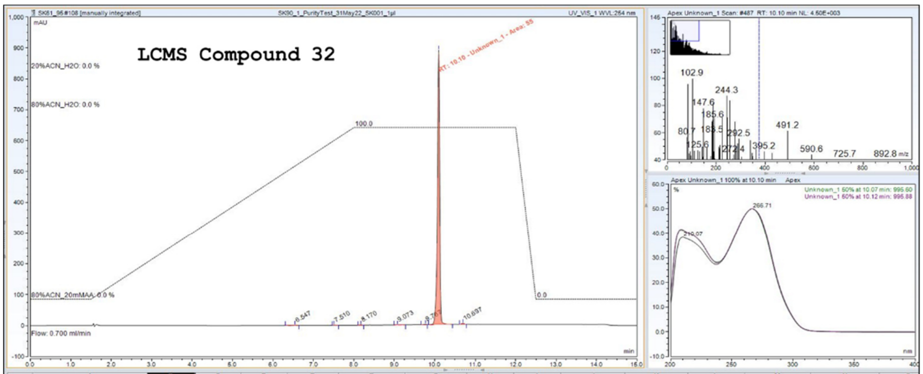

	A	B	C	D	E	F	G	H	I	J	K	L	M	N	O
1	Peak Name	Ret. Time	Area	Rel Area	Amount	Overall Ion Ratio	Quant. Ion	Conf. Ion #1	Ion Ratio #1	Ion Ratio #1	Ion Ratio #1	Conf.Ion #2	Ion Ratio #2	Ion Ratio #2	Ion Ra
2		min	mAU*min	%		Confirmation	m/z	m/z	(Expected)	(Observed)	Within Window	m/z	(Expected)	(Observed)	Within V
4		6.55	0	0.07	n.a.	Not confirmed	n.a.	n.a.	n.a.	n.a.	n.a.	n.a.	n.a.	n.a.	n.a
5		7.51	0	0.06	n.a.	Not confirmed	n.a.	n.a.	n.a.	n.a.	n.a.	n.a.	n.a.	n.a.	n.a
6		8.17	0	0.10	n.a.	Not confirmed	n.a.	n.a.	n.a.	n.a.	n.a.	n.a.	n.a.	n.a.	n.a
7		9.07	0	0.09	n.a.	Not confirmed	n.a.	n.a.	n.a.	n.a.	n.a.	n.a.	n.a.	n.a.	n.a
8		9.77	0	0.15	n.a.	Not confirmed	n.a.	n.a.	n.a.	n.a.	n.a.	n.a.	n.a.	n.a.	n.a
9	Unknown_1	10.10	55	99.15	n.a.	Not confirmed	n.a.	n.a.	n.a.	n.a.	n.a.	n.a.	n.a.	n.a.	n.a
10		10.70	0	0.38	n.a.	Not confirmed	n.a.	n.a.	n.a.	n.a.	n.a.	n.a.	n.a.	n.a.	n.a

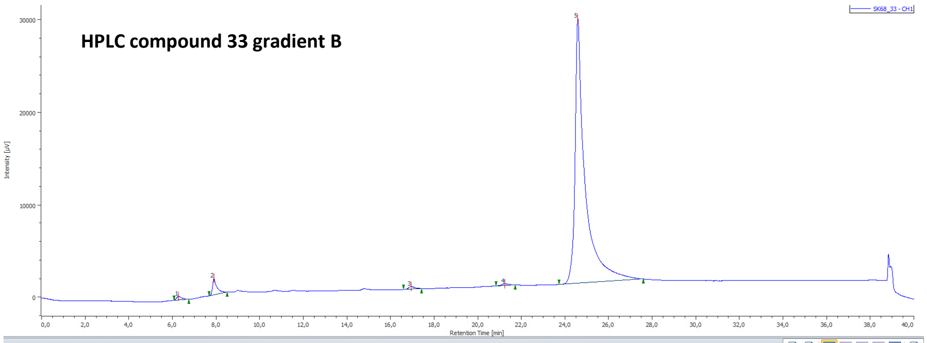

Peak Info. Table: SK68_33

#	Peak Name	CH	tR	Area	Height	Area%	Height%	Quantity	NTP	Resolution	Symmetry Factor	Warning
1	Unknown	1	6,283	6085	375	0,638	1,203	N/A	3917	4,647	1,525	
2	Unknown	1	7,908	24971	1774	2,618	5,683	N/A	11148	23,963	2,021	
3	Unknown	1	16,933	5502	298	0,577	0,955	N/A	22098	9,005	1,385	
4	Unknown	1	21,233	4291	201	0,450	0,642	N/A	28629	6,095	1,144	
5	Unknown	1	24,575	912805	28569	95,717	91,517	N/A	27068	N/A	2,402	

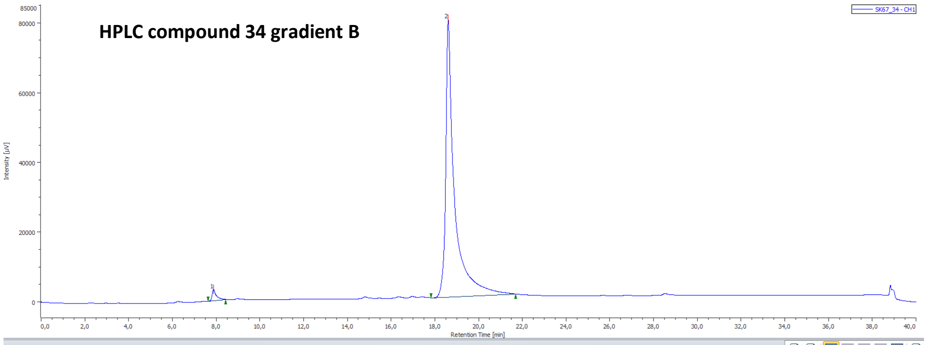

Peak Info. Table: SK67_34

#	Peak Name	CH	tR	Area	Height	Area%	Height%	Quantity	NTP	Resolution	Symmetry Factor	Warning
1	Unknown	1	7,883	43705	3318	1,923	4,005	N/A	13007	27,454	1,860	
2	Unknown	1	18,600	2228827	79542	98,077	95,995	N/A	21595	N/A	2,399	

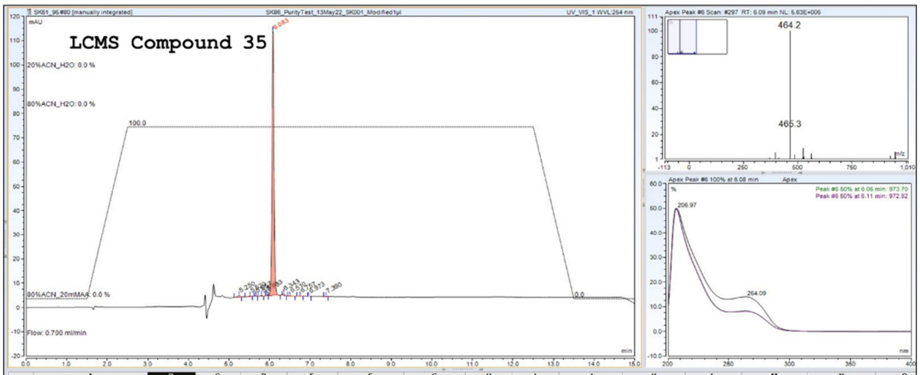

	A	B	C	D	E	F	G	H	I	J	K	L	M	N	O
1–2	Peak Name	Ret. Time min	Area mAU*min	Rel Area %	Amount n.a.	Overall Ion Ratio Confirmation	Quant. Ion m/z	Conf. Ion #1 m/z	Ion Ratio #1 (Expected)	Ion Ratio #1 (Observed)	Ion Ratio #1 Within Window	Conf.Ion #2 m/z	Ion Ratio #2 (Expected)	Ion Ratio #2 (Observed)	Ion Ra Within W
4		5.25	0	0.75	n.a.	Not confirmed	n.a.	n.a.	n.a.	n.a.	n.a.	n.a.	n.a.	n.a.	n.a
5		5.52	0	0.31	n.a.	Not confirmed	n.a.	n.a.	n.a.	n.a.	n.a.	n.a.	n.a.	n.a.	n.a
6		5.66	0	0.20	n.a.	Not confirmed	n.a.	n.a.	n.a.	n.a.	n.a.	n.a.	n.a.	n.a.	n.a
7		5.80	0	0.29	n.a.	Not confirmed	n.a.	n.a.	n.a.	n.a.	n.a.	n.a.	n.a.	n.a.	n.a
8		5.93	0	0.46	n.a.	Not confirmed	n.a.	n.a.	n.a.	n.a.	n.a.	n.a.	n.a.	n.a.	n.a
9		6.08	6	96.91	n.a.	Not confirmed	n.a.	n.a.	n.a.	n.a.	n.a.	n.a.	n.a.	n.a.	n.a
10		6.34	0	0.71	n.a.	Not confirmed	n.a.	n.a.	n.a.	n.a.	n.a.	n.a.	n.a.	n.a.	n.a

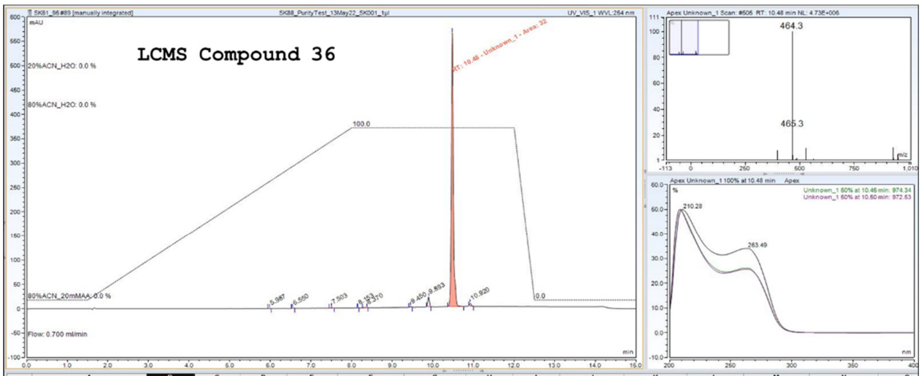

	A	B	C	D	E	F	G	H	I	J	K	L	M	N	O
1–2	Peak Name	Ret. Time min	Area mAU*min	Rel Area %	Amount	Overall Ion Ratio Confirmation	Quant. Ion m/z	Conf. Ion #1 m/z	Ion Ratio #1 (Expected)	Ion Ratio #1 (Observed)	Ion Ratio #1 Within Window	Conf.Ion #2 m/z	Ion Ratio #2 (Expected)	Ion Ratio #2 (Observed)	Ion Ra Within W
6		7.50	0	0.25	n.a.	Not confirmed	n.a.	n.a.	n.a.	n.a.	n.a.	n.a.	n.a.	n.a.	n.a
7		8.15	0	0.02	n.a.	Not confirmed	n.a.	n.a.	n.a.	n.a.	n.a.	n.a.	n.a.	n.a.	n.a
8		8.37	0	0.10	n.a.	Not confirmed	n.a.	n.a.	n.a.	n.a.	n.a.	n.a.	n.a.	n.a.	n.a
9		9.45	0	0.22	n.a.	Not confirmed	n.a.	n.a.	n.a.	n.a.	n.a.	n.a.	n.a.	n.a.	n.a
10		9.89	1	2.85	n.a.	Not confirmed	n.a.	n.a.	n.a.	n.a.	n.a.	n.a.	n.a.	n.a.	n.a
11	Unknown_1	10.48	32	95.70	n.a.	Not confirmed	n.a.	n.a.	n.a.	n.a.	n.a.	n.a.	n.a.	n.a.	n.a
12		10.92	0	0.75	n.a.	Not confirmed	n.a.	n.a.	n.a.	n.a.	n.a.	n.a.	n.a.	n.a.	n.a

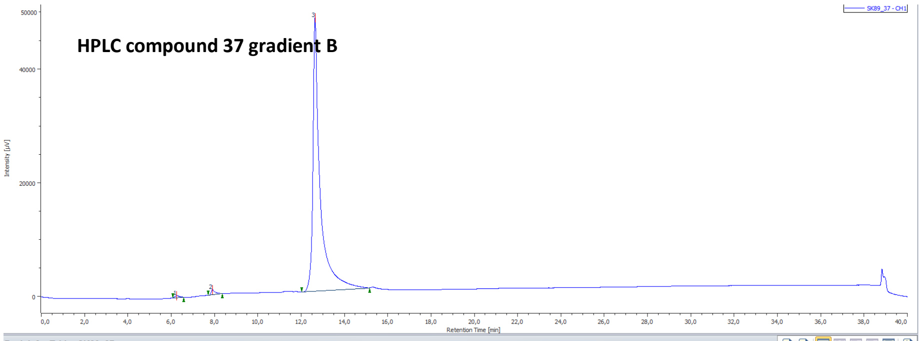

Peak Info. Table: SK89_37

#	Peak Name	CH	tR	Area	Height	Area%	Height%	Quantity	NTP	Resolution	Symmetry Factor	Warning
1	Unknown	1	6,258	4706	348	0,418	0,707	N/A	5126	5,042	1,320	
2	Unknown	1	7,900	11397	870	1,013	1,769	N/A	10858	12,912	2,083	
3	Unknown	1	12,642	1109120	47960	98,569	97,524	N/A	13628	N/A	2,310	

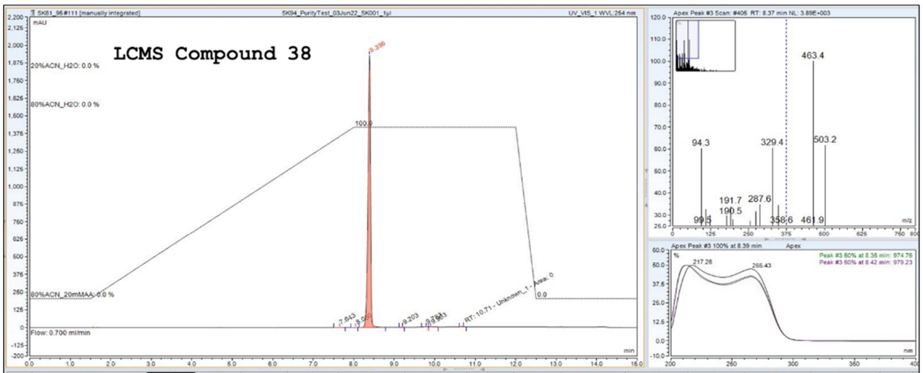

	A	B	C	D	E	F	G	H	I	J	K	L	M	N	O
1–2	Peak Name	Ret. Time min	Area mAU*min	Rel Area %	Amount	Overall Ion Ratio Confirmation	Quant. Ion m/z	Conf. Ion #1 m/z	Ion Ratio #1 (Expected)	Ion Ratio #1 (Observed)	Ion Ratio #1 Within Window	Conf.Ion #2 m/z	Ion Ratio #2 (Expected)	Ion Ratio #2 (Observed)	Ion Ra Within W
4		7.64	0	0.07	n.a.	Not confirmed	n.a.	n.a.	n.a.	n.a.	n.a.	n.a.	n.a.	n.a.	n.a
5		8.05	0	0.04	n.a.	Not confirmed	n.a.	n.a.	n.a.	n.a.	n.a.	n.a.	n.a.	n.a.	n.a
6		8.39	136	99.41	n.a.	Not confirmed	n.a.	n.a.	n.a.	n.a.	n.a.	n.a.	n.a.	n.a.	n.a
7		9.20	0	0.03	n.a.	Not confirmed	n.a.	n.a.	n.a.	n.a.	n.a.	n.a.	n.a.	n.a.	n.a
8		9.78	0	0.09	n.a.	Not confirmed	n.a.	n.a.	n.a.	n.a.	n.a.	n.a.	n.a.	n.a.	n.a
9		9.90	0	0.11	n.a.	Not confirmed	n.a.	n.a.	n.a.	n.a.	n.a.	n.a.	n.a.	n.a.	n.a
10	Unknown_1	10.71	0	0.25	n.a.	Not confirmed	n.a.	n.a.	n.a.	n.a.	n.a.	n.a.	n.a.	n.a.	n.a

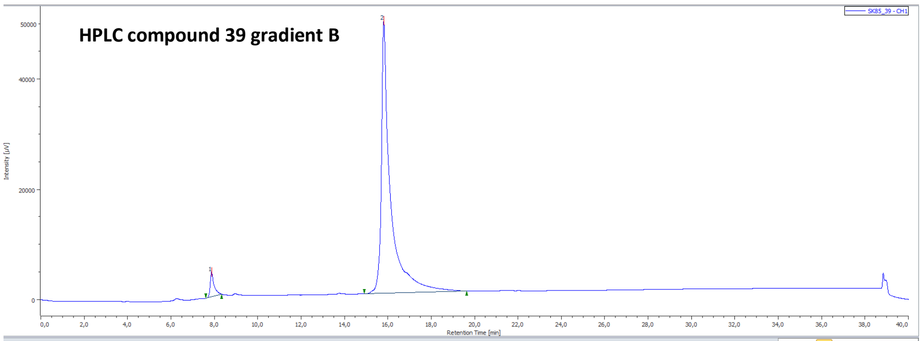

Peak Info. Table: SK85_39

#	Peak Name	CH	tR	Area	Height	Area%	Height%	Quantity	NTP	Resolution	Symmetry Factor	Warning
1	Unknown	1	7,875	56245	4495	3,770	8,349	N/A	13375	20,264	1,693	
2	Unknown	1	15,800	1435562	49339	96,230	91,651	N/A	15243	N/A	2,683	

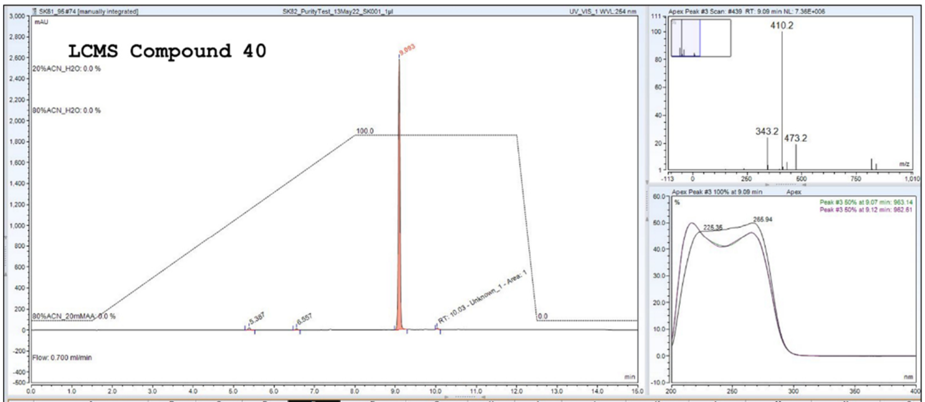

	A	B	C	D	E	F	G	H	I	J	K	L	M	N	O
1	Peak Name	Ret. Time	Area	Rel Area	Amount	Overall Ion Ratio	Quant. Ion	Conf. Ion #1	Ion Ratio #1	Ion Ratio #1	Ion Ratio #1	Conf.Ion #2	Ion Ratio #2	Ion Ratio #2	Ion Ra
2		min	mAU*min	%		Confirmation	m/z	m/z	(Expected)	(Observed)	Within Window	m/z	(Expected)	(Observed)	Within V
4		5.39	1	1.08	n.a.	Not confirmed	n.a.	n.a.	n.a.	n.a.	n.a.	n.a.	n.a.	n.a.	n.a
5		6.56	1	0.56	n.a.	Not confirmed	n.a.	n.a.	n.a.	n.a.	n.a.	n.a.	n.a.	n.a.	n.a
6		9.09	124	97.72	n.a.	Not confirmed	n.a.	n.a.	n.a.	n.a.	n.a.	n.a.	n.a.	n.a.	n.a
7	Unknown_1	10.03	1	0.64	n.a.	Not confirmed	n.a.	n.a.	n.a.	n.a.	n.a.	n.a.	n.a.	n.a.	n.a

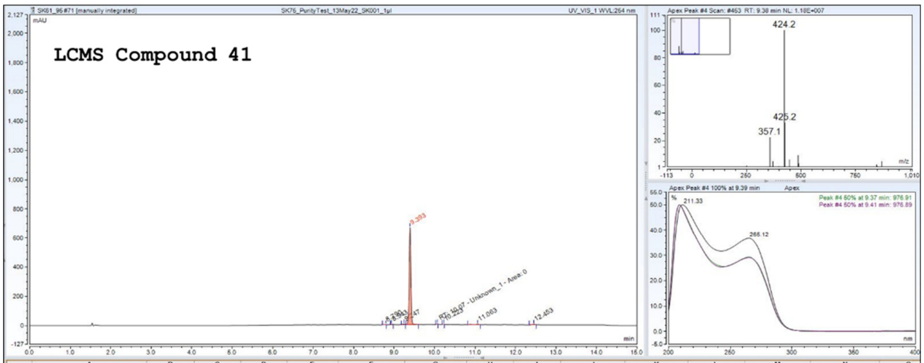

	A	B	C	D	E	F	G	H	I	J	K	L	M	N	O
1	Peak Name	Ret. Time	Area	Rel Area	Amount	Overall Ion Ratio	Quant. Ion	Conf. Ion #1	Ion Ratio #1	Ion Ratio #1	Ion Ratio #1	Conf.Ion #2	Ion Ratio #2	Ion Ratio #2	Ion Ra
2		min	mAU*min	%		Confirmation	m/z	m/z	(Expected)	(Observed)	Within Window	m/z	(Expected)	(Observed)	Within V
4		8.79	0	0.23	n.a.	Not confirmed	n.a.	n.a.	n.a.	n.a.	n.a.	n.a.	n.a.	n.a.	n.a
5		8.94	0	0.26	n.a.	Not confirmed	n.a.	n.a.	n.a.	n.a.	n.a.	n.a.	n.a.	n.a.	n.a
6		9.25	0	0.67	n.a.	Not confirmed	n.a.	n.a.	n.a.	n.a.	n.a.	n.a.	n.a.	n.a.	n.a
7		9.39	32	96.16	n.a.	Not confirmed	n.a.	n.a.	n.a.	n.a.	n.a.	n.a.	n.a.	n.a.	n.a
8	Unknown_1	10.07	0	0.32	n.a.	Not confirmed	n.a.	n.a.	n.a.	n.a.	n.a.	n.a.	n.a.	n.a.	n.a
9		10.22	0	0.20	n.a.	Not confirmed	n.a.	n.a.	n.a.	n.a.	n.a.	n.a.	n.a.	n.a.	n.a
10		11.06	0	1.27	n.a.	Not confirmed	n.a.	n.a.	n.a.	n.a.	n.a.	n.a.	n.a.	n.a.	n.a
11		12.45	0	0.89	n.a.	Not confirmed	n.a.	n.a.	n.a.	n.a.	n.a.	n.a.	n.a.	n.a.	n.a

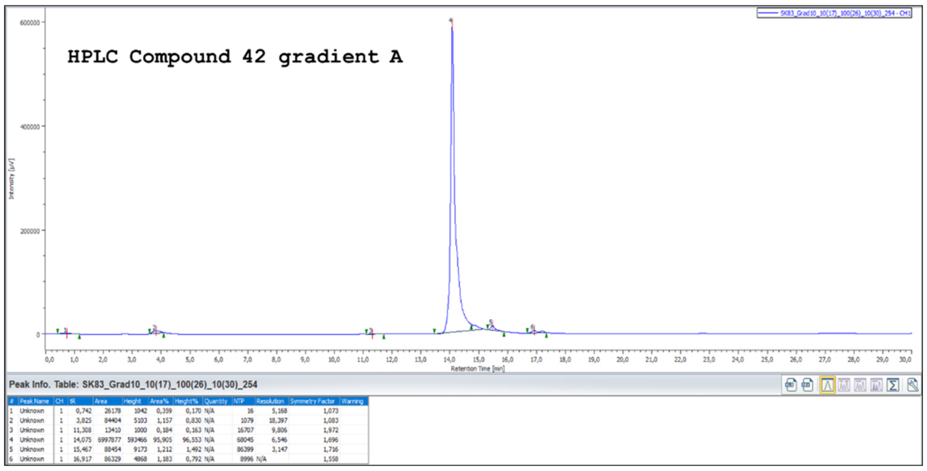

Peak Info. Table: SK83_Grad10_10(17)_100(26)_10(30)_254

#	Peak Name	CH	tR	Area	Height	Area%	Height%	Quantity	NTP	Resolution	Symmetry Factor	Warning
1	Unknown	1	0,742	26178	1042	0,359	0,170	N/A	16	5,168	1,073	
2	Unknown	1	3,825	84404	5103	1,157	0,830	N/A	1079	18,397	1,083	
3	Unknown	1	11,308	13410	1000	0,184	0,163	N/A	16707	9,806	1,972	
4	Unknown	1	14,075	6997877	593466	95,905	96,553	N/A	68045	6,546	1,696	
5	Unknown	1	15,467	88454	9173	1,212	1,492	N/A	86399	3,147	1,716	
6	Unknown	1	16,917	86329	4868	1,183	0,792	N/A	8996	N/A	1,558	

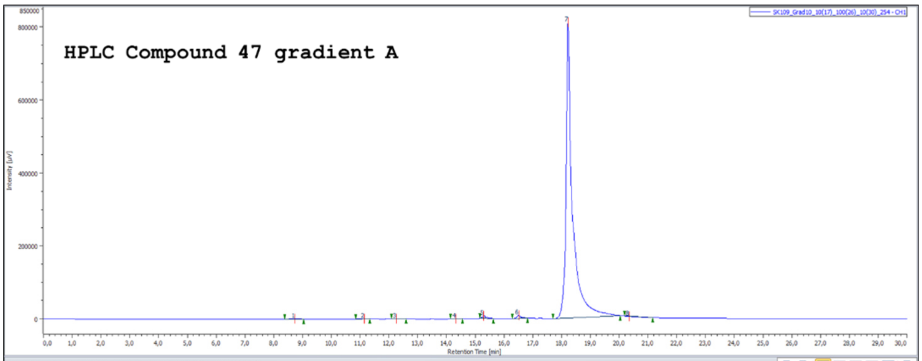

Peak Info. Table: SK109_Grad10_10(17)_100(26)_10(30)_254

#	Peak Name	CH	tR	Area	Height	Area%	Height%	Quantity	NTP	Resolution	Symmetry Factor	Warning
1	Unknown	1	8,742	27484	1300	0,214	0,157	N/A	3443	4,439	0,903	
2	Unknown	1	11,150	9206	699	0,072	0,084	N/A	8217	2,616	0,761	
3	Unknown	1	12,258	12432	907	0,097	0,109	N/A	18807	6,149	1,396	
4	Unknown	1	14,317	11278	1019	0,088	0,123	N/A	33318	3,691	1,348	
5	Unknown	1	15,283	70394	7641	0,548	0,921	N/A	83526	5,505	1,751	
6	Unknown	1	16,500	85472	8135	0,665	0,980	N/A	81166	6,955	1,340	
7	Unknown	1	18,217	12603211	809913	98,125	97,569	N/A	76582	3,096	1,933	
8	Unknown	1	20,342	24618	474	0,192	0,057	N/A	5345	N/A	2,351	

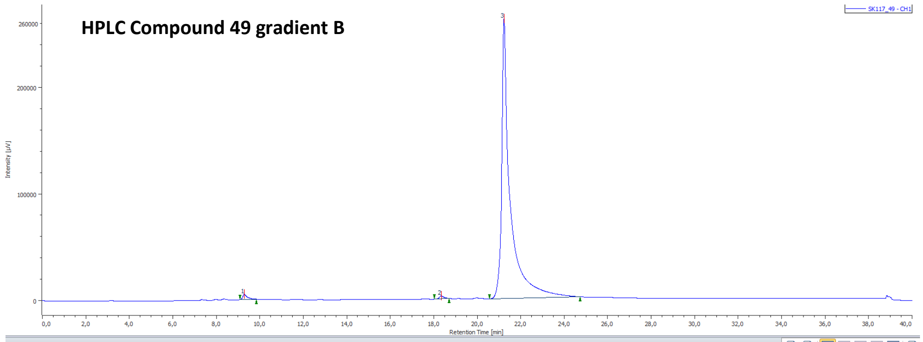

Peak Info. Table: SK117_49

#	Peak Name	CH	tR	Area	Height	Area%	Height%	Quantity	NTP	Resolution	Symmetry Factor	Warning
1	Unknown	1	9,275	72217	5109	0,996	1,892	N/A	14736	27,543	1,915	
2	Unknown	1	18,333	40395	2805	0,557	1,039	N/A	42938	7,269	1,247	
3	Unknown	1	21,225	7139559	262148	98,447	97,069	N/A	36591	N/A	2,403	

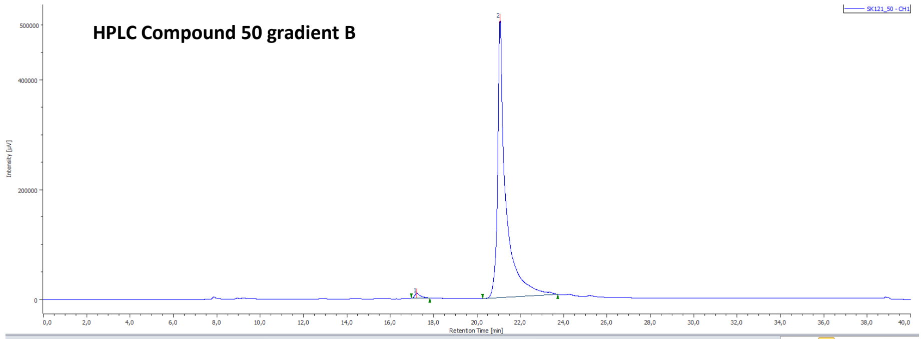

Peak Info. Table: SK121_50

#	Peak Name	CH	tR	Area	Height	Area%	Height%	Quantity	NTP	Resolution	Symmetry Factor	Warning
1	Unknown	1	17,217	161090	9226	1,192	1,785	N/A	28262	8,947	1,669	
2	Unknown	1	21,050	13348045	507646	98,808	98,215	N/A	35083	N/A	2,234	

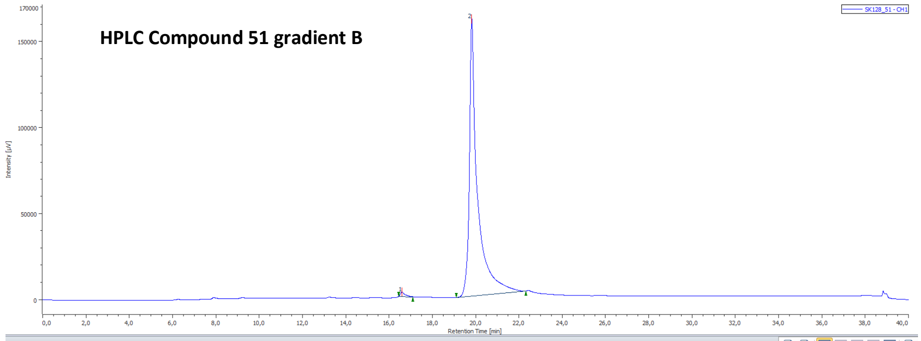

Peak Info. Table: SK128_51

#	Peak Name	CH	tR	Area	Height	Area%	Height%	Quantity	NTP	Resolution	Symmetry Factor	Warning
1	Unknown	1	16,583	39456	2602	0,932	1,593	N/A	34744	8,000	2,032	
2	Unknown	1	19,808	4195968	160745	99,068	98,407	N/A	30661	N/A	2,278	

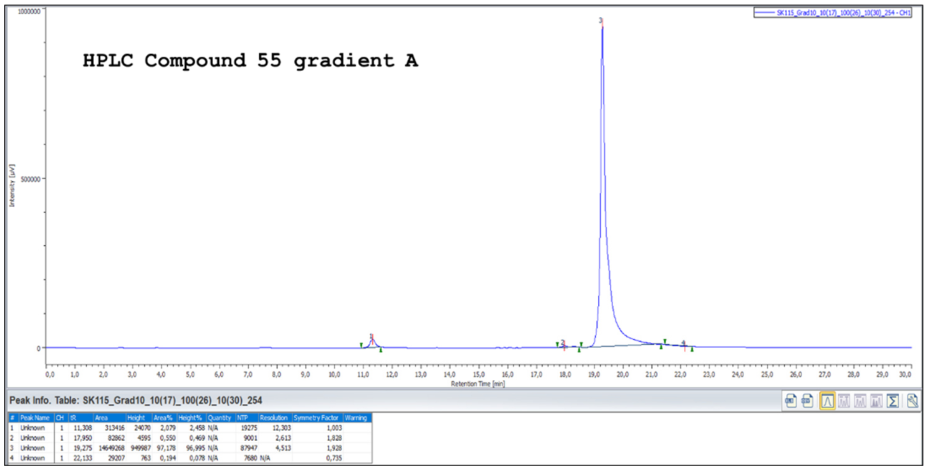

Peak Info. Table: SK115_Grad10_10(17)_100(26)_10(30)_254

#	Peak Name	CH	tR	Area	Height	Area%	Height%	Quantity	NTP	Resolution	Symmetry Factor	Warning
1	Unknown	1	11,308	313416	24070	2,079	2,458	N/A	19275	12,303	1,003	
2	Unknown	1	17,950	82862	4595	0,550	0,469	N/A	9001	2,613	1,828	
3	Unknown	1	19,275	14649268	949987	97,178	96,995	N/A	87947	4,513	1,928	
4	Unknown	1	22,133	29207	763	0,194	0,078	N/A	7680	N/A	0,735	

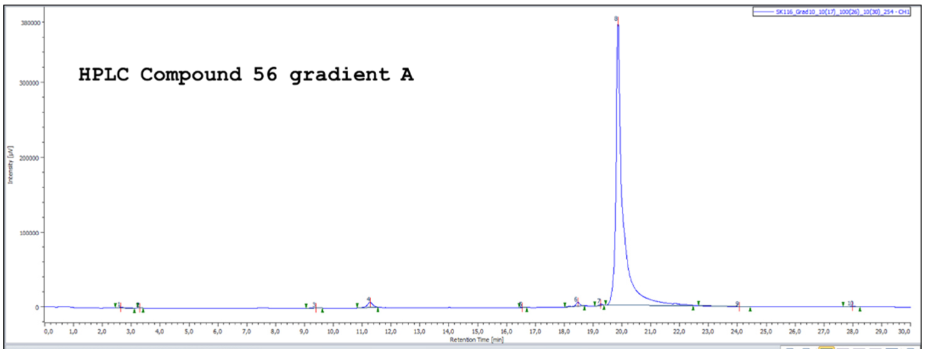

Peak Info. Table: SK116_Grad10_10(17)_100(26)_10(30)_254

#	Peak Name	CH	tR	Area	Height	Area%	Height%	Quantity	NTP	Resolution	Symmetry Factor	Warning
1	Unknown	1	2,633	11851	922	0,186	0,234	N/A	968	2,509	1,742	
2	Unknown	1	3,300	1624	240	0,025	0,061	N/A	4610	19,195	1,155	
3	Unknown	1	9,392	8002	499	0,125	0,126	N/A	7223	4,837	0,868	
4	Unknown	1	11,267	92654	6786	1,453	1,721	N/A	18067	20,326	0,890	
5	Unknown	1	16,550	3089	446	0,048	0,113	N/A	126728	9,363	1,064	
6	Unknown	1	18,458	56617	5425	0,888	1,376	N/A	109859	3,723	0,756	
7	Unknown	1	19,258	13078	1718	0,205	0,436	N/A	136998	2,494	0,857	
8	Unknown	1	19,850	6163748	378002	96,635	95,838	N/A	87995	2,874	2,057	
9	Unknown	1	24,058	22649	150	0,355	0,038	N/A	1300	2,370	0,626	
10	Unknown	1	27,975	5056	229	0,079	0,058	N/A	30107	N/A	0,882	

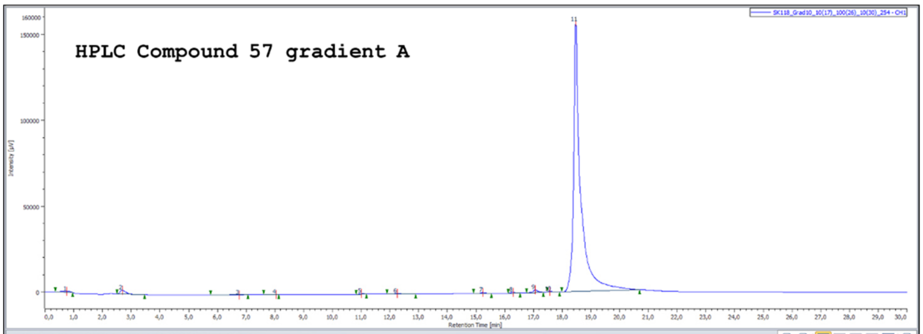

Peak Info. Table: SK118_Grad10_10(17)_100(26)_10(30)_254

#	Peak Name	CH	tR	Area	Height	Area%	Height%	Quantity	NTP	Resolution	Symmetry Factor	Warning
1	Unknown	1	0,750	16037	750	0,579	0,458	N/A	21	3,827	0,840	
2	Unknown	1	2,683	30298	2177	1,093	1,329	N/A	865	5,417	1,575	
3	Unknown	1	6,758	3517	77	0,127	0,047	N/A	559	1,664	0,642	
4	Unknown	1	8,042	198	12	0,007	0,007	N/A	6365	9,444	0,628	
5	Unknown	1	11,008	3196	379	0,115	0,231	N/A	37718	4,820	1,142	
6	Unknown	1	12,250	3671	270	0,132	0,165	N/A	28582	10,983	1,260	
7	Unknown	1	15,233	5897	519	0,213	0,317	N/A	57163	4,318	1,119	
8	Unknown	1	16,275	4792	520	0,173	0,317	N/A	80884	3,226	1,346	
9	Unknown	1	17,058	17682	1557	0,638	0,951	N/A	69897	2,080	1,097	
10	Unknown	1	17,558	5134	502	0,185	0,306	N/A	98263	3,517	2,140	
11	Unknown	1	18,458	2681422	157003	96,738	95,871	N/A	65190	N/A	2,001	

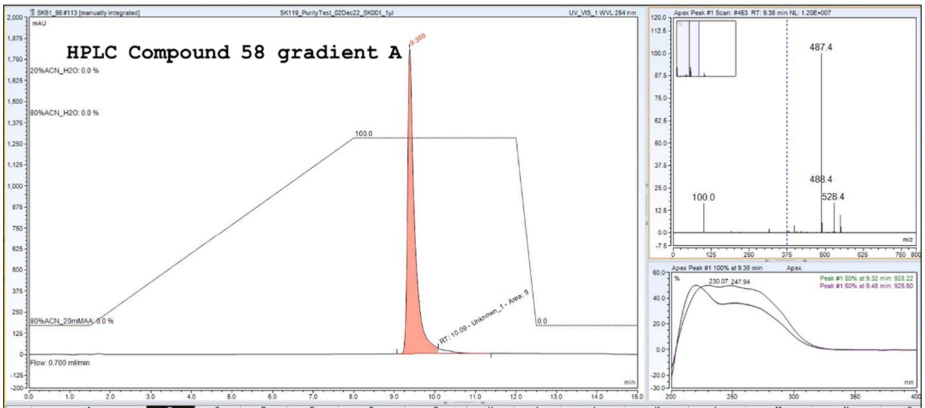

	A	B	C	D	E	F	G	H	I	J	K	L	M	N	O
1	Peak Name	Ret. Time	Area	Rel Area	Amount	Overall Ion Ratio	Quant. Ion	Conf. Ion #1	Ion Ratio #1	Ion Ratio #1	Ion Ratio #1	Conf.Ion #2	Ion Ratio #2	Ion Ratio #2	Ion Ra
2		min	mAU*min	%		Confirmation	m/z	m/z	(Expected)	(Observed)	Within Window	m/z	(Expected)	(Observed)	Within W
4		9.38	367	97.50	n.a.	Not confirmed	n.a.	n.a.	n.a.	n.a.	n.a.	n.a.	n.a.	n.a.	n.a
5	Unknown_1	10.09	9	2.50	n.a.	Not confirmed	n.a.	n.a.	n.a.	n.a.	n.a.	n.a.	n.a.	n.a.	n.a
6															

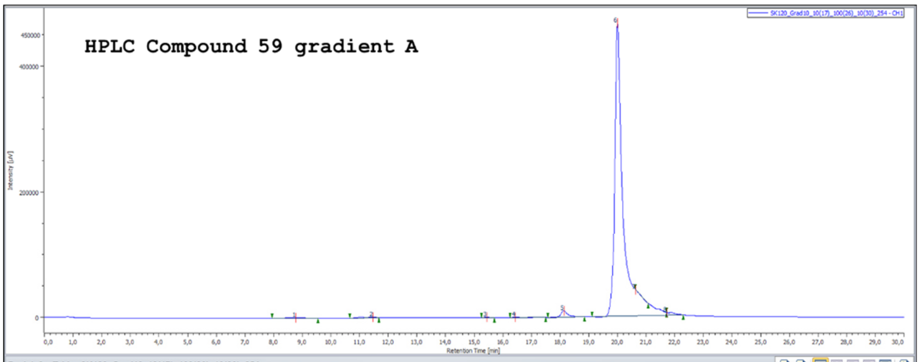

Peak Info. Table: SK120_Grad10_10(17)_100(26)_10(30)_254

#	Peak Name	CH	tR	Area	Height	Area%	Height%	Quantity	NTP	Resolution	Symmetry Factor	Warning
1	Unknown	1	8,775	38822	1197	0,385	0,247	N/A	1641	2,626	1,007	
2	Unknown	1	11,458	37986	1063	0,376	0,220	N/A	1502	5,241	0,642	
3	Unknown	1	15,433	7085	567	0,070	0,117	N/A	33306	2,965	1,144	
4	Unknown	1	16,433	11191	628	0,111	0,130	N/A	37794	4,799	3,659	
5	Unknown	1	18,117	168182	10242	1,667	2,118	N/A	39347	4,987	1,761	
6	Unknown	1	19,992	9749511	465987	96,617	96,357	N/A	42330	N/A	2,462	
7	Unknown	1	20,633	3	3	0,000	0,001	N/A	N/A	N/A	N/A	
8	Unknown	1	21,725	78150	3920	0,774	0,811	N/A	N/A	N/A	N/A	

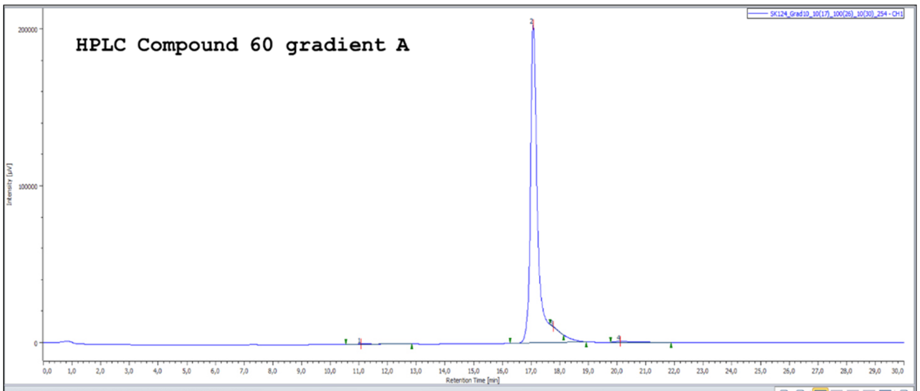

Peak Info. Table: SK124_Grad10_10(17)_100(26)_10(30)_254

#	Peak Name	CH	tR	Area	Height	Area%	Height%	Quantity	NTP	Resolution	Symmetry Factor	Warning
1	Unknown	1	11,058	22339	525	0,596	0,257	N/A	337	4,321	4,592	
2	Unknown	1	17,067	3690549	202690	98,508	99,232	N/A	32657	2,206	1,916	
3	Unknown	1	17,775	2343	258	0,063	0,126	N/A	71366	4,435	1,058	
4	Unknown	1	20,092	31229	784	0,834	0,384	N/A	10579	N/A	3,198	

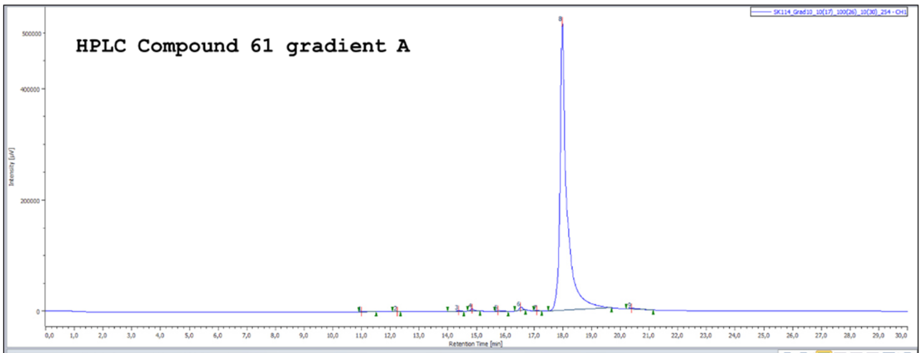

Peak Info. Table: SK114_Grad10_10(17)_100(26)_10(30)_254

#	Peak Name	CH	tR	Area	Height	Area%	Height%	Quantity	NTP	Resolution	Symmetry Factor	Warning
1	Unknown	1	11,000	8105	443	0,092	0,083	N/A	4913	3,174	2,962	
2	Unknown	1	12,242	801	142	0,009	0,027	N/A	97502	11,732	1,089	
3	Unknown	1	14,367	12014	1506	0,137	0,282	N/A	77511	2,177	1,157	
4	Unknown	1	14,833	39151	4145	0,447	0,775	N/A	70540	4,103	1,436	
5	Unknown	1	15,742	17352	1697	0,198	0,317	N/A	81514	3,767	1,932	
6	Unknown	1	16,533	48806	6378	0,557	1,193	N/A	108424	2,775	1,092	
7	Unknown	1	17,083	8820	1187	0,101	0,222	N/A	120715	3,606	1,280	
8	Unknown	1	17,975	8595171	517559	98,043	96,806	N/A	57753	4,849	1,857	
9	Unknown	1	20,392	36556	1579	0,417	0,295	N/A	13569	N/A	2,065	

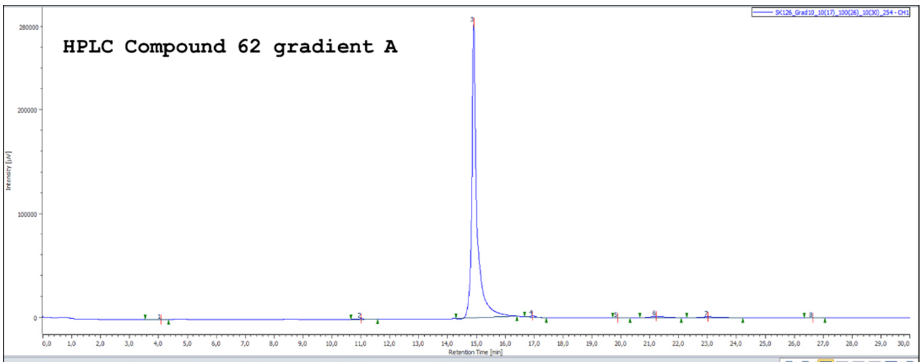

Peak Info. Table: SK126_Grad10_10(17)_100(26)_10(30)_254

#	Peak Name	CH	tR	Area	Height	Area%	Height%	Quantity	NTP	Resolution	Symmetry Factor	Warning
1	Unknown	1	4,092	1919	90	0,053	0,031	N/A	725	11,402	0,932	
2	Unknown	1	11,008	14581	646	0,402	0,224	N/A	5234	9,377	1,200	
3	Unknown	1	14,900	3505855	283974	96,619	98,277	N/A	71016	9,070	1,623	
4	Unknown	1	16,933	17765	1711	0,490	0,592	N/A	89899	8,131	1,268	
5	Unknown	1	19,883	2463	146	0,068	0,051	N/A	25133	1,951	1,686	
6	Unknown	1	21,217	41924	1234	1,155	0,427	N/A	9543	1,955	1,327	
7	Unknown	1	23,000	37942	892	1,046	0,309	N/A	9181	4,475	1,454	
8	Unknown	1	26,633	6102	259	0,168	0,090	N/A	25423	N/A	1,169	

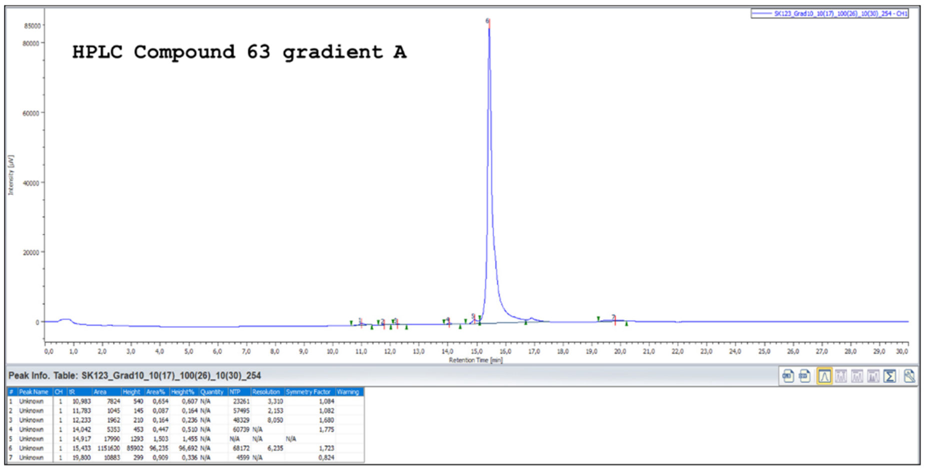

Peak Info. Table: SK123_Grad10_10(17)_100(26)_10(30)_254

#	Peak Name	CH	tR	Area	Height	Area%	Height%	Quantity	NTP	Resolution	Symmetry Factor	Warning
1	Unknown	1	10,983	7824	540	0,654	0,607	N/A	23261	3,310	1,084	
2	Unknown	1	11,783	1045	145	0,087	0,164	N/A	57495	2,153	1,082	
3	Unknown	1	12,233	1962	210	0,164	0,236	N/A	48329	8,050	1,680	
4	Unknown	1	14,042	5353	453	0,447	0,510	N/A	60739	N/A	1,775	
5	Unknown	1	14,917	17990	1293	1,503	1,455	N/A	N/A	N/A	N/A	
6	Unknown	1	15,433	1151620	85902	96,235	96,692	N/A	68172	6,235	1,723	
7	Unknown	1	19,800	10883	299	0,909	0,336	N/A	4599	N/A	0,824	

References

(1) Sheldrick, G. SHELXT - Integrated space-group and crystal-structure determination. *Acta Crystallographica Section A* 2015, *71* (1), 3.

(2) Sheldrick, G. Crystal structure refinement with SHELXL. *Acta Crystallographica Section C* 2015, *71* (1), 3.

(3) Önell, A.; Andersson, K. Kinetic determinations of molecular interactions using Biacore—minimum data requirements for efficient experimental design. *Journal of Molecular Recognition* 2005, *18* (4), 307.

(4) Matsumura, K.; Arai, N.; Hori, K.; Saito, T.; Sayo, N.; Ohkuma, T. Chiral Ruthenabicyclic Complexes: Precatalysts for Rapid, Enantioselective, and Wide-Scope Hydrogenation of Ketones. 2011, DOI:10.1021/ja202296w 10.1021/ja202296w.
